# Analysis of Metabolites and Gene Expression Changes Relative to Apricot (*Prunus armeniaca* L.) Fruit Quality During Development and Ripening

**DOI:** 10.3389/fpls.2020.01269

**Published:** 2020-08-19

**Authors:** Beatriz Ester García-Gómez, David Ruiz, Juan Alfonso Salazar, Manolo Rubio, Pedro José Martínez-García, Pedro Martínez-Gómez

**Affiliations:** Departamento de Mejora Vegetal, CEBAS-CSIC, Murcia, Spain

**Keywords:** apricot, RNA-Seq, qPCR, fruit quality, ripening, reference genomes, functional analysis, candidate genes****

## Abstract

Apricot (*Prunus armeniaca* L.) is a valuable worldwide agronomical crop, with a delicious fruit highlighted as a functional food with both nutritional and bioactive properties, remarkably beneficial to human health. Apricot fruit ripening is a coordinated developmental process which requires change in the expression of hundreds to thousands of genes to modify many biochemical and physiological processes arising from quality characteristics in ripe fruit. In addition, enhancing fruit and nutraceutical quality is one of the central objectives to be improved in the new varieties developed by breeding programs. In this study we analyzed the contents of main metabolites linked to the nutraceutical value of apricot fruits, together with the most important pomological characteristics and biochemical contents of fruit during the ripening process in two contrasted apricot genotypes. Additionally, the gene expression changes were analyzed using RNA-Seq and real time qPCR. Results showed that genes with differential expression in the biosynthetic pathways, such as phenylpropanoids, flavonoids, starch and sucrose and carotenoid metabolism, could be possible candidates as molecular markers of fruit quality characteristics for fruit color and soluble solid content. The gene involves in carotenoid metabolism *carotenoid cleavage dioxygenase 4*, and the gene *sucrose synthase* in starch and sucrose metabolism were identified as candidate genes in the ripening process for white skin ground color and flesh color and high soluble sugar content. The application of these candidate genes on marker-assisted selection in apricot breeding programs may contribute to the early selection of high-quality fruit genotypes with suitable nutraceutical values.

## Introduction

Apricot (*Prunus armeniaca* L.) is an ancient domesticated crop that has co-evolved with human civilization. This stone fruit species have been used for its edible fruits, also being highlighted as a functional food with both nutritional and bioactive properties, including anti-oxidant and anti-inflammatory activity (Sochor et al., 2010; Erdogan-Orhan and Kartal, 2011; Minaiyan et al., 2014). Today, apricots are commercially produced in 65 countries around the world. During the 2016/2017 season, the worldwide production of apricots increased reaching 4.25 million tons (http://www.fao.org/faostat/en/#home), Spain being the first top fresh apricot exporter worldwide, exporting 56 thousand tons (http://www.fepex.es/inicio.aspx). Apricot is a member of the *Prunus* genus inside the highly diverse Rosaceae family and seems phylogenetically closer to *P. armeniaca* than *P. persica* (Mowrey and Werner, 1990; Zhang et al., 2012; García-Gómez et al., 2018).

Apricot fruit ripening is a coordinated developmental process which requires change in the expression of hundreds to thousands of genes to modify many biochemical and physiological processes. Apricot fruit displays a high variability, giving rise to a great diversity in fruit size, shape, color, taste, aroma, firmness, and ripening date; most of these pomological characteristics are of interest for improving quality traits in apricot breeding programs. Fruit ripening leads to the breakdown of complex carbohydrates into sugars, reduces fruit firmness, color changes, decrease titratable acidity as well as an increase in flavor and aroma (Infante et al., 2008; Klee and Giovannoni, 2011). Understanding these mechanisms will enable implementing agronomical strategies that are more adaptable to climatic conditions and optimizing the selection of new apricot varieties with high quality and nutraceutical values. From the point of view of the consumers, these characteristics contribute to increase the attractiveness and acceptance of new apricot cultivars enriched in phenylpropanoids, carotenoids, and other nutraceutical compounds highly beneficial for human health (Machlin, 1995; Van den Berg et al., 2000; Bazzano et al., 2002).

The development and application of High-Throughput Sequencing (HTS) technologies and the existence of new methods of data analysis, which enable finding associations between genomic, transcriptomic and phenomics, have become the new tools applied by breeders to develop new improved varieties. The remarkable advances in computational theory and bioinformatics algorithms have greatly accelerated the implementation of this technology, significantly expanding the scope of studied species. As a result, during the last few years, up to 450 plant genomes were sequenced (October 2019, http://www.ncbi.nlm.nih.gov). From these genomes, 93 are assembled and annotated in the Kyoto Encyclopedia of Genes and Genomes (KEGG), KEGG Orthology (KO), ENZYME, Pathway and InterPro database (Phytozome version 12.1.6, http://phytozome.jgi.doe.gov), including the most important crops with high commercial value as maize, potato, rice, or wheat (Jiao and Schneeberger, 2017).

Another point of attention about the application of HTS technologies to plant breeding programs remains to address the dynamic and adaptive aspect of gene expression over time, and location undergoes in different scenarios because of the environment effect, developmental stage, tissue/organ location, and genotype. Quantitative and qualitative changes in gene expression are studied by transcriptomic disciplines, mRNA sequencing (RNA-Seq) being the best method for measuring and comparing gene expression levels. Although RNA-Seq has become a widely applied analytical technique, there is no consensus on which pipeline is the most appropriate for the analysis of RNA-Seq experiments. The final determination as to which is the best depends on the strategy adopted during the experimental design according to the initial hypothesis or the objectives (Conesa et al., 2016). If the studied organism genome or transcriptome is available, it will be used to identify the transcripts by mapping. On the other hand, the analysis of transcriptomes of non-model organisms whose genomic or transcriptomic sequences are not publicly available can be addressed using the genomes or transcriptomes of phylogenetically related organisms or by performing a *de novo* assembly and gene annotation (Surget-Groba and Montoya-Burgos, 2010). The application of quality controls during each of the phases of the analysis guarantees both the reproducibility and the reliability of the results obtained (MacManes, 2014).

To date, the dynamic changes in gene expression during fruit ripening process have been studied by whole transcriptome sequencing using HTS technologies in many related apricot species from *Prunus* genus including *P. persica* (Zhang et al., 2010; Wang et al., 2013; Pan et al., 2015; Sanhueza et al., 2015; Zhou et al., 2015; Pan et al., 2016; Wu et al., 2017; Ye et al., 2019), *P. salicina* (Kim et al., 2015b; Fang et al., 2016), *P. armeniaca* (Jo et al., 2015; Zhang et al., 2017b; Zhang et al., 2019), *P. mume* (Du et al., 2013; Xu et al., 2014), and *P. avium* (Alkio et al., 2014; Wei et al., 2015)

The objective of this study was the analysis of gene expression changes of fruits in two contrasted apricot genotypes during development and ripening process by using RNA-Seq and qPCR to identify candidate genes responsible for the fruit differences found between the two assayed genotypes in relation to the pomological characteristics and biochemical and metabolite contents.

## Materials and Methods

### Plant Material and Experimental Design

**Figure 1:**
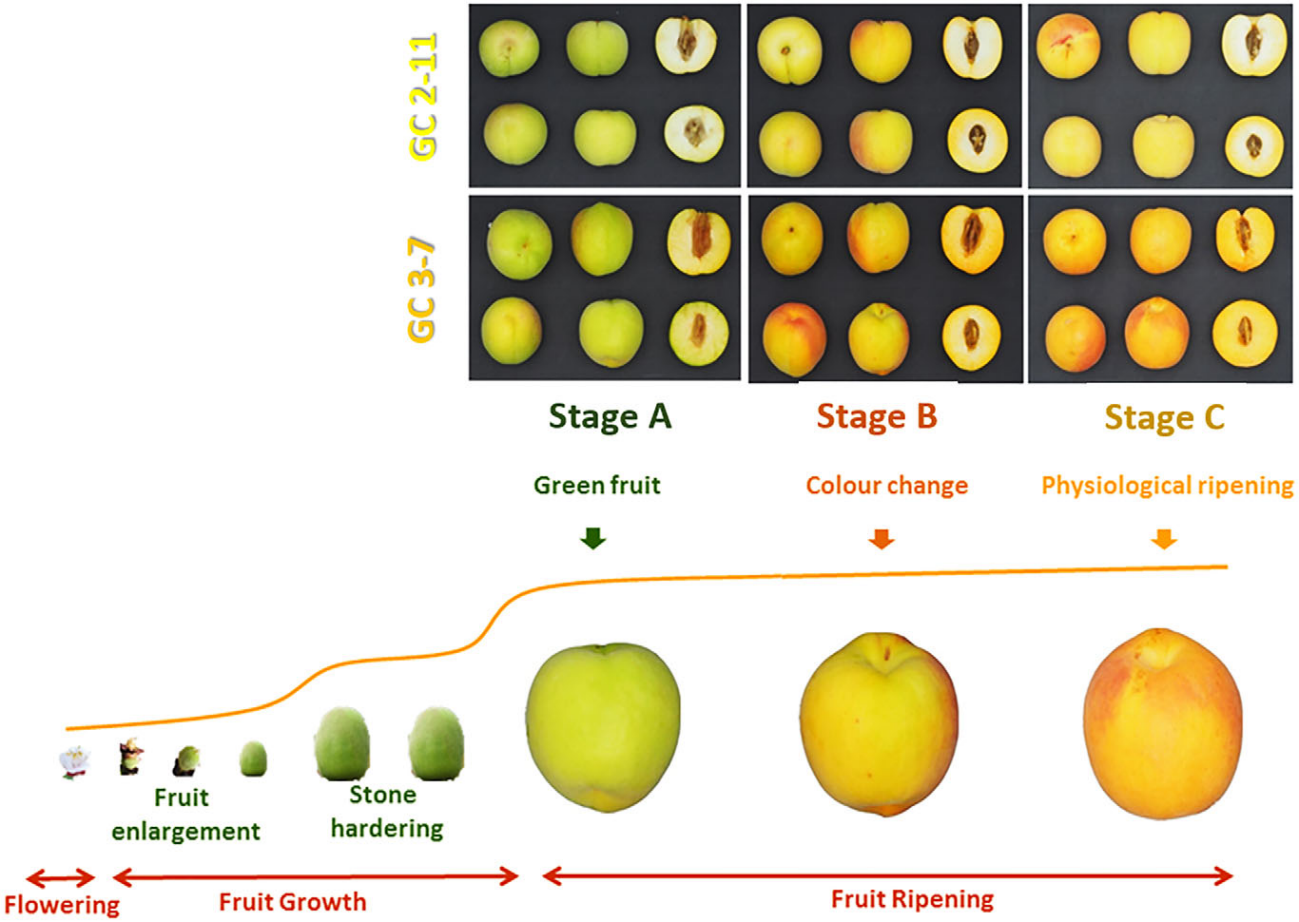
Fruit pictures from *P. armeniaca* fruit genotypes ‘GC 2-11’ and ‘GC 3-7’ analyzed in three different ripening stages including green fruit (Stage A), during color change (Stage B), and at physiological ripening (Stage C). Pictures were taken in a cold chamber illuminated with white leds. The photographic camera was an Olympus Pen Mini E-PM2 with 14–42 mm focus lens.

Plant material consisted on two apricot genotypes ‘GC 2-11’ and ‘GC 3-7’, both selections obtained from the cross between the North American cultivar ‘Goldrich’ (G) and the Spanish cultivar ‘Currot’ (C) (García-Gómez et al., 2018). These apricot releases showed contrasted fruit quality characteristics. ‘GC 2-11’ is self-compatible, early blooming, and has an intermediate-sized oblong fruit with yellow skin, red blush, and yellow flesh color. It has high soluble solid content and a low ethylene production rate. ‘GC 3-7’ is also self-compatible, early blooming, and has an intermediate-sized oblong fruit with orange skin, intense red blush, and light orange flesh color. It has a low-medium total soluble solid content and a medium ethylene production rate. The sampling was carried out the epicarp (including the pericarp and the mesocarp) due to the importance of considering all the edible fruit for assessing the supplementation of health promoter compounds in the diet. Besides, in the case of apricots, it is imperative to emphasize that peel and pulp are consumed together as an edible portion in contrast with other fruits such as peaches. These apricot selections were cultivated in the same experimental orchard of CEBAS-CSIC at Cieza (Murcia, South-East Spain, 241 m above sea level, lat. 38°16′N, long. 1°16′W) according to standard apricot orchard management. Ten fruits of each genotype were collected at three different ripening stages before stone hardening from both genotypes for RNA-Seq (during the first year of study) and qPCR analysis (during the second year) based on their skin ground color and firmness: green fruit (Stage A), during color change (Stage B), and at physiological ripening (Stage C) (**Figure 1**).

### Evaluation of Pomological Characteristics and Biochemical Contents

Pomology characteristics were analyzed including physical characterization (fruit weight, stone weight, skin ground color, flesh color, blush color, and firmness), biochemical compounds (total soluble solids, titratable acidity, and metabolite content), total chlorophyll and carotenoid content, CO_2_ and ethylene release. First, ten fruits were collected at three different ripening stages (green fruit, during color change, and at physiological ripening) of both genotypes (‘GC 2-11’ and ‘GC 3-7’) in 2016 for the evaluation of fruit quality characteristics. Fruit and stone weight was measured using a Blauscal digital balance (model AH-600), with an accuracy of 0.01 g. Skin ground color, blush color, and flesh color were determined with a Minolta Chroma Meter (CR-300; Minolta, Ramsey, NJ, USA) tri-stimulus color analyzer calibrated to a white porcelain reference plate using a CIELAB scale with color space coordinates L*, a*, and b*. The measure used to assess color was the Hue angle [H° = arctangent (b*/a*)], determined around the equatorial fruit (Brown and Walker, 1990). We take into account that values above 90 are closer to white, between 80 and 90 to yellow, 75 and 80 to light orange, 70 and 75 to orange, and below 70 the color tends to be more reddish. Firmness was quantified using a Lloyd press (model LR10K; Fareham, Hants, UK) by a compression test in Newton (N). Total soluble solid content (TSS) was measured as percentage using a hand-held refractometer (ATAGO Co. LTD., Tokyo, Japan). Finally, the titratable acidity (TA) was expressed as the predominant organic acid, malic acid (g/100 ml). TA was evaluated by titrating 2 g of sample diluted in 30 ml of distilled water with 0.1 mol l-1 NaOH to pH 8.1 by an automatic titration system. On the other hand, total chlorophyll (*a* and *b*) and carotenoid contents were determined by the method of Nagata and Yashita (1992) from a pool of ten fruit pericarps at three ripening stages (green fruit, during color change, and at physiological ripening) of both genotypes (‘GC 2-11’ and ‘GC 3-7’) in 2017. Three biological replicates for each ripening stage and genotype were analyzed. The dry residue from the lyophilized powder was dissolved in acetone:hexane (2:3) in a 1:10 w/v relation, centrifuged at 3,000 g for 10 min in a refrigerated centrifuge at 4°C and spectrophotometrically determined at 663, 645, 505, and 453 nm. Respiration rate and ethylene release were measured for each sample by placing two or three whole fruits in a sealed glass jar at 20°C per triplicate. Ethylene concentrations in the glass jar were sampled and monitored with a Perkin Elmer Autosystem gas chromatograph equipped with a thermal conductivity detector (TCD) and a flame ionization detector (FID). Respiration rate was determined by measure CO_2_ sampled and monitored by using a gas analyzer (Horiba Via 510, Irving, USA). Samples of 1 ml of headspace gas were taken from each glass jar with a calibrated syringe.

### Evaluation of Metabolite Contents

Metabolite content was determined by nuclear magnetic resonance spectroscopy (^1^H-NMR) from a pool of ten fruits (pericarp portion) at three ripening stages (green fruit, during color change, and at physiological ripening) of both genotypes (‘GC 2-11’ and ‘GC 3-7’) in 2016. Portions from this pool (10 g) were frozen in liquid nitrogen, lyophilized, and powdered. The dry residue was dissolved in 0.75 ml of CD_3_OD and 0.75 ml of D_2_O phosphate buffer containing sodium 3-trimethylsilyl-[2,2,3,3-D4]-propionate (TSP) (0.1% w/w) as internal standard (150 mM), vortex 1 min, centrifuged at 17,000 *g* 5 min and preserve the supernatant at room temperature. The ^1^H-NMR spectra of aqueous apricot extracts were recorded at 27°C on an AVANCE III HD 500 MHz, CryoProbe Prodigy BBO (Capitani et al., 2012). Carbohydrates (sucrose, glucose, xylose, fructose, and *myo*-inositol), organic acids (fumarate, malate, succinate, citrate, and formate) and precursors of phenylpropanoids (chlorogenate, epicatechin, and methyl nicotinate) were measured. Three biological replicates for each ripening stage and genotype were analyzed.

### Phenotype Data Analysis

Statistical analysis was performed in R version 3.5.1. Levene Test was applied for homoscedasticity, Shapiro–Wilk for normal distribution and Kruskal–Wallis Sum Rank Test as an alternative non-parametric ANOVA test. The statistical significance threshold was set at *p-*value < 0.05. Principal Component Analysis (PCA) was also performed over the phenological and metabolite content data to reduce the dimensionality retaining, thus identifying related groups, trend, or outliers. PCA graph was computed and visualized in R with the package ggbiplot (Wickham, 2016). A correlation matrix by the Spearman method was executed to resolve the correlation relationships between the quality traits analyzed.

### RNA Isolation and High-Throughput Sequencing

A pool of ten fruits (pericarp) at three ripening stages (green fruit, during color change, and at physiological ripening) and genotype (‘GC 2-11’ and ‘GC 3-7’) were collected during the first year of study, frozen in liquid nitrogen and stored at −80°C. Total RNA was extracted using a modified PowerPlant RNA Isolation Kit^®^ (Qiagen, Hilden, Germany) treated with DNAse On-Spin Column DNase I Kit (Qiagen, Hilden, Germany). Finally, RNA was concentrated and purified with UltraClean Plant RNA Isolation Kit (Qiagen, Hilden, Germany). The purity and quantity of total RNA samples were assessed using a NanoDrop^®^ One Spectrophotometer (Thermo Scientific, Wilmington, USA) and normalized at the same concentration (0.5 µg, 50 ng/µl). Integrity was established by capillary electrophoresis in 2100 Bioanalyzer System (Agilent, Santa Clara, U.S.A.) (Schroeder et al., 2006). Three biological replicates were assayed for each comparison of genotype and stage. RNA samples were sent to Sistemas Genómicos (Valencia, Spain) for library preparation and RNA sequencing. RNA-Seq libraries are prepared from total RNA using poly(A) enrichment of the mRNA. mRNA enriched libraries were sequenced using the HiSeq™2000 Sequencing System platform (Illumina, San Diego, CA, USA), with two technical replicates each sample. The datasets generated for this study can be found in the NCBI SRA repository as a Bioproject entitled ‘Prunus armeniaca fruit ripening process’, with the accession number PRJNA562811 (https://www.ncbi.nlm.nih.gov/bioproject/562811). The quality control of raw sequencing libraries obtained was analyzed with FastQC version 0.11.7 (Andrews, 2010) (https://www.bioinformatics.babraham.ac.uk/projects/fastqc/). Trimmomatic version 0.38 (Bolger et al., 2014) (http://www.usadellab.org/cms/?page=trimmomatic) was used for trimming adapter and filter low-quality sequences (Phred score < 30) of FASTQ files.

### Transcriptome Analysis and Mapping

Reference mei (*P. mume_*V1) (Zhang et al., 2012), peach (*P. persica_* V2) (Verde et al., 2017), and apricot (*P. armeniaca_*V1) (Jiang et al., 2019) genomes and transcriptomes were simultaneously used as reference sequences for read analysis and mapping our candidates genes. However, for functional candidate gene analysis, studies were focused on mei and apricot reference genomes. Indeed, a synteny between reference genomes was plotted by dot-plot graph executed in D-Genies webpage (Cabanettes and Klopp, 2018) (http://dgenies.toulouse.inra.fr/) to certify the validity of this step. For the backward step, the annotation of *P. armeniaca* was download from GDR (rosaceae.org), having blast information of the whole list of genes (mrna) of the species. The file was parsed, and the already identified IDs of *P. mume* and *P. persica* in the first step were used as identifier to get the list of genes from *P. armeniaca* associated with them. In the case of genes not identified, their sequences were mapped using gmap (Wu and Watanabe, 2005) to the reference genome of apricot and physical position (coordinates), and candidates genes were extracted by a custom python script. If no IDs were identified, at least physical position of these candidates genes are known. Doing this second step avoid to re-do the whole analysis, taking advances of the new resources and allowing also to validate the obtained results, as can been observed. High-quality sequencing libraries were mapped to the references genomes and transcriptomes of *P. persica* and *P. mume* with Tophat version 2.1.1 (Kim et al., 2013) (https://ccb.jhu.edu/software/tophat/index.shtml) and HISAT version 2.1.0 (Kim et al., 2015a) (https://ccb.jhu.edu/software/hisat2/index.shtml). Each genome was indexed with Bowtie version 2.3.4.1 (Langmead and Salzberg, 2012) (https://sourceforge.net/projects/bowtie-bio/). Finally, gene quantification and count matrix construction were performed with featureCounts (Liao et al., 2014) (http://bioinf.wehi.edu.au/featureCounts/). The results obtained were normalized to Transcript Per Million (TPM) (Li et al., 2010). In addition, representation of nucleotide abundance and other statistical data on mapped and unmapped reads in Sequence Alignment Map and Binary Alignment Map (SAM and BAM files) were analyzed by using SAMStat version 1.5.1 (Lassmann et al., 2010) (http://samstat.sourceforge.net). The index used to determine the quality of alignment and assembly was the Mapping Quality Score (MAPQ) (Li et al., 2008), which quantify the probability of a misplaced read. The optimum MAPQ value is considered to be greater than or equal to 30. SAMtools version 1.8 (Li et al., 2009) (http://samtools.sourceforge.net/) is the tool used to transform, index and sort the files generated by the mappers according to the needs of the protocol.

### Differentially Expressed Genes Analysis

Several statistical packages developed in R version 3.5.1 were employed for Differentially Expressed Genes Analysis (DEGs). For DEG analysis were applied five different statistical packages setting on default parameters: edgeR version 3.24.0 (Robinson et al., 2010), baySeq version 2.16.0 (Hardcastle and Kelly, 2010), EBSeq version 1.22.0 (Leng et al., 2015), NOISeq version 2.26.0 (Tarazona et al., 2015) and DESeq2 version 1.22.1 (Love et al., 2014). In most of the analysis Trimmed Mean of M-values (TMM) (Robinson and Oshlack, 2010) were applied, except for EBSeq, where median normalization was applied. As biological replicates, we grouped the samples from each genotype (three replicates each ‘GC 2-11’ and ‘GC 3-7’) and ripening stage (two replicates each stage A, stage B and stage C). The DEG analysis was filtered by 0.1 false discovery rate (FDR) (Benjamini and Hochberg, 1995) and the DEGs obtained in each method were merged in a consensus result (Costa-Silva et al., 2017). The consensus result was represented as Venn diagrams done with the web application Venn Diagram (http://bioinformatics.psb.ugent.be/webtools/Venn/).

### Gene Annotation, Enrichment Analysis, and Pathway Visualization

Gene Annotation was performed with BiomaRt version 2.38.0 in R (Durinck et al., 2009), which provides easy access to public databases repositories as Ensembl, KEGG, KO, Uniprot, Pfam, Panther, Interpro and Gene Ontology (GO). We also added the best hits found in biomaRt for Arabidopsis (*Arabidopsis thaliana* L.) and peach, by Basic Local Alignment Search Tool (BLAST) (https://blast.ncbi.nlm.nih.gov/Blast.cgi). Enrichment Analysis was plotted with Web Gene Ontology Annotation Plot (WEGO) (http://wego.genomics.org.cn/) web-based tool (Ye et al., 2018) was used for visualizing, comparing, and plotting GO annotation results in a bar chart. Singular Enrichment Analysis (SEA) in AgriGO version 2.0 (Tian et al., 2017) was used to plot GO hierarchical graph containing all statistically significant terms. Broader representation of GO terms was performed on GO Consortium web-based platform (Ashburner et al., 2000; Consortium, 2004). DEGs were plotted on their KEGG biosynthetic pathways with GAGE version 2.32.0 (Luo et al., 2009), and visualized with Pathview version 1.22.0 (Luo and Brouwer, 2013), both R packages.

### Weighted Gene Co-expression Network Analysis

For clustering DEGs, we perform Weighted Gene Co-expression Network Analysis (WGCNA) with the R package WGCNA version 1.66 (Zhang and Horvath, 2005; Langfelder and Horvath, 2008). WGCNA is useful for describing the correlation patterns among gene expression across RNA-Seq experiments. The eigengene modules (ME) are obtained using automatic network construction function blockwise. The clustered gene trees were created following the average method (Wilks, 2011). Cluster analysis by gene expression is typically the first step in data analysis, reducing the complexity and dimensionality of the data, predict functions or identify shared regulatory mechanism based on specific features. So, the genes within a ME have similar expression pattern than the genes in different modules, revealing the natural data structures and gaining some initial insights regarding data distribution (Jiang et al., 2004).

### Complementary Gene Expression Analysis by quantitative real-time PCR

RNA-Seq experiment was validated by using RT-qPCR in a complementary experiment. New plant material collected during the second year from a pool of ten fruit pericarps, with three replicates for each ripening stage and genotype. Total RNA was extracted following the same protocol detailed above (RNA isolation and High-Throughput Sequencing). Specific primers were designed with Primer3 software based on apricot sequencing libraries (**Supplementary Data Sheet 1**) or from bibliography references as *carotenoid cleavage dioxygenase 4* (*CCD4*), *sorbitol dehydrogenase* (*SDH*), *alcohol acyltransferase* (*AAT*), *sucrose synthase* (*SS*) and *β-galactosidase* (*BGAL*) (Adami et al., 2013; Pirona et al., 2013). The cDNA was synthesized with SuperScript III Reverse Transcriptase (Thermo Fisher Scientific). RT-qPCR experiments were conducted in One Step Plus real-time PCR system (Applied Biosystems). For all RT-qPCR reactions, a 10 μl mix was made including 5 μl Power SYBR^®^ Green PCR Master Mix (Applied Biosystems), 0.5 μl of each primer (5 μM) and 2 µl of cDNA (2.5 ng/µl). The genes from peach *18S rRNA* (*S18*) (Rasori et al., 2002); and plum *cyclophilin 1* (*CYP1*) *ribosomal protein L12* (*RPL12*) and *ubiquitin* (UBI) (Niu et al., 2014) were analyzed as candidate reference genes by RefFinder web-based tool (Xie et al., 2012) (no longer available). Analysis was run with default settings. Amplification conditions were 10 min at 95°C, followed by 40 cycles of 15 seconds (s) at 95°C, 1 min at 60°C, and for melt curve 15 s at 95°C, 1 min at 60°C and increasing 0.3°C till 0.15 s at 95°C. Normalized Relative Quantification (NRQ) for the genes of interest was calculated using the modified 2^–ΔΔCt^ method (Pfaffl, 2001; Vandesompele et al., 2002). Correlations between TPM and NRQ, and TPM and metabolite content were calculated using the Pearson correlation coefficient (Udvardi et al., 2008). Three biological replicates and two technical replicates were assayed.

## Results

### Evolution of Pomological Characteristics and Biochemical and Metabolite Contents of Apricot Fruit During the Ripening Process

The results obtained from the evaluation of pomological characteristics and biochemical and metabolite contents in fruits for both apricot genotypes at the three ripening stages were analyzed to identify significant statistical differences between genotypes and stages (**Tables 1** and **2**).

**Table 1:** Evaluation of fruit pomological characteristics and biochemical contents in the two apricot genotypes assayed ‘GC 2-11’ and ‘GC 3-7’ at three ripening stages including green fruit (Stage A), during color change (Stage B), and at physiological ripening (Stage C).

Stage	Stage A		Stage B		Stage C	
Genotype	‘GC 2-11’	‘GC 3-7’	‘GC 2-11’	‘GC 3-7’	‘GC 2-11’	‘GC 3-7’
Fruit weight (g)	52.12 ± 2.18	61.37 ± 2.29	54.98 ± 3.85	69.59 ± 3.91	59.26 ± 3.30	83.85 ± 4.46
Stone weight (g)	4.22 ± 0.22	5.04 ± 0.31	3.57 ± 0.06	4.56 ± 0.22	3.35 ± 0.11	4.21 ± 0.08
Skin ground color (H°)	111.52 ± 1.84	111.21 ± 1.69	102.72 ± 2.49	89.79 ± 6.56	95.20 ± 2.88	77.07 ± 2.93
Blush color (H°)	97.9 ± 3.59	90.8 ± 16.4	81.26 ± 13.3	64.42 ± 7.21	74.10 ± 9.00	63.88 ± 5.60
Flesh color (H°)	105.33 ± 2.84	105.96 ± 3.63	94.18 ± 18.4	86.13 ± 14.50	87.40 ± 3.21	76.69 ± 1.63
Firmness (N)	232.99 ± 36.21	288.11 ± 24.40	76.70 ± 11.05	114.80 ± 19.63	39.07 ± 8.56	40.10 ± 24.94
Total soluble solids (%)	12.3 ± 0.2	6.8 ± 0.2	14.8 ± 0.2	11.9 ± 0.4	14.1 ± 0.3	12.8 ± 0.8
Titratable acidity (malic acid g/100 ml)	2.11 ± 0.01	2.06 ± 0.04	1.98 ± 0.01	2.04 ± 0.10	1.78 ± 0.02	1.89 ± 0.05
CO2 (µl/kg h)	13.46 ± 1.49	9.12 ± 1.37	10.58 ± 5.84	11.42 ± 3.22	7.08 ± 2.44	15.08 ± 5.05
Ethylene (mg/kg h)	–	–	1.15 ± 1.07	1.44 ± 0.91	0.15 ± 0.10	37.86 ± 19.90
Chlorophyll a (mg/g FW)	5.34 ± 1.36	19.33 ± 0.70	1.95 ± 0.01	2.81 ± 0.28	0.29 ± 0.24	0.38 ± 0.21
Chlorophyll b (mg/g FW)	0.21 ± 0.34	1.5 ± 0.06	0.17 ± 0.20	0.11 ± 0.33	0.59 ± 0.42	0.10 ± 0.46
Carotenoids (mg/g FW)	6.22 ± 0.23	33.02 ± 5.81	59.47 ± 0.98	37.79 ± 2.17	15.88 ± 0.98	57.61 ± 1.29

Three biological replicates were assayed.

**Table 2:** Evaluation of metabolite contents recorded in ^1^H-NMR spectra in the two apricot genotypes assayed ‘GC 2_11’ and ‘GC 3_7’ 7’at three ripening stages including green fruit (Stage A), during color change (Stage B), and at physiological ripening (Stage C).

Stage	Stage A		Stage B		Stage C	
Genotype	‘GC 2-11’	‘GC 3-7’	‘GC 2-11’	‘GC 3-7’	‘GC 2-11’	‘GC 3-7’
Sucrose	19.370 ± 2.790	2.638 ± 0.098	36.494 ± 1.499	31.387 ± 5.782	40.128 ± 1.379	46.965 ± 10.990
Glucose	3.017 ± 0.407	15.588 ± 0.914	3.530 ± 0.083	15.951 ± 2.776	5.639 ± 0.123	11.128 ± 2.346
Xylose	0.185 ± 0.044	0.210 ± 0.017	0.251 ± 0.011	0.250 ± 0.027	0.309 ± 0.014	0.225 ± 0.053
Fructose	0.317 ± 0.042	3.440 ± 0.463	0.510 ± 0.214	1.346 ± 0.647	0.527 ± 0.033	1.521 ± 0.737
*Myo*-Inositol	0.292 ± 0.040	0.384 ± 0.023	0.364 ± 0.013	0.461 ± 0.081	0.402 ± 0.020	0.404 ± 0.101
Fumarate	0.006 ± 0.001	0.004 ± 0.000	0.008 ± 0.000	0.009 ± 0.001	0.011 ± 0.000	0.014 ± 0.003
Malate	3.754 ± 0.852	4.761 ± 0.373	3.086 ± 0.469	4.387 ± 0.301	3.023 ± 0.069	3.544 ± 0.611
Succinate	0.019 ± 0.002	0.008 ± 0.005	0.009 ± 0.001	0.026 ± 0.003	0.009 ± 0.011	0.016 ± 0.004
Citrate	5.309 ± 2.299	11.106 ± 0.141	2.854 ± 0.155	13.641 ± 2.061	3.456 ± 0.338	6.943 ± 2.425
Formate	0.0001 ± 0.001	0.006 ± 0.001	0.005 ± 0.000	0.001 ± 0.001	0.005 ± 0.001	0.001 ± 0.001
Chlorogenate	0.049 ± 0.003	0.128 ± 0.022	0.045 ± 0.006	0.199 ± 0.032	0.056 ± 0.004	0.077 ± 0.027
Epicatechin	0.009 ± 0.007	0.014 ± 0.0001	0.009 ± 0.001	0.018 ± 0.001	0.014 ± 0.004	0.016 ± 0.006
Methyl nicotinate	0.012 ± 0.006	0.023 ± 0.009	0.013 ± 0.004	0.019 ± 0.007	0.011 ± 0.006	0.016 ± 0.004

Metabolites identified were expressed in mg/g fresh fruit weight (FW). Three biological replicates were assayed.

Evaluation of pomological traits evidenced the fruit color of ‘GC 2-11’ (yellow skin, red blush, and yellow flesh color) and ‘GC 3-7’ (orange skin, intense red blush and light orange flesh color) with a bigger fruit and stone. Values of firmness, however, were similar at the end of the ripening process (**Table 1**). In addition, biochemical evaluation showed a higher soluble solid content and a low ethylene production rate in ‘GC 2-11’ with a higher content of carotenoids in ‘GC 3-7’. Values of acidity and chlorophyll contents, however, were similar at the end of the ripening process (**Table 1**).

On the other hand, evolution of metabolite contents showed the clear increase of sucrose, glucose, xylose, fructose and myo-inositol associated with the decrease of fumarate, malate, succinate, and citrate in both genotypes during the ripening process mainly between the stages B and C. When genotypes are compared, a higher content of chlorogenate is shown in all the assayed stages in ‘GC 3-7’ in comparison with ‘GC 2-11’ (**Table 2**).

Levene Test showed no homoscedasticity and Shapiro–Wilk Test showed no normal distribution of the pomological, biochemical and metabolite data, hence we applied the non-parametric Kruskal–Wallis Rank Sum Test with a threshold of *p*-value < 0.05 for significant statistical differences. Skin ground color, blush color and flesh color, stone weight, fruit weight, titratable acidity, and ethylene release showed statistical differences between genotypes and ripening stages (**Supplementary Table 1**). Glucose, fructose, malate, citrate, formate, chlorogenate, and methyl nicotinate show statistical differences only between genotypes. Sucrose, xylose, fumarate, chlorophyll *a*, soluble solids, carotenoid content, and firmness showed statistical differences only between ripening stages. CO_2_ releases, *myo*-inositol, succinate, epicatechin, chlorophyll *b* show no statistical differences between genotypes or ripening stages (**Supplementary Table 2**). In addition, Spearman correlation matrix (**Supplementary Figure 1** and **Supplementary Data Sheet 2**) showed a positive correlation between skin ground color, flesh color, blush color, chlorophyll content and firmness, and between sucrose and soluble solid content, while a negative correlation between sucrose and soluble solids with skin ground color, flesh color, blush color, firmness and chlorophyll content. Glucose also showed a positive correlation with phenylpropanoids (chlorogenate, epicatechin, and methyl nicotinate). Fumarate displayed a negative correlation with firmness, skin ground color, blush color, flesh color, while a positive correlation with soluble solids was also observed.

**Figure 2:**
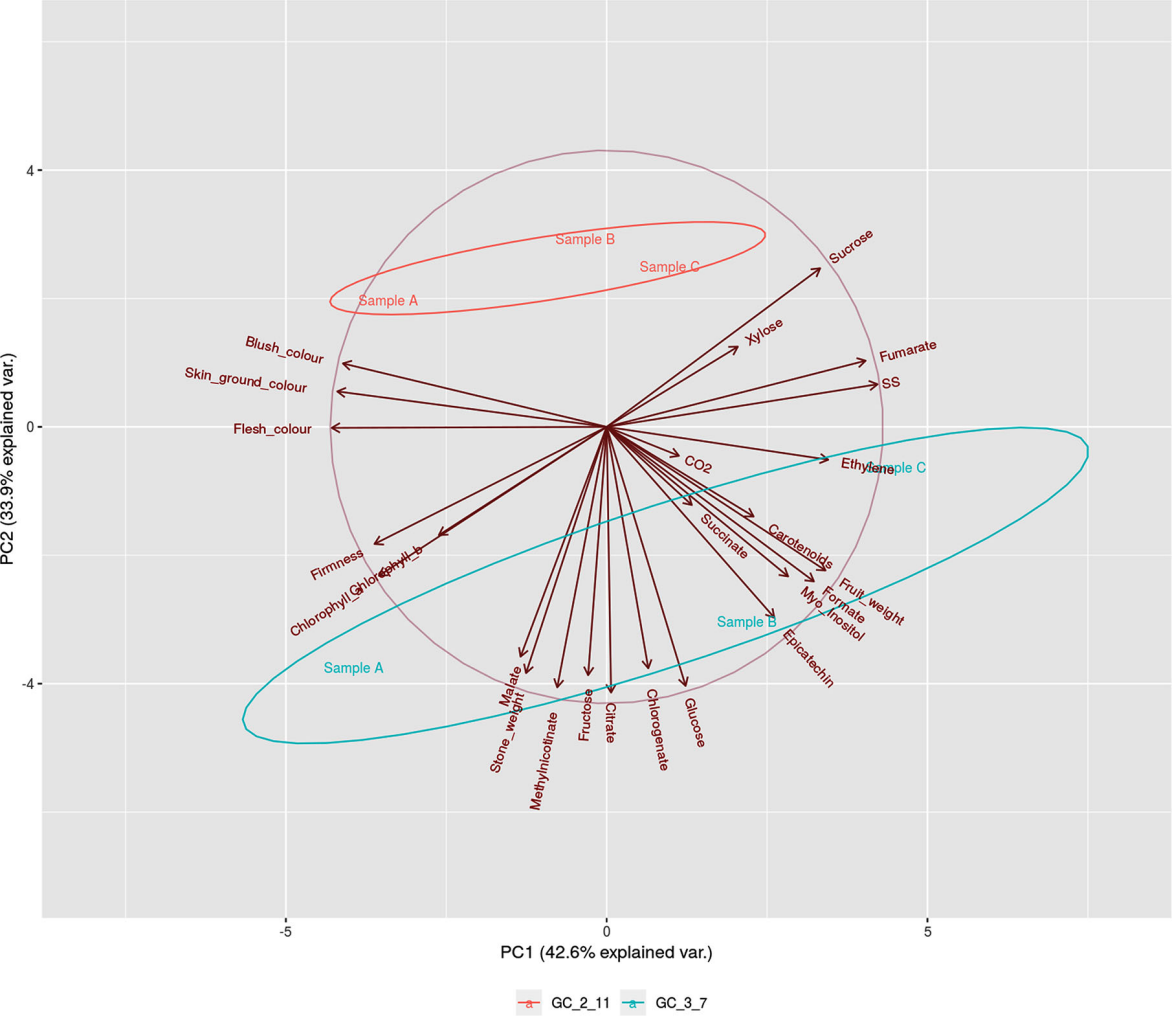
PCA biplot of phenological traits (fruit weight, stone weight, skin ground color, blush color, flesh color and firmness), biochemical (Total soluble solids, Titratable acidity, CO2, Ethylene, Chlorophyll *a*, Chlorophyll *b*, and Carotenoids) and metabolite (Sucrose Glucose, Xylose, Fructose, Myo-Inositol, Fumarate, Malate, Succinate, Citrate, Formate, Chlorogenate, Epicatechinand Methyl nicotinate) contents during ripening process [green fruit (Sample A), during color change (Samble B), and physiological ripening (Sample C)] in the two genotypes of *P. armeniaca* ‘GC 2-11’ and ‘GC 3-7’. Ellipses round samples which belongs to the same group with a 0.68 of probability (default settings for continuous variables). Three biological replicates were assayed to evaluate each phenological, biochemical and metabolite parameter.

On the other hand, PCA representation of phenological traits and metabolite content (**Figure 2**) showed the relationship between variables after logarithmic transformation of the data. The main principal components obtained explained the variance of 42.6% for Principal Component 1 (PC1) and 33.9% for PC2. Samples by genotype cluster together. Genotype ‘GC 2-11’ showed a positive correlation, while ‘GC 3-7’ displays a negative correlation with PC2. Stage A in both genotypes has a negative correlation while stage C has a positive correlation with PC1. Variables as skin ground color, blush color, flesh color, soluble solids and ethylene release were highly explained by PC1, while fructose, citrate, and malate were mainly explained by PC2. Therefore, PC1 mostly explained variance associated with fruit color and ripening stage represented by ethylene release and soluble solid content, while PC2 explained variance associated with acid taste represented by the content of citrate, malate, and fructose.

### Sequenced Libraries Quality Control

Sequenced raw read libraries analyzed with FastQC (**Supplementary File 1**) showed poor quality sequencing in 3′ and 5′ ends, with several repeated *k*-mers in forward reads as well as a deficient Phred quality score along the entire length of the reverse reads, with a high degree of repeatability and presence of *k*-mers. An acceptable number of duplication sequences, *k-*mer occurrence or GC percentage are organism-specific, and these values must be homogeneous in all samples which belong to the same experiment (Conesa et al., 2016). The high repeatability of the genomes in plants makes tough filter the sequences without losing valuable information, so it was decided to trim the reads with low-quality or presence of very repetitive sequences, suspected of being residual Illumina adapters. After removing these fragments, the quality of the sequenced libraries was analyzed again with FastQC (**Supplementary File 2**).

### Mapping of Whole Transcriptomes

Optimized sequencing libraries were then aligned and assembled with HISAT and TopHat to the reference genomes and transcriptomes of *P. persica* and *P. mume* (**Supplementary Figure 3**, **Supplementary Tables 3** and **4**). The percentage of total pairs of reads mapped to the genomic sequences is higher than to the transcriptomes. When we attend to the differences between species, the assembly and alignment of total pairs of reads to *P. mume* are higher compared to *P. persica*, arising 88.36% mapped pairs of reads to *P. mume* genome performed with HISAT as the best result. Initially, alignment and assembly of total pairs of reads with HISAT lead to a higher percentage of mapped pairs of reads, but also a higher percentage of mapped pairs of reads aligned discordant or in multiple locations. TopHat was more conservative in this sense, avoiding pairs of reads that align discordantly or in multiple sites, so the pairs of reads mapped once with TopHat were higher than those obtained with HISAT.

A comparison between the different MAPQ values on mapping strategies addressed showed 84.62% of mapped reads with a MAPQ ≥ 30, employing *P. mume* genome as reference sequence performed with HISAT as the best result. In opposition, we only get 67.70% of mapped reads using *P. persica* transcriptome as reference sequence with TopHat as the worst result (**Supplementary Figure 4**, **Supplementary Tables 5** and **6**). The best results were the alignment and assembly to the *P. mume* genome using TopHat (82.15%), HISAT to the *P. mume* transcriptome (79.48%), and TopHat to the *P. mume* transcriptome (77.99%). HISAT was selected as the most efficient mapper in terms of percentage of reads aligned with high MAPQ values. Considering the sequence used as the reference sequence, the mapped reads to *P. mume* were higher than *P. persica*. Thus *P. mume* genome is chosen as the reference sequence, which best fits our data.

The assemblies obtained were evaluated for accuracy with SAMStat to determine the quality of the alignments. An average of 51.7 million pairs of reads was generated from the six samples sequenced. The assembly to *P. mume* genome performed with HISAT was filtered by mapping quality score (MAPQ ≥ 30), removing unmapped or multi-mapped reads with SAMtools.

### Differentially Expressed Genes Analysis

Count matrix of mapped reads to *P. mume* genome was performed with featureCounts, getting the number of assigned or unassigned reads to features (annotated genes). Unassigned features were likely to align to non-coding genomic regions or unannotated genes (**Supplementary Table 7**). This was the proposed strategy to group the samples that presented greater similarity. So, Euclidian distance matrix of gene expression was calculated after the normalization of each library (**Supplementary Table 8**), and the results were plotted in a heatmap, complemented with a multidimensional scaling plot (MDS) and a correlation matrix chart (**Supplementary Figure 5**). The higher correlation was obtained among the samples that belong to the same genotype, being ‘GC 2-11’ the genotype, which shows less distance between its samples. Inside each sample, the higher distance was found between stage A and stage C.

DEG analysis was performed grouping the samples as biological replicates by genotype or ripening stage (**Supplementary Table 9A**). In the analysis without replicates (**Supplementary Table 9B**), these were simulated on the assumption that read counts follow a multinomial distribution as NOISeq does. Since version 1.20, DESeq2 did not perform statistical analysis without replicates, DEGs were obtained by logarithmic transformation of the count matrix and selection of genes with fold change values over a range of ±1.5.

**Figure 3:**
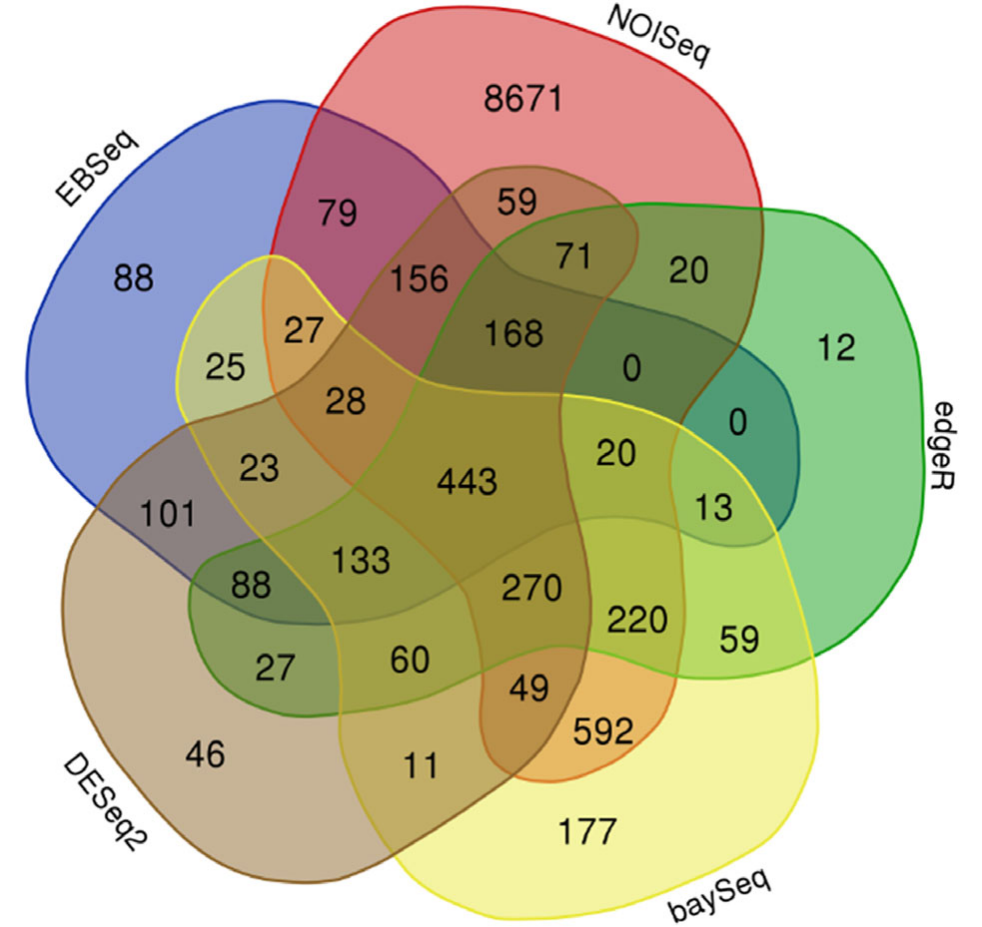
Venn diagram which represents the consensus result obtained from Differentially Expressed Genes Analysis (DEGs) performed on *P. mume*, grouping the samples by genotype and using different statistical packages including NOISeq, edgeR, baySeq, DESeq2, and EBSeq. Three biological replicates and two technical replicates were assayed for each comparison of genotype and stage.

After DEG analysis with five different statistical packages, the results obtained were merged in a consensus result with common genes. Consensus results were plotted in a Venn diagram, where only the comparison between genotypes was of interest because of the number of genes differentially expressed for downstream analysis, arising from 443 DEGs (**Figure 3** and **Supplementary Data Sheet 3**). As consequence of the backward step from this set of 443 DEGs, 310 of the total were identified with an *P. amerniaca* IDs (**Figure 3** and **Supplementary Data Sheet 3**, column C). In addition, for the rest of 133 IDs with an unknown IDs from *P. armeniaca*, the backward mapping allowed the identification of 85 of them (**Figure 3** and **Supplementary Data Sheet 3**, column C). Only a total of 48 genes remained without a specific ID from the reference apricot genome but with a physical position on the genome (**Supplementary Data Sheet 4**).

### Gene Annotation, Enrichment Analysis, and Pathway Visualization

**Figure 4:**
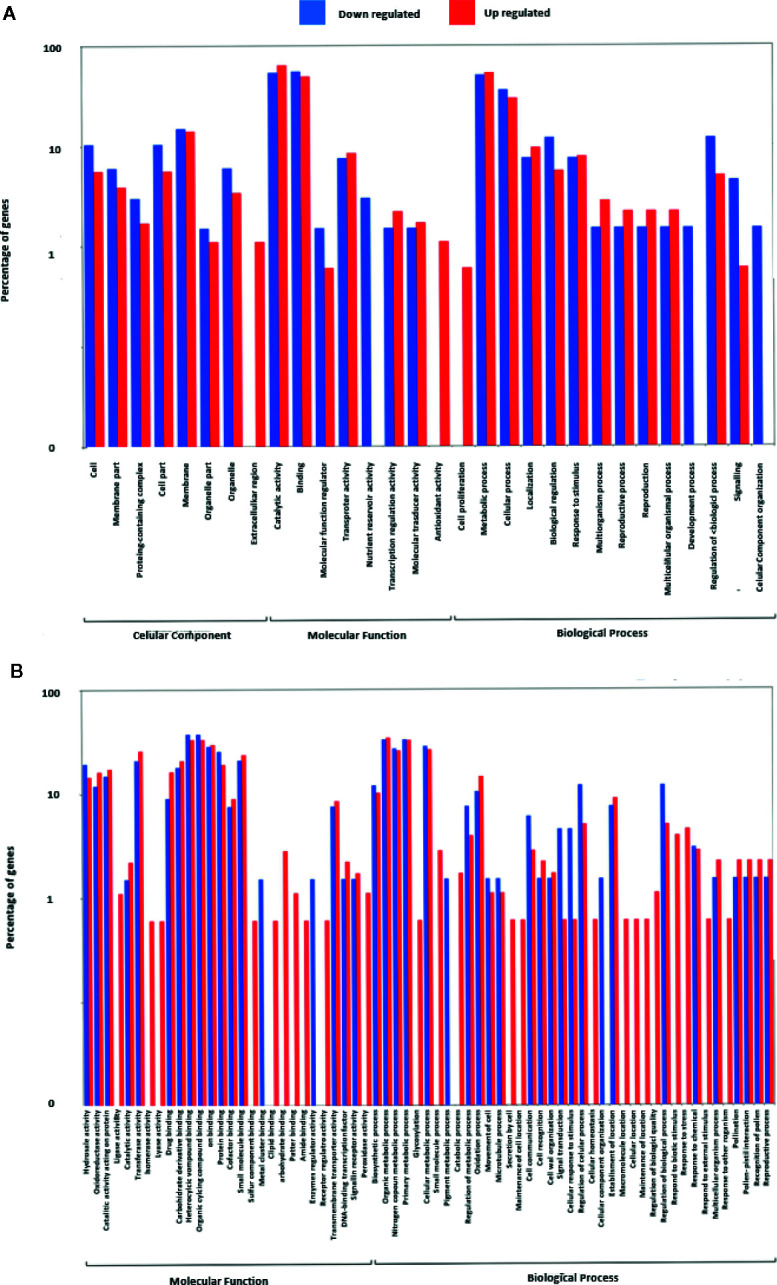
Gene Ontology term enrichment of DEGs found in contrast grouping by genotype. **(A)** Up- and down-regulated DEGs annotated with GO terms. **(B)** Up- and down-regulated DEGs annotated in Molecular Function. Three biological replicates and two technical replicates were assayed for each comparison of genotype and stage.

**Figure 5:**
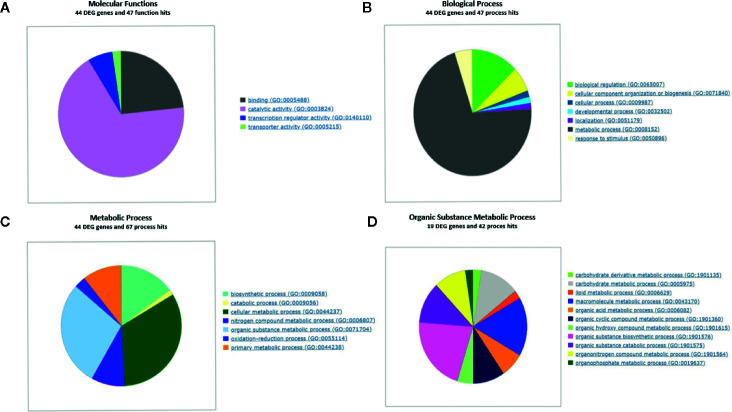
Gene Ontology term enrichment of DEGs found in contrast grouping by genotype for different ontology levels. **(A)** DEGs genes annotated with GO Slim into Molecular Function term. **(B)** DEGs annotated with GO Slim into Biological Process term. **(C)** DEGs annotated with GO Slim into Metabolic Process term. **(D)** DEGs annotated with GO Slim into Organic substance metabolic process. GO enrichment analysis plot with WEGO and GO Consortium webserver. Three biological replicates and two technical replicates were assayed for each comparison of genotype and stage.

To elucidate the fundamental processes altered in ripe apricot fruit, we searched for functional enrichment categories in the set of DEGs obtained identifying homologous genes between mei, peach, and Arabidopsis (**Supplementary Data Sheets 5** and **6**). Enrichment analysis (**Figures 4** and **5**) was performed over up- and down-regulated DEGs obtained in contrast to grouping samples by genotype. The keynote GO terms were annotated in Molecular Function and Biological Process (**Supplementary Table 10**). Plotting up-regulated and down-regulated DEGs in a bar chart for Molecular Function GO annotation, DEGs up-regulated are mainly involved in ligase activity, isomerase activity, lyase activity, sulfur compound binding, carbohydrate binding, lipid binding, amide binding, pattern binding, receptor regulatory activity, and peroxidase activity; while DEGs down-regulated are implied in metal cluster binding and enzyme regulator activity. GO terms annotated in Biological Process are up-regulated for glycosylation, catabolic process, secretion by cell, maintenance of location in cell, cellular homeostasis, macromolecule organization, cellular localization, maintenance of location, regulation of biological quality, response to biotic stimulus, response to stress, response to external stimulus, and response to other organisms. On the other side, down-regulated DEGs were annotated in pigment metabolic process and cellular component organization. DEG genes annotated are implicated in catalytic activity (72.2%), followed by binding (25.0%), transcription regulator activity (6.8%), and transporter activity (2.3%). Most of these genes belong to the metabolic process (74.7%). When we analyzed the organic metabolic process affected, significant annotations were macromolecule metabolic process (36.8%), carbohydrate metabolic process (26.3%), organic acid metabolic process (15.8%), and organic cyclic compound metabolic process. Singular enrichment analysis (SEA) for Biological Process indicates protein phosphorylation and drug transmembrane transport as main GO terms, for Molecular Function highlights catalytic and transport activity (**Supplementary Figure 6**).

**Figure 6:**
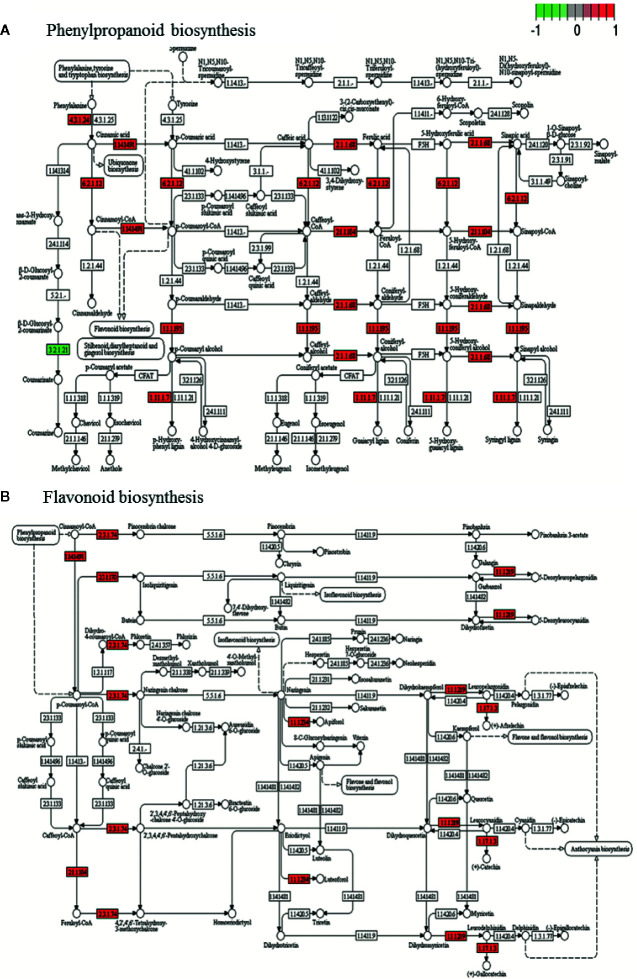
KEGG of phenylpropanoids **(A)** and flavonoid **(B)** biosynthesis pathway with the representation of DEG obtained during genotype contrast in *P. mume* where several genes involved are up-regulated (red) or down-regulated (green) in ‘GC 3-7’ with respect to ‘GC 2-11’ genotype. Gene involved in each pathway is represented in small boxes by its Enzyme Commission numbers (E.C.) code. Three biological replicates and two technical replicates were assayed for each comparison of genotype and stage.

DEGs annotated in KEGG biosynthetic pathways were mainly located in glycolysis/gluconeogenesis (pmum00010), pentose and glucuronate interconversions (pmum00040) starch and sucrose metabolism (pmum00500), terpenoid biosynthesis (pmum00130), carotenoid biosynthesis (pmum00906), phenylalanine metabolism (pmum00360), phenylpropanoid biosynthesis (pmum00940), flavonoid biosynthesis (pmum00941), and cyanoamino acid metabolism (pmum00460) (**Supplementary File 3**). The most affected pathways are phenylpropanoid and flavonoid biosynthesis (**Figure 6**), followed by starch and sucrose, and phenylalanine metabolism (**Supplementary Figure 7**).

### Weighted Gene Co-Expression Network Analysis

**Figure 7:**
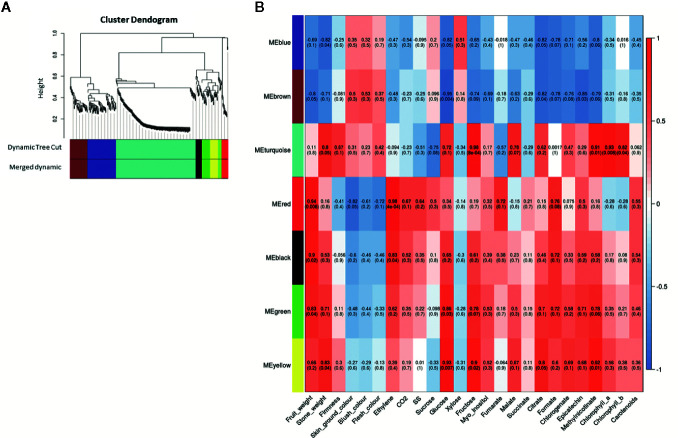
Weighted correlation network analysis during apricot fruit ripening obtained with WGCNA R package. **(A)** Gene dendrogram and module colors obtained by average linkage hierarchical clustering where every leaf represents a DEG. The color row underneath the dendrogram shows the seven eigengene modules (ME) assignments determined by the Dynamic Tree Cut (threshold set for cut the dendrogram and obtain ME clustering). **(B)** Heatmap representation of the eigengene module-quality trait relationship for *P. mume* genotype contrast obtained when analyzing DEG expression and quantitative quality traits. Inside each module the Pearson correlation value is indicated and between parenthesis the *p*-value significance of its correlation. Three biological replicates and two technical replicates were assayed for each comparison of genotype and stage.

We got seven ME after WGCNA including blue, brown, turquoise, red, black, green and yellow (**Figure 7**). A total of 443 genes were clustered in these seven ME. When we analyze each ME, 80 genes belong to ME blue, 49 genes to ME brown, 226 genes to ME turquoise, 17 genes to ME red, 17 genes to ME black, 25 genes to ME green, and 29 genes to ME yellow (**Supplementary Data Sheet 7**). Additionally, we analyzed the principal KEGG pathways involved in fruit ripening for each ME (**Supplementary Data Sheet 8**) and other genes of interest not annotated on KEGG pathways but described in other databases. For significant correlation in ME with quality characteristics, we set a threshold over 0.5 for correlation coefficient and under 0.5 for *p-*value statistical significance.

ME blue was positively correlated with xylose and negatively correlated with fruit weight, stone weight, CO_2_ release, glucose, fructose, citrate, formate, chlorogenate, epicatechin, and methyl nicotinate. Cluster in this ME we find the genes of starch and sucrose metabolism *LOC103322685* (*PARG04316m02*; EC:2.4.1.14), *LOC103333663* (*PARG19611m01*; EC:3.1.3.12), *LOC103338042* (unknown PARG ID; EC:3.1.3.12) and *LOC103343477* (*PARG01655m01*; EC:3.2.1.21), phenylpropanoid biosynthesis pathway *LOC103332355* (unknown PARG ID; EC:4.3.1.24), *LOC103335298* (*PARG29723m01*; EC:1.1.1.324), *LOC103338042* (unknown PARG ID; EC:3.2.1.21), *LOC103339178* (unknown PARG ID; EC:1.14.14.91) and *LOC103343477* (*PARG01655m01*; EC:3.2.1.21), phenylalanine metabolism *LOC103332355* (unknown PARG ID; EC:4.3.1.24) and *LOC103339178* (unknown PARG ID; EC:1.14.14.91), *LOC103338042* (EC:3.2.1.21; unknown PARG ID) and *LOC103343477* (EC:3.2.1.21; *PARG01655m01*) and flavonoid biosynthesis *LOC103339178* (EC:1.14.14.91; unknown PARG ID). Other genes of interest are *LOC103329206* (*PARG00063m07*) predicted as a pheophytinase, and *LOC103328016 (PARG00063m07)* as ABC transporter pleiotropic drug resistance (PDR).

ME brown showed a positive correlation with blush color while a negative correlation with fruit weight, stone weight, glucose, fructose, *myo*-inositol, malate, citrate, formate, chlorogenate, epicatechin, and methyl nicotinate. Cluster in this ME we find genes of starch and sucrose metabolism *LOC103326244* (*PARG10672m01*; EC:3.2.1.21) and *LOC103327693* (*PARG15761m01*; EC:3.2.1.26), phenylalanine metabolism *LOC103328252* (EC:6.2.1.12; *PARG15135m02*) and *LOC103340770* (EC:2.1.1.104; *PARG27724m01*), phenylpropanoid biosynthesis *LOC103326244* (*PARG10672m01*; EC:3.2.1.21), *LOC103328252* (*PARG15135m02*; EC:6.2.1.12) and *LOC103340770* (EC:2.1.1.104; *PARG27724m01*), and flavonoid biosynthesis *LOC103340770* (EC:2.1.1.104; *PARG27724m01*). Another gene of interest is *LOC103326648* (*PARG22281m01*), predicted as a transcription factor (TF) with MYB/SANT domain, and *LOC103331940 (PARG18142m01*) predicted as MADS-box protein SOC1.

ME turquoise showed a positive correlation with stone weight, firmness, fructose, glucose, malate, citrate, chlorophylls content, and methyl nicotinate, while a negative correlation with sucrose, soluble solid content, and fumarate. We found several genes related phenylalanine metabolism *LOC103342143* (*PARG02182m01*; EC:4.3.1.24), carotenoid biosynthesis *LOC103328471* (*PARG01425m02*; EC:5.3.99.8), phenylpropanoid biosynthesis *LOC103327307* (*PARG00008m01*; EC:1.11.1.7), *LOC103342143* (*PARG02182m01*; EC:4.3.1.24) and *LOC103343871* (*PARG24274m01*; EC:2.1.1.68), flavonoid biosynthesis *LOC103324315* (*PARG03336m04*; EC:2.3.1.74) and *LOC103341429* (*PARG06615m01*; EC:1.17.1.3), glycolysis and gluconeogenesis *LOC103332280* (*PARG18437m02*; EC:5.1.3.15), and circadian rhythm *LOC103324315* (*PARG03336m04*; EC:2.3.1.74). Other genes of interest were *LOC103323450* (unknown PARG ID) predicted as anthocyanidin 3-O-glucosyltransferase 7-like, *LOC103341429* (*PARG06615m01*) as a leucoanthocyanidin reductase-like, *LOC103322538* (*PARG05244m01*) as probable pectinesterase/pectinesterase inhibitor 35, and *LOC103342143* (*PARG02182m01*) as a phenylalanine ammonia-lyase 1. In addition, other identified genes included the TFs *LOC103327289* (*PARG28632m01*) predicted as MYB108, *LOC103330396* (*PARG12740m01*), *LOC103337200* (*PARG23210m01*) as a probable WRKY TF 45, *LOC107880999* (*PARG12882m01*), *LOC103332564* (*PARG18828m01), LOC103323278* (*PARG04365m02*)*, LOC103323316* (*PARG04392m03*), and *LOC103323318* (*PARG04392m01*) predicted as ABC transporter-like.

ME red displayed a significant positive correlation with fruit weight, ethylene and CO_2_ release, soluble solid content, fumarate, formate, epicatechin, and carotenoid content, while a negative correlation with skin ground color, flesh color and blush color. We found the gene from starch and sucrose metabolism and phenylpropanoid biosynthesis *LOC103330627* (*PARG01659m01*; EC:3.2.1.21), and phenylalanine biosynthesis *LOC103329641* (*PARG13381m01;* EC:4.2.1.20). A TF is identified, *LOC103342585* (*PARG26597m01)* as L10-interacting MYB domain-containing protein-like. ME black displayed a positive correlation with stone weight, fruit weight, ethylene and CO_2_ release, glucose, fructose, formate, epicatechin, methyl nicotinate, and carotenoids content. The only gene of interest found in this cluster is *LOC103333822* (*PARG04832m02*) as sugar transporter ERD6-like 7.

ME green, however, had a positive correlation with stone weight, fruit weight, ethylene release, glucose, fructose, *myo*-inositol, citrate, formate, chlorogenate, epicatechin, and methyl nicotinate. We find the gene of flavonoid biosynthesis *LOC103320869* (PARG07267m01; EC:1.1.1.219 and 1.1.1.234). Other genes of interest are *LOC103334507* (*PARG20234m01*) predicted as 1-aminocyclopropane-1-carboxylate oxidase homolog 11-like, and *LOC103323820* (*PARG03840m02*) predicted as glutathione transferase GST 23-like.

Finally, ME yellow displayed a positive correlation with stone weight, fruit weight, glucose, fructose, *myo*-inositol, malate, citrate, formate, chlorogenate, epicatechin, methyl nicotinate, and chlorophyll *a* content. In yellow ME, we found a gene involved in phenylpropanoid biosynthesis *LOC103335288* (*PARG29722m01*; EC:1.1.1.324). Other genes of interest are *LOC103324123* (*PARG03642m01*) described as 1-aminocyclopropane-1-carboxylate oxidase homolog 1-like, *LOC103330065* (*PARG13187m01*) as 2-hydroxyflavanone C-glucosyltransferase, *LOC103333172* (*PARG19510m01*) as multidrug resistance protein from MATE family, and *LOC103324364 (PARG00058m01)* predicted as pheophorbide A oxygenase.

### Gene Expression Analysis by Quantitative Real-Time PCR

**Figure 8:**
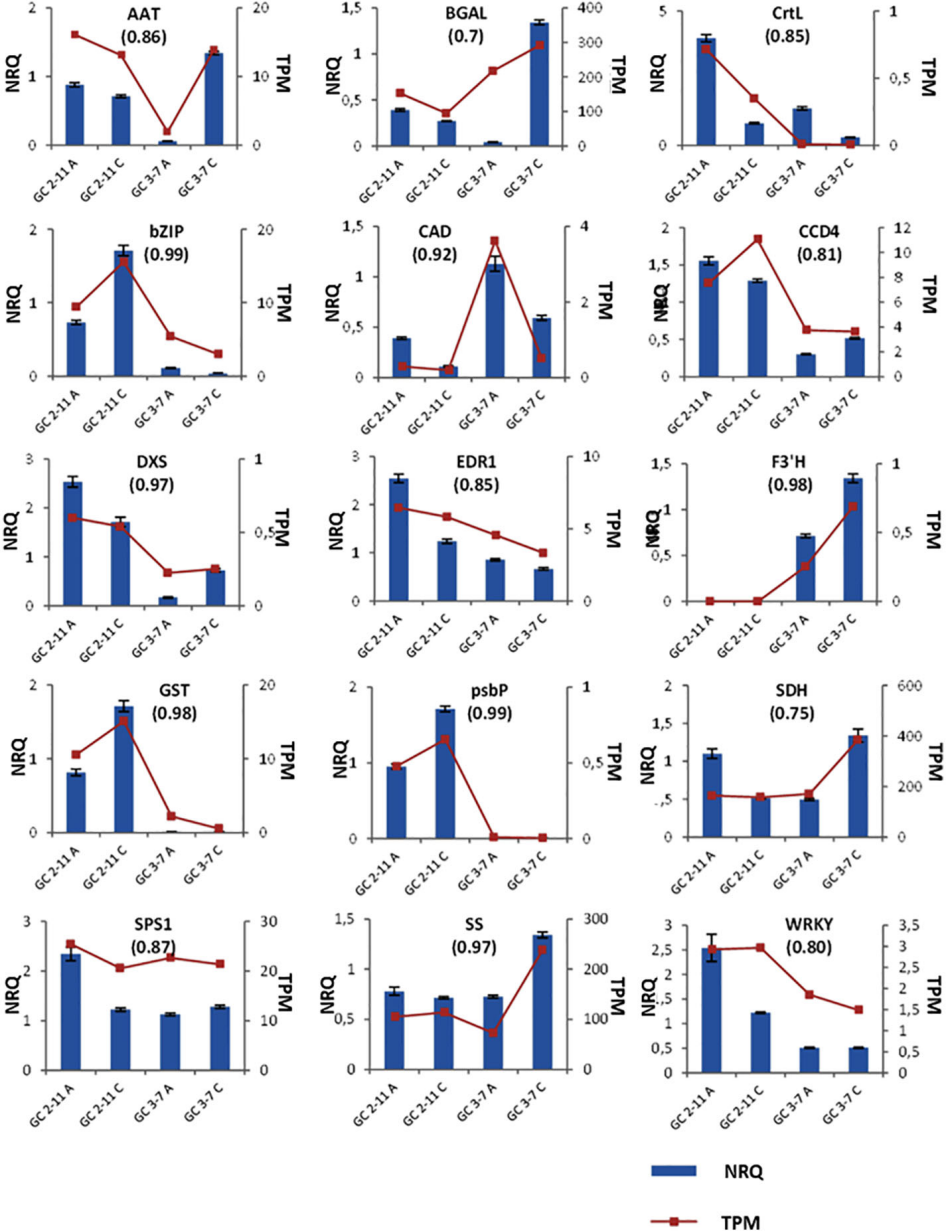
Plots for the NRQ (Normalized Relative Quantification) of 15 genes analyzed by RT-qPCR and each TPM (Transcript Per Million) value obtained in RNA-Seq experiment. In brackets, Pearson correlation coefficient obtained from contrast between TPM and NRQ. From upper left to down right: *Methanol O-anthraniloyl transferase-like* (*AAT*), *β-galactosidase* (*BGAL*), *lycopene β-cyclase* (*CrtL*), *common plant regulatory factor 1-like* (*bZIP*), *cinnamyl-alcohol dehydrogenase* (*CAD*), *carotenoids cleavage dioxygenase 4* (*CCD4*), *1-deoxy-D-xylulose-5-phosphate synthase* (*DXS*), *ethylene transduction gene* (*EDR1*), *flavonoid 3′-hydroxylase 1-like* (*F3′H*), *glutathione transferase GST 23-like* (*GST*), *psbP domain-containing protein 6* (*psbP*), *sorbitol dehydrogenase* (*SDH*), *sucrose-phosphate synthase 1* (*SPS1*), *sucrose synthase* (*SS*) and *probable serine/threonine-protein kinase PBL16* (*WRKY*). Three biological replicates and two technical replicates were assayed. Standard deviations are indicated with vertical bars.

RNA-Seq was validated by analyzing fifteen genes highly expressed through RT-qPCR. According to the analysis run in RefFinder, we obtain a comprehensive gene stability values of 1.141 for *S18*, 1.565 for *CYP1*, 2.28 for *RPL12*, 4.229 for *ACT*, and 4.729 for *UBI*. The lower value of comprehensive gene stability, most stable is the expression of a gene between samples. Then, we use as reference genes *S18*, *RPL12* and *CYP1*, the most stable genes analyzed. These genes were described with *P. persica* and *P. mume* annotation, and most of them are implied in the ripening process (**Supplementary Data Sheet 1**). The samples taken in the course of advanced color change (Stage B) were discarded because of the difficulty to establish effective criteria at sampling and the high variability found in the results obtained. All the genes analyzed have a Pearson correlation coefficient over 0.7 in TPM-NRQ comparison (**Figure 8**), are genome-wide distributed, and most of them are related to quality traits at fruit ripening process. Performing RT-qPCR on a new set of samples gives us the greatest confidence in the results obtained by validating the HTS technology and the underlying biological response.

### Candidate Genes Related to Pomological Characteristics and Biochemical and Metabolite Contents in Apricot Fruits

Taking into account the analysis previously accomplished and integrating the results obtained, we proposed a variety of candidate genes for monitoring fruit ripening process linked to quality traits in ripe fruit such as fruit color and soluble solid accumulation.

**Figure 9:**
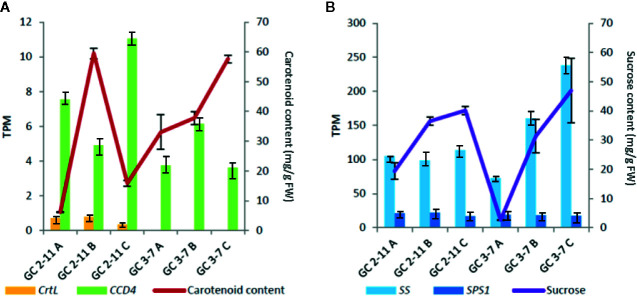
Cross-linked graph of relation between metabolite contents and gene expression obtained in RNA-Seq. **(A)** Relation of carotenoid content with the expression of *capsanthin/capsorubin synthase* or *lycopene β-cyclase* [*CrtL, LOC103328471* (*PARG02182m01*)] and *carotenoid cleavage dioxygenase 4* (*CCD4*). **(B)** Relation of sucrose content with the expression of *sucrose synthase* [*SS, LOC103340632* (*PARG27579m02*)] and *sucrose-phosphate synthase 1* [(*SPS1, LOC103341128* (unknown PARG ID)]. Standard deviations are indicated with vertical bars.

The most important correlation with significant statistical *p-*value obtained in WGCNA for starch and sucrose metabolism found in ME blue, brown, turquoise and yellow (**Figures 6** and **7**, **Supplementary Data Sheets 5** and **7**). In addition, we found a positive correlation with sucrose, while fructose and sucrose have a negative correlation and *vice-versa* for all the ME cited above. In ME blue, with high negative correlation with glucose, we find a *sucrose-phosphate synthase* (*LOC103322685; PARG04316m02)* which leads to the transformation of D-fructose-6P and UDP-glucose into sucrose-6’P, and two *β-glucosidases* (*LOC103338042* (unknown PARG ID) and *LOC103343477* (*PARG01655m01*). In ME brown, with high negative correlation with glucose, cluster a *β-glucosidase* [*LOC103326244* (*PARG10672m01*)] implied in starch and sucrose metabolism leading to D-glucose, and a *β-fructofuranosidase* [*LOC103327693* (*PARG15761m01*)] which catalyze the dephosphorylation from sucrose into D-glucose and D-fructose. ME turquoise displayed the highest positive correlation with fructose content and lower significant *p-*value due to the presence in this cluster of the gene *glucose-6-phosphate 1-epimerase*, which transforms D-glucose-6P between its two stereoisomers α and β and may lead to β-D-fructose-6P through the *glucose-6-phosphate isomerase*. Sucrose is transported and accumulated into the vacuole by membrane transporter as early-response to dehydration gene (*ERD6*) (Zhang et al., 2019), cluster in ME black. Besides, *SPS1* is up-regulated at the beginning of ripening in ‘GC 2-11’, while *SS* is up-regulated in ‘GC 3-7’ at the end of the ripening process (**Figure 8**). When we compared the sucrose content during fruit quality evaluation (**Supplementary Table 2**) and the gene expression of two suspicious genes responsible for sucrose biosynthesis *sucrose synthase* [*SS*, *LOC103340632* (*PARG27579m02*)] and *sucrose-phosphate synthase 1* [*SPS1, LOC103341128* (unknown PARG ID)], Pearson correlation coefficient shows higher positive correlation between sucrose content and *SS* gene expression (Pearson coef.: 0.7) than *SPS1* (Pearson coef.: −0.2) (**Figure 9**), *SS* being a candidate gene for the biosynthesis of sucrose during fruit ripening in apricot.

Several genes related to phenylalanine, phenylpropanoids, and flavonoid biosynthesis, the precursors of anthocyanin compounds, were identified in ME blue, brown, green, yellow, and turquoise (**Figures 6** and **7** and **Supplementary Data Sheets 5** and **7**). In the phenylpropanoid biosynthesis pathway we find two *phenylalanine ammonia lyases* (*PAL, LOC103332355* (unknown PARG ID) and *LOC103342143* (PARG02182m01) which catalyzes the transformation from phenylalanine to cinnamic acid, a *trans-cinnamate 4-monooxygenase* [*C4H, LOC103339178* (unknown PARG ID)] transforming cinnamic acid to *p*-coumaric acid, a *4-coumarate-CoA ligase 1-like* [*4CL*, *LOC103328252* (*PARG15135m02*)] catalyzing cinnamic acid or *p*-coumaric acid to cinnamoyl-CoA or *p*-coumaryl-CoA, and in the end of the pathway identified a probable *caffeoyl-CoA O-methyltransferase* [*LOC103340770, PARG27724m01*)] transforming caffeoyl-CoA to feruloyl-CoA. Following with flavonoid biosynthesis pathway, we find the gene for *chalcone/stilbene synthase* [*CHS, LOC103324315* (*PARG03336m04*)] catalyzing the reaction from *p*-coumaroyl-CoA to naringenin chalcone. The last gene implicated on anthocyanin synthesis identified is a bifunctional *dihydroflavonol 4-reductase/flavanone 4-reductase* [*DFR*, *LOC103320869* (*PARG07267m01*)], which synthesizes leucocyanidin. Other genes of interest not annotated on KEGG pathways are a *leucoanthocyanidin reductase-like* [*ANS*, *LOC103341429* (*PARG06615m01*)], leading leucoanthocyanidin, then *anthocyanidin 3-O-glucosyltransferase* [*UFGT*, *LOC103323450* (unknown PARG ID)] convert cyanidin into cyanidin 3-glucoside. *Flavonoid 3’-hydroxylase 1-like* [*F3′H, LOC103337305* (*PARG23256m01*)], a cytochrome P450 responsible for anthocyanin biosynthesis, is up-regulated in ‘GC 3-7’ (**Figure 8**). Besides, a *glutathione transferase GST 23-like* [*GST*, *LOC103323820* (*PARG03840m02*)], related to the antioxidant system ascorbate-glutathione and degradation of hydrogen peroxide during ripening (Fuentealba et al., 2017) and responsible for organic acid and secondary metabolism compounds translocation into the vacuole (Shiratake and Martinoia, 2007) was differentially expressed in contrast by genotypes in *P. mume* and up-regulated in ‘GC 2-11’.

After the analysis of the blush color, this trait showed highest negative (reddish color for lower Hue° values) correlation with ME red (**Figure 7**, **Supplementary Data Sheets 5** and **7**), where we found the gene *LOC103342585* (*PARG26597m01*) as L10-interacting MYB domain-containing protein-like.

ABC and MATE transporters find in ME turquoise, blue, and yellow (**Figure 7** and **Supplementary Data Sheet 5**), were responsible for the transit of many substances like organic acids or secondary metabolism. ATP binding cassette ABC transporters were described as responsible for citrate and malate accumulation. ABC transporters of Multidrug Resistance-associated Protein (MRP) together with glutathione transferases, found one gene in ME green, are described as anthocyanin transporters by the deposition of large amounts of phenolic compounds in vacuoles, being implicated in fruit pigmentation. On the other hand, ABC transporters of Pleiotropic Drug Resistance (PDR) subfamily are responsible for terpenoid transport. Most of these ABC transporters were found up-regulated in ‘GC 3-7’ (**Supplementary Data Sheet 3**), which display higher levels of citrate, orange flesh and skin ground color, and reddish blush (**Supplementary Tables 1** and **2**). Multidrug and toxin efflux transporters (MATE) family are suggested as responsible for flavonoid and other phenolic compounds transport (Shiratake and Martinoia, 2007), up-regulated in ‘GC 3-7’ (**Supplementary Data Sheet 3**).

For carotenoids biosynthesis pathway we only identified a *capsanthin/capsorubin synthase* or *lycopene β-cyclase* [*CrtL*, *LOC103328471* (*PARG02182m01*)] in ME turquoise (**Figure 7**, **Supplementary File 3**, **Supplementary Data Sheets 5** and **7**), related to the secondary metabolism of carotenoids by lycopene cyclization, yielding *β*-carotene, which does not correlate with carotenoid content. This gene is up-regulated at the beginning of the ripening process in ‘GC 2-11’, the light yellow flesh and skin ground color genotype, which is the opposite of what we expect when we take into account that this gene leads the synthesis of *β*-carotene, responsible for the orange color in fruits and the precursor of vitamin A. Going further, we analyze the expression of *carotenoid cleavage dioxygenase 4* (*CCD4*), which has been reported as a carotenoid degradative enzyme responsible for white flesh phenotype in peach. White flesh in peach was described as a monogenic trait controlling *Y* locus ligated to *CCD4* gene expression (Adami et al., 2013); it is up-regulated in ‘GC 2-11’ (**Figure 8**). It was described in yellow/white peach that carotenoid accumulation in skin ground color and flesh color depends on the expression profile of *lycopene β-cyclase* (*PpLCYB*) and *carotenoid cleavage dioxygenase 4* (*PpCCD4*) (Cao et al., 2017). When we calculated the Pearson correlation coefficient between carotenoid content obtained at fruit quality evaluation (**Supplementary Table 1**) and the gene expression of *CCD4* and *CrtL*, we obtain a negative correlation for *CCD4* (Pearson coef.: −0.71) while a lower correlation with *CrtL* (Pearson coef.: −0.19) taking in account the low expression level for the *CrtL* gene which shows differential expression levels between white fleshed apricot fruits of about 0.5 TPM and yellow flesh of 0 TPM (**Figure 9A**). The coordinate expression of both genes during ripening process caused an increase in the synthesis of carotenoids in both genotypes during color change, followed by a decrease in carotenoid content only in the light yellow genotype ‘GC 2-11’, where *CCD4* is up-regulated, the expression of *CCD4* being the major determinant for carotenoid content by degrading *β*-carotene into apocarotenoid compounds.

As a carotenoid precursor biosynthesis enzyme, we identified *1-deoxy-D-xylulose-5-phosphate synthase* (*DXS*), which catalyzes the fusion of pyruvate and glyceraldehyde-3-phosphate in the 2-C-methyl-D-erythritol 4-phosphate pathway (MEP pathway), yielding 1-deoxy-D-xylulose 5-phosphate (DXP), precursor compound in carotenoid biosynthesis (Brandi et al., 2011). It is found up-regulated in ‘GC 2-11’.

Skin ground color and flesh color were negatively correlated with ME green and yellow (**Figure 7**, **Supplementary Data Sheets 5** and **7**), which are also positively correlated with ethylene burst, which is described as the regulator of some genes involved in carotenoid biosynthesis pathways (Marty et al., 2005) (**Supplementary File 3**). Some apricot varieties, as climacteric fruits, release ethylene in the final ripening stages. It has been described that the loss of firmness as a result of the degradation of galactosyl-containing polymers by *β*-galactosidase, up-regulated in ‘GC 3-7’ (**Figure 8**), stimulates ethylene release (Kovacs and Nemeth-Szerdahelyi, 2002). We find the genes responsible for ethylene biosynthesis *LOC103334507* (*PARG20234m01*) and *LOC103324123* (*PARG03642m01*), both predicted as *1-aminocyclopropane-1-carboxylate oxidase homolog like* (*ACO*). However, no correlation was found between ethylene release and *ACO* expression even though it was described in apricot that *ACO* was strongly up-regulated during ripening before ethylene production (Mbéguié-A-Mbéguié et al., 1999). The other candidate gene involved in ethylene perception assayed was *Ethylene transduction gene* (*EDR1*), responsible for ethylene response transduction gene at fruit ripening in peach (Wang et al., 2017), and it is up-regulated in ‘GC 2-11’, which did not produce ethylene. No candidate genes were proposed for ethylene signaling.

Related with loss firmness at ripening process, we found a *pectin methylesterase inhibitor* gene [*PMEI, LOC103322538* (*PARG05244m01*)], cluster in ME turquoise (**Figure 7**, **Supplementary Data Sheets 5** and **7**) and up-regulated in ‘GC 2-11’ (**Supplementary Data Sheet 3**), with a slow loss of firmness if we compared to ‘GC 3-7’ (**Supplementary Table 1**). The modification of pectins during ripening resulted in tissue softening and an overall loss of firmness. The inhibition of this enzymatic activity may avoid or retard softening in ripe fruit (Femenia et al., 1998). Another gene assayed was *β-galactosidase* [*BGAL*, *LOC103340681* (*PARG27772m02*)] which induced loss of firmness during ripening by the degradation of galactosyl-containing polymers (Kovacs and Nemeth-Szerdahelyi, 2002) and found up-regulated in ‘GC 3-7’ at the end of the ripening process. Besides, we analyze the expression of *cinnamyl-alcohol dehydrogenase* [*CAD, LOC103335323* (*PARG29726m01*)], involved in lignin biosynthesis, which may be related to firmness during fruit development (Gabotti et al., 2015; Zhang et al., 2017b). It is up-regulated in ‘GC 3-7’ at the beginning of the ripening process.

For chlorophyll degradation and color green loss from green to ripe fruit (**Supplementary Table 2**), we found differential expression in genes related with chlorophyll dephytylation in ME blue *LOC103329206* (*PARG00063m07*) as a pheophytinase, and ME yellow *LOC103324364* (*PARG00058m01*) as a pheophorbide A oxygenase (Guyer et al., 2014) (**Figure 7** and **Supplementary Data Sheet 5**). Another gene related with photosynthesis was *psbP domain-containing protein 6* [*psbP*, *LOC103326460* (*PARG10392m01*)]; as part of the photosystem II, *PsbP* increases the affinity of the water oxidation site for chloride ions and provides the conditions required for high-affinity binding of calcium ions (Kochhar et al., 1996). It is differentially expressed in contrast by genotypes in *P. mume.*

Finally, we get some TF as a MADS-box TF found in ME brown **(** **Figure 7** and **Supplementary Data Sheet 5**), with a major expression in fruit tissues related to fruit development (Xu et al., 2014; Wells et al., 2015). A *common plant regulatory factor 1-like* [*bZIP, LOC103332281* (*PARG18436m04*)], which was a homologous gene of *CPRF1_PETCR* in *Petroselinum crispum* and bind to *chalcone/stilbene synthase* (*CHS*) gene promoter, a key enzyme during anthocyanin biosynthesis, was up-regulated in ‘GC 2-11’ at the end of the ripening process. Furthermore, a *probable serine/threonine-protein kinase PBL16* (*WRKY*), a plant specific TF that controls environment response to a biotic stimulus, was up-regulated at the beginning of ripening in ‘GC 2-11’.

## Discussion

### Pomological Characteristics and Biochemical and Metabolite Contents in Apricot Fruits

The biosynthesis of carotenoids was responsible for the orange color in the skin ground color and flesh color in both genotypes. In adition, anthocyanins were responsible for the reddish blush color, arise the acquisition of definitive color of the ripe fruit. The content of phenylpropanoids (chlorogenate, epicatechin, and methyl nicotinate), precursors of anthocyanin biosynthesis, showed a slight decrease during the ripening process in ‘GC 3-7’ while no significant changes in ‘GC 2-11’. The principal differences found between genotypes and stages were related to skin ground color, flesh color, and blush color. In genotype ‘GC 2-11’ the carotenoid content increased during the ripening process reaching the maximum amount during color change, followed by an abrupt decrease at physiological ripening. On the other hand, carotenoid content in genotype ‘GC 3-7’ undergoes a gradual but constant increase throughout the ripening process, reaching higher levels than ‘GC 2-11’ at the end of the ripening process. If we pay attention to the skin ground color and flesh color changes, it is correlated with the carotenoid content, while the biosynthesis of phenylpropanoid may be correlated with blush color intensity. Chlorophyll content decreased in both genotypes until it is completely degraded, finishing the ripening process with the total loss of green hue and the acquisition of final light yellow/orange fruit color.

Metabolite evolution of apricot fruits during the ripening process agrees with recent results in peach (Lillo-Carmona et al., 2020). Total soluble solids measured as a percentage is an approximation to the strength of the solution as the percentage of sucrose in fruit juice, but it was affected by the presence of other solutes, while results of carbohydrate content obtained at ^1^H-NMR is an accurate and more reliable result. Sucrose and glucose were the major soluble sugars in apricot ripe fruit, reaching the peak of maximum concentration at ripe fruit, higher in genotype ‘GC 3-7’ than in ‘GC 2-11’. At the beginning of the ripening process, glucose content was higher than sucrose, but sucrose content enhanced significantly at the end of ripening, surpassing the amount of glucose in both genotypes. These results represented the pattern of sugar accumulation during the development and ripening of the fruit undergoes from being glucose-predominant to sucrose-predominant at the end of the fruit ripening process.

During the development of apricot fruit, there was a continuous accumulation of organic acids, and their final concentration was determined by the balance between the biosynthesis of organic acids, its degradation, and the vacuolar storage. Malate and citrate were the most abundant organic acids in apricot fruit, and titratable acidity decreases as a result of its degradation during ripening. At green fruit, malate is the predominant compound, whereas the ratio was changing with the rapid increase of citrate at ripening stage when the malate declined at physiological ripening. However, citrate also decreased at the ripening stage; the ratio between citrate and other organic acids increases, becoming the major organic acid at ripe fruit. The significant decrease in citric acid and the small reduction of malic acid contributed to acidity loss. When we compared the amount in organic acids in both genotypes, ‘GC 3-7’ had higher levels of organic acids than ‘GC 2-11’, which resumed in higher titratable acidity.

Finally, there was a pronounced release of ethylene and firmness decline. In climacteric fruit, ethylene is the principal ripening trigger, partially responsible for fruit softening rate and carotenoid biosynthesis (Hayama et al., 2006; Kita et al., 2007). In this sense, apricot fruit was described as differentially sensitive to ethylene (Mbeguie-A-Mbeguie et al., 2002). ‘GC 3-7’ release much more ethylene than ‘GC 2-11’, which may be related with the faster softening occurred during ripening and the orange fruit color in ‘GC 3-7’ in contrast with slowly softening and light yellow fruit color in ‘GC 2-11’.

### Mapping of Whole Transcriptomes

The alignment and assembly of RNA-Seq reads in the non-model organism *P. armeniaca* to the genomes and transcriptomes of *P. persica* and P. *mume* show very different results depending on the protocol applied. A significantly higher percentage of aligned reads with MAPQ ≥ 30 were obtained when we map HISAT to the genome sequence of *P. mume* in comparison with *P. persica*. Although up to now *P. persica* has been used as a model species in the study of the genus *Prunus* sp., this taxon belongs to the subgenus *Amygdalus*, different from the subgenus *Prunus* to which apricot belongs (Potter, 2012). In our case the use of the genome of *P. mume* as a reference sequence owing its closer phylogenetic proximity, because these species belong to the same subgenus *Prunus* and *Armeniaca* section (Potter, 2012), seems an ideal strategy as has been confirmed by the posterior analysis using the reference genome of this species for our backward step. These mapping results were also contrasted with the reference genome of *P. armeniaca*, allowing us the identification of the physical position (coordinates) of candidates genes.

### Differentially Expressed Genes and Enrichment in Relation to Pomological Characteristics and Biochemical and Metabolite Contents

There was no agreement on which protocol for DEG identification is the most appropriate, so the consensus among five DEG identification methods guarantees a list of DEGs with more accuracy, sensitivity, and robustness in gene expression estimation reliable results (Costa-Silva et al., 2017). DEGs without replicates will not be considered because they are only useful for exploring the data but will not provide the kind of proper statistical inference on differences between samples or estimate the biological variability of each gene (Love et al., 2014; Conesa et al., 2016; Love et al., 2016).

The DEG identification method was the crucial decision for the differential expression analysis in RNA-Seq data and will be the key to understand the phenotype–genotype variation through the biological interpretation of the data. The confidence in the quantitative analysis depends on this point more than on depth read or read length.

It is essential to include at least three replicates to capture the biological variability between samples. Accordingly, after the exploration of concordance between the samples, we decided to group the samples by genotypes. It means that as DEG analysis result, we obtained those genes differentially expressed in apricot fruit during ripening process among the two genotypes ‘GC 2-11’ and ‘GC 3-7’.

The annotation of DEGs using GO terms as ontologies to represent biological knowledge offered a detailed source for functional transcriptomic studies based on a dynamic, structured, and controlled vocabulary. GO terms provide a classification that covers several domains by developing a comprehensive and computational model of biological systems, ranging from molecular to organism level (Consortium, 2004). Gene Set Enrichment Analysis of DEGs and pathway identification defines even more precisely the exact physiological process affected, describing a comprehensive knowledge representation.

During the development and ripening the main biological processes affected were catalytic activity, binding, and transporter activity, which means degradation, modification, or translocation of molecules; most of these processes are related to secondary metabolism. Transcription regulator activity also appears represented as the importance of gene expression regulation during the ripening process. Main GO terms up-regulated annotated in Biological Process are glycosylation and catabolic process, while down-regulated is pigment metabolic process. This is strongly correlated with carbohydrate metabolism and pigment content biosynthesis in each of the genotypes under study.

KEGG enrichment analysis of DEGs identifies affected pathways, phenylpropanoid biosynthesis being the most altered pathway. Phenylpropanoid pathway synthesizes the precursor compounds of anthocyanins, which are linked to reddish blush color (Ye et al., 2019) in the genotype ‘GC 3-7’. Other precursors of anthocyanin compounds are flavonoids, phenylpropanoid biosynthesis, and phenylalanine metabolism, also up-regulated in ‘GC 3-7’. Finally, we can describe the pathways related with soluble solid content, starch and sucrose metabolism and glycolysis/glucogenesis, responsible for the biosynthesis of sucrose from glucose and fructose, increasing soluble solid content as responsible to the sweet taste in the ripe fruit apricot. Some genes of starch and sucrose metabolism pathway were up-regulated in ‘GC 3-7’, which could be correlated with the higher sucrose and soluble solid content in this genotype.

### Candidate Genes Related to Pomological Characteristics and Biochemical and Metabolite Contents in Apricot Fruits

A large-scale transcriptomic studies of apricot and related species ripening process showed a significant up- and down-regulated transcript levels of genes related to stress conditions, cell wall metabolism, transcription factors (MADS-box, AUX/IAA, bZIP, bHLH, and MYB), heat shock proteins (HSPs), ethylene biosynthesis, starch and sucrose metabolism, organic acids metabolism, phenylpropanoid and carotenoid biosynthesis (Trainotti et al., 2003; Grimplet et al., 2005; Trainotti et al., 2006; Trainotti et al., 2007; Manganaris et al., 2009; Manganaris et al., 2010; Manganaris et al., 2011).

The principal enzymes involved in starch and sucrose metabolism are sucrose synthase (SS), sucrose phosphate synthase (SPS), and sorbitol dehydrogenase (SDH). These are under tight regulatory control, increasing its expression at the end of the ripening process, which is consistent with the correlation analysis between enzyme activity and sugar accumulation (Xi et al., 2016; Zhang et al., 2019). The drastic increase of SS and sucrose during the ripening process is responsible for the rapid increase of soluble solid content in fruits (Meixia et al., 2006). In addition, a high positive correlation between sucrose content and *SS* expression during fruit ripening has been shown for the genotype ‘GC3-7’. However, this correlation was not observed for the for the genotype ‘GC2-1’ when sucrose content was increased from stage A to stage C (**Figure 9B**). Another significant increase in glucose and fructose was found due to the increase in the activity of sorbitol oxygenase (SO) and sorbitol dehydrogenase (SDH), where sorbitol was converted to glucose and fructose *via* these two enzymes, which suggest that the accumulation of these sugars mainly comes from sorbitol catalysis. Though there were no significant differences between epicarp and mesocarp, as the sweetest sugar, fructose ratio is significantly higher in the flesh than in the skin, which is consistent with the fact that sweet taste is stronger in the flesh than in the skin (Xi et al., 2016; Zhang et al., 2019). Thus, *SDH* [*LOC103333266* (*PARG19420m03*)], described as a possible key factor during climacteric ripening in Japanese plum (Fernandez i Marti et al., 2018), and *SS* [*LOC103340632* (*PARG27579m02*)] described as the main enzyme responsible for sugar accumulation in apricot fruit (Xi et al., 2016), were both proposed as candidate genes responsible for the increase of sugar content during fruit ripening in apricot.

The red blush present in the skin of apricot was due to the presence of anthocyanins. The major anthocyanin compounds found in apricot fruit are cyanidin-3-O-rutinoside, cyanidin-3-O-glucoside, and peonidin-3-O-rutinoside (Bureau et al., 2009). The early anthocyanin biosynthesis genes (EBGs) are *chalcone synthase* (*CHS*), *chalcone isomerase* (*CHI*), *flavanone 3-hydroxylase* (*F3H*), *flavonoid 3′-hydroxylase* (*F3′H*), *flavonoid 3′5 ′-hydroxylase* (*F3′5 ′H*), and *dihydroflavonol 4-reductase* (*DFR*), which lead to the production of flavonols. The late anthocyanin biosynthesis genes (LBGs) are *anthocyanidin reductase* (*ANR*), *leucoanthocyanidin reductase* (*LAR*), *anthocyanidin synthase/leucoanthocyanidin dioxygenase* (*ANS/LDOX*), and *UDP flavonoid 3-O-glucosyltransferase* (*UFGT*). The TF MYB10 has been implicated in the regulation of the last three enzymes of the metabolic pathway DFR, ANS and UFGT, which were the key to explain contrasting patterns of anthocyanin accumulation in peach and Japanese plum (Ravaglia et al., 2013; Gonzalez et al., 2016). While EBGs are regulated by R2R3-MYB TFs without co-regulators, LBGs need MBW complex (Tanaka et al., 2008; Petroni and Tonelli, 2011; Xu et al., 2015). The expression of flavonoid biosynthesis genes correlated with anthocyanin accumulation and red coloration, but there was some variability in the specific step involved. In most species, only LBGs correlated well with anthocyanin synthesis, such as tomato (Povero et al., 2011) and pepper (Borovsky et al., 2004).

However, in other species the transcript levels not only of LGBs, but also of some EBGs were higher in red compared to non-red fruits, such Chinese bayberry (Niu et al., 2010), apple (Takos et al., 2006a; Takos et al., 2006b), pear (Feng et al., 2010), grape (Boss et al., 1996), cherry (Wei et al., 2015), peach (Ravaglia et al., 2013), Japanese plum (Gonzalez et al., 2016), mei (Zhang et al., 2017a), and apricot (Lin-Wang et al., 2010; Kayesh et al., 2013). In apple, peach, Japanese plum, and sweet cherry, the transcript level of MYB10 was up-regulated during fruit red coloration development (Takos et al., 2006a; Lin-Wang et al., 2011; Cheng et al., 2015; Wei et al., 2015; Gonzalez et al., 2016). Besides, it was described in apricot that anthocyanin biosynthesis is regulated by a *MYB10* TF inducing the expression of *DFR.* Almost the whole precursor pathways for anthocyanin biosynthesis are identified from phenylalanine to cyanidin 3-O-glucoside, the last steps to synthesize cyanidin-3-O-rutinoside, and peonidin-3-O-rutinoside are not yet elucidated, although it is known that the enzymes involved must be of the type UDP-rhamnose as anthocyanidin-3-glucoside rhamnosyl transferase. Similar to this function, we identified some genes with UDP-glucuronosyl/UDP-glucosyltransferase activity like *7-deoxyloganetin glucosyltransferase-like isoform X1* [*LOC103319666* (*PARG08586m01*)] and *7-deoxyloganetin glucosyltransferase-like* [*LOC103323807* (unknown PARG ID)] as possible candidates. Taking all together, the genes found as *MYB* [*LOC103342585* (*PARG26597m01*)], *CHS* [*LOC103324315* (*PARG03336m04*)], *ANS* (*LOC103341429*), *UFGT* (*LOC103323450*, unknown PARG ID), *DFR* [*LOC103320869* (*PARG07267m01*)], *7-deoxyloganetin glucosyltransferase-like isoform X1* [*LOC103319666* (*PARG08586m01*)] and *7-deoxyloganetin glucosyltransferase-like* [*LOC103323807* (unknown PARG ID)] may be the most probable candidate genes responsible for anthocyanin biosynthesis in apricot. Another interesting gene was the TF *bZIP* [*LOC103332281* (*PARG18436m04*)], bind to *chalcone/stilbene synthase* (*CHS*) gene promoter, key enzyme during anthocyanin biosynthesis, and it was up-regulated in ‘GC 2-11’ at the end of ripening process.

In apricot, the amount of carotenoids in the tissues is not attributed solely to the ability to synthesize carotenoids; the regulatory mechanisms based on degradation and accumulation of carotenoids were postulated as responsible for carotenoid content (Marty et al., 2005). We analyze the *carotenoid cleavage dioxygenase 4* (*CCD4*), responsible for the enzymatic cleavage of *β*-carotene resulting in the production of volatile norisoprenoids (apocarotenoids) and related to fruit aroma during ripening (Zhang et al., 2019). This gene cosegregated with the *Y* locus, which seems to control white/yellow flesh in peach, and it was suggested as a molecular marker for white peach cultivar selection (Brandi et al., 2011; Adami et al., 2013; Ma et al., 2014). Further investigation on carotenoid profile compounds and the potential role of *CCD4* allelic segregation in different apricot varieties will be needed to validate this result. We also propose the gene *DXS* [*LOC103335117* (*PARG01985m01*)] as carotenoid biosynthesis precursor.

Concerning texture in peach, the expression of genes involved in softening started before the appearance of the ethylene climacteric rise at S3 stage, regarding the occurrence of the ethylene climacteric. Moreover, some cell wall modifying enzymes expressed in S3 could be involved in cell enlargement associated with the vigorous fruit growth that occurred after stone hardening (Trainotti et al., 2003; Trainotti et al., 2006). Pectins, the main compound responsible for firmness in apricot fruit, comprised a highly complex polysaccharide network with a structurally diverse range of glycan chains, glycosidic linkages, and other substituents, such as acetyl and methyl groups. Most studies of pectin depolymerization have focused on a small group of enzymes: pectin methylesterase (PME), *β*-galactosidase (*β-*GAL), polygalacturonase (PG), and pectin lyases (PL). PL showed maximum expression just before climacteric stage causing massive degradation of pectins and decreasing its expression before ethylene release (Brummell et al., 2004; Vicente et al., 2007). By contrast, the other pectin-degrading enzymes (PG, PME, and *β*-GAL) show a typical ripening-pattern expression increasing its expression at the ripening stage during ethylene release. PME mainly catalyzes the de-esterification of high methyl esterified pectin to generate low methyl esterified pectin, which can be further hydrolyzed by PG (Chen et al., 2000; Vicente et al., 2007). So, PL would appear to have the task to carry out an early degradation of pectins, making them more susceptible to the subsequent attack of other degrading enzymes as PME and *β*-GAL. Other enzymes probably involved were acetyl esterase (AE), rhamnogalacturonan hydrolases, and lyases (RG) and pectin methylesterase inhibitor (PMEI) (Chen et al., 2000; Vicente et al., 2007). PMEIs were found up-regulated in half-ripe and ripe stage during fruit ripening. PMEIs also play a role in plant defense mechanism against pathogens. Their expression do not correlate well with fruit firmness in apricot and may correlate with fruit susceptibility to pathogen attacks (Chen et al., 2000). Expansins (EXPs) also affect pectin depolymerization by non-hydrolytic activity, and one expansin transcript was found increased during apricot ripening, possibly by increasing substrate accessibility to other enzymes (Brummell et al., 2004). Finally, *cinnamyl-alcohol dehydrogenase* (*CAD*), as a precursor for phenylpropanoid compounds in lignin biosynthesis, was described as responsible for cleavage stone by affecting the deposition of lignin in the endocarp of apricot fruit (Zhang et al., 2019). Considering all the above, we proposed as candidate genes for firmness control during ripening process *CAD* [*LOC103335323* (*PARG29726m01*)]*, PMEI* [*LOC103322538* (*PARG05244m01*)] and *βGAL* [*LOC103340681* (*PARG27772m02*)].

## Conclusions

Within the framework of this work, we expanded our knowledge about apricot fruit ripening, providing original information about the dynamic expression of genes involved in the fruit ripening process in two genotypes which differ in fruit color, soluble solid content, and firmness. DEGs in the two assayed apricot genotypes at three fruit ripening stages showed important variation in the biosynthetic pathways of phenylpropanoids, flavonoids, carotenoids biosynthesis and, starch and sucrose metabolism. These genes could be possible candidates as molecular markers for fruit color and soluble solid content. We identified the gene for carotenoid biosynthesis *lycopene β-cyclase* differentially expressed between genotypes but not correlated with orange skin ground color and flesh color or carotenoid content. The gene *carotenoid cleavage dioxygenase 4*, which acts downstream *lycopene β-cyclase* in the carotenoid pathways, degrading *β*-carotene into apocarotenoids compounds, is highly correlated with carotenoid content and correlated with light-yellow/white flesh. On the other hand, sucrose content is mainly due to the expression of the gene *sucrose synthase* in starch and sucrose metabolism. *Carotenoid cleavage dioxygenase 4* and *sucrose synthase* are identified as candidate genes for light yellow/white fruit color and high soluble solid content at the ripening process. This information may be useful to improve agronomical production through the identification of candidate genes involved in fruit ripening and biochemical and metabolite contents that may be applied in monitoring the ripening process in apricot fruit. The expression of these candidate genes was highly correlated with the fruit quality traits of interest and could be implemented in MAS to increase the efficiency of apricot breeding programs by the early selection of new genotypes with high-quality fruits and high nutraceutical values. In addition, results showed the suitability of using different reference genomes and transcriptomes related to *Prunus* species (mainly mei and apricot but also peach) as reference sequences in transcriptomic analysis due to its phylogenetic proximity.

## Data Availability Statement

The datasets generated for this study can be found in the NCBI SRA repository as a Bioproject entitled ‘Prunus armeniaca fruit ripening process’, with the accession number PRJNA562811 (https://www.ncbi.nlm.nih.gov/bioproject/562811).

## Author Contributions

BG-G contributed to laboratory experiments, bioinformatics data analysis, manuscript elaboration, and discussion. DR, MR, and PM-G participated in the design and coordination of the study. DR and JS performed the phenotypic evaluation. PJM-G contributed to bioinformatics data analysis and manuscript elaboration. All authors discussed the results and commented on the manuscript.

## Funding

This study has been supported by the projects “Apricot breeding” (AGL2017-86627-R) from the Spanish Ministry of Economy and Competitiveness and “Breeding stone fruit species assisted by molecular tools” from the Seneca Foundation of the Region of Murcia (19879/GERM/15). The authors offer grateful thanks to Seneca Foundation of the Region of Murcia for financial support to JS in Murcia inside the Saavedra Fajardo program.

## Conflict of Interest

The authors declare that the research was conducted in the absence of any commercial or financial relationships that could be construed as a potential conflict of interest.

The reviewer CC declared a past co-authorship with one of the authors PJM-G to the handling Editor.
