# Supplementary material for: Analysis of Metabolites and Gene Expression Changes Relative to Apricot (Prunus armeniaca L.) Fruit Quality During Development and Ripening
Source: Front Plant Sci. 2020 Aug 19;11:1269. doi: 10.3389/fpls.2020.01269 (PMC7466674; doi:10.3389/fpls.2020.01269)
Supplement: Supplementary file 1 [file DataSheet_1.zip › FastQC_raw/A_S1_L001_R1_001_fastqc/fastqc_report.html]

A\_S1\_L001\_R1\_001.fastq FastQC Report


FastQC Report

jue 31 may 2018  
A\_S1\_L001\_R1\_001.fastq

## Summary

- Basic Statistics
- Per base sequence quality
- Per sequence quality scores
- Per base sequence content
- Per base GC content
- Per sequence GC content
- Per base N content
- Sequence Length Distribution
- Sequence Duplication Levels
- Overrepresented sequences
- Kmer Content

## Basic Statistics

| Measure | Value |
| --- | --- |
| Filename | A\_S1\_L001\_R1\_001.fastq |
| File type | Conventional base calls |
| Encoding | Sanger / Illumina 1.9 |
| Total Sequences | 25712237 |
| Filtered Sequences | 0 |
| Sequence length | 101 |
| %GC | 45 |

## Per base sequence quality

## Per sequence quality scores

## Per base sequence content

## Per base GC content

## Per sequence GC content

## Per base N content

## Sequence Length Distribution

## Sequence Duplication Levels

## Overrepresented sequences

No overrepresented sequences

## Kmer Content

| Sequence | Count | Obs/Exp Overall | Obs/Exp Max | Max Obs/Exp Position |
| --- | --- | --- | --- | --- |
| TCTTC | 9162155 | 2.8976884 | 6.323416 | 7 |
| CTTCT | 8798675 | 2.7827318 | 5.6645474 | 1 |
| TTCTT | 9022660 | 2.5438213 | 5.629228 | 6 |
| CTTCA | 7480145 | 2.3887968 | 7.6059217 | 1 |
| CACCA | 6280410 | 2.271822 | 6.360806 | 1 |
| TCCTC | 6346380 | 2.2515533 | 5.891803 | 2 |
| CTTGG | 4369195 | 2.2244043 | 7.618404 | 1 |
| CTCCA | 6114155 | 2.1903207 | 13.994715 | 1 |
| CCTTG | 5118025 | 2.175137 | 5.3973885 | 1 |
| TCTTG | 5513730 | 2.088948 | 5.201544 | 7 |
| CTTGA | 5371070 | 2.0547454 | 5.7647743 | 1 |
| CTCCT | 5686330 | 2.0173824 | 10.187716 | 1 |
| CTTTG | 5285180 | 2.002359 | 5.4419723 | 1 |
| CTCTG | 4687835 | 1.9923084 | 9.567291 | 1 |
| TCCTT | 6296320 | 1.9913191 | 5.4205093 | 2 |
| CTGCA | 4340225 | 1.8625655 | 5.8634586 | 1 |
| CTCTT | 5884720 | 1.8611435 | 6.7893176 | 1 |
| TCCAA | 5600270 | 1.8058981 | 6.529929 | 2 |
| GTTGG | 2768885 | 1.688672 | 6.513373 | 1 |
| CCTCA | 4712070 | 1.688041 | 5.880838 | 1 |
| TCCAT | 5176670 | 1.6531783 | 5.782958 | 2 |
| TTCAA | 5745240 | 1.6515449 | 5.295841 | 7 |
| CTCTC | 4619455 | 1.6388791 | 5.379745 | 1 |
| CTCAG | 3739605 | 1.6048154 | 7.7922454 | 1 |
| TCCAC | 4311690 | 1.5446098 | 5.087581 | 2 |
| CTCAA | 4777115 | 1.5404583 | 6.049442 | 1 |
| TCCAG | 3586560 | 1.5391376 | 5.450833 | 2 |
| CTGGA | 2929560 | 1.5060167 | 5.3195167 | 1 |
| CCCAA | 4112445 | 1.4876012 | 6.5582376 | 1 |
| GGCAG | 2146735 | 1.4829823 | 5.321805 | 1 |
| CTCAT | 4278595 | 1.3663765 | 6.00807 | 1 |
| CTGGG | 1983035 | 1.3566656 | 5.127888 | 1 |
| CCCAT | 3649285 | 1.3073114 | 6.2228303 | 1 |
| GTGGG | 1577575 | 1.2928869 | 5.121656 | 1 |
| GCCAG | 2132270 | 1.2296221 | 5.235835 | 1 |
| CCCCA | 3001675 | 1.206249 | 6.091327 | 1 |
| CCCAG | 2496815 | 1.2019538 | 6.379902 | 1 |
| GTCCA | 2626745 | 1.1272423 | 7.5290585 | 1 |
| GTCCT | 2550355 | 1.0838892 | 6.322337 | 1 |
| CTCGG | 1432440 | 0.8180707 | 5.0546484 | 1 |
| CTCCG | 1627890 | 0.77608865 | 5.94418 | 1 |
| GTCGG | 989655 | 0.67705864 | 5.0747356 | 1 |

Produced by FastQC (version 0.10.1)
