# Supplementary material for: Analysis of Metabolites and Gene Expression Changes Relative to Apricot (Prunus armeniaca L.) Fruit Quality During Development and Ripening
Source: Front Plant Sci. 2020 Aug 19;11:1269. doi: 10.3389/fpls.2020.01269 (PMC7466674; doi:10.3389/fpls.2020.01269)
Supplement: Supplementary file 1 [file DataSheet_1.zip › FastQC_raw/A_S1_L001_R2_001_fastqc/fastqc_report.html]

A\_S1\_L001\_R2\_001.fastq FastQC Report


FastQC Report

jue 31 may 2018  
A\_S1\_L001\_R2\_001.fastq

## Summary

- Basic Statistics
- Per base sequence quality
- Per sequence quality scores
- Per base sequence content
- Per base GC content
- Per sequence GC content
- Per base N content
- Sequence Length Distribution
- Sequence Duplication Levels
- Overrepresented sequences
- Kmer Content

## Basic Statistics

| Measure | Value |
| --- | --- |
| Filename | A\_S1\_L001\_R2\_001.fastq |
| File type | Conventional base calls |
| Encoding | Sanger / Illumina 1.9 |
| Total Sequences | 25712237 |
| Filtered Sequences | 0 |
| Sequence length | 101 |
| %GC | 45 |

## Per base sequence quality

## Per sequence quality scores

## Per base sequence content

## Per base GC content

## Per sequence GC content

## Per base N content

## Sequence Length Distribution

## Sequence Duplication Levels

## Overrepresented sequences

| Sequence | Count | Percentage | Possible Source |
| --- | --- | --- | --- |
| NNNNNNNNNNNNNNNNNNNNNNNNNNNNNNNNNNNNNNNNNNNNNNNNNN | 40004 | 0.1555835067948386 | No Hit |

## Kmer Content

| Sequence | Count | Obs/Exp Overall | Obs/Exp Max | Max Obs/Exp Position |
| --- | --- | --- | --- | --- |
| CTCTC | 6855505 | 3.7683492 | 6.419019 | 1 |
| TCTCT | 7603300 | 3.2665622 | 5.62175 | 7 |
| GAAGA | 9191015 | 3.1494222 | 8.154971 | 2 |
| TCTTC | 6407175 | 2.752678 | 5.264506 | 7 |
| CTTCT | 5942695 | 2.5531259 | 6.2015038 | 1 |
| GAGAA | 6638565 | 2.2747917 | 5.196932 | 2 |
| GGAAG | 5655870 | 2.2377772 | 6.011957 | 1 |
| CTTCA | 5104605 | 2.1839156 | 7.923474 | 1 |
| CTCCA | 3711390 | 2.0315766 | 5.55096 | 1 |
| CCCAA | 3671460 | 2.0013373 | 5.2900677 | 1 |
| CTCTG | 4004810 | 1.9783612 | 5.4487963 | 1 |
| TTCAA | 5525355 | 1.8399072 | 5.1879168 | 2 |
| TCCAA | 4241790 | 1.8072064 | 5.169867 | 7 |
| CTCAA | 4228800 | 1.801672 | 6.72283 | 1 |
| CTTTG | 4643170 | 1.7927319 | 5.7517633 | 1 |
| GAAAA | 5949685 | 1.7656777 | 5.733715 | 2 |
| GGAAA | 4915085 | 1.6842185 | 5.204911 | 1 |
| CTTGG | 3772435 | 1.6747788 | 5.133286 | 1 |
| CTCAG | 3274950 | 1.6110657 | 5.6680684 | 1 |
| CTTGA | 4078190 | 1.5680256 | 5.9254966 | 1 |

Produced by FastQC (version 0.10.1)
