# Supplementary material for: Analysis of Metabolites and Gene Expression Changes Relative to Apricot (Prunus armeniaca L.) Fruit Quality During Development and Ripening
Source: Front Plant Sci. 2020 Aug 19;11:1269. doi: 10.3389/fpls.2020.01269 (PMC7466674; doi:10.3389/fpls.2020.01269)
Supplement: Supplementary file 1 [file DataSheet_1.zip › FastQC_raw/A_S1_L002_R1_001_fastqc/fastqc_report.html]

A\_S1\_L002\_R1\_001.fastq FastQC Report


FastQC Report

vie 1 jun 2018  
A\_S1\_L002\_R1\_001.fastq

## Summary

- Basic Statistics
- Per base sequence quality
- Per sequence quality scores
- Per base sequence content
- Per base GC content
- Per sequence GC content
- Per base N content
- Sequence Length Distribution
- Sequence Duplication Levels
- Overrepresented sequences
- Kmer Content

## Basic Statistics

| Measure | Value |
| --- | --- |
| Filename | A\_S1\_L002\_R1\_001.fastq |
| File type | Conventional base calls |
| Encoding | Sanger / Illumina 1.9 |
| Total Sequences | 25289134 |
| Filtered Sequences | 0 |
| Sequence length | 101 |
| %GC | 45 |

## Per base sequence quality

## Per sequence quality scores

## Per base sequence content

## Per base GC content

## Per sequence GC content

## Per base N content

## Sequence Length Distribution

## Sequence Duplication Levels

## Overrepresented sequences

No overrepresented sequences

## Kmer Content

| Sequence | Count | Obs/Exp Overall | Obs/Exp Max | Max Obs/Exp Position |
| --- | --- | --- | --- | --- |
| TCTTC | 9004180 | 2.8940372 | 6.3986297 | 7 |
| CTTCT | 8660430 | 2.7835526 | 5.6755967 | 1 |
| TTCTT | 8872405 | 2.5422034 | 5.651128 | 6 |
| CTTCA | 7358720 | 2.389555 | 7.5759044 | 1 |
| CACCA | 6178515 | 2.2737596 | 6.285679 | 1 |
| TCCTC | 6251505 | 2.2539043 | 5.91428 | 2 |
| CTTGG | 4296060 | 2.2213793 | 7.58021 | 1 |
| CTCCA | 6009930 | 2.1891477 | 14.056854 | 1 |
| CCTTG | 5044945 | 2.1782515 | 5.359899 | 1 |
| TCTTG | 5421100 | 2.0866425 | 5.136664 | 7 |
| CTTGA | 5271250 | 2.0498831 | 5.822128 | 1 |
| CTCCT | 5594425 | 2.017002 | 10.184864 | 1 |
| CTTTG | 5196575 | 2.0002203 | 5.3676424 | 1 |
| TCCTT | 6207500 | 1.995155 | 5.360037 | 2 |
| CTCTG | 4619215 | 1.9944345 | 9.587359 | 1 |
| CTGCA | 4265595 | 1.8607415 | 5.8944526 | 1 |
| CTCTT | 5767735 | 1.85381 | 6.706316 | 1 |
| TCCAA | 5499640 | 1.8042794 | 6.512536 | 2 |
| CCTCT | 4790405 | 1.7271224 | 5.010955 | 3 |
| GTTGG | 2729400 | 1.6901306 | 6.4639764 | 1 |
| CCTCA | 4635185 | 1.6883898 | 5.820252 | 1 |
| TCCAT | 5092230 | 1.6535705 | 5.769497 | 2 |
| TTCAA | 5637755 | 1.6488636 | 5.3251023 | 7 |
| CTCTC | 4546815 | 1.639299 | 5.399905 | 1 |
| CTCAG | 3679845 | 1.6052251 | 7.8380904 | 1 |
| TCCAC | 4251875 | 1.5487673 | 5.092299 | 2 |
| CTCAA | 4704680 | 1.5434752 | 6.0804267 | 1 |
| TCCAG | 3526665 | 1.5384047 | 5.513402 | 2 |
| CTGGA | 2882655 | 1.5059125 | 5.347209 | 1 |
| CCCAA | 4037775 | 1.4859445 | 6.6932416 | 1 |
| GGCAG | 2111920 | 1.4820957 | 5.2955847 | 1 |
| CTCAT | 4211965 | 1.3677272 | 5.977699 | 1 |
| CTGGG | 1953795 | 1.3571349 | 5.0995607 | 1 |
| CCCAT | 3591165 | 1.3081003 | 6.139473 | 1 |
| GTGGG | 1549680 | 1.2891004 | 5.10909 | 1 |
| GCCAG | 2101460 | 1.231457 | 5.260412 | 1 |
| CCCCA | 2958155 | 1.2086974 | 6.127264 | 1 |
| CCCAG | 2455160 | 1.2013711 | 6.401055 | 1 |
| GTCCA | 2591190 | 1.1303312 | 7.5231524 | 1 |
| GTCCT | 2512110 | 1.0846517 | 6.212687 | 1 |
| CTCGG | 1413075 | 0.8196124 | 5.135481 | 1 |
| CTCCG | 1600745 | 0.77529097 | 5.933855 | 1 |
| GTCGG | 978080 | 0.6793888 | 5.0708947 | 1 |

Produced by FastQC (version 0.10.1)
