# Supplementary material for: Analysis of Metabolites and Gene Expression Changes Relative to Apricot (Prunus armeniaca L.) Fruit Quality During Development and Ripening
Source: Front Plant Sci. 2020 Aug 19;11:1269. doi: 10.3389/fpls.2020.01269 (PMC7466674; doi:10.3389/fpls.2020.01269)
Supplement: Supplementary file 1 [file DataSheet_1.zip › FastQC_raw/A_S1_L002_R2_001_fastqc/fastqc_report.html]

A\_S1\_L002\_R2\_001.fastq FastQC Report


FastQC Report

vie 1 jun 2018  
A\_S1\_L002\_R2\_001.fastq

## Summary

- Basic Statistics
- Per base sequence quality
- Per sequence quality scores
- Per base sequence content
- Per base GC content
- Per sequence GC content
- Per base N content
- Sequence Length Distribution
- Sequence Duplication Levels
- Overrepresented sequences
- Kmer Content

## Basic Statistics

| Measure | Value |
| --- | --- |
| Filename | A\_S1\_L002\_R2\_001.fastq |
| File type | Conventional base calls |
| Encoding | Sanger / Illumina 1.9 |
| Total Sequences | 25289134 |
| Filtered Sequences | 0 |
| Sequence length | 101 |
| %GC | 45 |

## Per base sequence quality

## Per sequence quality scores

## Per base sequence content

## Per base GC content

## Per sequence GC content

## Per base N content

## Sequence Length Distribution

## Sequence Duplication Levels

## Overrepresented sequences

| Sequence | Count | Percentage | Possible Source |
| --- | --- | --- | --- |
| NNNNNNNNNNNNNNNNNNNNNNNNNNNNNNNNNNNNNNNNNNNNNNNNNN | 41481 | 0.16402696905319097 | No Hit |

## Kmer Content

| Sequence | Count | Obs/Exp Overall | Obs/Exp Max | Max Obs/Exp Position |
| --- | --- | --- | --- | --- |
| CTCTC | 6782920 | 3.7867281 | 6.4511237 | 1 |
| TCTCT | 7518360 | 3.2818031 | 5.659776 | 7 |
| GAAGA | 9033800 | 3.1496286 | 8.162728 | 2 |
| TCTTC | 6303475 | 2.7514994 | 5.291843 | 7 |
| CTTCT | 5842640 | 2.5503426 | 6.1863136 | 1 |
| GAGAA | 6532840 | 2.2776701 | 5.1680975 | 2 |
| GGAAG | 5559080 | 2.237233 | 5.98151 | 1 |
| CTTCA | 5024370 | 2.1845717 | 7.998022 | 1 |
| CTCCA | 3657865 | 2.0340915 | 5.6249814 | 1 |
| CCCAA | 3618795 | 2.0044816 | 5.28525 | 1 |
| CTCTG | 3941800 | 1.97833 | 5.4481006 | 1 |
| TTCAA | 5426845 | 1.8376781 | 5.229242 | 2 |
| TCCAA | 4171440 | 1.8066169 | 5.2302985 | 7 |
| CTCAA | 4157760 | 1.8006922 | 6.738977 | 1 |
| CTTTG | 4566595 | 1.7920027 | 5.8046184 | 1 |
| GAAAA | 5868790 | 1.7726295 | 5.7688394 | 2 |
| GGAAA | 4851885 | 1.6916064 | 5.2380953 | 1 |
| CTTGG | 3711470 | 1.6745837 | 5.075147 | 1 |
| CTCAG | 3223215 | 1.611346 | 5.674433 | 1 |
| CTTGA | 4000420 | 1.563677 | 5.90156 | 1 |

Produced by FastQC (version 0.10.1)
