# Supplementary material for: Analysis of Metabolites and Gene Expression Changes Relative to Apricot (Prunus armeniaca L.) Fruit Quality During Development and Ripening
Source: Front Plant Sci. 2020 Aug 19;11:1269. doi: 10.3389/fpls.2020.01269 (PMC7466674; doi:10.3389/fpls.2020.01269)
Supplement: Supplementary file 1 [file DataSheet_1.zip › FastQC_raw/B_S2_L001_R1_001_fastqc/fastqc_report.html]

B\_S2\_L001\_R1\_001.fastq FastQC Report


FastQC Report

jue 31 may 2018  
B\_S2\_L001\_R1\_001.fastq

## Summary

- Basic Statistics
- Per base sequence quality
- Per sequence quality scores
- Per base sequence content
- Per base GC content
- Per sequence GC content
- Per base N content
- Sequence Length Distribution
- Sequence Duplication Levels
- Overrepresented sequences
- Kmer Content

## Basic Statistics

| Measure | Value |
| --- | --- |
| Filename | B\_S2\_L001\_R1\_001.fastq |
| File type | Conventional base calls |
| Encoding | Sanger / Illumina 1.9 |
| Total Sequences | 28233500 |
| Filtered Sequences | 0 |
| Sequence length | 101 |
| %GC | 44 |

## Per base sequence quality

## Per sequence quality scores

## Per base sequence content

## Per base GC content

## Per sequence GC content

## Per base N content

## Sequence Length Distribution

## Sequence Duplication Levels

## Overrepresented sequences

No overrepresented sequences

## Kmer Content

| Sequence | Count | Obs/Exp Overall | Obs/Exp Max | Max Obs/Exp Position |
| --- | --- | --- | --- | --- |
| TCTTC | 10114065 | 2.8957698 | 6.3939924 | 7 |
| CTTCT | 9719930 | 2.7829244 | 5.663703 | 1 |
| TTCTT | 10028935 | 2.5418813 | 5.5850105 | 6 |
| CTTCA | 8198300 | 2.3779655 | 7.746757 | 1 |
| TCCTC | 6955310 | 2.2495344 | 5.922 | 2 |
| CACCA | 6711045 | 2.227682 | 6.2364297 | 1 |
| CTTGG | 4766100 | 2.2108018 | 7.6069064 | 1 |
| CTCCA | 6695410 | 2.1937988 | 14.224057 | 1 |
| CCTTG | 5580835 | 2.1616256 | 5.2631154 | 1 |
| TCTTG | 6073375 | 2.0824456 | 5.2236657 | 7 |
| CTTGA | 5893080 | 2.0470543 | 5.9029574 | 1 |
| CTCCT | 6191125 | 2.002376 | 10.3031845 | 1 |
| CTCTG | 5126700 | 1.985726 | 9.867027 | 1 |
| CTTTG | 5787430 | 1.9844004 | 5.585333 | 1 |
| TCCTT | 6905175 | 1.9770288 | 5.410131 | 2 |
| CTCTT | 6464530 | 1.8508673 | 6.8497176 | 1 |
| CTGCA | 4705210 | 1.8463069 | 5.8111525 | 1 |
| TCCAA | 6164285 | 1.8113732 | 6.52501 | 2 |
| GTTGG | 3055290 | 1.6972443 | 6.686829 | 1 |
| CCTCA | 5128195 | 1.6802893 | 5.6625433 | 1 |
| CTCTC | 5187810 | 1.6778773 | 5.5225363 | 1 |
| TCCAT | 5748680 | 1.6674387 | 5.9436693 | 2 |
| TTCAA | 6358335 | 1.6539822 | 5.3188004 | 7 |
| CTCAG | 4072575 | 1.5980633 | 7.9727817 | 1 |
| TCCAC | 4735915 | 1.5517563 | 5.19099 | 2 |
| CTCAA | 5278775 | 1.5511664 | 6.0589585 | 1 |
| TCCAG | 3909945 | 1.5342479 | 5.5757346 | 2 |
| CTGGA | 3197400 | 1.5025436 | 5.240662 | 1 |
| GGCAG | 2326095 | 1.4787701 | 5.4660044 | 1 |
| CCCAA | 4454515 | 1.4786435 | 6.5483155 | 1 |
| CTGGG | 2189545 | 1.37399 | 5.2403398 | 1 |
| CTCAT | 4682885 | 1.3582985 | 6.2765026 | 1 |
| CCCAT | 4005235 | 1.3123436 | 6.2690344 | 1 |
| GTGGG | 1743925 | 1.3105778 | 5.4758525 | 1 |
| CCCCA | 3327905 | 1.2317663 | 6.1532545 | 1 |
| CCCAG | 2759205 | 1.2230568 | 6.4628844 | 1 |
| GCCAG | 2261305 | 1.2004031 | 5.2439203 | 1 |
| GTCCA | 2925415 | 1.147922 | 7.81216 | 1 |
| GGGGG | 1089220 | 1.1073724 | 5.1010118 | 1 |
| GTCCT | 2794355 | 1.0823382 | 6.4126363 | 1 |
| GTCAG | 1851480 | 0.87005985 | 5.161255 | 1 |
| CTCGG | 1545650 | 0.8099079 | 5.1220245 | 1 |
| CTCCG | 1804075 | 0.78935754 | 5.9521947 | 1 |
| GTCGG | 1093700 | 0.686322 | 5.2759905 | 1 |

Produced by FastQC (version 0.10.1)
