# Supplementary material for: Analysis of Metabolites and Gene Expression Changes Relative to Apricot (Prunus armeniaca L.) Fruit Quality During Development and Ripening
Source: Front Plant Sci. 2020 Aug 19;11:1269. doi: 10.3389/fpls.2020.01269 (PMC7466674; doi:10.3389/fpls.2020.01269)
Supplement: Supplementary file 1 [file DataSheet_1.zip › FastQC_raw/B_S2_L001_R2_001_fastqc/fastqc_report.html]

B\_S2\_L001\_R2\_001.fastq FastQC Report


FastQC Report

jue 31 may 2018  
B\_S2\_L001\_R2\_001.fastq

## Summary

- Basic Statistics
- Per base sequence quality
- Per sequence quality scores
- Per base sequence content
- Per base GC content
- Per sequence GC content
- Per base N content
- Sequence Length Distribution
- Sequence Duplication Levels
- Overrepresented sequences
- Kmer Content

## Basic Statistics

| Measure | Value |
| --- | --- |
| Filename | B\_S2\_L001\_R2\_001.fastq |
| File type | Conventional base calls |
| Encoding | Sanger / Illumina 1.9 |
| Total Sequences | 28233500 |
| Filtered Sequences | 0 |
| Sequence length | 101 |
| %GC | 45 |

## Per base sequence quality

## Per sequence quality scores

## Per base sequence content

## Per base GC content

## Per sequence GC content

## Per base N content

## Sequence Length Distribution

## Sequence Duplication Levels

## Overrepresented sequences

| Sequence | Count | Percentage | Possible Source |
| --- | --- | --- | --- |
| NNNNNNNNNNNNNNNNNNNNNNNNNNNNNNNNNNNNNNNNNNNNNNNNNN | 43678 | 0.15470274673703224 | No Hit |

## Kmer Content

| Sequence | Count | Obs/Exp Overall | Obs/Exp Max | Max Obs/Exp Position |
| --- | --- | --- | --- | --- |
| CTCTC | 7858060 | 3.9542193 | 6.649087 | 1 |
| TCTCT | 8763140 | 3.4354665 | 5.7792926 | 7 |
| GAAGA | 10131840 | 3.1464367 | 8.112704 | 2 |
| TCTTC | 7070670 | 2.7719574 | 5.2679687 | 7 |
| CTTCT | 6615300 | 2.593436 | 6.3496194 | 1 |
| GAGAA | 7423810 | 2.3054597 | 5.3984756 | 2 |
| GGAAG | 6187985 | 2.2359629 | 6.11223 | 1 |
| CTTCA | 5610710 | 2.1835275 | 7.886673 | 1 |
| CTCCA | 4099360 | 2.0477474 | 5.691742 | 1 |
| CCCAA | 4074180 | 2.0202978 | 5.2371006 | 1 |
| CTCTG | 4367175 | 1.9775435 | 5.2972693 | 1 |
| CAACA | 4865370 | 1.8658916 | 5.133098 | 1 |
| TTCAA | 6094880 | 1.834431 | 5.1561813 | 2 |
| CTCAA | 4715265 | 1.8216369 | 6.9522986 | 1 |
| TCCAA | 4660865 | 1.8006207 | 5.3094573 | 7 |
| CTTTG | 5088090 | 1.7949849 | 5.743786 | 1 |
| GAAAA | 6682280 | 1.7834902 | 5.7886715 | 2 |
| GGAAA | 5461650 | 1.696112 | 5.20443 | 1 |
| CTTGG | 4075215 | 1.6605656 | 5.076819 | 1 |
| CTCAG | 3573850 | 1.606485 | 5.8203115 | 1 |
| CTTGA | 4475150 | 1.5672146 | 5.9485006 | 1 |
| GTTTG | 4660895 | 1.4796377 | 5.01327 | 1 |

Produced by FastQC (version 0.10.1)
