# Supplementary material for: Analysis of Metabolites and Gene Expression Changes Relative to Apricot (Prunus armeniaca L.) Fruit Quality During Development and Ripening
Source: Front Plant Sci. 2020 Aug 19;11:1269. doi: 10.3389/fpls.2020.01269 (PMC7466674; doi:10.3389/fpls.2020.01269)
Supplement: Supplementary file 1 [file DataSheet_1.zip › FastQC_raw/B_S2_L002_R1_001_fastqc/fastqc_report.html]

B\_S2\_L002\_R1\_001.fastq FastQC Report


FastQC Report

jue 31 may 2018  
B\_S2\_L002\_R1\_001.fastq

## Summary

- Basic Statistics
- Per base sequence quality
- Per sequence quality scores
- Per base sequence content
- Per base GC content
- Per sequence GC content
- Per base N content
- Sequence Length Distribution
- Sequence Duplication Levels
- Overrepresented sequences
- Kmer Content

## Basic Statistics

| Measure | Value |
| --- | --- |
| Filename | B\_S2\_L002\_R1\_001.fastq |
| File type | Conventional base calls |
| Encoding | Sanger / Illumina 1.9 |
| Total Sequences | 27717702 |
| Filtered Sequences | 0 |
| Sequence length | 101 |
| %GC | 44 |

## Per base sequence quality

## Per sequence quality scores

## Per base sequence content

## Per base GC content

## Per sequence GC content

## Per base N content

## Sequence Length Distribution

## Sequence Duplication Levels

## Overrepresented sequences

No overrepresented sequences

## Kmer Content

| Sequence | Count | Obs/Exp Overall | Obs/Exp Max | Max Obs/Exp Position |
| --- | --- | --- | --- | --- |
| TCTTC | 9970845 | 2.9049315 | 6.401993 | 7 |
| CTTCT | 9579405 | 2.7908883 | 5.610682 | 1 |
| TTCTT | 9866200 | 2.5451407 | 5.6078835 | 6 |
| CTTCA | 8073395 | 2.3846018 | 7.7136054 | 1 |
| TCCTC | 6848915 | 2.253553 | 5.973758 | 2 |
| CACCA | 6585405 | 2.2271008 | 6.2464957 | 1 |
| CTTGG | 4681995 | 2.209789 | 7.619542 | 1 |
| CTCCA | 6581890 | 2.1955955 | 14.327085 | 1 |
| CCTTG | 5484290 | 2.1612418 | 5.267748 | 1 |
| TCTTG | 5957210 | 2.0786617 | 5.1554246 | 7 |
| CTTGA | 5776555 | 2.0434568 | 5.8641157 | 1 |
| CTCCT | 6091560 | 2.0043545 | 10.298507 | 1 |
| CTCTG | 5050605 | 1.9903357 | 9.94562 | 1 |
| CTTTG | 5684335 | 1.9834468 | 5.4527073 | 1 |
| TCCTT | 6784160 | 1.9765145 | 5.342801 | 2 |
| CTCTT | 6372620 | 1.8566154 | 6.895771 | 1 |
| CTGCA | 4621075 | 1.8462125 | 5.7600503 | 1 |
| TCCAA | 6045770 | 1.8103684 | 6.6027975 | 2 |
| GTTGG | 2994340 | 1.6926141 | 6.5790834 | 1 |
| CTCTC | 5105270 | 1.6798276 | 5.595684 | 1 |
| CCTCA | 5035105 | 1.6796168 | 5.695138 | 1 |
| TCCAT | 5650125 | 1.6688516 | 5.923967 | 2 |
| TTCAA | 6235055 | 1.653155 | 5.326195 | 7 |
| CTCAG | 4006850 | 1.6008171 | 7.9771695 | 1 |
| CTCAA | 5194375 | 1.5554235 | 6.2109933 | 1 |
| TCCAC | 4649325 | 1.5509279 | 5.1994324 | 2 |
| TCCAG | 3846975 | 1.5369438 | 5.5972733 | 2 |
| CTGGA | 3139505 | 1.5022314 | 5.298208 | 1 |
| GGCAG | 2284680 | 1.4786996 | 5.416208 | 1 |
| CCCAA | 4368420 | 1.4773445 | 6.4840865 | 1 |
| CTGGG | 2150035 | 1.3726015 | 5.2742496 | 1 |
| CTCAT | 4594565 | 1.3570757 | 6.238686 | 1 |
| CCCAT | 3933715 | 1.3122138 | 6.302647 | 1 |
| GTGGG | 1709025 | 1.306725 | 5.5194097 | 1 |
| CCCCA | 3267265 | 1.2309152 | 6.14556 | 1 |
| CCCAG | 2717095 | 1.225986 | 6.4036655 | 1 |
| GCCAG | 2226890 | 1.2034177 | 5.3015428 | 1 |
| GTCCA | 2870130 | 1.1466748 | 7.798714 | 1 |
| GGGGG | 1073510 | 1.1102502 | 5.0971556 | 1 |
| GTCCT | 2739540 | 1.0795944 | 6.3943105 | 1 |
| GTCAG | 1812000 | 0.8670293 | 5.1232734 | 1 |
| CTCGG | 1520730 | 0.8106139 | 5.1118402 | 1 |
| CTCCG | 1772880 | 0.7890496 | 6.002473 | 1 |
| GTCGG | 1065565 | 0.6802662 | 5.316404 | 1 |

Produced by FastQC (version 0.10.1)
