# Supplementary material for: Analysis of Metabolites and Gene Expression Changes Relative to Apricot (Prunus armeniaca L.) Fruit Quality During Development and Ripening
Source: Front Plant Sci. 2020 Aug 19;11:1269. doi: 10.3389/fpls.2020.01269 (PMC7466674; doi:10.3389/fpls.2020.01269)
Supplement: Supplementary file 1 [file DataSheet_1.zip › FastQC_raw/B_S2_L002_R2_001_fastqc/fastqc_report.html]

B\_S2\_L002\_R2\_001.fastq FastQC Report


FastQC Report

jue 31 may 2018  
B\_S2\_L002\_R2\_001.fastq

## Summary

- Basic Statistics
- Per base sequence quality
- Per sequence quality scores
- Per base sequence content
- Per base GC content
- Per sequence GC content
- Per base N content
- Sequence Length Distribution
- Sequence Duplication Levels
- Overrepresented sequences
- Kmer Content

## Basic Statistics

| Measure | Value |
| --- | --- |
| Filename | B\_S2\_L002\_R2\_001.fastq |
| File type | Conventional base calls |
| Encoding | Sanger / Illumina 1.9 |
| Total Sequences | 27717702 |
| Filtered Sequences | 0 |
| Sequence length | 101 |
| %GC | 45 |

## Per base sequence quality

## Per sequence quality scores

## Per base sequence content

## Per base GC content

## Per sequence GC content

## Per base N content

## Sequence Length Distribution

## Sequence Duplication Levels

## Overrepresented sequences

| Sequence | Count | Percentage | Possible Source |
| --- | --- | --- | --- |
| NNNNNNNNNNNNNNNNNNNNNNNNNNNNNNNNNNNNNNNNNNNNNNNNNN | 45067 | 0.1625928440965272 | No Hit |

## Kmer Content

| Sequence | Count | Obs/Exp Overall | Obs/Exp Max | Max Obs/Exp Position |
| --- | --- | --- | --- | --- |
| CTCTC | 7762215 | 3.9736087 | 6.6552205 | 1 |
| TCTCT | 8651860 | 3.4534907 | 5.7305946 | 7 |
| GAAGA | 9974210 | 3.1568453 | 8.176361 | 2 |
| TCTTC | 6960385 | 2.778319 | 5.3184457 | 7 |
| CTTCT | 6500925 | 2.59492 | 6.3268766 | 1 |
| GAGAA | 7292270 | 2.3080096 | 5.43821 | 2 |
| GGAAG | 6078905 | 2.2378638 | 6.0880537 | 1 |
| CTTCA | 5515120 | 2.1853242 | 8.005872 | 1 |
| CTCCA | 4022605 | 2.0441792 | 5.7158766 | 1 |
| CCCAA | 4011875 | 2.0238159 | 5.2623477 | 1 |
| CTCTG | 4311130 | 1.9869432 | 5.368512 | 1 |
| CAACA | 4787595 | 1.8694011 | 5.213297 | 1 |
| TTCAA | 5976985 | 1.8331774 | 5.18477 | 2 |
| CTCAA | 4629085 | 1.8208246 | 6.889344 | 1 |
| TCCAA | 4586115 | 1.8039227 | 5.308658 | 7 |
| CTTTG | 4988700 | 1.792797 | 5.7000237 | 1 |
| GAAAA | 6560740 | 1.7852302 | 5.86543 | 2 |
| GGAAA | 5357530 | 1.6956625 | 5.242276 | 1 |
| CTTGG | 4001615 | 1.6604452 | 5.0995383 | 1 |
| CTCAG | 3507475 | 1.6047263 | 5.801677 | 1 |
| CTTGA | 4389770 | 1.566021 | 6.004895 | 1 |

Produced by FastQC (version 0.10.1)
