# Supplementary material for: Analysis of Metabolites and Gene Expression Changes Relative to Apricot (Prunus armeniaca L.) Fruit Quality During Development and Ripening
Source: Front Plant Sci. 2020 Aug 19;11:1269. doi: 10.3389/fpls.2020.01269 (PMC7466674; doi:10.3389/fpls.2020.01269)
Supplement: Supplementary file 1 [file DataSheet_1.zip › FastQC_raw/C_S3_L001_R1_001_fastqc/fastqc_report.html]

C\_S3\_L001\_R1\_001.fastq FastQC Report


FastQC Report

jue 31 may 2018  
C\_S3\_L001\_R1\_001.fastq

## Summary

- Basic Statistics
- Per base sequence quality
- Per sequence quality scores
- Per base sequence content
- Per base GC content
- Per sequence GC content
- Per base N content
- Sequence Length Distribution
- Sequence Duplication Levels
- Overrepresented sequences
- Kmer Content

## Basic Statistics

| Measure | Value |
| --- | --- |
| Filename | C\_S3\_L001\_R1\_001.fastq |
| File type | Conventional base calls |
| Encoding | Sanger / Illumina 1.9 |
| Total Sequences | 24618718 |
| Filtered Sequences | 0 |
| Sequence length | 101 |
| %GC | 45 |

## Per base sequence quality

## Per sequence quality scores

## Per base sequence content

## Per base GC content

## Per sequence GC content

## Per base N content

## Sequence Length Distribution

## Sequence Duplication Levels

## Overrepresented sequences

No overrepresented sequences

## Kmer Content

| Sequence | Count | Obs/Exp Overall | Obs/Exp Max | Max Obs/Exp Position |
| --- | --- | --- | --- | --- |
| TCTTC | 8790350 | 2.8401654 | 6.1135817 | 7 |
| CTTCT | 8468375 | 2.7361352 | 5.7664723 | 1 |
| TTCTT | 8777925 | 2.5233731 | 5.48188 | 6 |
| CTTCA | 7236335 | 2.380681 | 7.8374057 | 1 |
| TCCTC | 6178385 | 2.2436767 | 6.0350375 | 2 |
| CACCA | 5907700 | 2.224303 | 6.3242383 | 1 |
| CTCCA | 5922130 | 2.1898193 | 14.430802 | 1 |
| CTTGG | 4095460 | 2.1772304 | 7.439955 | 1 |
| CCTTG | 4873950 | 2.1415317 | 5.2873964 | 1 |
| TCTTG | 5275495 | 2.0623333 | 5.0391836 | 7 |
| CTTGA | 5106805 | 2.0327785 | 5.7927732 | 1 |
| CTCCT | 5536190 | 2.0104637 | 10.556258 | 1 |
| CTTTG | 5073570 | 1.9833953 | 5.477613 | 1 |
| TCCTT | 6103655 | 1.9720932 | 5.4072404 | 2 |
| CTCTG | 4449550 | 1.9550575 | 9.998967 | 1 |
| CTGCA | 4129485 | 1.8475002 | 5.7860985 | 1 |
| CTCTT | 5635405 | 1.8208015 | 6.8488235 | 1 |
| TCCAA | 5398130 | 1.8083019 | 6.820969 | 2 |
| CCTCA | 4540730 | 1.6790205 | 5.738997 | 1 |
| CTCTC | 4605010 | 1.6723065 | 5.5133557 | 1 |
| GTTGG | 2594370 | 1.668752 | 6.1102934 | 1 |
| TCCAT | 5038950 | 1.6577634 | 5.8829618 | 2 |
| TTCAA | 5535455 | 1.6498071 | 5.187362 | 7 |
| CTCAG | 3523650 | 1.5764539 | 8.035801 | 1 |
| CTCAA | 4644950 | 1.5559967 | 6.3138485 | 1 |
| TCCAG | 3475880 | 1.5550821 | 5.681342 | 2 |
| TCCAC | 4175645 | 1.5440235 | 5.1048217 | 2 |
| CTGGA | 2780170 | 1.5049363 | 5.215795 | 1 |
| GGCAG | 2026400 | 1.4916879 | 5.117042 | 1 |
| CCCAA | 3858395 | 1.4527209 | 6.486764 | 1 |
| CTGGG | 1905495 | 1.3775758 | 5.1637464 | 1 |
| CTCAT | 4095055 | 1.3472315 | 6.1048117 | 1 |
| CCCAT | 3470495 | 1.2832811 | 6.035248 | 1 |
| CCCAG | 2420045 | 1.2169139 | 6.4222355 | 1 |
| CCCCA | 2890505 | 1.2013012 | 6.057856 | 1 |
| GTCCA | 2564665 | 1.1474115 | 7.5396323 | 1 |
| GTCCT | 2433880 | 1.0694059 | 6.106652 | 1 |
| CTCCG | 1617490 | 0.79879045 | 6.2485156 | 1 |

Produced by FastQC (version 0.10.1)
