# Supplementary material for: Analysis of Metabolites and Gene Expression Changes Relative to Apricot (Prunus armeniaca L.) Fruit Quality During Development and Ripening
Source: Front Plant Sci. 2020 Aug 19;11:1269. doi: 10.3389/fpls.2020.01269 (PMC7466674; doi:10.3389/fpls.2020.01269)
Supplement: Supplementary file 1 [file DataSheet_1.zip › FastQC_raw/C_S3_L001_R2_001_fastqc/fastqc_report.html]

C\_S3\_L001\_R2\_001.fastq FastQC Report


FastQC Report

jue 31 may 2018  
C\_S3\_L001\_R2\_001.fastq

## Summary

- Basic Statistics
- Per base sequence quality
- Per sequence quality scores
- Per base sequence content
- Per base GC content
- Per sequence GC content
- Per base N content
- Sequence Length Distribution
- Sequence Duplication Levels
- Overrepresented sequences
- Kmer Content

## Basic Statistics

| Measure | Value |
| --- | --- |
| Filename | C\_S3\_L001\_R2\_001.fastq |
| File type | Conventional base calls |
| Encoding | Sanger / Illumina 1.9 |
| Total Sequences | 24618718 |
| Filtered Sequences | 0 |
| Sequence length | 101 |
| %GC | 45 |

## Per base sequence quality

## Per sequence quality scores

## Per base sequence content

## Per base GC content

## Per sequence GC content

## Per base N content

## Sequence Length Distribution

## Sequence Duplication Levels

## Overrepresented sequences

| Sequence | Count | Percentage | Possible Source |
| --- | --- | --- | --- |
| NNNNNNNNNNNNNNNNNNNNNNNNNNNNNNNNNNNNNNNNNNNNNNNNNN | 38581 | 0.15671409047376064 | No Hit |

## Kmer Content

| Sequence | Count | Obs/Exp Overall | Obs/Exp Max | Max Obs/Exp Position |
| --- | --- | --- | --- | --- |
| CTCTC | 6555715 | 3.8309693 | 6.5936813 | 1 |
| TCTCT | 7367230 | 3.3656116 | 5.6819487 | 7 |
| GAAGA | 8795490 | 3.092253 | 8.000376 | 2 |
| TCTTC | 6104345 | 2.7886808 | 5.242933 | 7 |
| CTTCT | 5707415 | 2.6073492 | 6.567313 | 1 |
| GAGAA | 6479580 | 2.2780426 | 5.437001 | 2 |
| GGAAG | 5442420 | 2.2154508 | 5.8252544 | 1 |
| CTTCA | 4869690 | 2.1969573 | 8.096679 | 1 |
| CTCCA | 3519450 | 2.0310657 | 5.632217 | 1 |
| CCCAA | 3537295 | 2.0159557 | 5.3157635 | 1 |
| CTCTG | 3824550 | 1.997817 | 5.4510827 | 1 |
| TTCAA | 5263065 | 1.8331195 | 5.148966 | 2 |
| CTCAA | 4091710 | 1.822996 | 6.842761 | 1 |
| TCCAA | 4059285 | 1.8085495 | 5.155788 | 7 |
| CTTTG | 4428725 | 1.8085278 | 5.833246 | 1 |
| GAAAA | 5779340 | 1.7548424 | 5.6371903 | 2 |
| GGAAA | 4787190 | 1.6830446 | 5.031405 | 1 |
| CTTGG | 3557840 | 1.6613019 | 5.0727262 | 1 |
| CTCAG | 3100035 | 1.5991989 | 5.664284 | 1 |
| CTTGA | 3892900 | 1.5699301 | 5.958637 | 1 |

Produced by FastQC (version 0.10.1)
