# Supplementary material for: Analysis of Metabolites and Gene Expression Changes Relative to Apricot (Prunus armeniaca L.) Fruit Quality During Development and Ripening
Source: Front Plant Sci. 2020 Aug 19;11:1269. doi: 10.3389/fpls.2020.01269 (PMC7466674; doi:10.3389/fpls.2020.01269)
Supplement: Supplementary file 1 [file DataSheet_1.zip › FastQC_raw/C_S3_L002_R1_001_fastqc/fastqc_report.html]

C\_S3\_L002\_R1\_001.fastq FastQC Report


FastQC Report

jue 31 may 2018  
C\_S3\_L002\_R1\_001.fastq

## Summary

- Basic Statistics
- Per base sequence quality
- Per sequence quality scores
- Per base sequence content
- Per base GC content
- Per sequence GC content
- Per base N content
- Sequence Length Distribution
- Sequence Duplication Levels
- Overrepresented sequences
- Kmer Content

## Basic Statistics

| Measure | Value |
| --- | --- |
| Filename | C\_S3\_L002\_R1\_001.fastq |
| File type | Conventional base calls |
| Encoding | Sanger / Illumina 1.9 |
| Total Sequences | 24195543 |
| Filtered Sequences | 0 |
| Sequence length | 101 |
| %GC | 45 |

## Per base sequence quality

## Per sequence quality scores

## Per base sequence content

## Per base GC content

## Per sequence GC content

## Per base N content

## Sequence Length Distribution

## Sequence Duplication Levels

## Overrepresented sequences

No overrepresented sequences

## Kmer Content

| Sequence | Count | Obs/Exp Overall | Obs/Exp Max | Max Obs/Exp Position |
| --- | --- | --- | --- | --- |
| TCTTC | 8644275 | 2.84021 | 6.098313 | 7 |
| CTTCT | 8341600 | 2.7407615 | 5.800948 | 1 |
| TTCTT | 8638205 | 2.5259316 | 5.508451 | 6 |
| CTTCA | 7123240 | 2.3839111 | 7.858563 | 1 |
| TCCTC | 6073125 | 2.2421157 | 6.0088964 | 2 |
| CACCA | 5819995 | 2.2292037 | 6.371356 | 1 |
| CTCCA | 5841815 | 2.1967683 | 14.47825 | 1 |
| CTTGG | 4029470 | 2.178443 | 7.4652653 | 1 |
| CCTTG | 4788900 | 2.139478 | 5.2645254 | 1 |
| TCTTG | 5186165 | 2.0620277 | 5.079767 | 7 |
| CTTGA | 5011025 | 2.0293891 | 5.78376 | 1 |
| CTCCT | 5443115 | 2.0095243 | 10.567961 | 1 |
| CTTTG | 4984565 | 1.9818711 | 5.4007134 | 1 |
| TCCTT | 6003355 | 1.9724952 | 5.4607286 | 2 |
| CTCTG | 4378905 | 1.9563096 | 9.953576 | 1 |
| CTGCA | 4057075 | 1.8461872 | 5.7973213 | 1 |
| CTCTT | 5540735 | 1.8204942 | 6.85687 | 1 |
| TCCAA | 5305455 | 1.8085306 | 6.868004 | 2 |
| CCTCA | 4468135 | 1.6802068 | 5.784873 | 1 |
| GTTGG | 2552575 | 1.6699483 | 6.179782 | 1 |
| CTCTC | 4513305 | 1.6662513 | 5.5679154 | 1 |
| TCCAT | 4960350 | 1.6600641 | 5.876044 | 2 |
| TTCAA | 5441095 | 1.6506904 | 5.2096357 | 7 |
| CTCAG | 3472615 | 1.5802265 | 8.01824 | 1 |
| TCCAG | 3421740 | 1.5570757 | 5.638668 | 2 |
| CTCAA | 4567200 | 1.5568732 | 6.300251 | 1 |
| TCCAC | 4105395 | 1.5438013 | 5.091492 | 2 |
| CTGGA | 2732030 | 1.5044384 | 5.185298 | 1 |
| GGCAG | 2000375 | 1.4977885 | 5.159774 | 1 |
| CCCAA | 3806475 | 1.4579751 | 6.471222 | 1 |
| CTGGG | 1869385 | 1.3741912 | 5.1738515 | 1 |
| CTCAT | 4032790 | 1.3496406 | 6.172717 | 1 |
| CCCAT | 3427785 | 1.2889916 | 6.1239247 | 1 |
| GTGGG | 1447295 | 1.287454 | 5.0360565 | 1 |
| CCCAG | 2379610 | 1.2167249 | 6.374035 | 1 |
| CCCCA | 2846580 | 1.2027732 | 6.051601 | 1 |
| GTCCA | 2525640 | 1.149302 | 7.480023 | 1 |
| GTCCT | 2393640 | 1.0693771 | 6.149104 | 1 |
| CTCCC | 2548130 | 1.0570396 | 5.0337086 | 1 |
| CTCCG | 1586930 | 0.79662454 | 6.142301 | 1 |

Produced by FastQC (version 0.10.1)
