# Supplementary material for: Analysis of Metabolites and Gene Expression Changes Relative to Apricot (Prunus armeniaca L.) Fruit Quality During Development and Ripening
Source: Front Plant Sci. 2020 Aug 19;11:1269. doi: 10.3389/fpls.2020.01269 (PMC7466674; doi:10.3389/fpls.2020.01269)
Supplement: Supplementary file 1 [file DataSheet_1.zip › FastQC_raw/C_S3_L002_R2_001_fastqc/fastqc_report.html]

C\_S3\_L002\_R2\_001.fastq FastQC Report


FastQC Report

jue 31 may 2018  
C\_S3\_L002\_R2\_001.fastq

## Summary

- Basic Statistics
- Per base sequence quality
- Per sequence quality scores
- Per base sequence content
- Per base GC content
- Per sequence GC content
- Per base N content
- Sequence Length Distribution
- Sequence Duplication Levels
- Overrepresented sequences
- Kmer Content

## Basic Statistics

| Measure | Value |
| --- | --- |
| Filename | C\_S3\_L002\_R2\_001.fastq |
| File type | Conventional base calls |
| Encoding | Sanger / Illumina 1.9 |
| Total Sequences | 24195543 |
| Filtered Sequences | 0 |
| Sequence length | 101 |
| %GC | 45 |

## Per base sequence quality

## Per sequence quality scores

## Per base sequence content

## Per base GC content

## Per sequence GC content

## Per base N content

## Sequence Length Distribution

## Sequence Duplication Levels

## Overrepresented sequences

| Sequence | Count | Percentage | Possible Source |
| --- | --- | --- | --- |
| NNNNNNNNNNNNNNNNNNNNNNNNNNNNNNNNNNNNNNNNNNNNNNNNNN | 39671 | 0.16395994915261872 | No Hit |

## Kmer Content

| Sequence | Count | Obs/Exp Overall | Obs/Exp Max | Max Obs/Exp Position |
| --- | --- | --- | --- | --- |
| CTCTC | 6465220 | 3.8398414 | 6.613899 | 1 |
| TCTCT | 7251690 | 3.368196 | 5.6175904 | 7 |
| GAAGA | 8659560 | 3.0998895 | 8.022198 | 2 |
| TCTTC | 6007835 | 2.7904623 | 5.251314 | 7 |
| CTTCT | 5610355 | 2.6058443 | 6.5559525 | 1 |
| GAGAA | 6364810 | 2.2784312 | 5.376741 | 2 |
| GGAAG | 5352995 | 2.2176719 | 5.8715286 | 1 |
| CTTCA | 4795755 | 2.2005048 | 8.090999 | 1 |
| CTCCA | 3466180 | 2.0337064 | 5.5447054 | 1 |
| CCCAA | 3484660 | 2.0197833 | 5.3101916 | 1 |
| CTCTG | 3757055 | 1.9950917 | 5.5171666 | 1 |
| TTCAA | 5175205 | 1.8345444 | 5.1931143 | 2 |
| CTCAA | 4025505 | 1.8247058 | 6.847678 | 1 |
| TCCAA | 3994415 | 1.8106132 | 5.209942 | 7 |
| CTTTG | 4356070 | 1.808999 | 5.8235807 | 1 |
| GAAAA | 5678465 | 1.7564322 | 5.6207666 | 2 |
| GGAAA | 4715735 | 1.6881063 | 5.0888844 | 1 |
| CTTGG | 3498950 | 1.661263 | 5.0197473 | 1 |
| CTCAG | 3048015 | 1.5989674 | 5.690639 | 1 |
| CTTGA | 3837440 | 1.5743172 | 5.987747 | 1 |

Produced by FastQC (version 0.10.1)
