# Supplementary material for: Analysis of Metabolites and Gene Expression Changes Relative to Apricot (Prunus armeniaca L.) Fruit Quality During Development and Ripening
Source: Front Plant Sci. 2020 Aug 19;11:1269. doi: 10.3389/fpls.2020.01269 (PMC7466674; doi:10.3389/fpls.2020.01269)
Supplement: Supplementary file 1 [file DataSheet_1.zip › FastQC_raw/D_S4_L001_R1_001_fastqc/fastqc_report.html]

D\_S4\_L001\_R1\_001.fastq FastQC Report


FastQC Report

dom 3 jun 2018  
D\_S4\_L001\_R1\_001.fastq

## Summary

- Basic Statistics
- Per base sequence quality
- Per sequence quality scores
- Per base sequence content
- Per base GC content
- Per sequence GC content
- Per base N content
- Sequence Length Distribution
- Sequence Duplication Levels
- Overrepresented sequences
- Kmer Content

## Basic Statistics

| Measure | Value |
| --- | --- |
| Filename | D\_S4\_L001\_R1\_001.fastq |
| File type | Conventional base calls |
| Encoding | Sanger / Illumina 1.9 |
| Total Sequences | 27913080 |
| Filtered Sequences | 0 |
| Sequence length | 101 |
| %GC | 44 |

## Per base sequence quality

## Per sequence quality scores

## Per base sequence content

## Per base GC content

## Per sequence GC content

## Per base N content

## Sequence Length Distribution

## Sequence Duplication Levels

## Overrepresented sequences

No overrepresented sequences

## Kmer Content

| Sequence | Count | Obs/Exp Overall | Obs/Exp Max | Max Obs/Exp Position |
| --- | --- | --- | --- | --- |
| TCTTC | 9863285 | 2.852516 | 6.322021 | 7 |
| CTTCT | 9532615 | 2.7568843 | 5.6564326 | 1 |
| TTCTT | 9894665 | 2.5313525 | 5.710668 | 6 |
| CTTCA | 8079645 | 2.3627717 | 7.6283097 | 1 |
| CTTGG | 4731340 | 2.2348816 | 7.472308 | 1 |
| CACCA | 6659840 | 2.2262366 | 6.3755045 | 1 |
| TCCTC | 6707290 | 2.1928484 | 5.5618024 | 2 |
| CTCCA | 6532620 | 2.1595929 | 13.955834 | 1 |
| CCTTG | 5453350 | 2.1430352 | 5.097242 | 1 |
| TCTTG | 6058225 | 2.10599 | 5.2462015 | 7 |
| CTTGA | 5801175 | 2.0391536 | 5.846616 | 1 |
| CTCTG | 5070125 | 1.992437 | 9.57529 | 1 |
| CTTTG | 5715560 | 1.9868711 | 5.4083357 | 1 |
| TCCTT | 6753690 | 1.9532042 | 5.1958194 | 2 |
| CTCCT | 5960165 | 1.9485871 | 9.97223 | 1 |
| CTGCA | 4722460 | 1.8765374 | 5.776608 | 1 |
| CTCTT | 6363020 | 1.8402203 | 6.7694645 | 1 |
| TCCAA | 6073735 | 1.7960083 | 6.4839416 | 2 |
| GTTGG | 2949410 | 1.6745956 | 5.90694 | 1 |
| TTCAA | 6357245 | 1.6629012 | 5.268605 | 7 |
| TCCAT | 5671180 | 1.6584523 | 5.673758 | 2 |
| CCTCA | 5006330 | 1.6550226 | 5.6198306 | 1 |
| CTCTC | 5018550 | 1.64074 | 5.2624955 | 1 |
| CTCAG | 4022425 | 1.5983685 | 7.8289747 | 1 |
| TCCAC | 4655260 | 1.5389643 | 5.01006 | 2 |
| CTCAA | 5192405 | 1.5353984 | 6.201008 | 1 |
| TCCAG | 3834610 | 1.5237375 | 5.324707 | 2 |
| GGCAG | 2327600 | 1.5106403 | 5.3472443 | 1 |
| CTGGA | 3086875 | 1.47439 | 5.339284 | 1 |
| CCCAA | 4353400 | 1.4552449 | 6.7179856 | 1 |
| CTGGG | 2170335 | 1.3930175 | 5.4973125 | 1 |
| CTCAT | 4604040 | 1.346383 | 6.0202055 | 1 |
| CCCAT | 3918665 | 1.2954559 | 6.045693 | 1 |
| GCCAG | 2231870 | 1.2050838 | 5.0165 | 1 |
| CCCCA | 3217250 | 1.2023319 | 5.8906264 | 1 |
| CCCAG | 2673470 | 1.2009343 | 6.5238957 | 1 |
| GTCCA | 2894510 | 1.1501751 | 7.280235 | 1 |
| CTCCC | 2877410 | 1.063453 | 5.0682573 | 1 |
| GTCCT | 2692495 | 1.0580857 | 5.841039 | 1 |
| CTCGG | 1523775 | 0.8136661 | 5.1594486 | 1 |
| CTCCG | 1779990 | 0.7907487 | 5.97664 | 1 |

Produced by FastQC (version 0.10.1)
