# Supplementary material for: Analysis of Metabolites and Gene Expression Changes Relative to Apricot (Prunus armeniaca L.) Fruit Quality During Development and Ripening
Source: Front Plant Sci. 2020 Aug 19;11:1269. doi: 10.3389/fpls.2020.01269 (PMC7466674; doi:10.3389/fpls.2020.01269)
Supplement: Supplementary file 2 [file DataSheet_2.zip › FastQC_optimized/C_S3_L002_R2_001_reverse_paired_fastqc/fastqc_report.html]

C\_S3\_L002\_R2\_001\_reverse\_paired.fastq.gz FastQC Report


FastQC Report

vie 22 jun 2018  
C\_S3\_L002\_R2\_001\_reverse\_paired.fastq.gz

## Summary

- Basic Statistics
- Per base sequence quality
- Per sequence quality scores
- Per base sequence content
- Per base GC content
- Per sequence GC content
- Per base N content
- Sequence Length Distribution
- Sequence Duplication Levels
- Overrepresented sequences
- Kmer Content

## Basic Statistics

| Measure | Value |
| --- | --- |
| Filename | C\_S3\_L002\_R2\_001\_reverse\_paired.fastq.gz |
| File type | Conventional base calls |
| Encoding | Sanger / Illumina 1.9 |
| Total Sequences | 24195543 |
| Filtered Sequences | 0 |
| Sequence length | 81 |
| %GC | 45 |

## Per base sequence quality

## Per sequence quality scores

## Per base sequence content

## Per base GC content

## Per sequence GC content

## Per base N content

## Sequence Length Distribution

## Sequence Duplication Levels

## Overrepresented sequences

| Sequence | Count | Percentage | Possible Source |
| --- | --- | --- | --- |
| NNNNNNNNNNNNNNNNNNNNNNNNNNNNNNNNNNNNNNNNNNNNNNNNNN | 41997 | 0.17357328992368554 | No Hit |

## Kmer Content

| Sequence | Count | Obs/Exp Overall | Obs/Exp Max | Max Obs/Exp Position |
| --- | --- | --- | --- | --- |
| CTCTC | 5247590 | 3.9120657 | 5.712494 | 6 |
| TCTCT | 5876550 | 3.4210458 | 4.4201155 | 9 |
| GAAGA | 6701215 | 3.040815 | 3.2740068 | 75-77 |

Produced by FastQC (version 0.10.1)
