# Supplementary figures and images for: Analysis of Metabolites and Gene Expression Changes Relative to Apricot (Prunus armeniaca L.) Fruit Quality During Development and Ripening (part 2 of 3)
Source: Front Plant Sci. 2020 Aug 19;11:1269. doi: 10.3389/fpls.2020.01269 (PMC7466674; doi:10.3389/fpls.2020.01269)

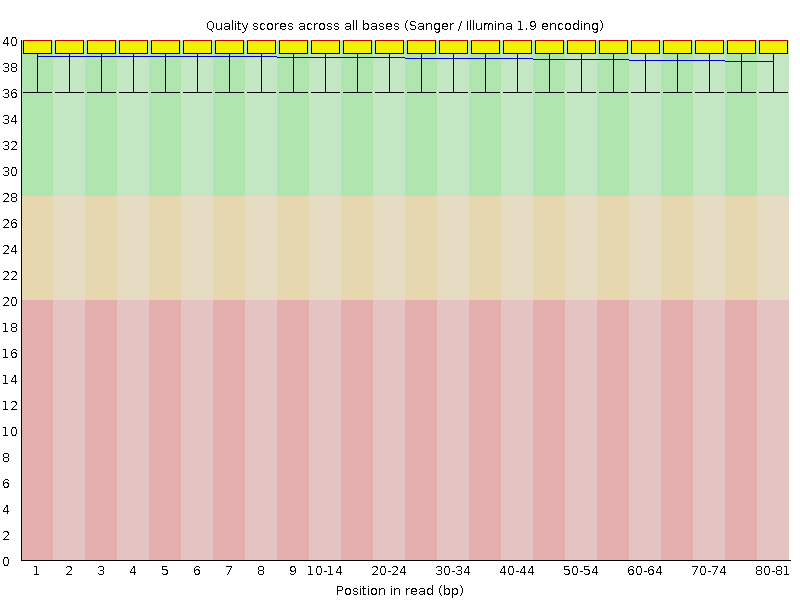

Supplement: Supplementary file 2 [file DataSheet_2.zip › FastQC_optimized/A_S1_L001_R1_001_forward_paired_fastqc/Images/per_base_quality.png]

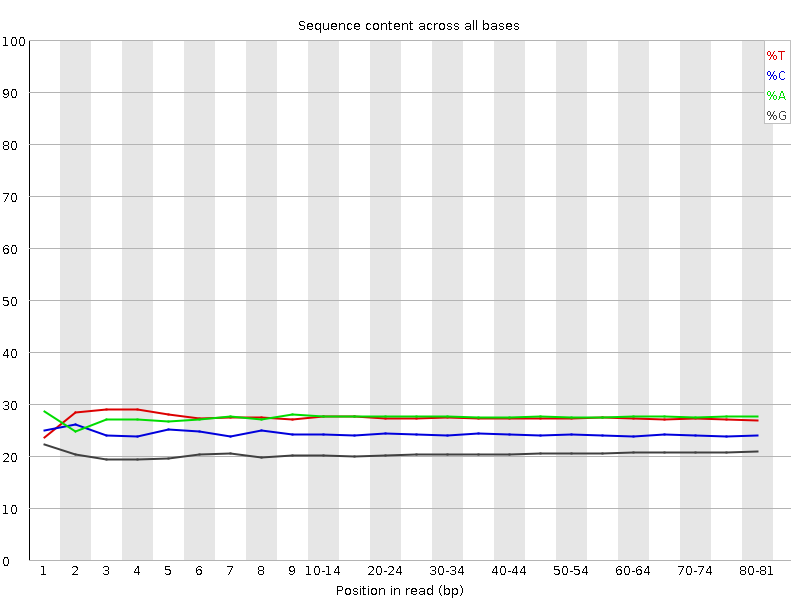

Supplement: Supplementary file 2 [file DataSheet_2.zip › FastQC_optimized/A_S1_L001_R1_001_forward_paired_fastqc/Images/per_base_sequence_content.png]

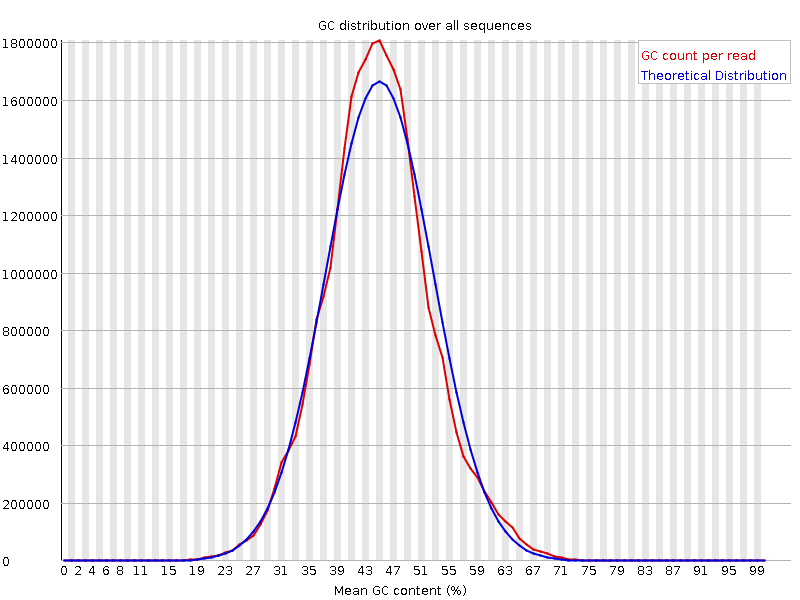

Supplement: Supplementary file 2 [file DataSheet_2.zip › FastQC_optimized/A_S1_L001_R1_001_forward_paired_fastqc/Images/per_sequence_gc_content.png]

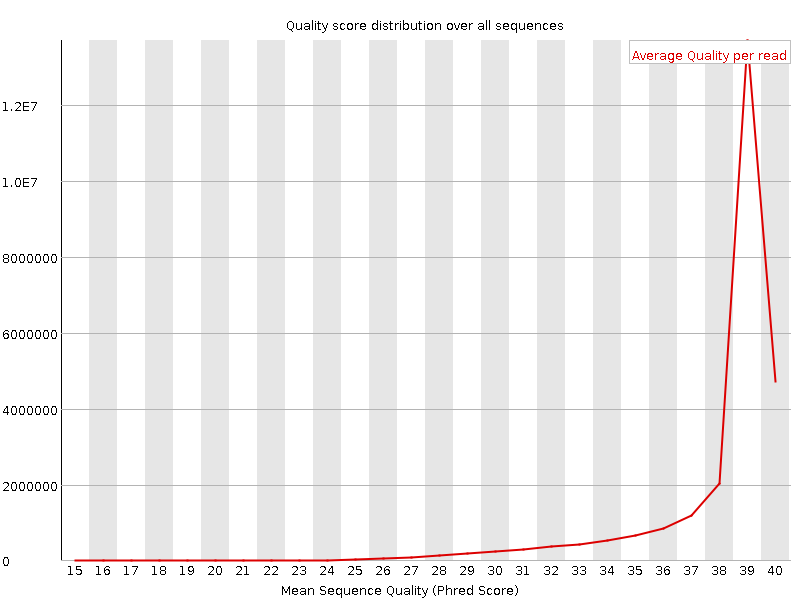

Supplement: Supplementary file 2 [file DataSheet_2.zip › FastQC_optimized/A_S1_L001_R1_001_forward_paired_fastqc/Images/per_sequence_quality.png]

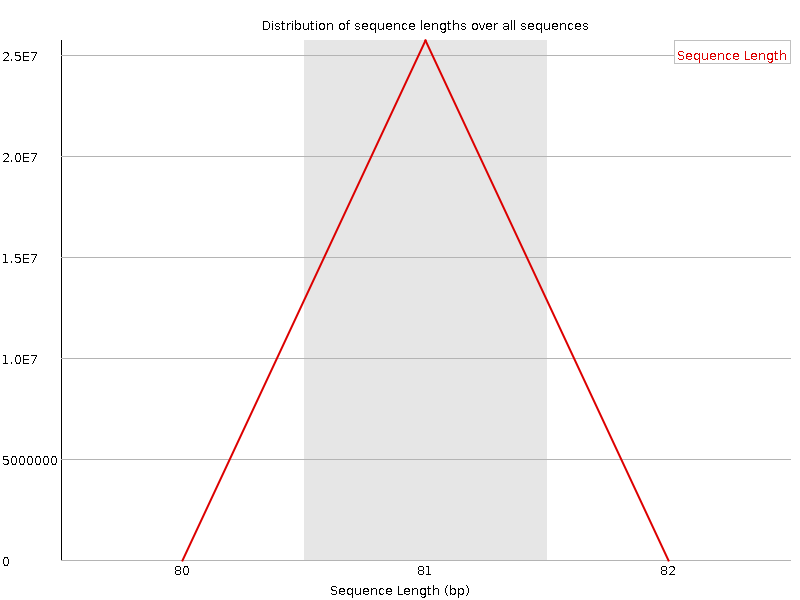

Supplement: Supplementary file 2 [file DataSheet_2.zip › FastQC_optimized/A_S1_L001_R1_001_forward_paired_fastqc/Images/sequence_length_distribution.png]

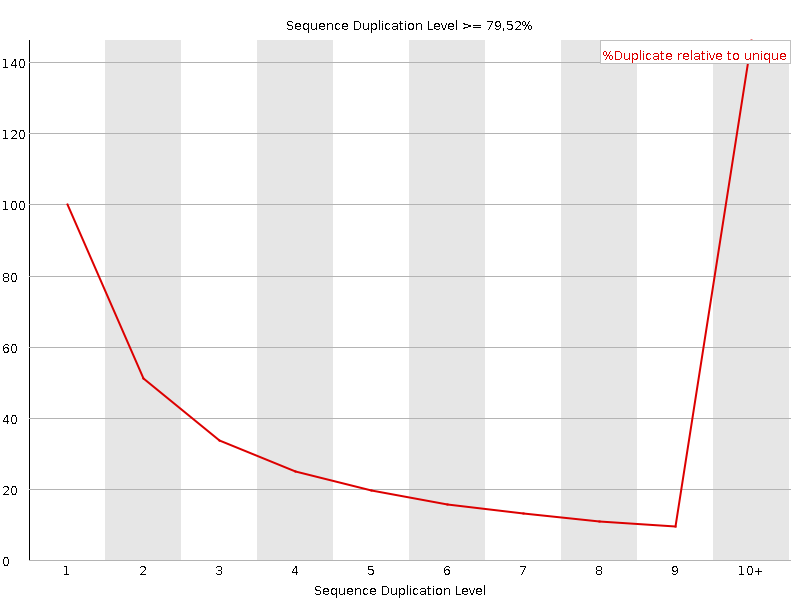

Supplement: Supplementary file 2 [file DataSheet_2.zip › FastQC_optimized/A_S1_L001_R2_001_reverse_paired_fastqc/Images/duplication_levels.png]

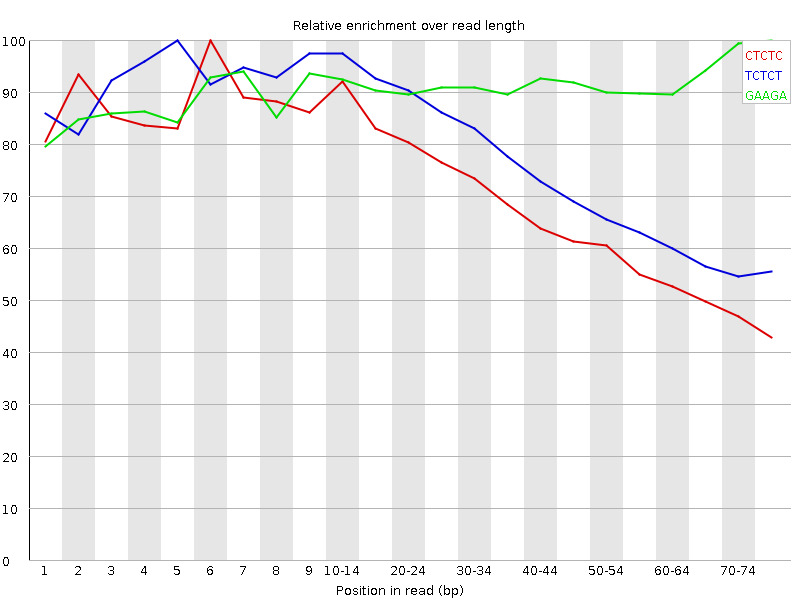

Supplement: Supplementary file 2 [file DataSheet_2.zip › FastQC_optimized/A_S1_L001_R2_001_reverse_paired_fastqc/Images/kmer_profiles.png]

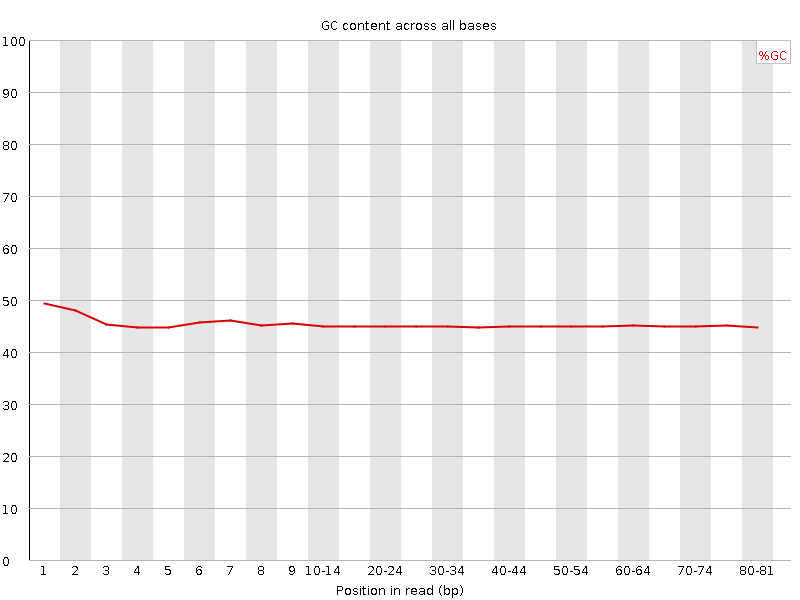

Supplement: Supplementary file 2 [file DataSheet_2.zip › FastQC_optimized/A_S1_L001_R2_001_reverse_paired_fastqc/Images/per_base_gc_content.png]

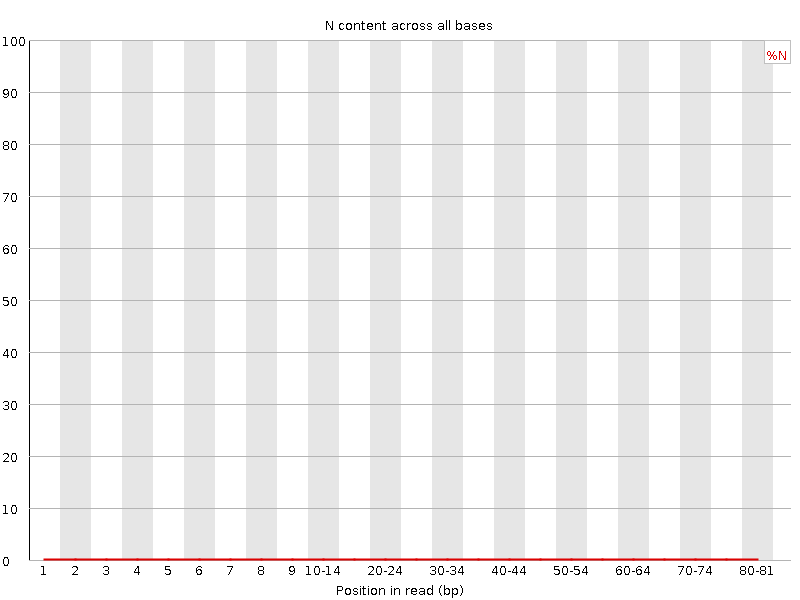

Supplement: Supplementary file 2 [file DataSheet_2.zip › FastQC_optimized/A_S1_L001_R2_001_reverse_paired_fastqc/Images/per_base_n_content.png]

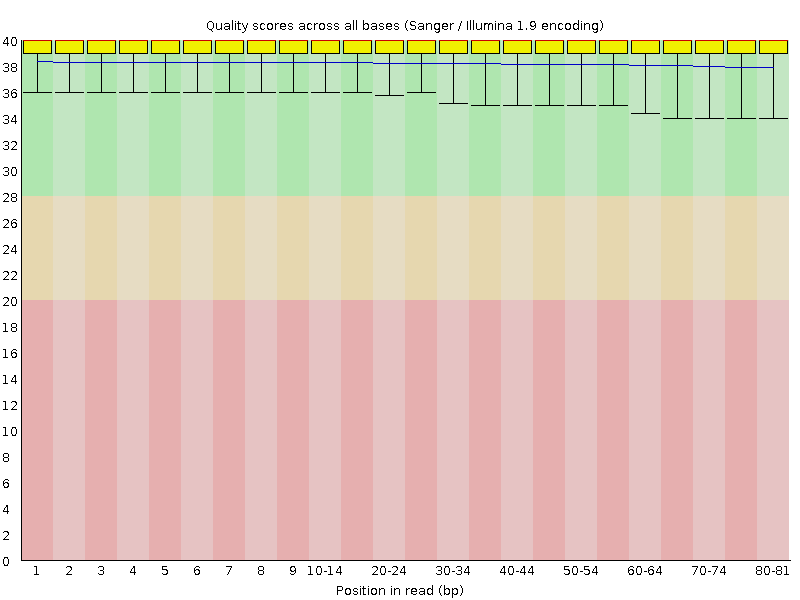

Supplement: Supplementary file 2 [file DataSheet_2.zip › FastQC_optimized/A_S1_L001_R2_001_reverse_paired_fastqc/Images/per_base_quality.png]

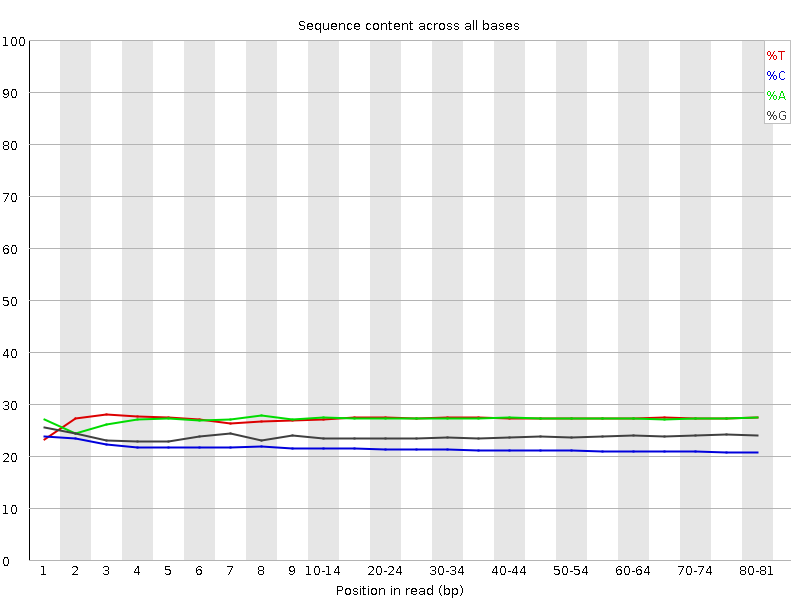

Supplement: Supplementary file 2 [file DataSheet_2.zip › FastQC_optimized/A_S1_L001_R2_001_reverse_paired_fastqc/Images/per_base_sequence_content.png]

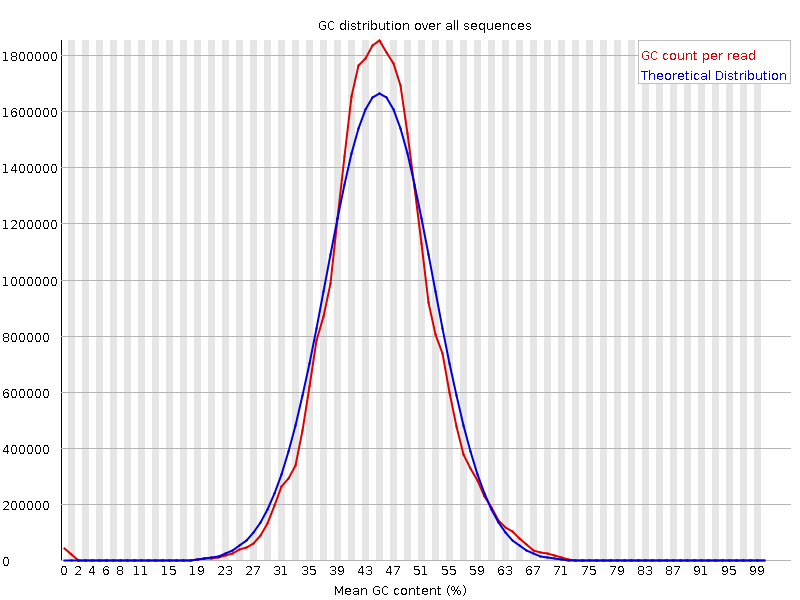

Supplement: Supplementary file 2 [file DataSheet_2.zip › FastQC_optimized/A_S1_L001_R2_001_reverse_paired_fastqc/Images/per_sequence_gc_content.png]

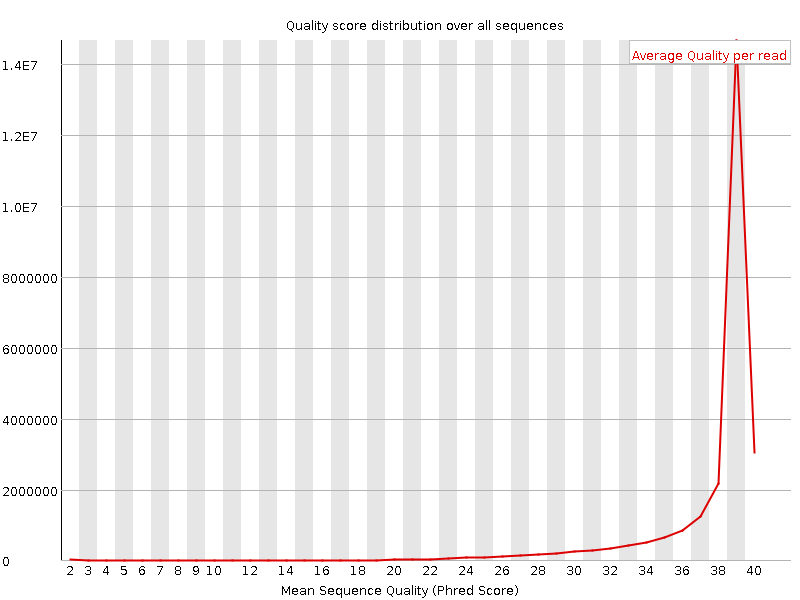

Supplement: Supplementary file 2 [file DataSheet_2.zip › FastQC_optimized/A_S1_L001_R2_001_reverse_paired_fastqc/Images/per_sequence_quality.png]

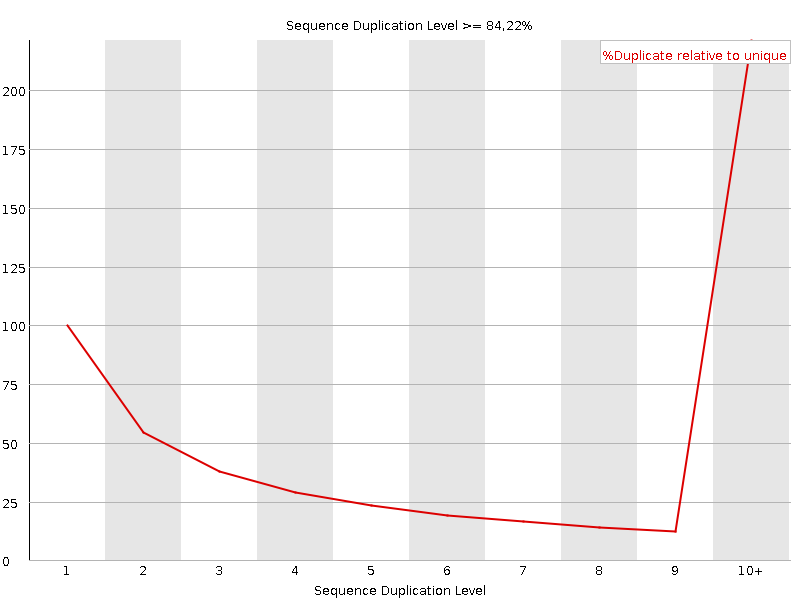

Supplement: Supplementary file 2 [file DataSheet_2.zip › FastQC_optimized/A_S1_L002_R1_001_forward_paired_fastqc/Images/duplication_levels.png]

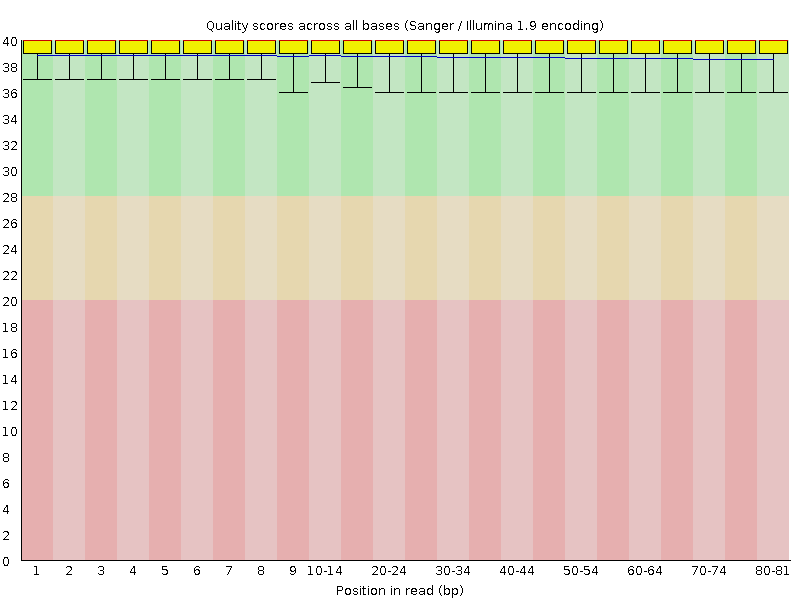

Supplement: Supplementary file 2 [file DataSheet_2.zip › FastQC_optimized/A_S1_L002_R1_001_forward_paired_fastqc/Images/per_base_quality.png]

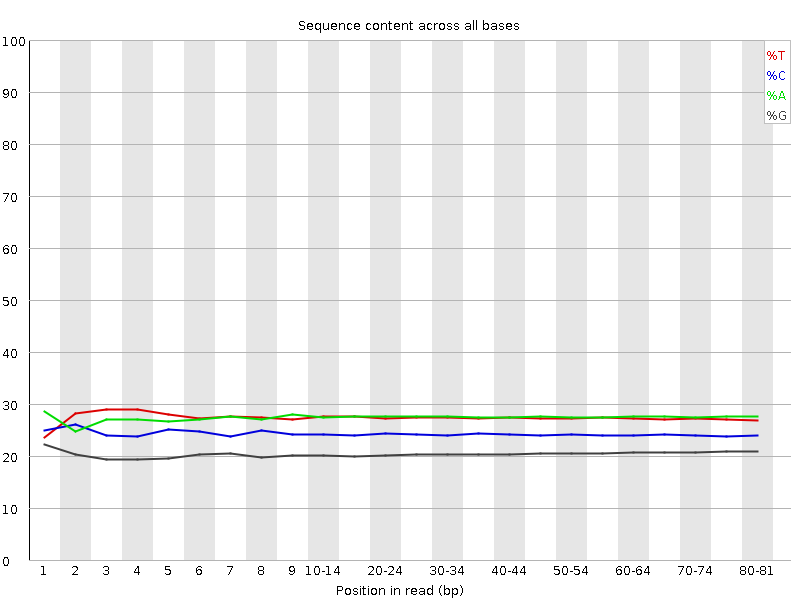

Supplement: Supplementary file 2 [file DataSheet_2.zip › FastQC_optimized/A_S1_L002_R1_001_forward_paired_fastqc/Images/per_base_sequence_content.png]

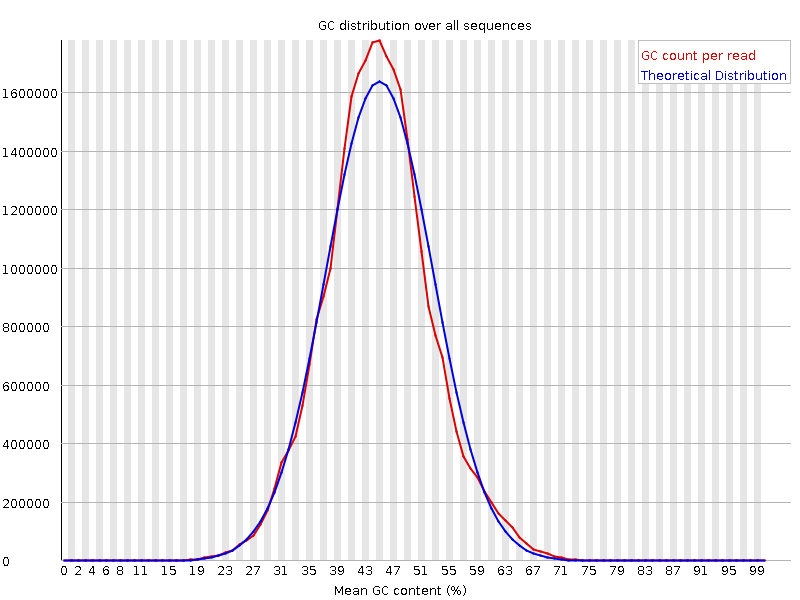

Supplement: Supplementary file 2 [file DataSheet_2.zip › FastQC_optimized/A_S1_L002_R1_001_forward_paired_fastqc/Images/per_sequence_gc_content.png]

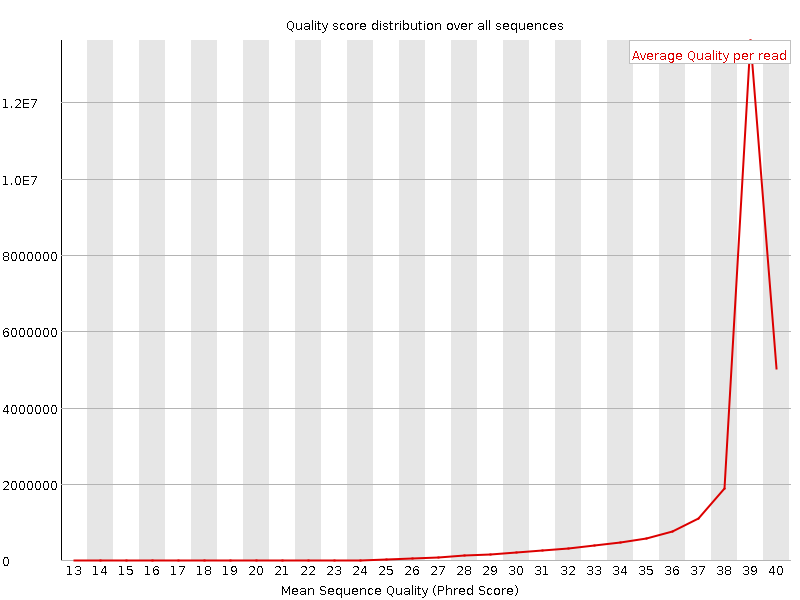

Supplement: Supplementary file 2 [file DataSheet_2.zip › FastQC_optimized/A_S1_L002_R1_001_forward_paired_fastqc/Images/per_sequence_quality.png]

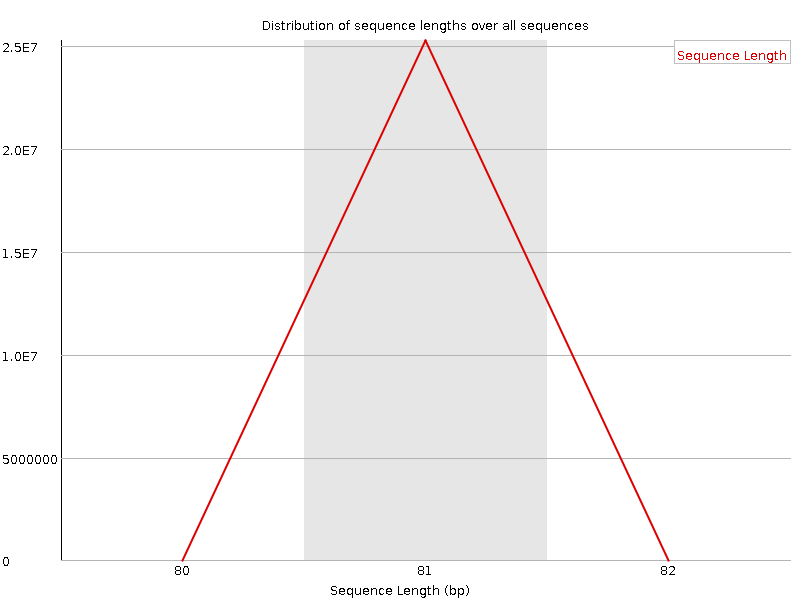

Supplement: Supplementary file 2 [file DataSheet_2.zip › FastQC_optimized/A_S1_L002_R1_001_forward_paired_fastqc/Images/sequence_length_distribution.png]

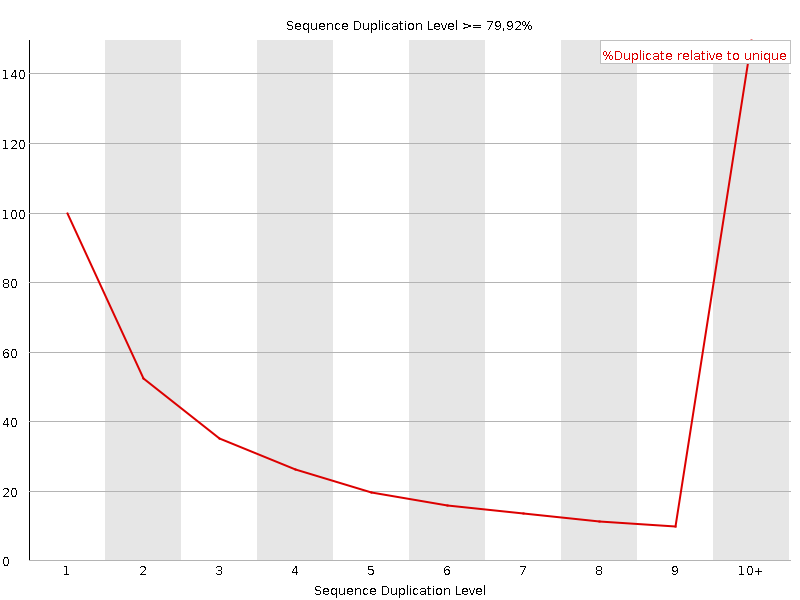

Supplement: Supplementary file 2 [file DataSheet_2.zip › FastQC_optimized/A_S1_L002_R2_001_reverse_paired_fastqc/Images/duplication_levels.png]

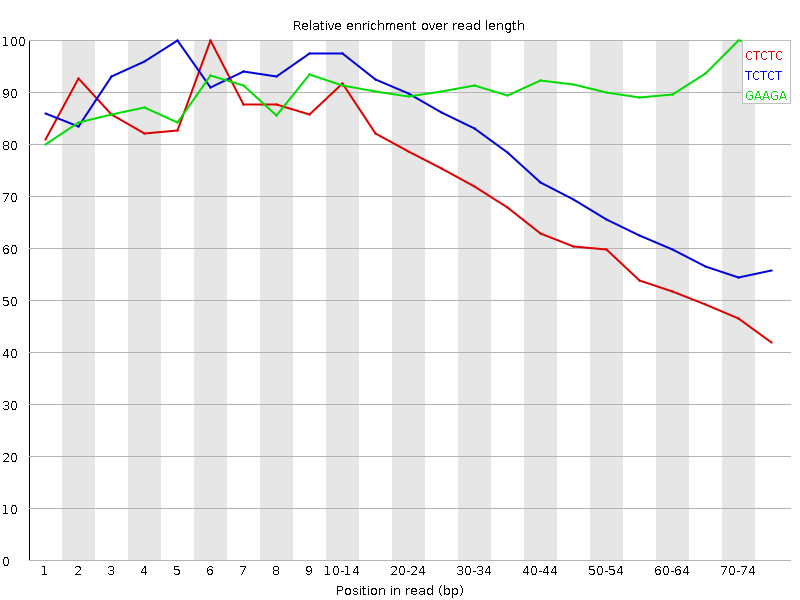

Supplement: Supplementary file 2 [file DataSheet_2.zip › FastQC_optimized/A_S1_L002_R2_001_reverse_paired_fastqc/Images/kmer_profiles.png]

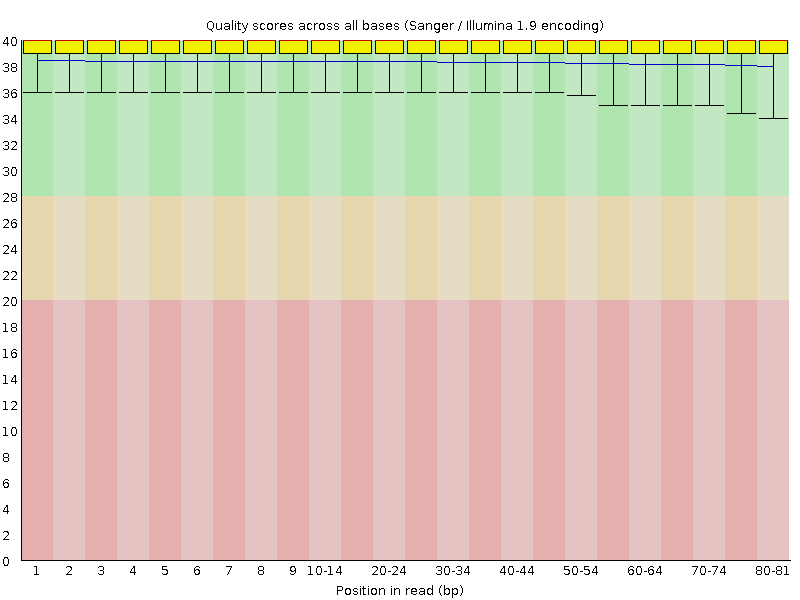

Supplement: Supplementary file 2 [file DataSheet_2.zip › FastQC_optimized/A_S1_L002_R2_001_reverse_paired_fastqc/Images/per_base_quality.png]

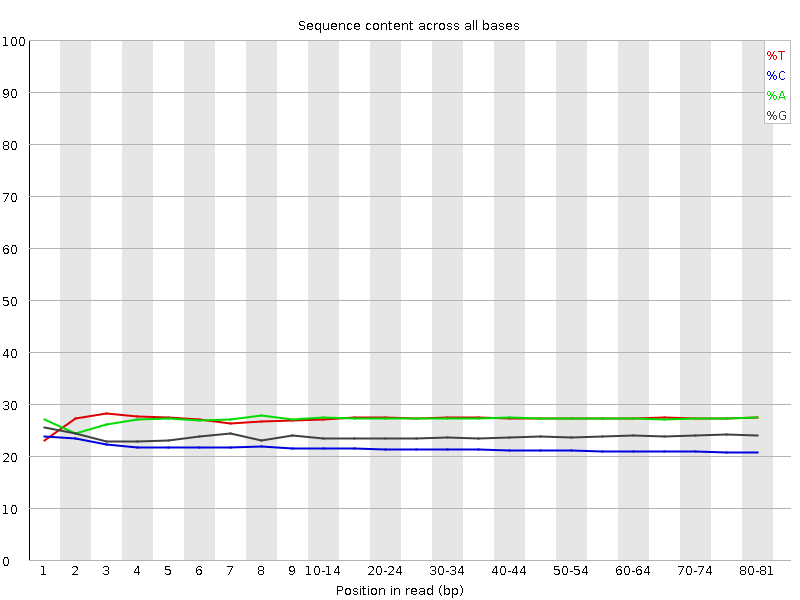

Supplement: Supplementary file 2 [file DataSheet_2.zip › FastQC_optimized/A_S1_L002_R2_001_reverse_paired_fastqc/Images/per_base_sequence_content.png]

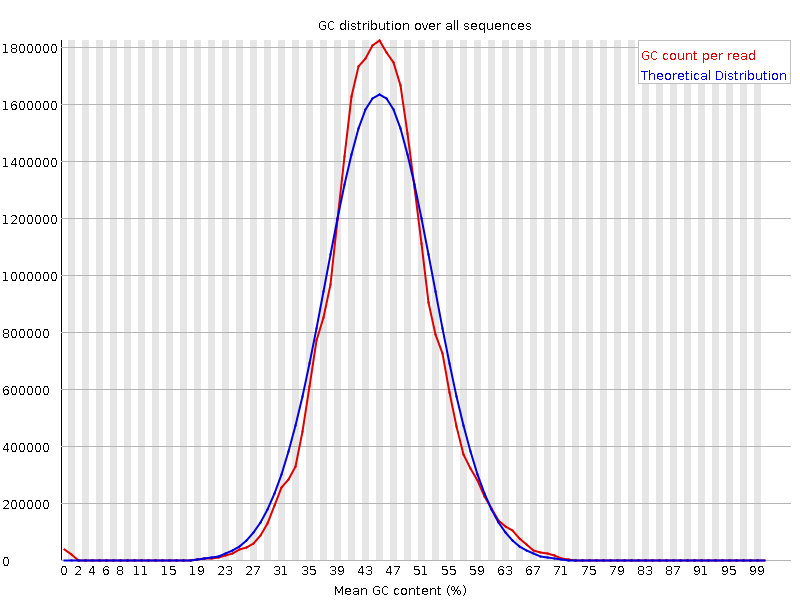

Supplement: Supplementary file 2 [file DataSheet_2.zip › FastQC_optimized/A_S1_L002_R2_001_reverse_paired_fastqc/Images/per_sequence_gc_content.png]

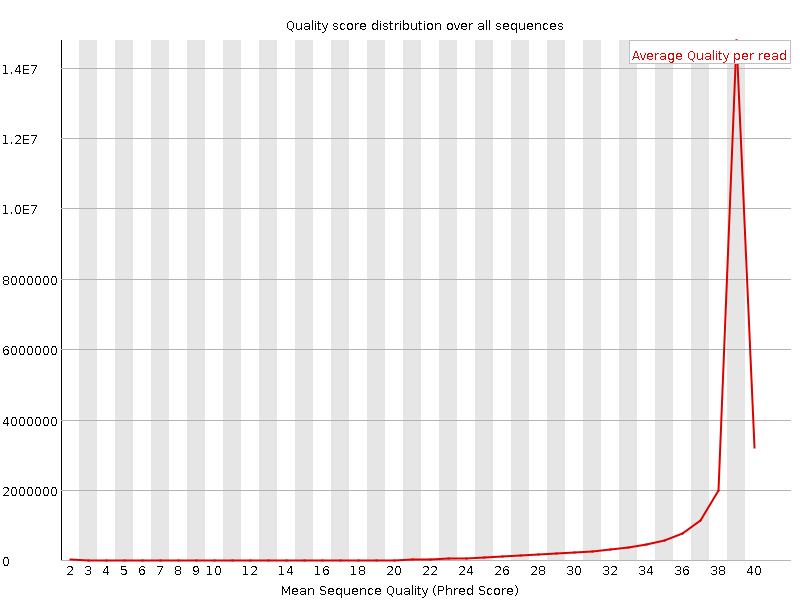

Supplement: Supplementary file 2 [file DataSheet_2.zip › FastQC_optimized/A_S1_L002_R2_001_reverse_paired_fastqc/Images/per_sequence_quality.png]

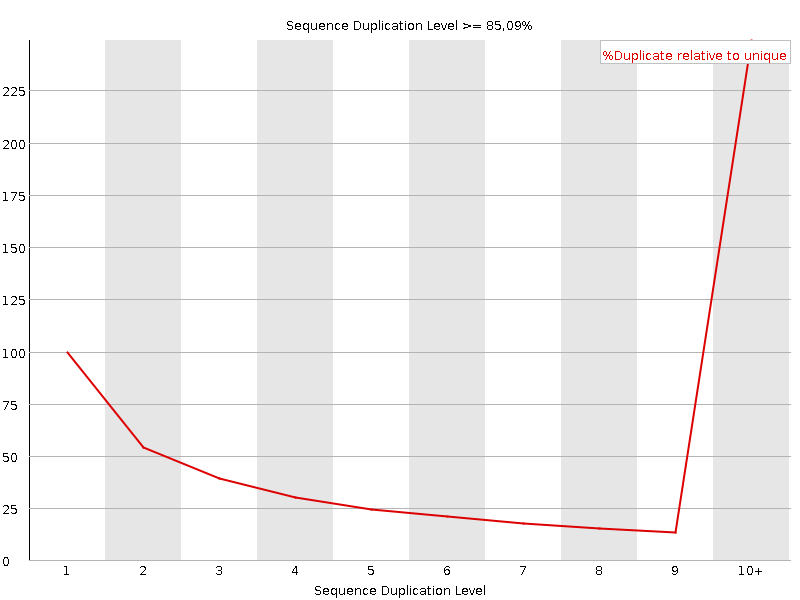

Supplement: Supplementary file 2 [file DataSheet_2.zip › FastQC_optimized/B_S2_L001_R1_001_forward_paired_fastqc/Images/duplication_levels.png]

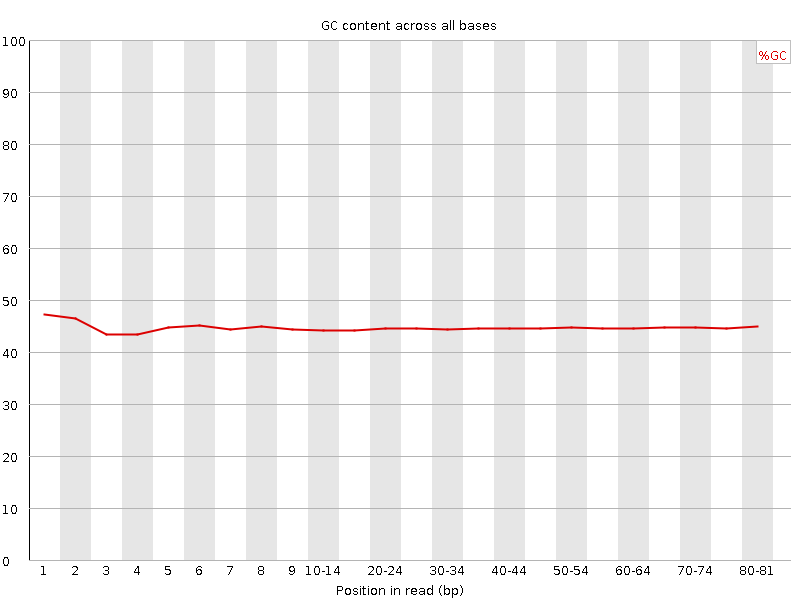

Supplement: Supplementary file 2 [file DataSheet_2.zip › FastQC_optimized/B_S2_L001_R1_001_forward_paired_fastqc/Images/per_base_gc_content.png]

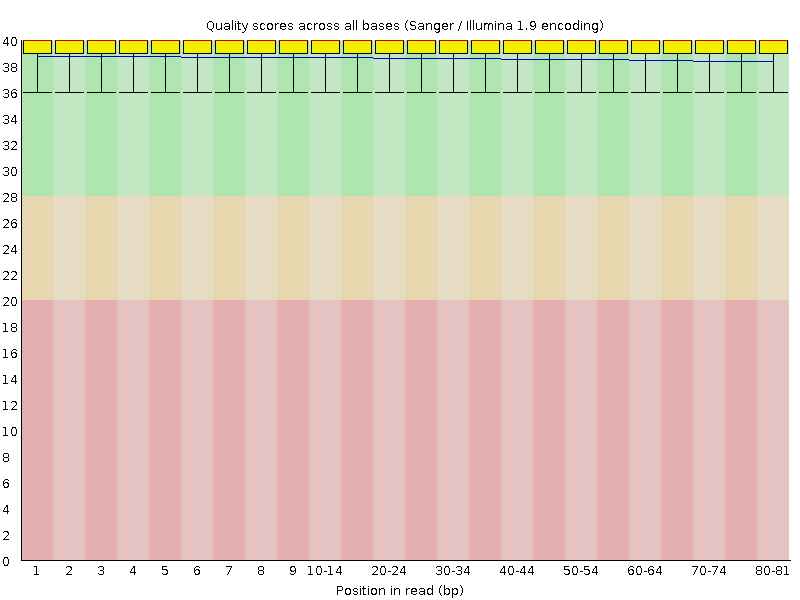

Supplement: Supplementary file 2 [file DataSheet_2.zip › FastQC_optimized/B_S2_L001_R1_001_forward_paired_fastqc/Images/per_base_quality.png]

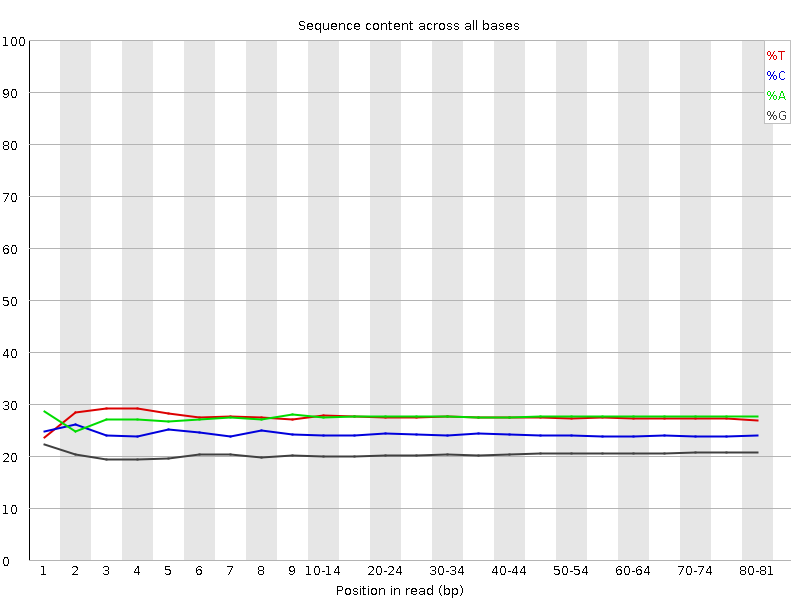

Supplement: Supplementary file 2 [file DataSheet_2.zip › FastQC_optimized/B_S2_L001_R1_001_forward_paired_fastqc/Images/per_base_sequence_content.png]

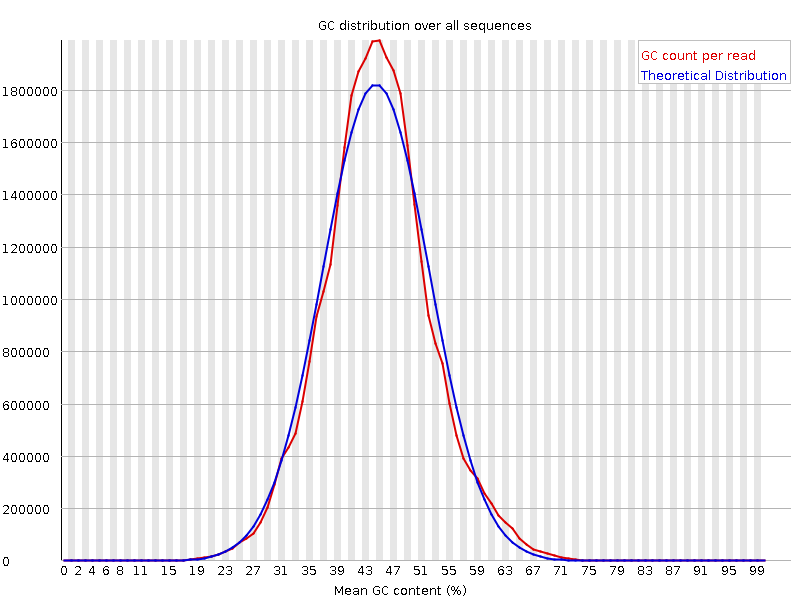

Supplement: Supplementary file 2 [file DataSheet_2.zip › FastQC_optimized/B_S2_L001_R1_001_forward_paired_fastqc/Images/per_sequence_gc_content.png]

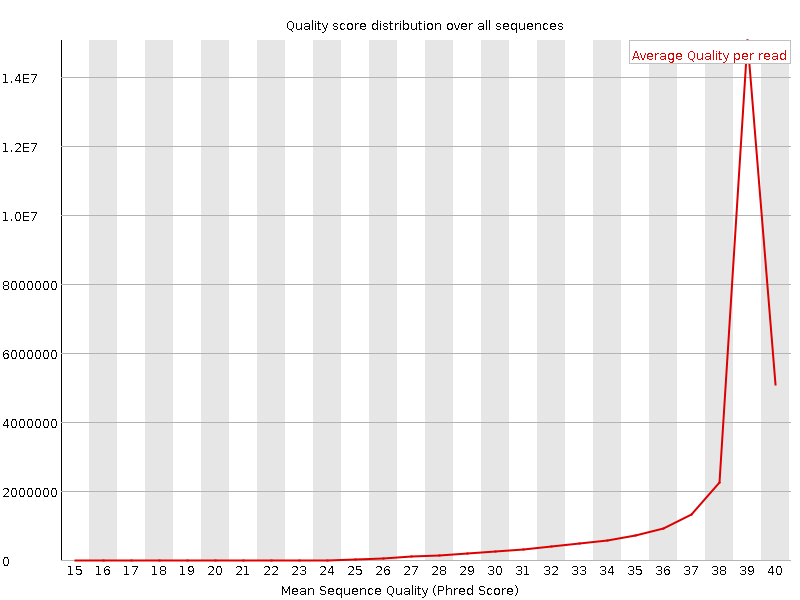

Supplement: Supplementary file 2 [file DataSheet_2.zip › FastQC_optimized/B_S2_L001_R1_001_forward_paired_fastqc/Images/per_sequence_quality.png]

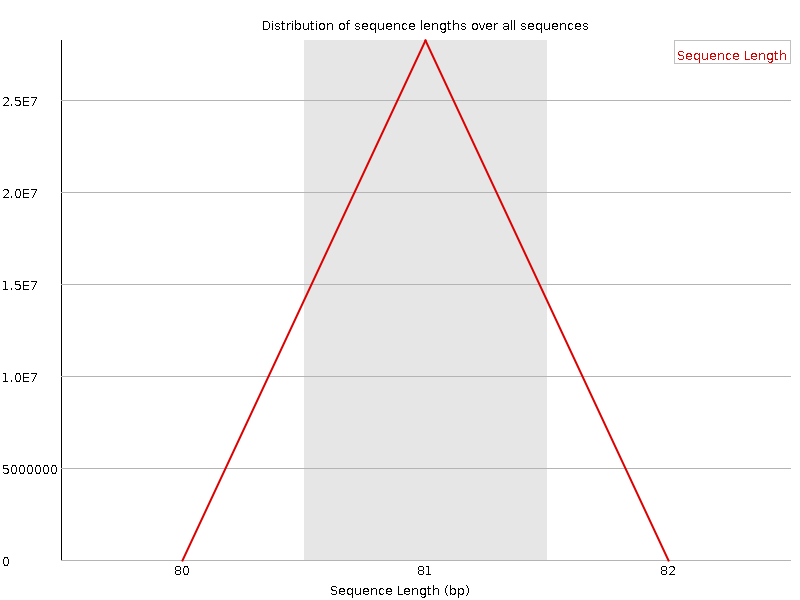

Supplement: Supplementary file 2 [file DataSheet_2.zip › FastQC_optimized/B_S2_L001_R1_001_forward_paired_fastqc/Images/sequence_length_distribution.png]

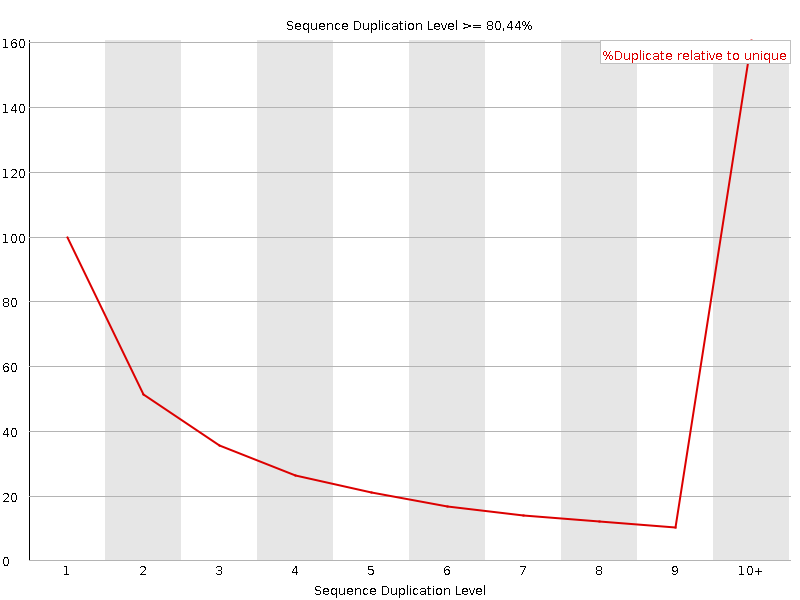

Supplement: Supplementary file 2 [file DataSheet_2.zip › FastQC_optimized/B_S2_L001_R2_001_reverse_paired_fastqc/Images/duplication_levels.png]

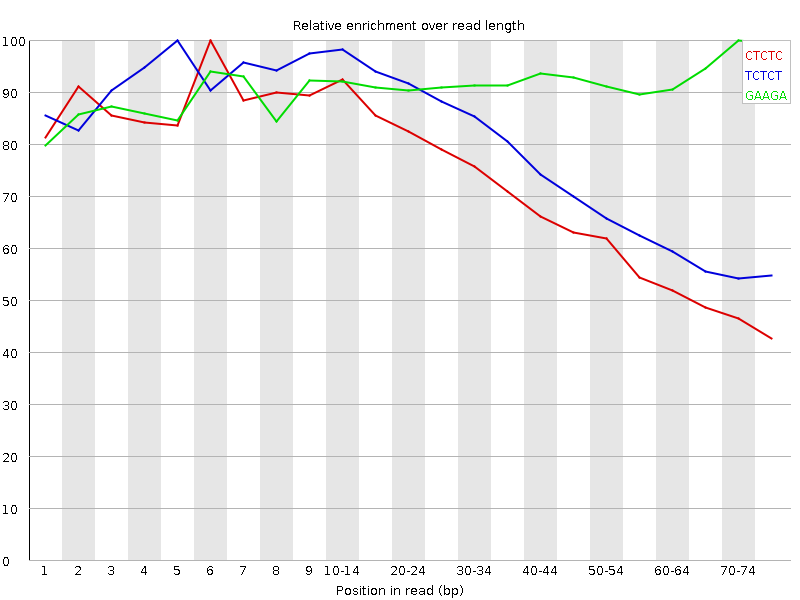

Supplement: Supplementary file 2 [file DataSheet_2.zip › FastQC_optimized/B_S2_L001_R2_001_reverse_paired_fastqc/Images/kmer_profiles.png]

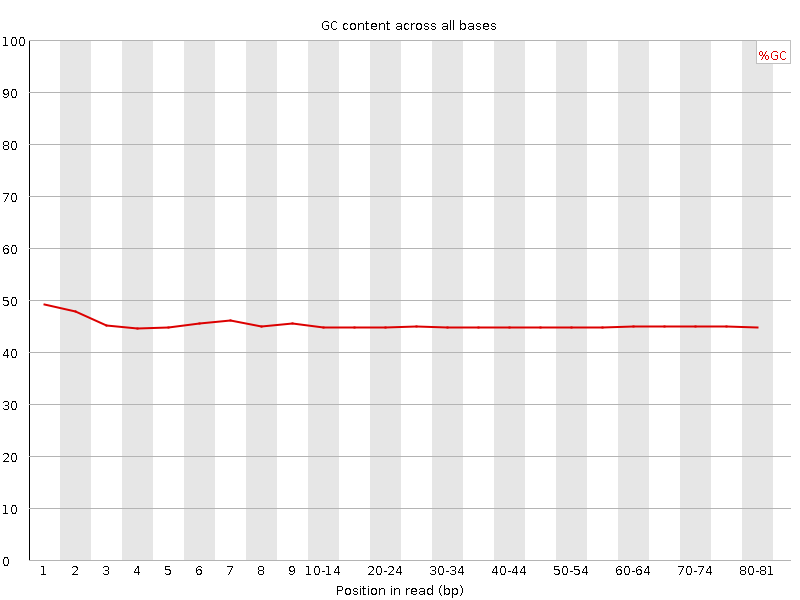

Supplement: Supplementary file 2 [file DataSheet_2.zip › FastQC_optimized/B_S2_L001_R2_001_reverse_paired_fastqc/Images/per_base_gc_content.png]

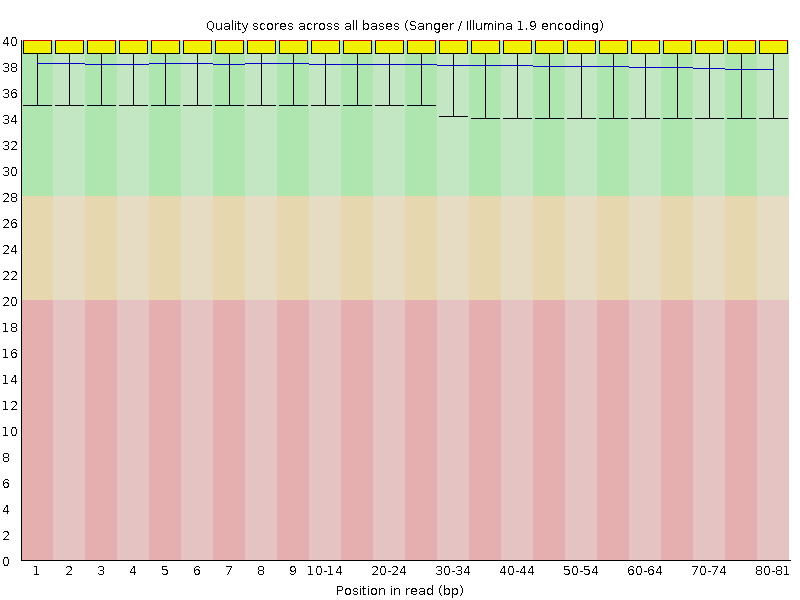

Supplement: Supplementary file 2 [file DataSheet_2.zip › FastQC_optimized/B_S2_L001_R2_001_reverse_paired_fastqc/Images/per_base_quality.png]

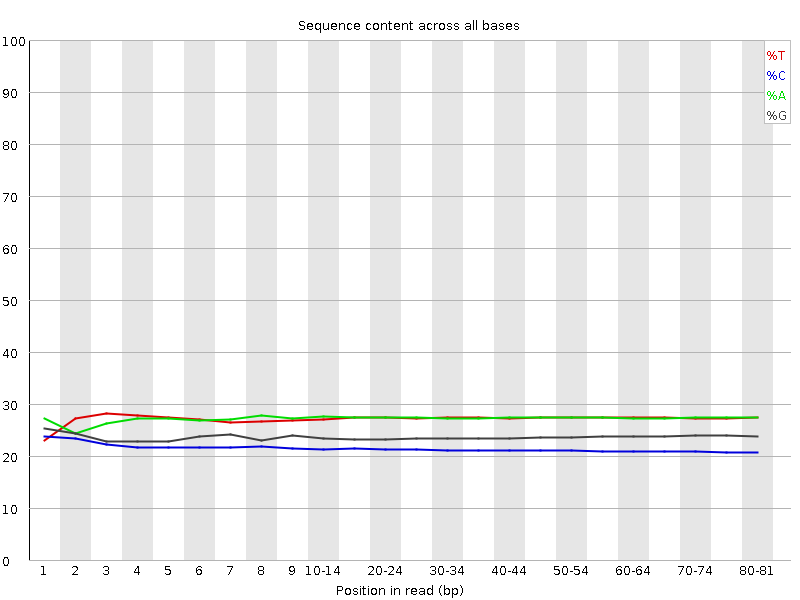

Supplement: Supplementary file 2 [file DataSheet_2.zip › FastQC_optimized/B_S2_L001_R2_001_reverse_paired_fastqc/Images/per_base_sequence_content.png]

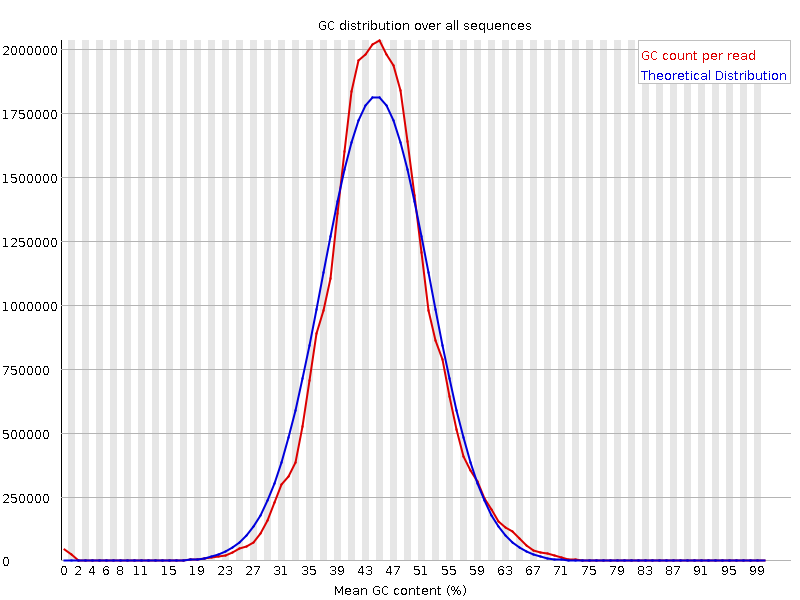

Supplement: Supplementary file 2 [file DataSheet_2.zip › FastQC_optimized/B_S2_L001_R2_001_reverse_paired_fastqc/Images/per_sequence_gc_content.png]

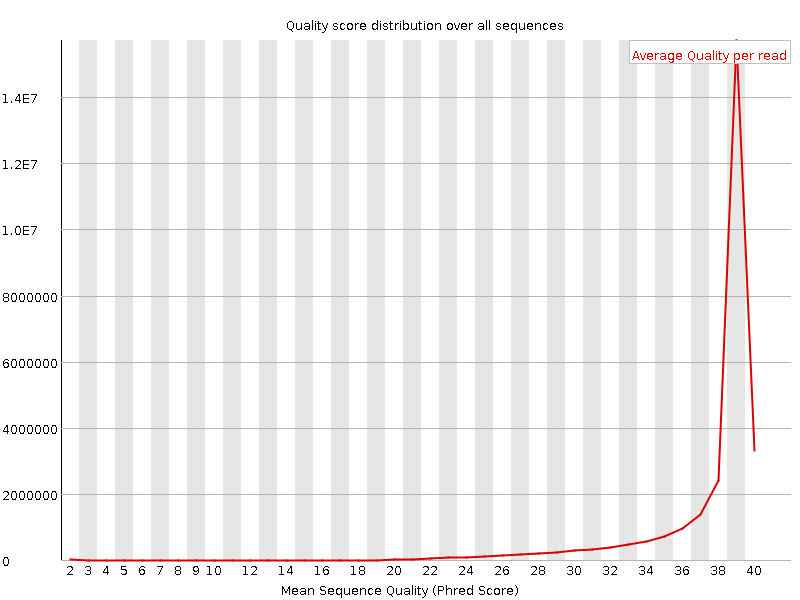

Supplement: Supplementary file 2 [file DataSheet_2.zip › FastQC_optimized/B_S2_L001_R2_001_reverse_paired_fastqc/Images/per_sequence_quality.png]

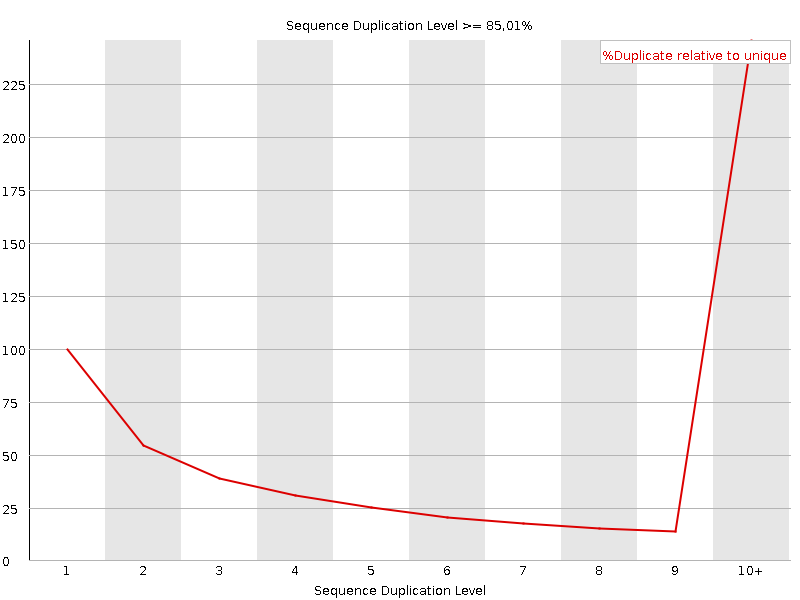

Supplement: Supplementary file 2 [file DataSheet_2.zip › FastQC_optimized/B_S2_L002_R1_001_forward_paired_fastqc/Images/duplication_levels.png]

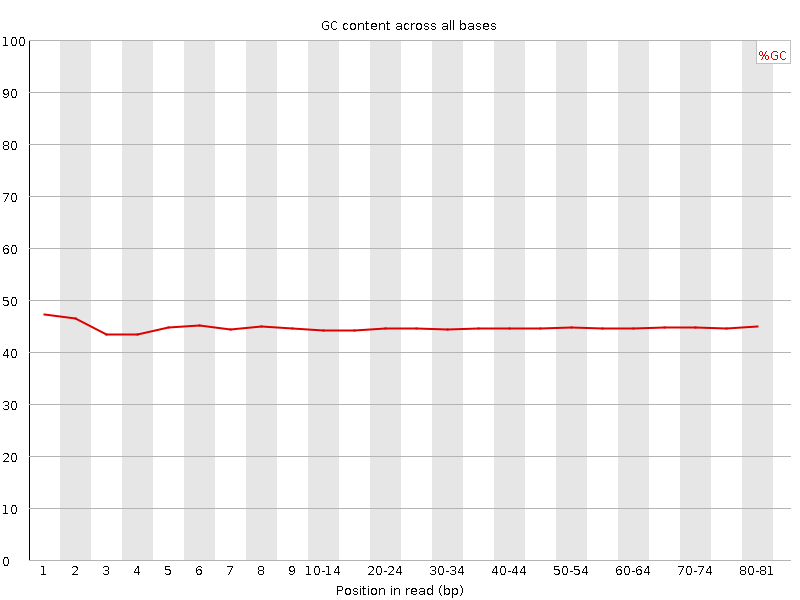

Supplement: Supplementary file 2 [file DataSheet_2.zip › FastQC_optimized/B_S2_L002_R1_001_forward_paired_fastqc/Images/per_base_gc_content.png]

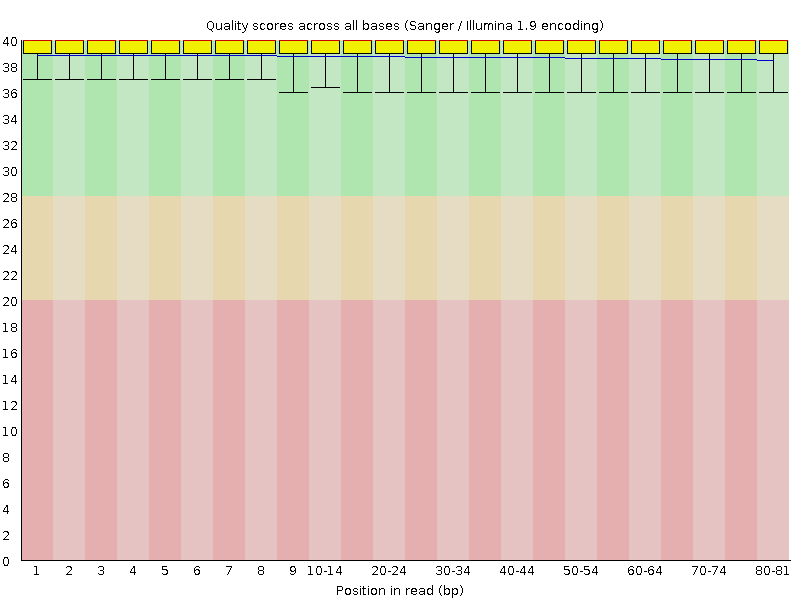

Supplement: Supplementary file 2 [file DataSheet_2.zip › FastQC_optimized/B_S2_L002_R1_001_forward_paired_fastqc/Images/per_base_quality.png]

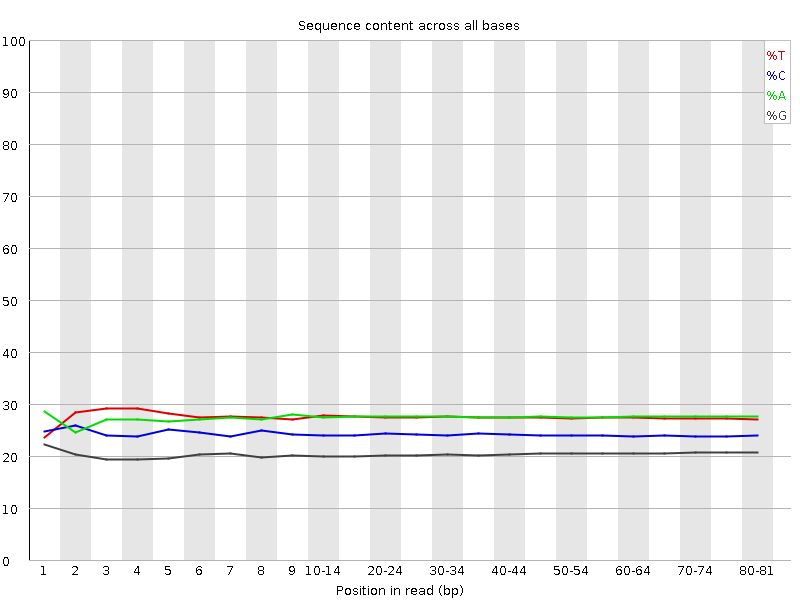

Supplement: Supplementary file 2 [file DataSheet_2.zip › FastQC_optimized/B_S2_L002_R1_001_forward_paired_fastqc/Images/per_base_sequence_content.png]

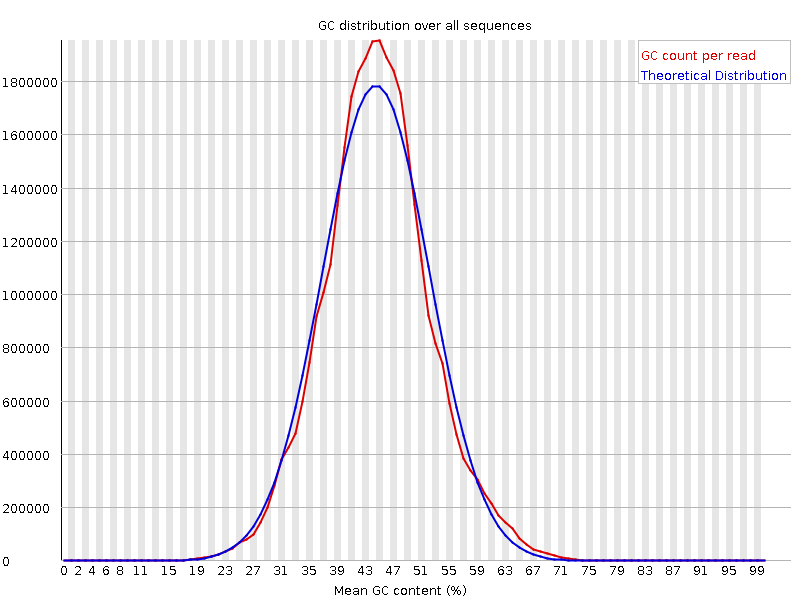

Supplement: Supplementary file 2 [file DataSheet_2.zip › FastQC_optimized/B_S2_L002_R1_001_forward_paired_fastqc/Images/per_sequence_gc_content.png]

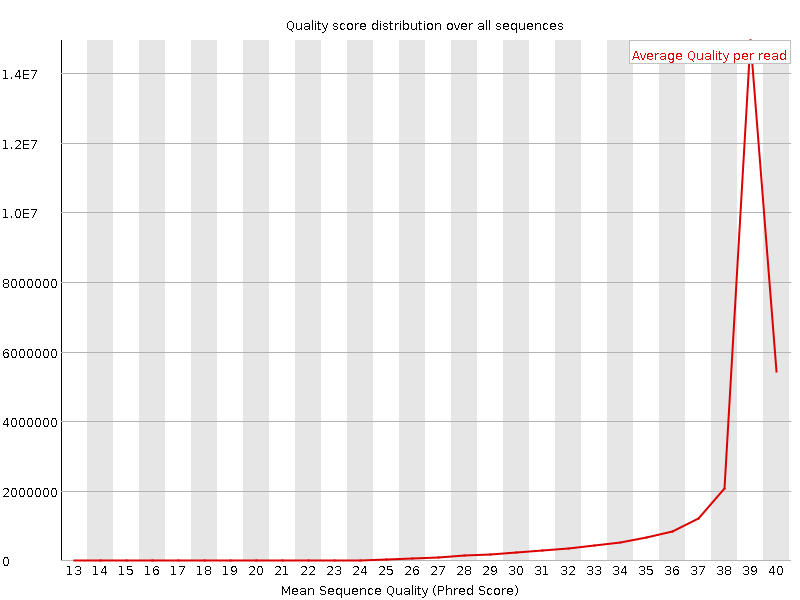

Supplement: Supplementary file 2 [file DataSheet_2.zip › FastQC_optimized/B_S2_L002_R1_001_forward_paired_fastqc/Images/per_sequence_quality.png]

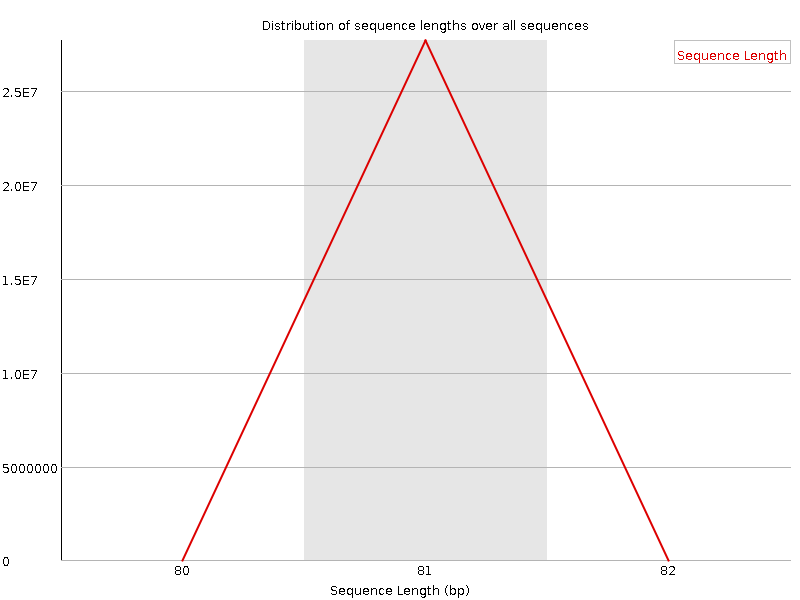

Supplement: Supplementary file 2 [file DataSheet_2.zip › FastQC_optimized/B_S2_L002_R1_001_forward_paired_fastqc/Images/sequence_length_distribution.png]

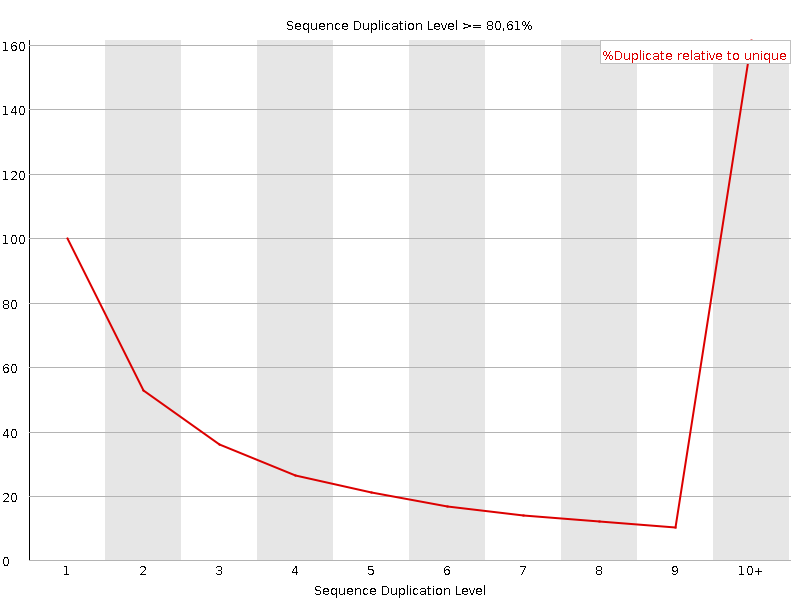

Supplement: Supplementary file 2 [file DataSheet_2.zip › FastQC_optimized/B_S2_L002_R2_001_reverse_paired_fastqc/Images/duplication_levels.png]

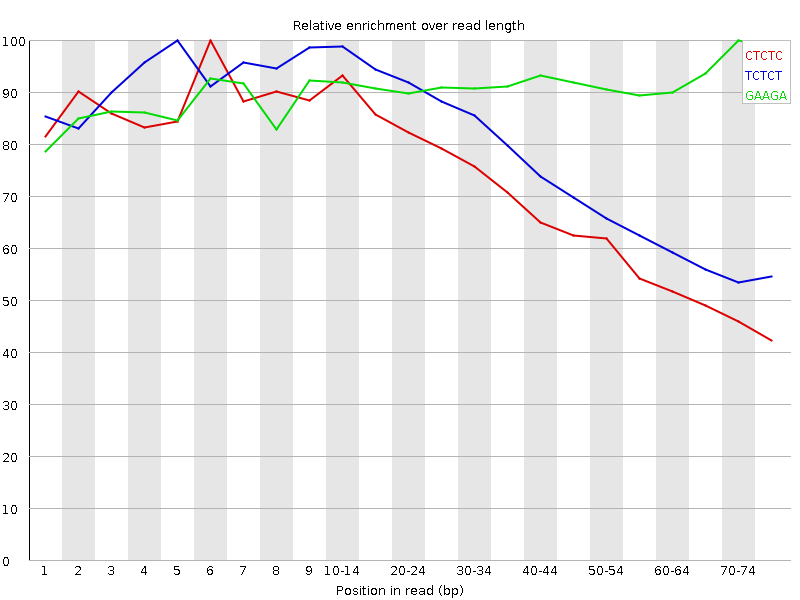

Supplement: Supplementary file 2 [file DataSheet_2.zip › FastQC_optimized/B_S2_L002_R2_001_reverse_paired_fastqc/Images/kmer_profiles.png]

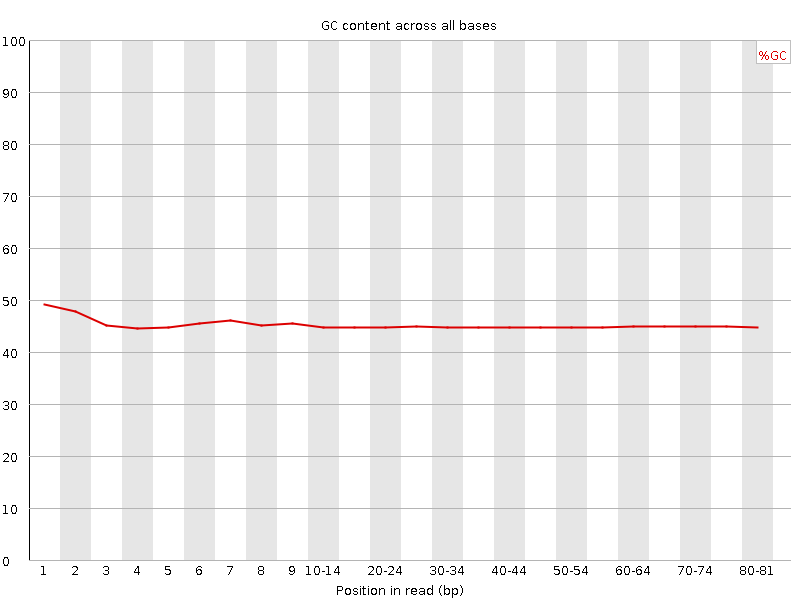

Supplement: Supplementary file 2 [file DataSheet_2.zip › FastQC_optimized/B_S2_L002_R2_001_reverse_paired_fastqc/Images/per_base_gc_content.png]

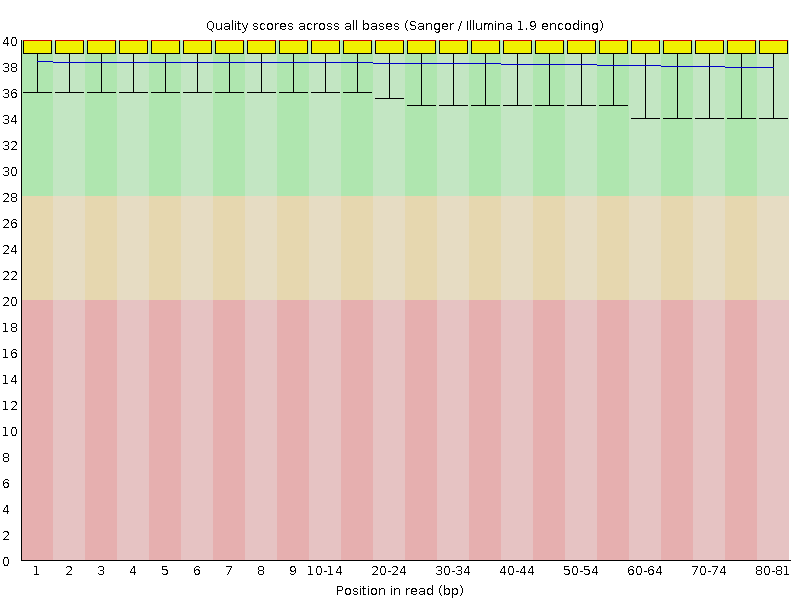

Supplement: Supplementary file 2 [file DataSheet_2.zip › FastQC_optimized/B_S2_L002_R2_001_reverse_paired_fastqc/Images/per_base_quality.png]

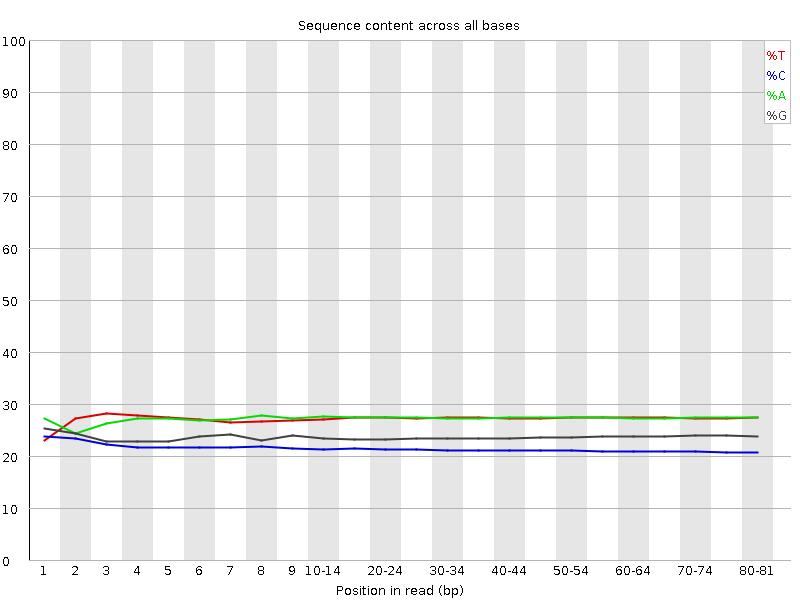

Supplement: Supplementary file 2 [file DataSheet_2.zip › FastQC_optimized/B_S2_L002_R2_001_reverse_paired_fastqc/Images/per_base_sequence_content.png]

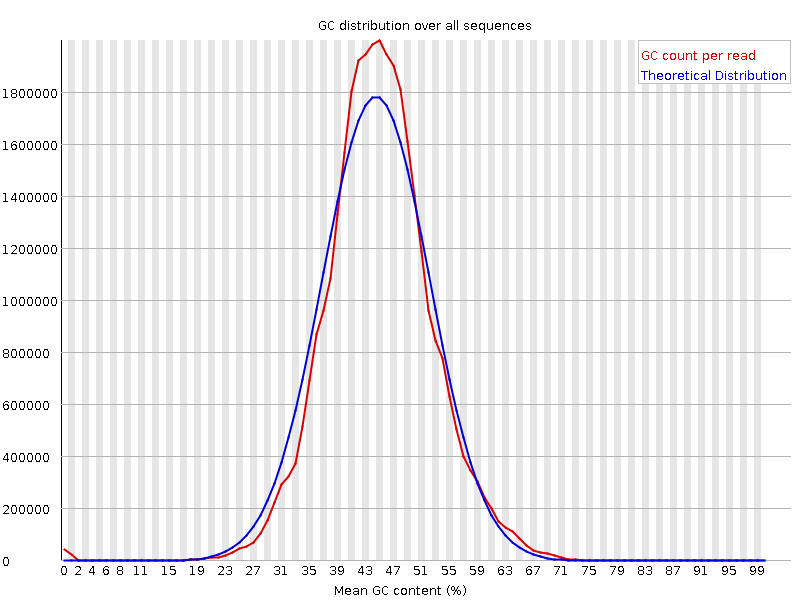

Supplement: Supplementary file 2 [file DataSheet_2.zip › FastQC_optimized/B_S2_L002_R2_001_reverse_paired_fastqc/Images/per_sequence_gc_content.png]

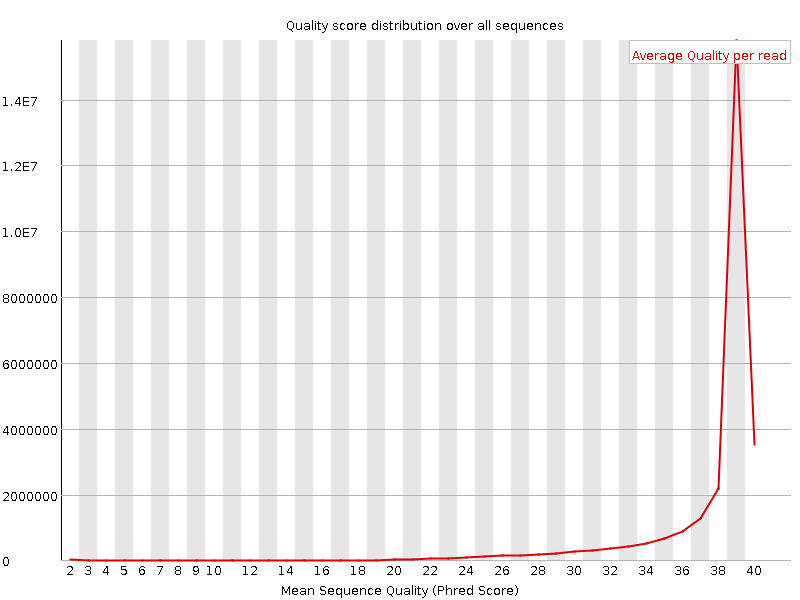

Supplement: Supplementary file 2 [file DataSheet_2.zip › FastQC_optimized/B_S2_L002_R2_001_reverse_paired_fastqc/Images/per_sequence_quality.png]

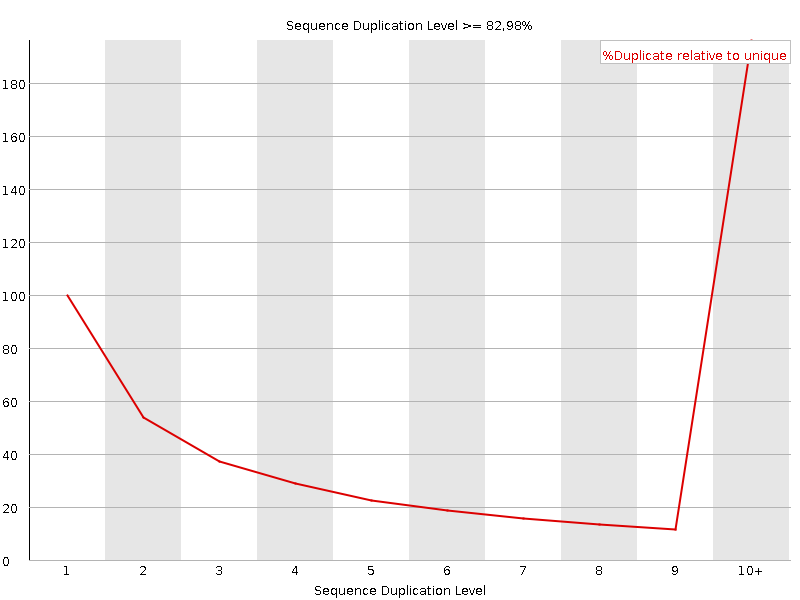

Supplement: Supplementary file 2 [file DataSheet_2.zip › FastQC_optimized/C_S3_L001_R1_001_forward_paired_fastqc/Images/duplication_levels.png]

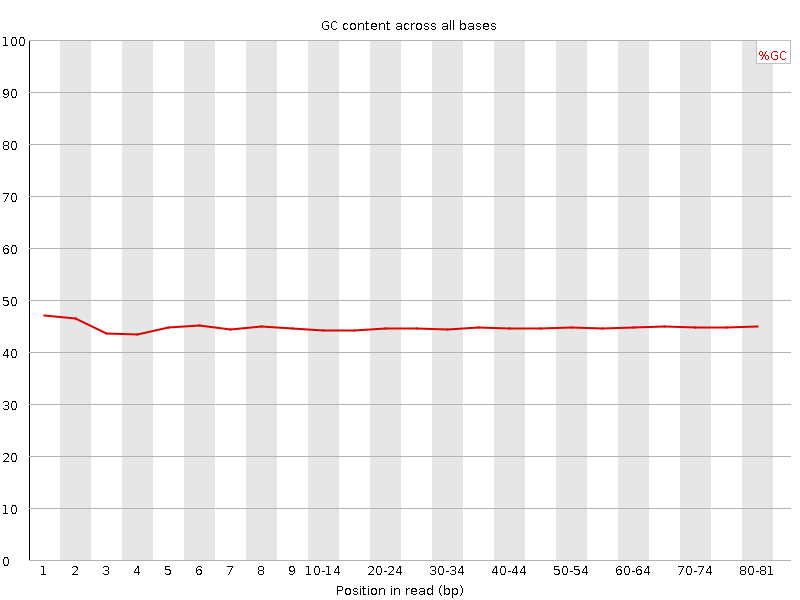

Supplement: Supplementary file 2 [file DataSheet_2.zip › FastQC_optimized/C_S3_L001_R1_001_forward_paired_fastqc/Images/per_base_gc_content.png]

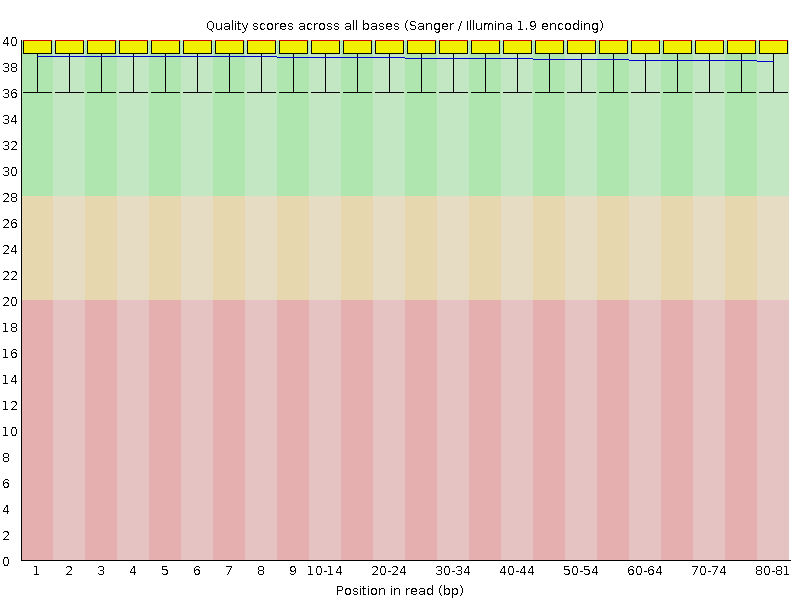

Supplement: Supplementary file 2 [file DataSheet_2.zip › FastQC_optimized/C_S3_L001_R1_001_forward_paired_fastqc/Images/per_base_quality.png]

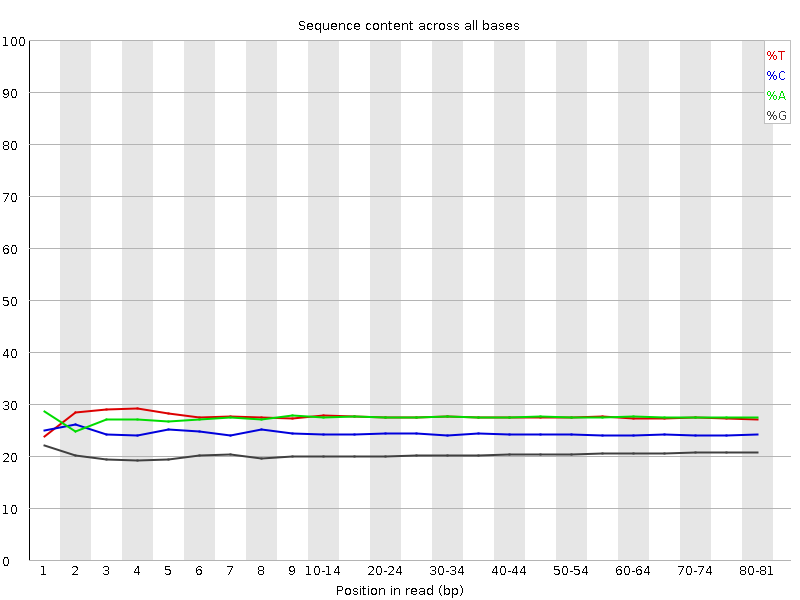

Supplement: Supplementary file 2 [file DataSheet_2.zip › FastQC_optimized/C_S3_L001_R1_001_forward_paired_fastqc/Images/per_base_sequence_content.png]

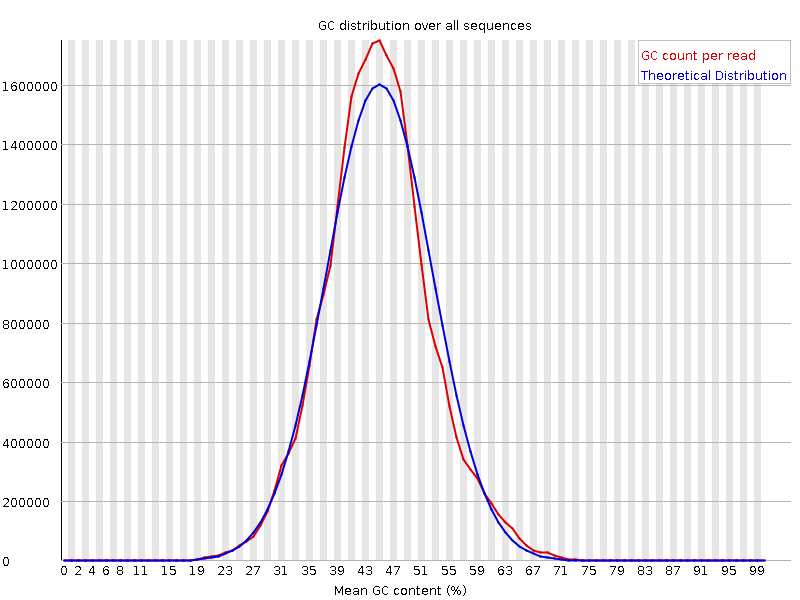

Supplement: Supplementary file 2 [file DataSheet_2.zip › FastQC_optimized/C_S3_L001_R1_001_forward_paired_fastqc/Images/per_sequence_gc_content.png]

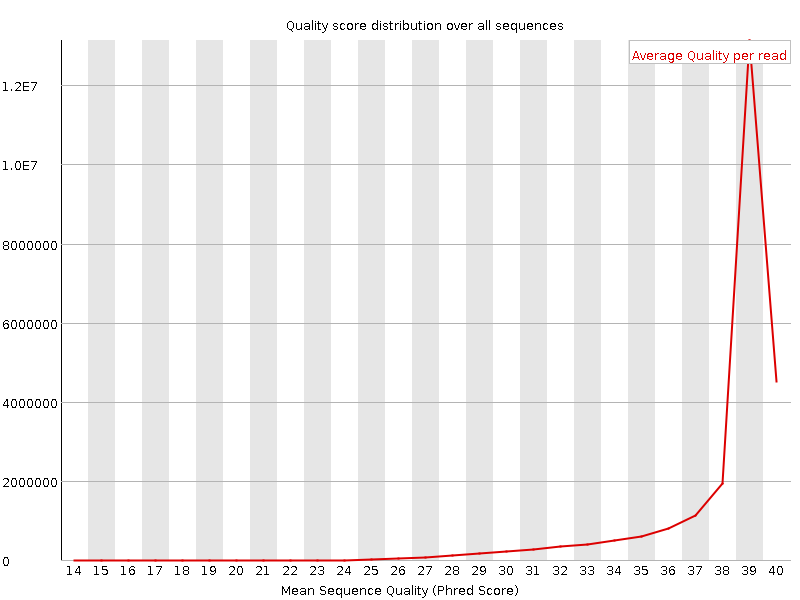

Supplement: Supplementary file 2 [file DataSheet_2.zip › FastQC_optimized/C_S3_L001_R1_001_forward_paired_fastqc/Images/per_sequence_quality.png]

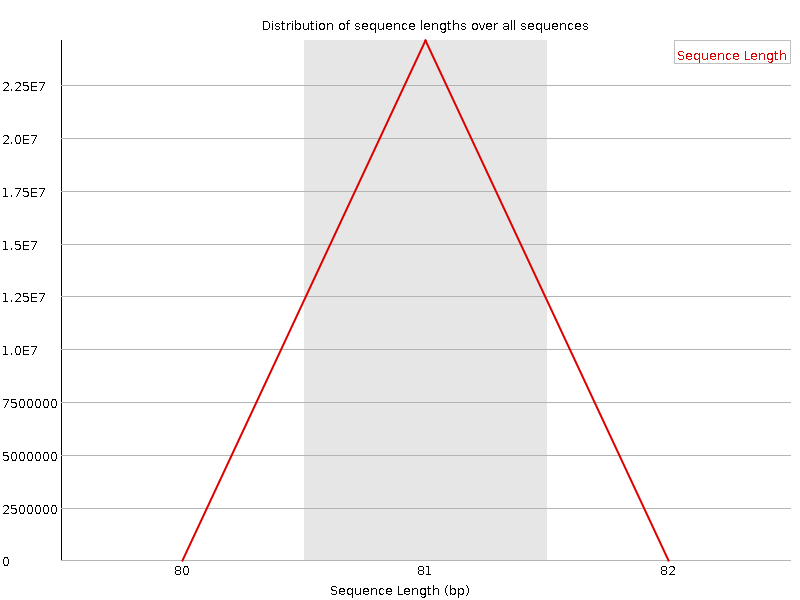

Supplement: Supplementary file 2 [file DataSheet_2.zip › FastQC_optimized/C_S3_L001_R1_001_forward_paired_fastqc/Images/sequence_length_distribution.png]

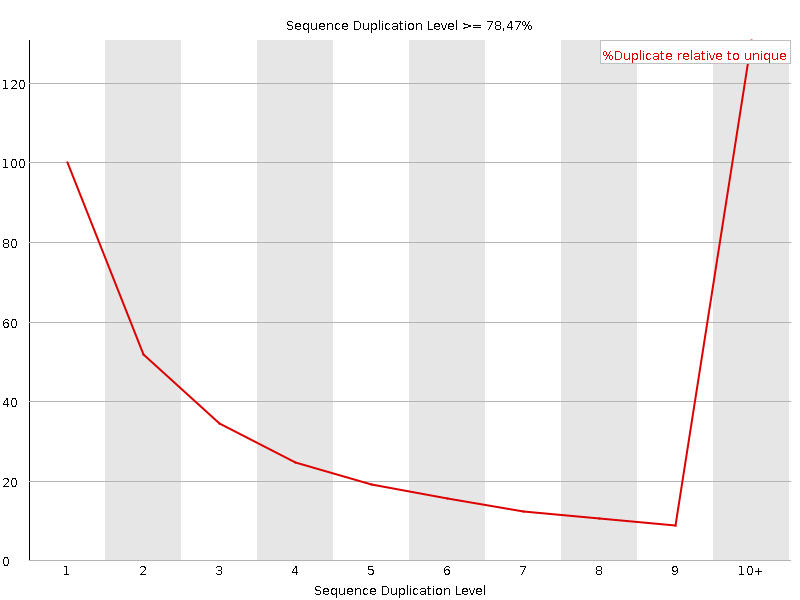

Supplement: Supplementary file 2 [file DataSheet_2.zip › FastQC_optimized/C_S3_L001_R2_001_reverse_paired_fastqc/Images/duplication_levels.png]

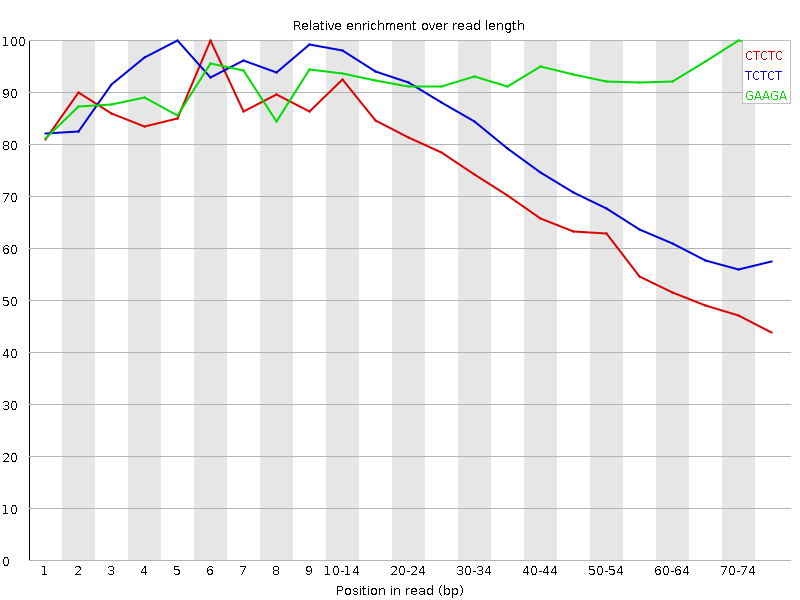

Supplement: Supplementary file 2 [file DataSheet_2.zip › FastQC_optimized/C_S3_L001_R2_001_reverse_paired_fastqc/Images/kmer_profiles.png]

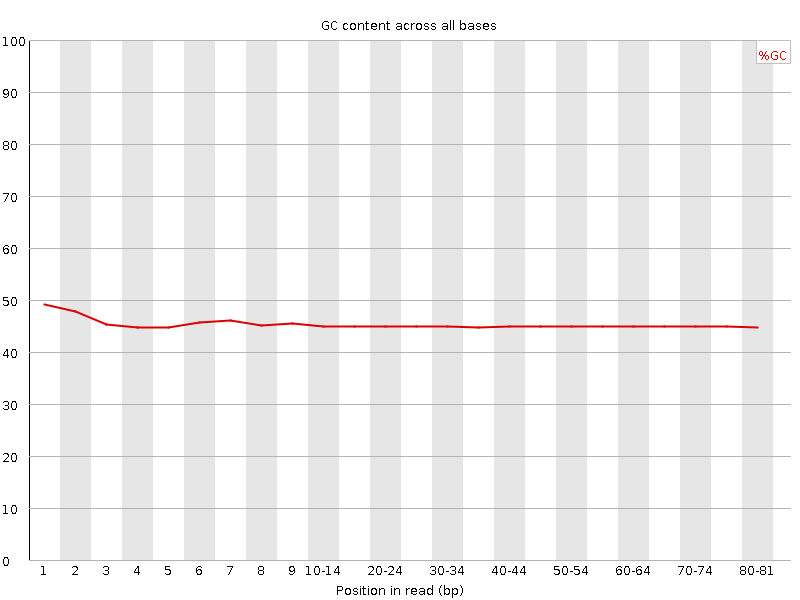

Supplement: Supplementary file 2 [file DataSheet_2.zip › FastQC_optimized/C_S3_L001_R2_001_reverse_paired_fastqc/Images/per_base_gc_content.png]

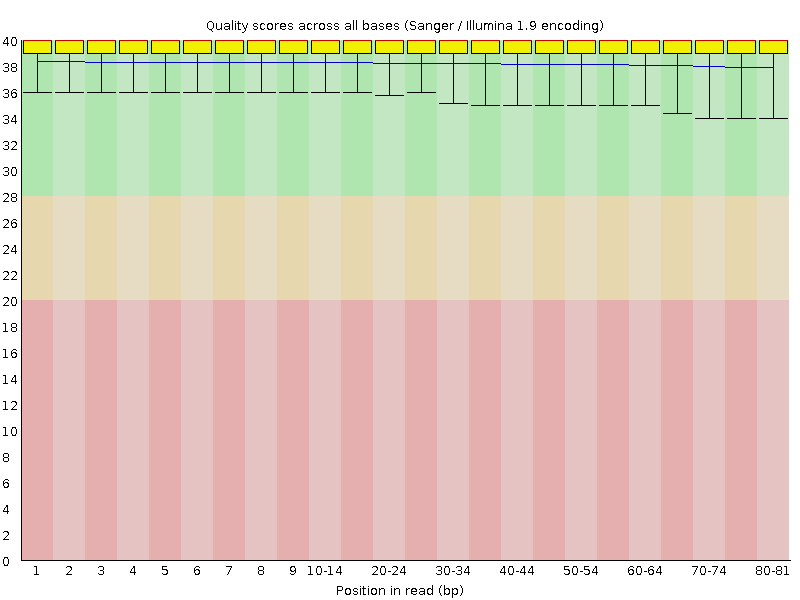

Supplement: Supplementary file 2 [file DataSheet_2.zip › FastQC_optimized/C_S3_L001_R2_001_reverse_paired_fastqc/Images/per_base_quality.png]

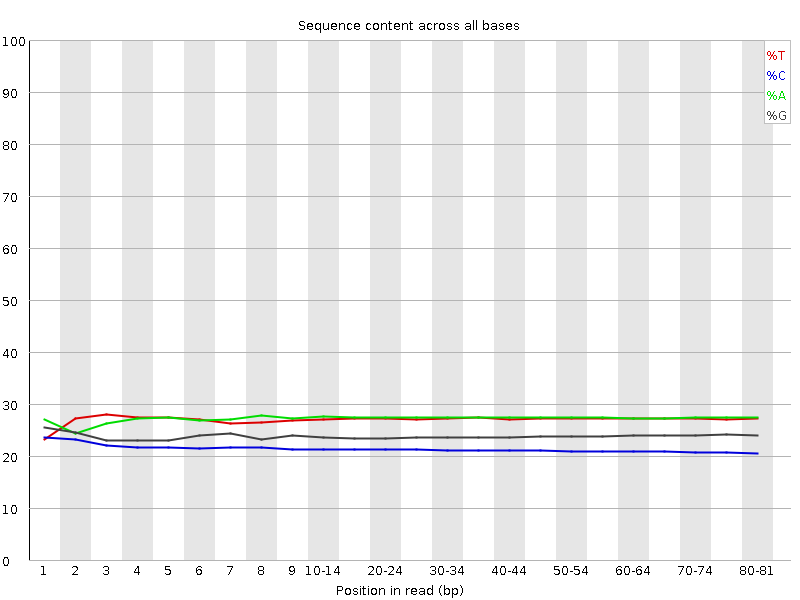

Supplement: Supplementary file 2 [file DataSheet_2.zip › FastQC_optimized/C_S3_L001_R2_001_reverse_paired_fastqc/Images/per_base_sequence_content.png]

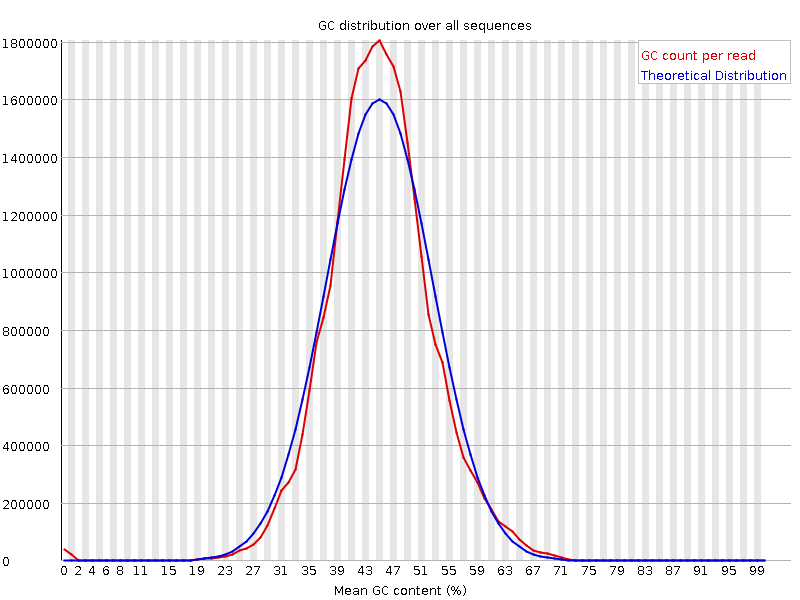

Supplement: Supplementary file 2 [file DataSheet_2.zip › FastQC_optimized/C_S3_L001_R2_001_reverse_paired_fastqc/Images/per_sequence_gc_content.png]

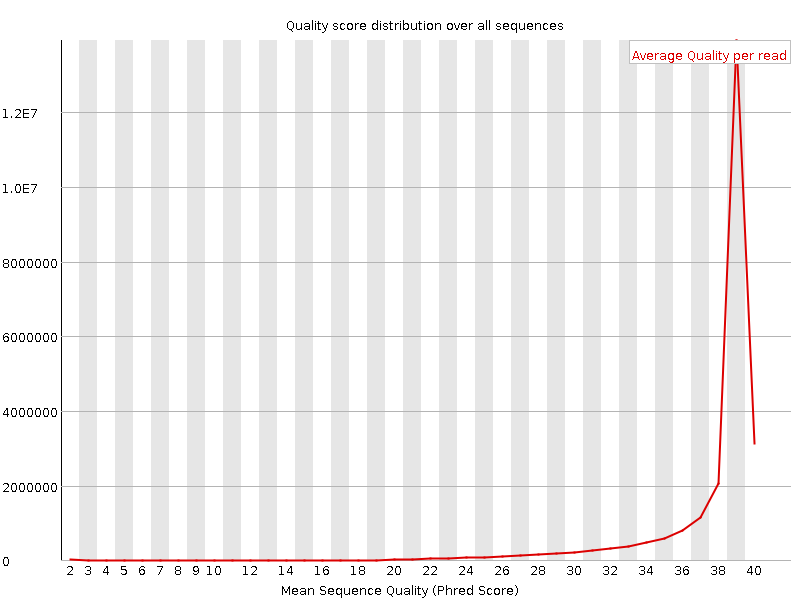

Supplement: Supplementary file 2 [file DataSheet_2.zip › FastQC_optimized/C_S3_L001_R2_001_reverse_paired_fastqc/Images/per_sequence_quality.png]

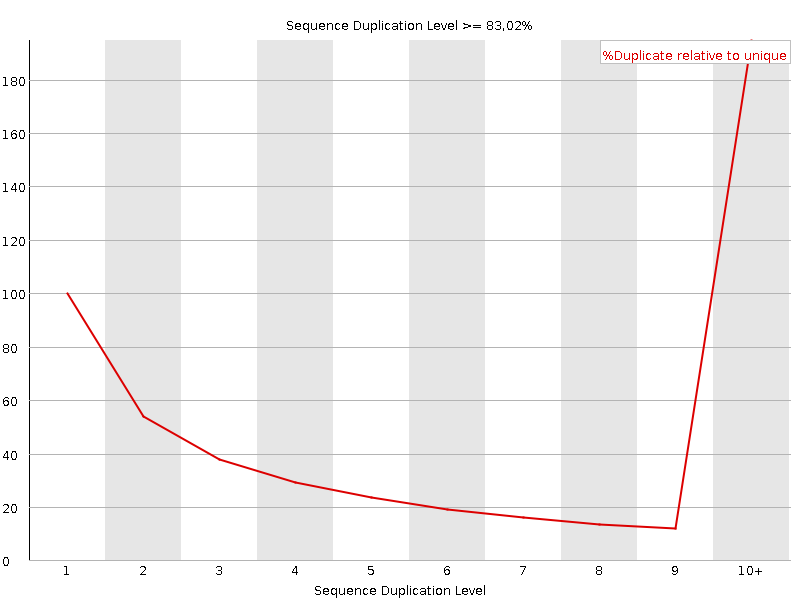

Supplement: Supplementary file 2 [file DataSheet_2.zip › FastQC_optimized/C_S3_L002_R1_001_forward_paired_fastqc/Images/duplication_levels.png]

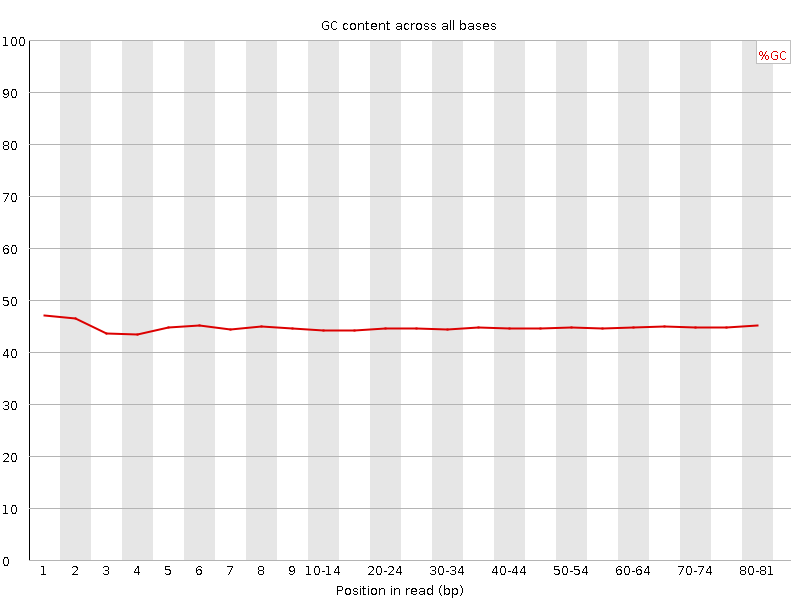

Supplement: Supplementary file 2 [file DataSheet_2.zip › FastQC_optimized/C_S3_L002_R1_001_forward_paired_fastqc/Images/per_base_gc_content.png]

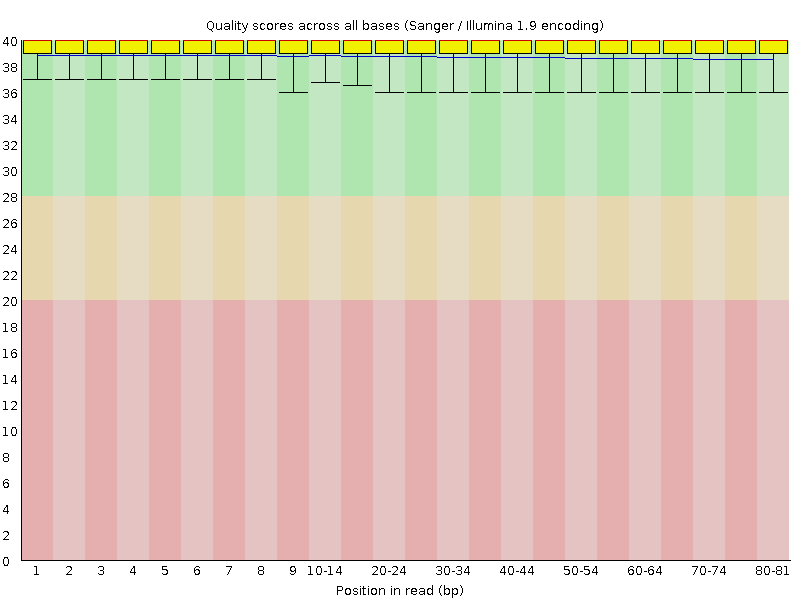

Supplement: Supplementary file 2 [file DataSheet_2.zip › FastQC_optimized/C_S3_L002_R1_001_forward_paired_fastqc/Images/per_base_quality.png]

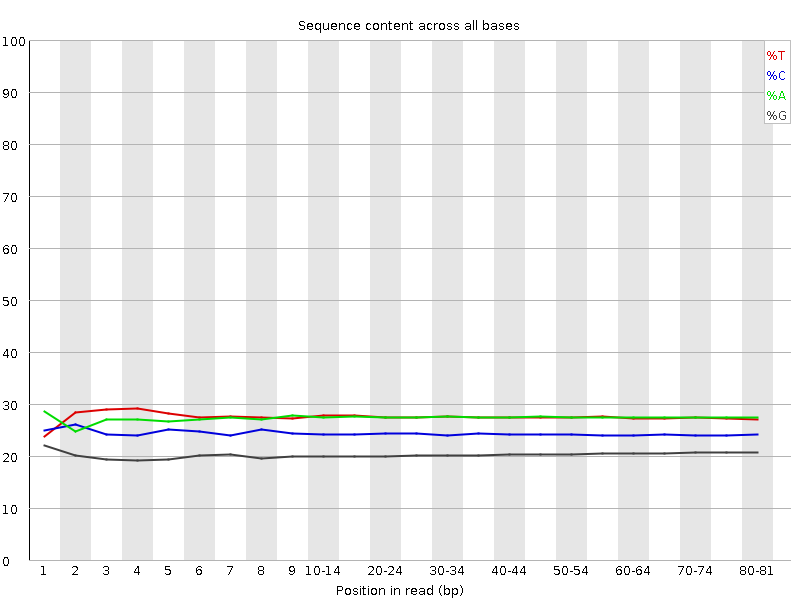

Supplement: Supplementary file 2 [file DataSheet_2.zip › FastQC_optimized/C_S3_L002_R1_001_forward_paired_fastqc/Images/per_base_sequence_content.png]

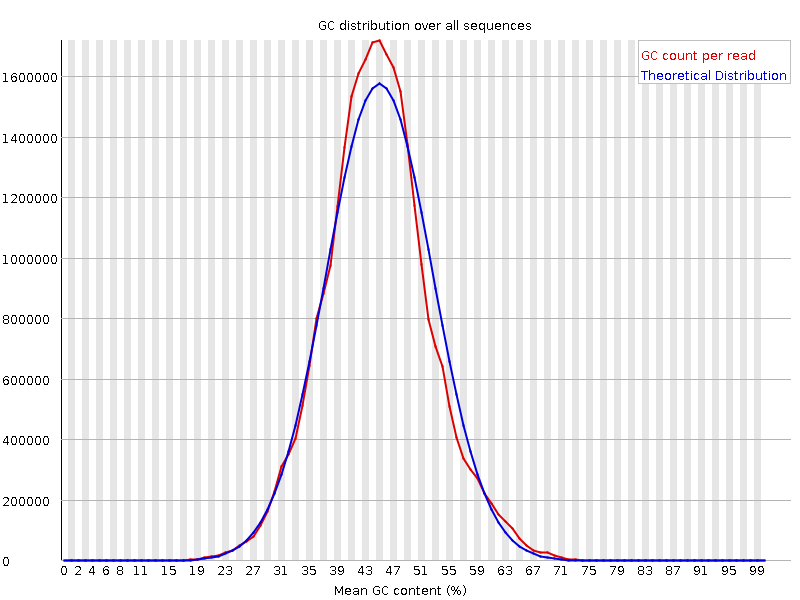

Supplement: Supplementary file 2 [file DataSheet_2.zip › FastQC_optimized/C_S3_L002_R1_001_forward_paired_fastqc/Images/per_sequence_gc_content.png]

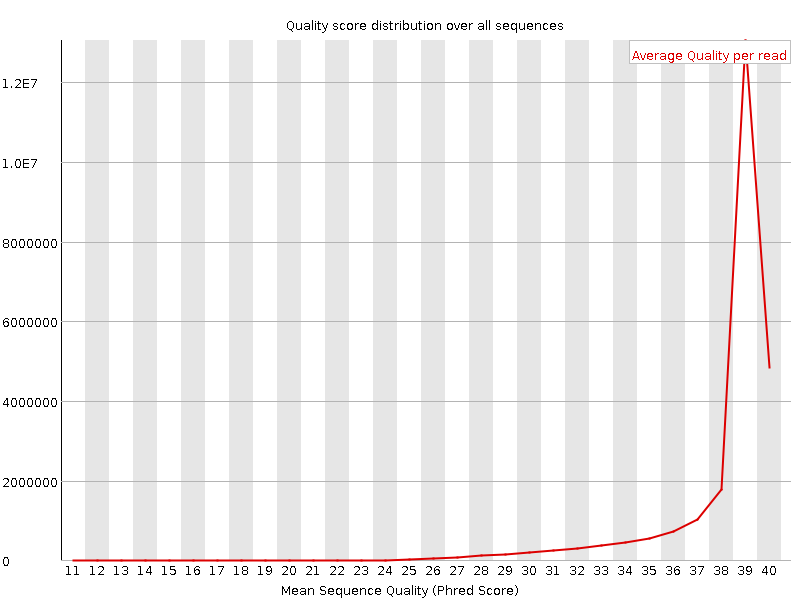

Supplement: Supplementary file 2 [file DataSheet_2.zip › FastQC_optimized/C_S3_L002_R1_001_forward_paired_fastqc/Images/per_sequence_quality.png]

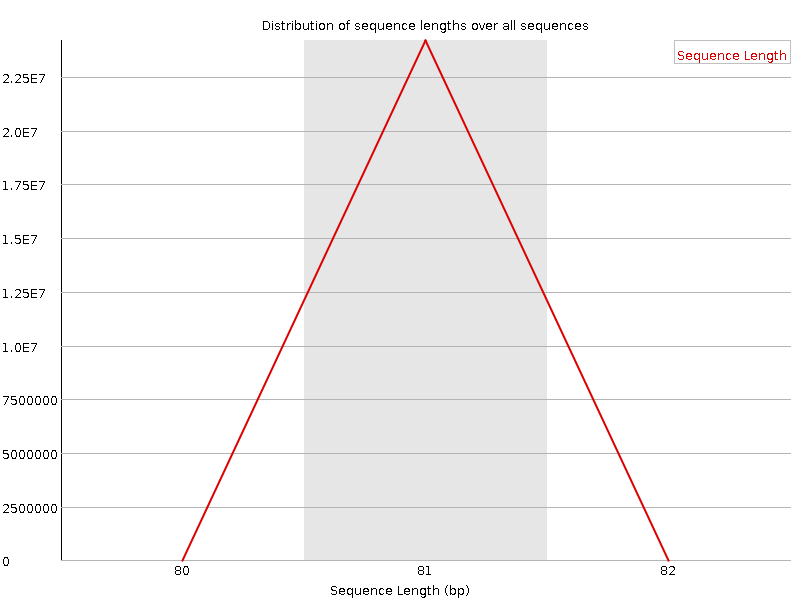

Supplement: Supplementary file 2 [file DataSheet_2.zip › FastQC_optimized/C_S3_L002_R1_001_forward_paired_fastqc/Images/sequence_length_distribution.png]

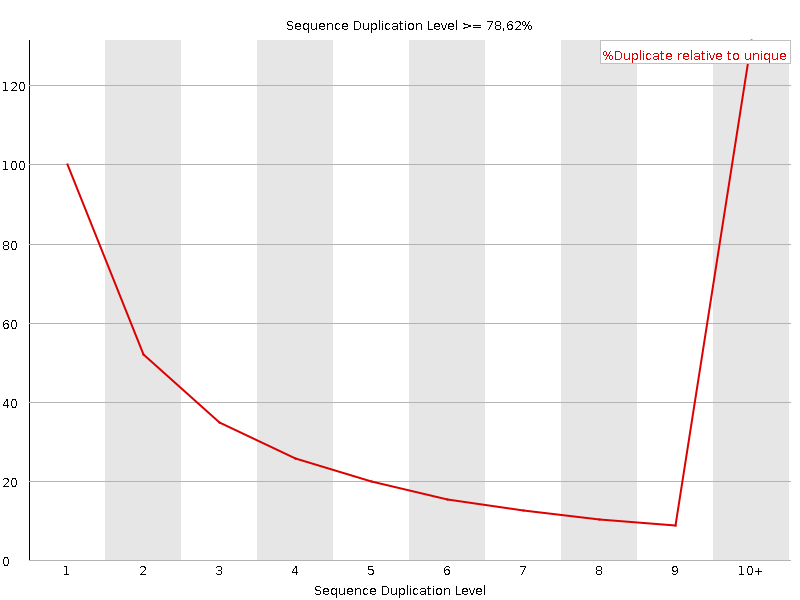

Supplement: Supplementary file 2 [file DataSheet_2.zip › FastQC_optimized/C_S3_L002_R2_001_reverse_paired_fastqc/Images/duplication_levels.png]

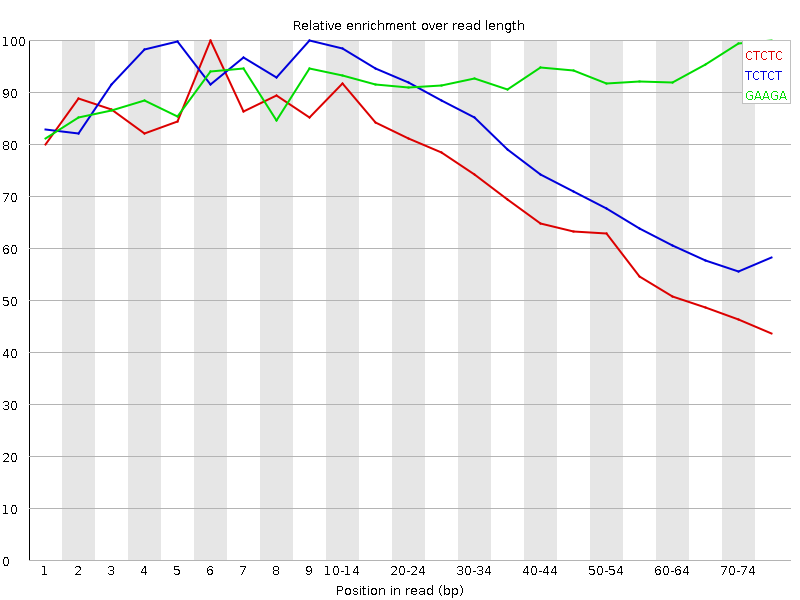

Supplement: Supplementary file 2 [file DataSheet_2.zip › FastQC_optimized/C_S3_L002_R2_001_reverse_paired_fastqc/Images/kmer_profiles.png]

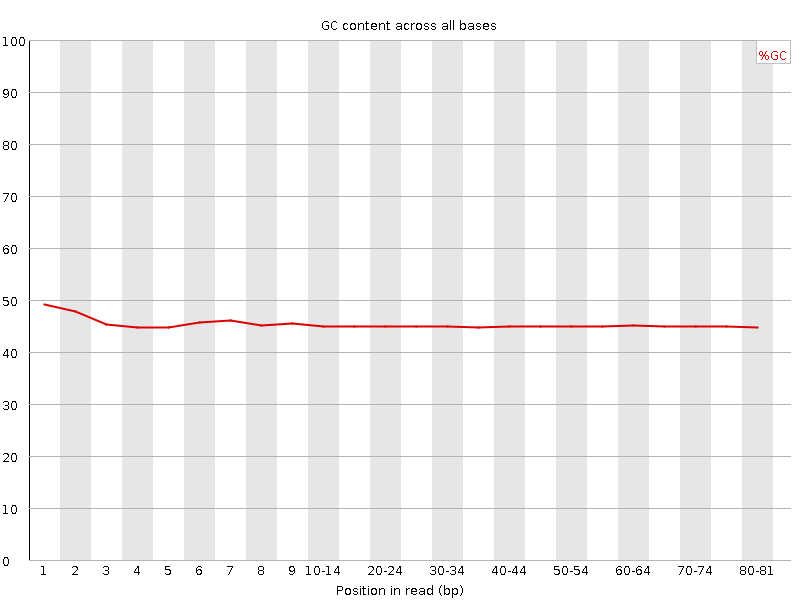

Supplement: Supplementary file 2 [file DataSheet_2.zip › FastQC_optimized/C_S3_L002_R2_001_reverse_paired_fastqc/Images/per_base_gc_content.png]

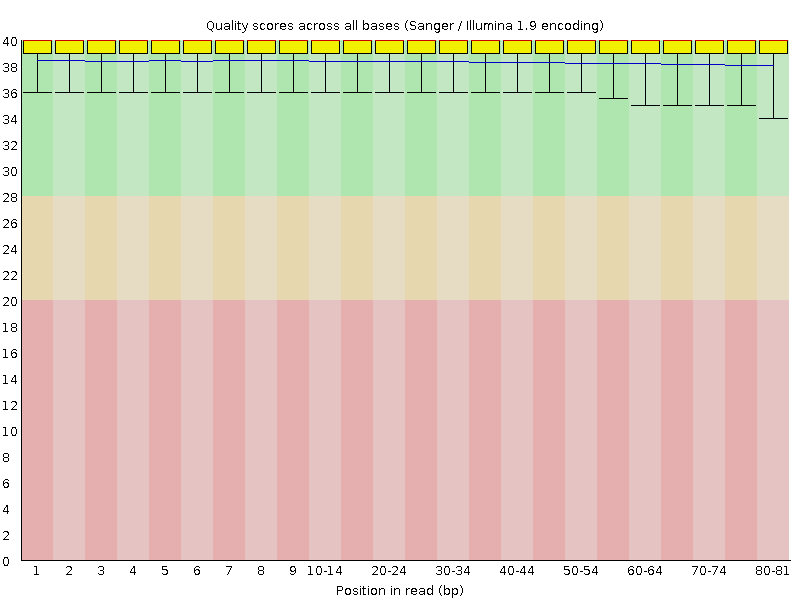

Supplement: Supplementary file 2 [file DataSheet_2.zip › FastQC_optimized/C_S3_L002_R2_001_reverse_paired_fastqc/Images/per_base_quality.png]

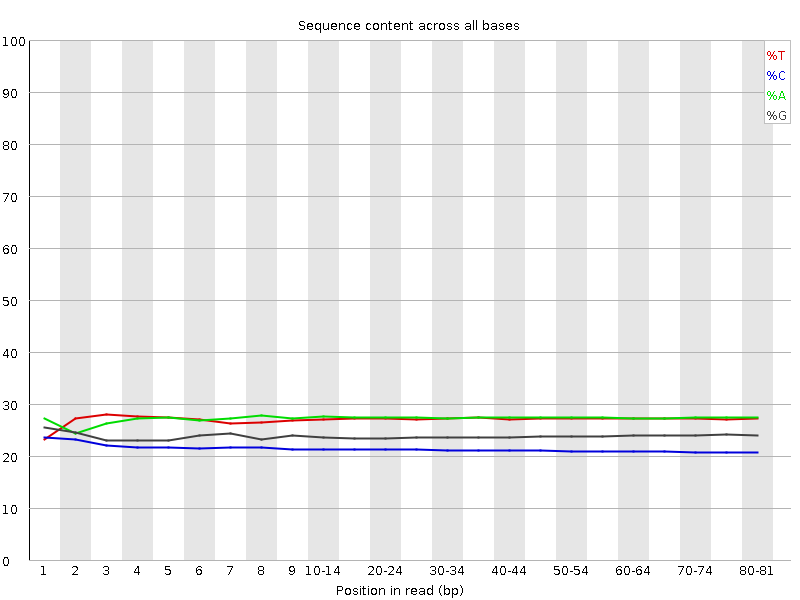

Supplement: Supplementary file 2 [file DataSheet_2.zip › FastQC_optimized/C_S3_L002_R2_001_reverse_paired_fastqc/Images/per_base_sequence_content.png]

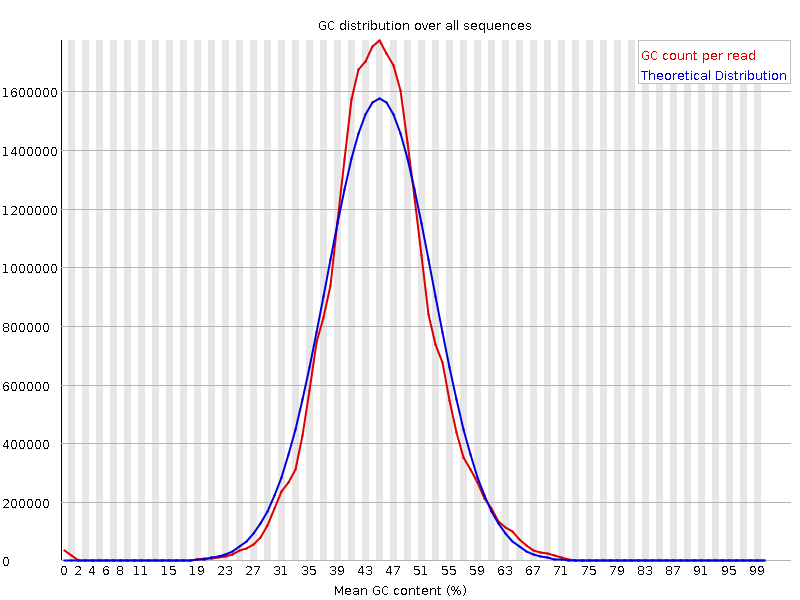

Supplement: Supplementary file 2 [file DataSheet_2.zip › FastQC_optimized/C_S3_L002_R2_001_reverse_paired_fastqc/Images/per_sequence_gc_content.png]

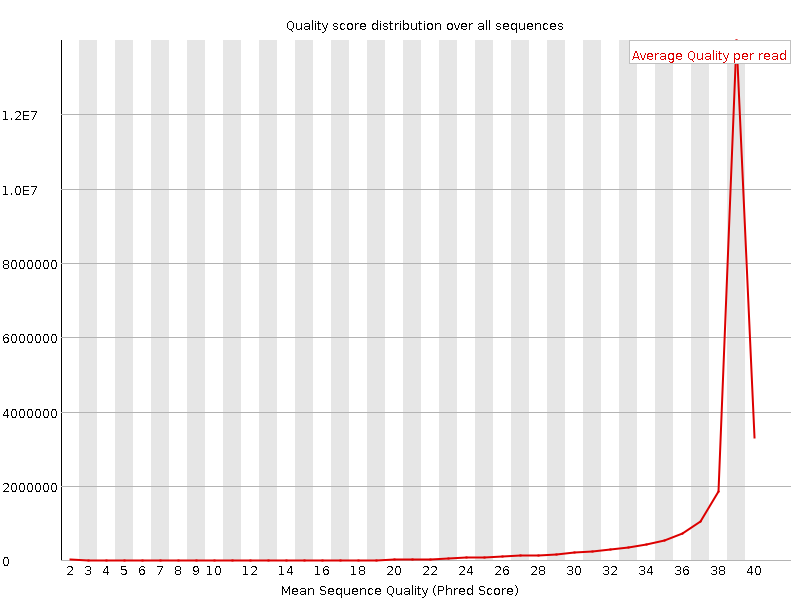

Supplement: Supplementary file 2 [file DataSheet_2.zip › FastQC_optimized/C_S3_L002_R2_001_reverse_paired_fastqc/Images/per_sequence_quality.png]

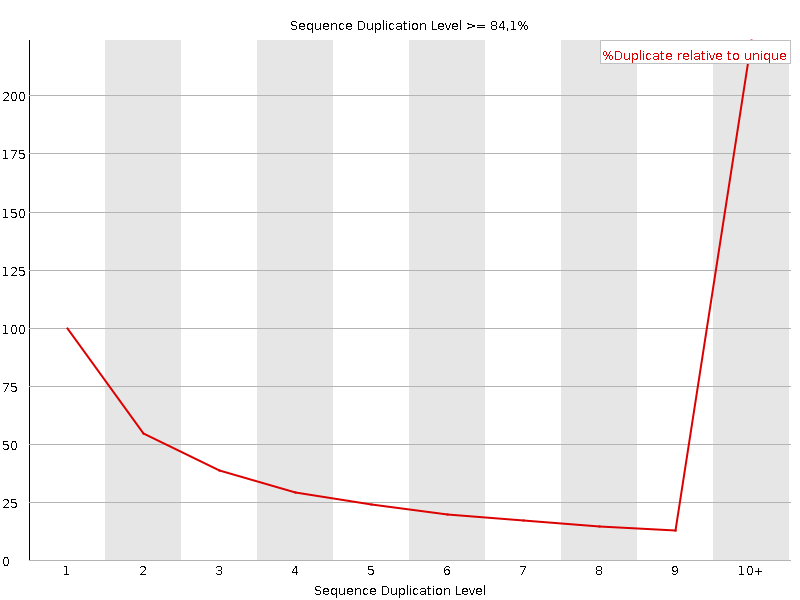

Supplement: Supplementary file 2 [file DataSheet_2.zip › FastQC_optimized/D_S4_L001_R1_001_forward_paired_fastqc/Images/duplication_levels.png]

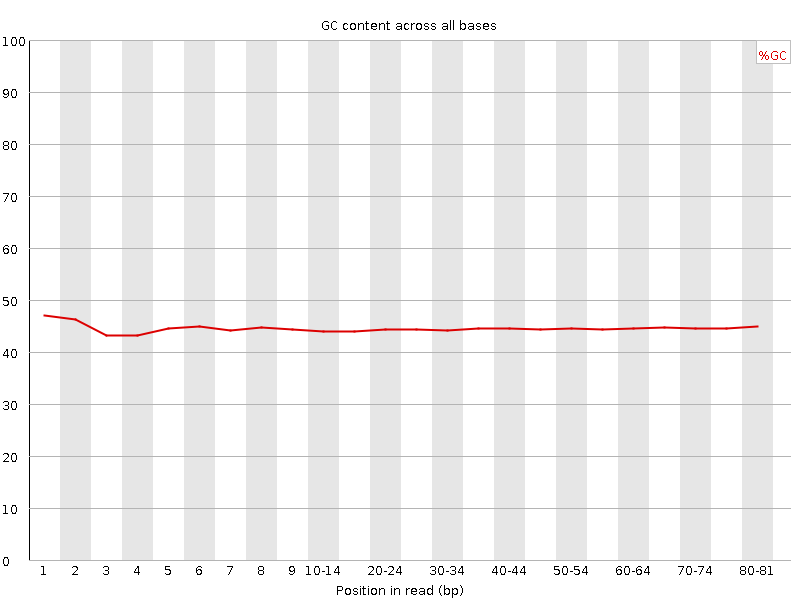

Supplement: Supplementary file 2 [file DataSheet_2.zip › FastQC_optimized/D_S4_L001_R1_001_forward_paired_fastqc/Images/per_base_gc_content.png]

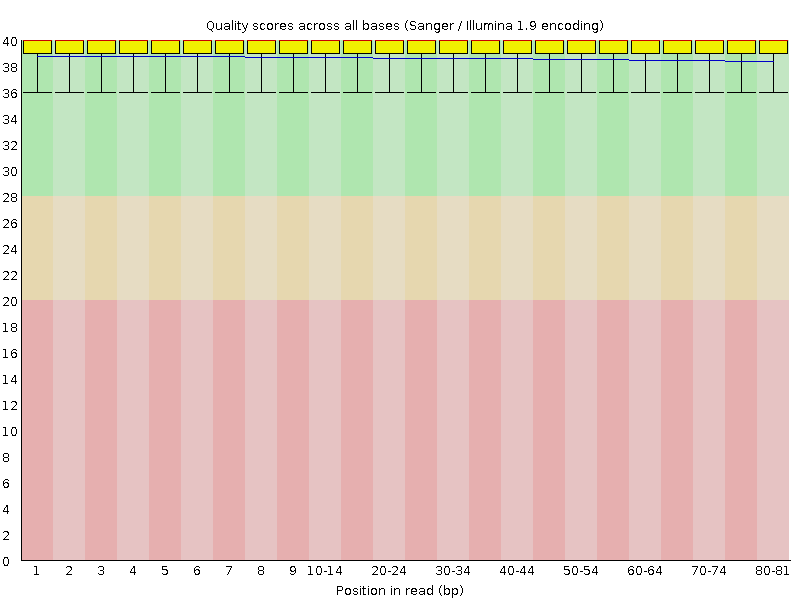

Supplement: Supplementary file 2 [file DataSheet_2.zip › FastQC_optimized/D_S4_L001_R1_001_forward_paired_fastqc/Images/per_base_quality.png]

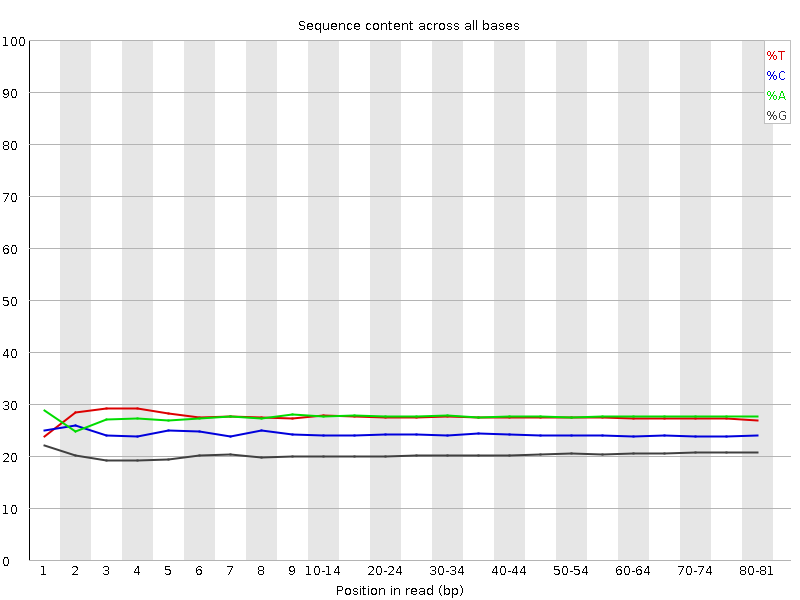

Supplement: Supplementary file 2 [file DataSheet_2.zip › FastQC_optimized/D_S4_L001_R1_001_forward_paired_fastqc/Images/per_base_sequence_content.png]

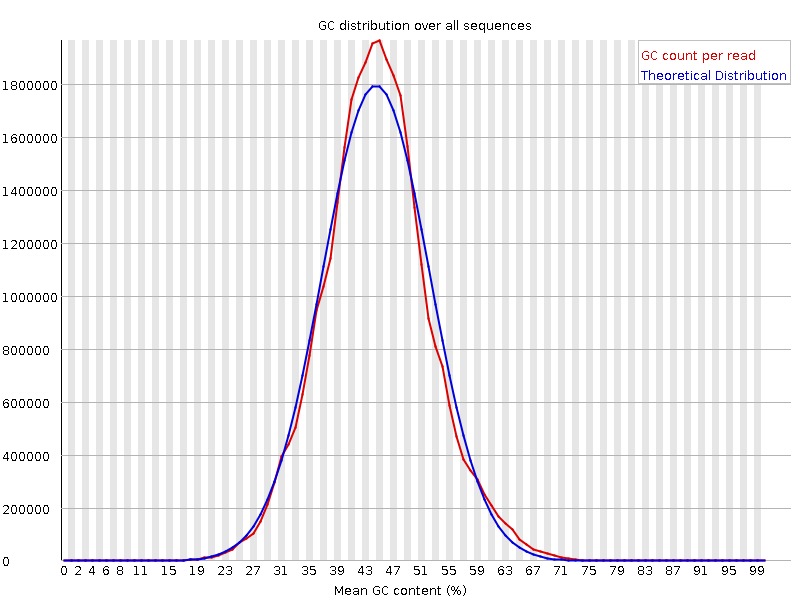

Supplement: Supplementary file 2 [file DataSheet_2.zip › FastQC_optimized/D_S4_L001_R1_001_forward_paired_fastqc/Images/per_sequence_gc_content.png]

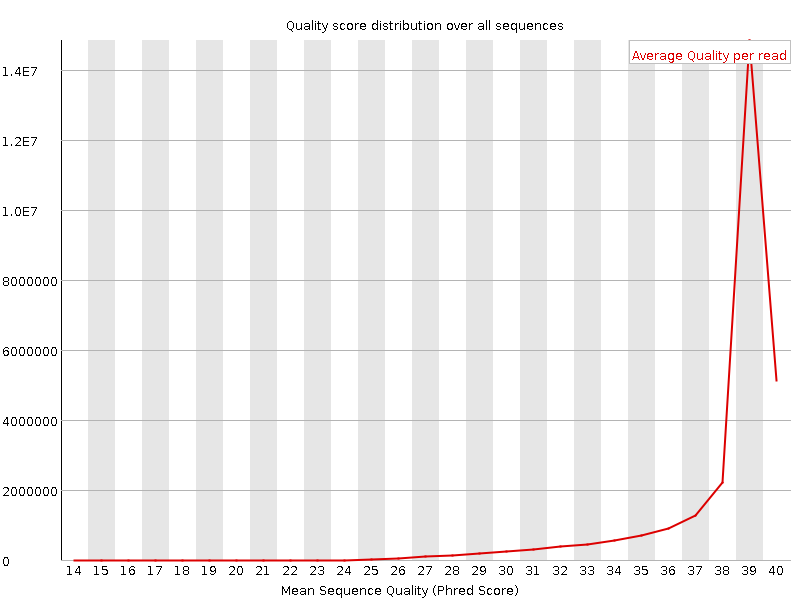

Supplement: Supplementary file 2 [file DataSheet_2.zip › FastQC_optimized/D_S4_L001_R1_001_forward_paired_fastqc/Images/per_sequence_quality.png]

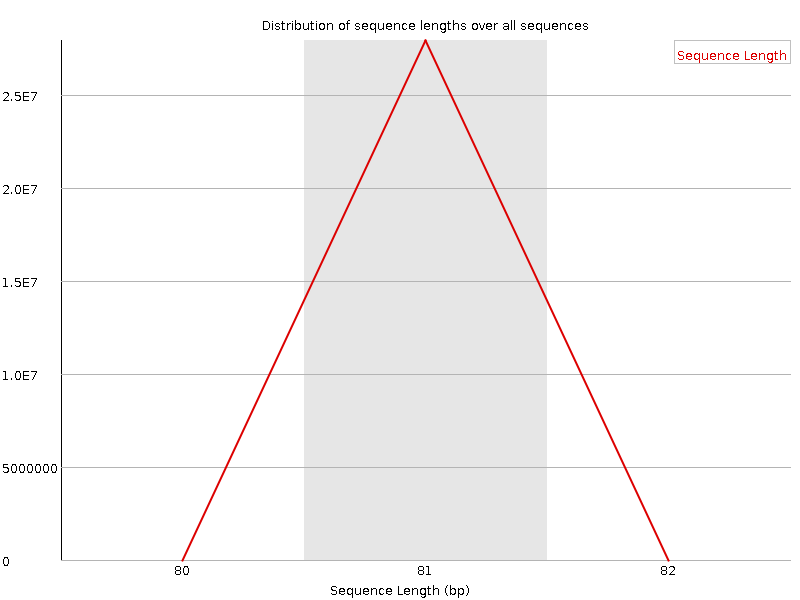

Supplement: Supplementary file 2 [file DataSheet_2.zip › FastQC_optimized/D_S4_L001_R1_001_forward_paired_fastqc/Images/sequence_length_distribution.png]

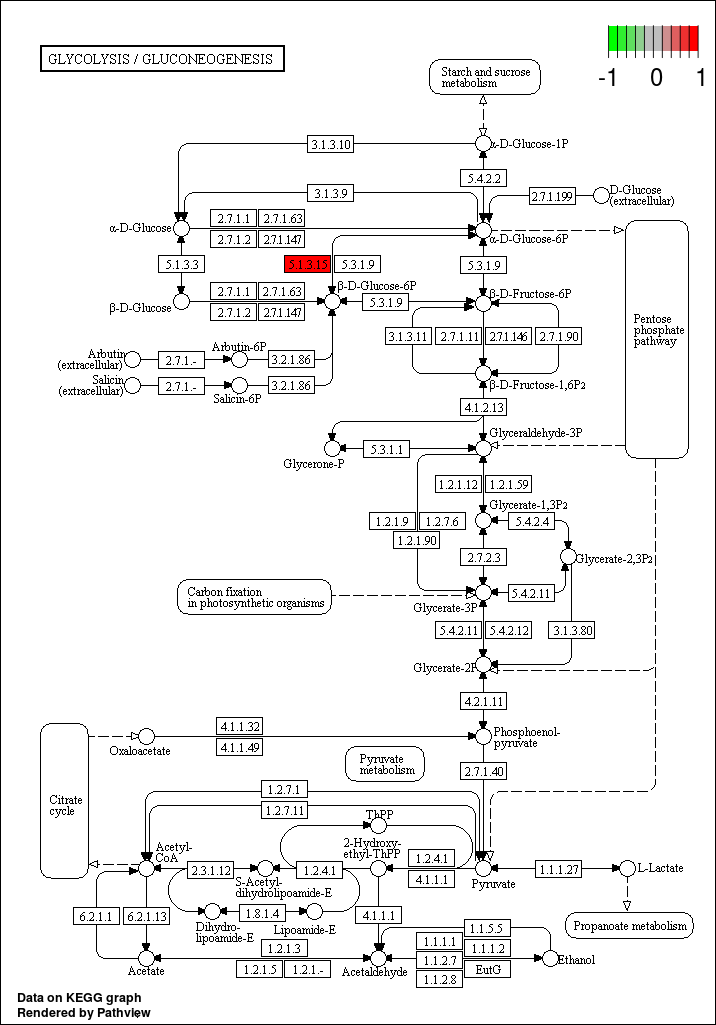

Supplement: Supplementary file 3 [file DataSheet_3.zip › Pathview_results/pmum00010.pathview.png]

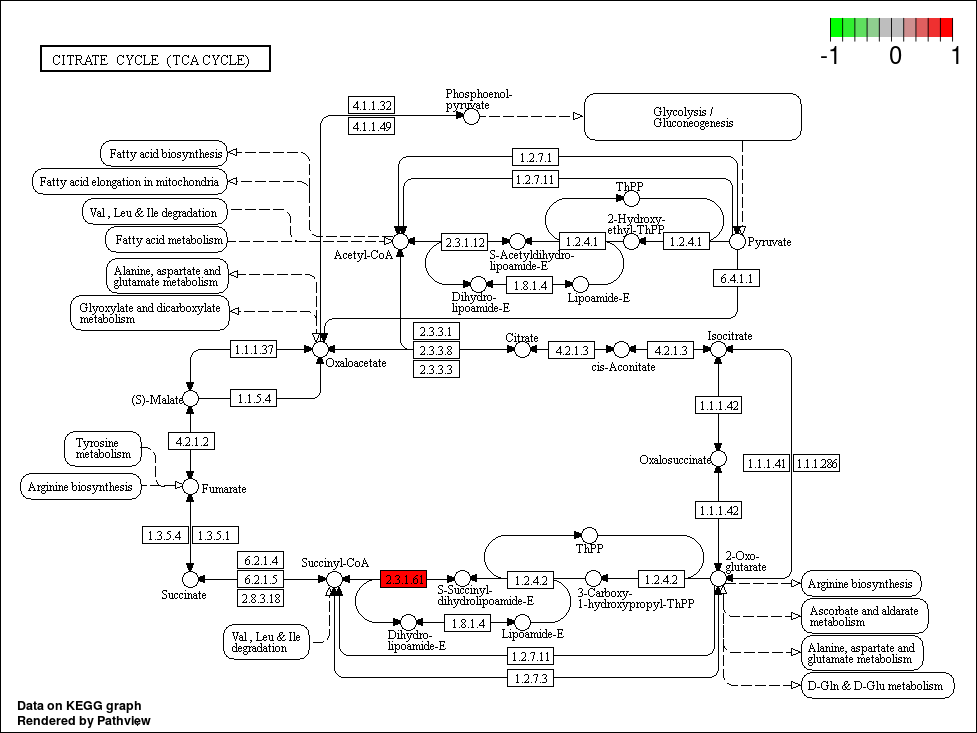

Supplement: Supplementary file 3 [file DataSheet_3.zip › Pathview_results/pmum00020.pathview.png]

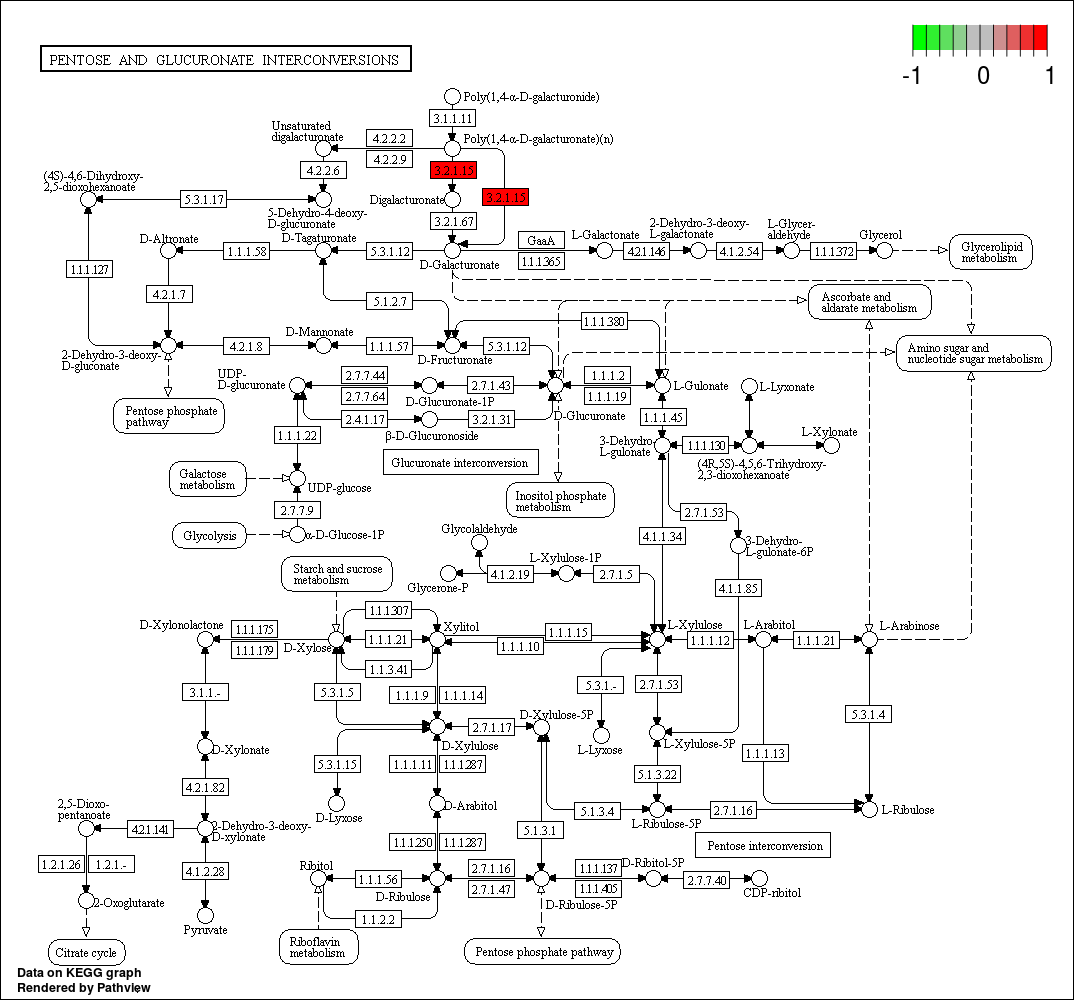

Supplement: Supplementary file 3 [file DataSheet_3.zip › Pathview_results/pmum00040.pathview.png]

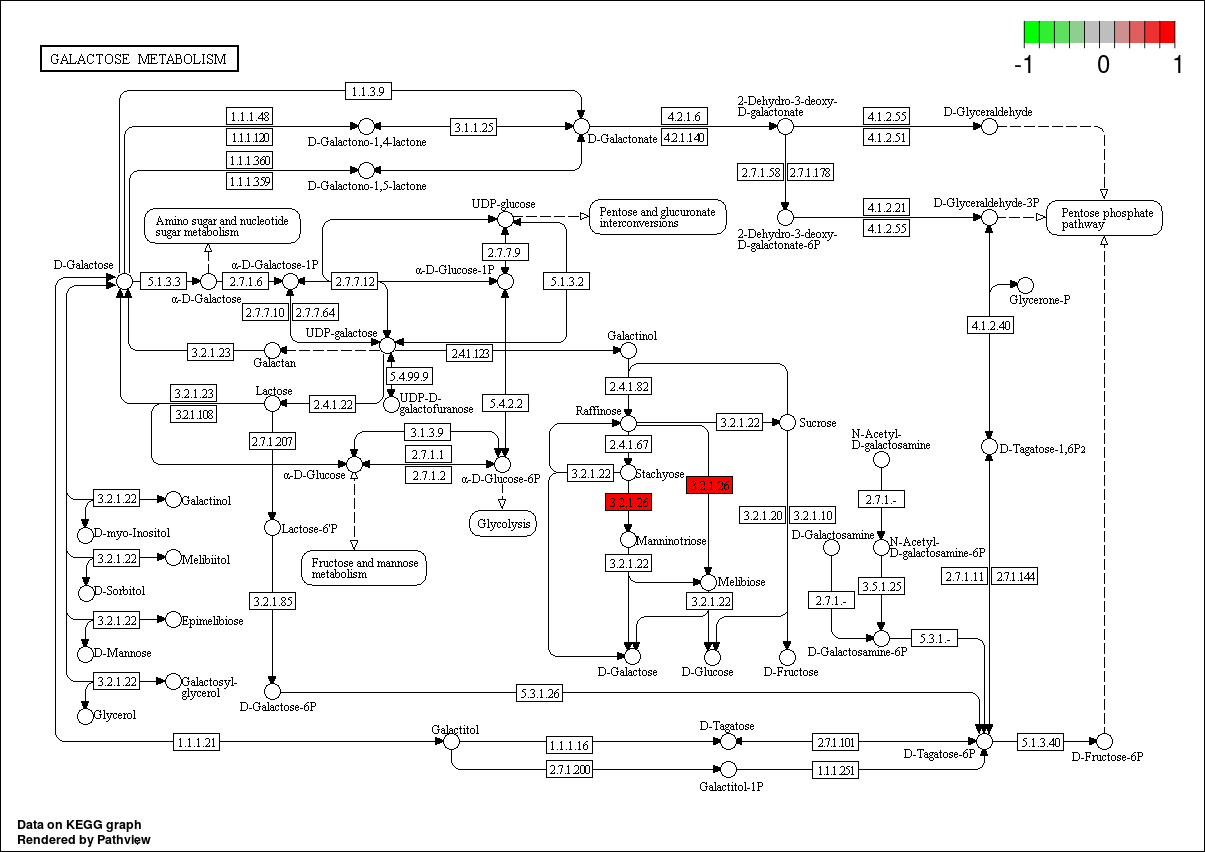

Supplement: Supplementary file 3 [file DataSheet_3.zip › Pathview_results/pmum00052.pathview.png]

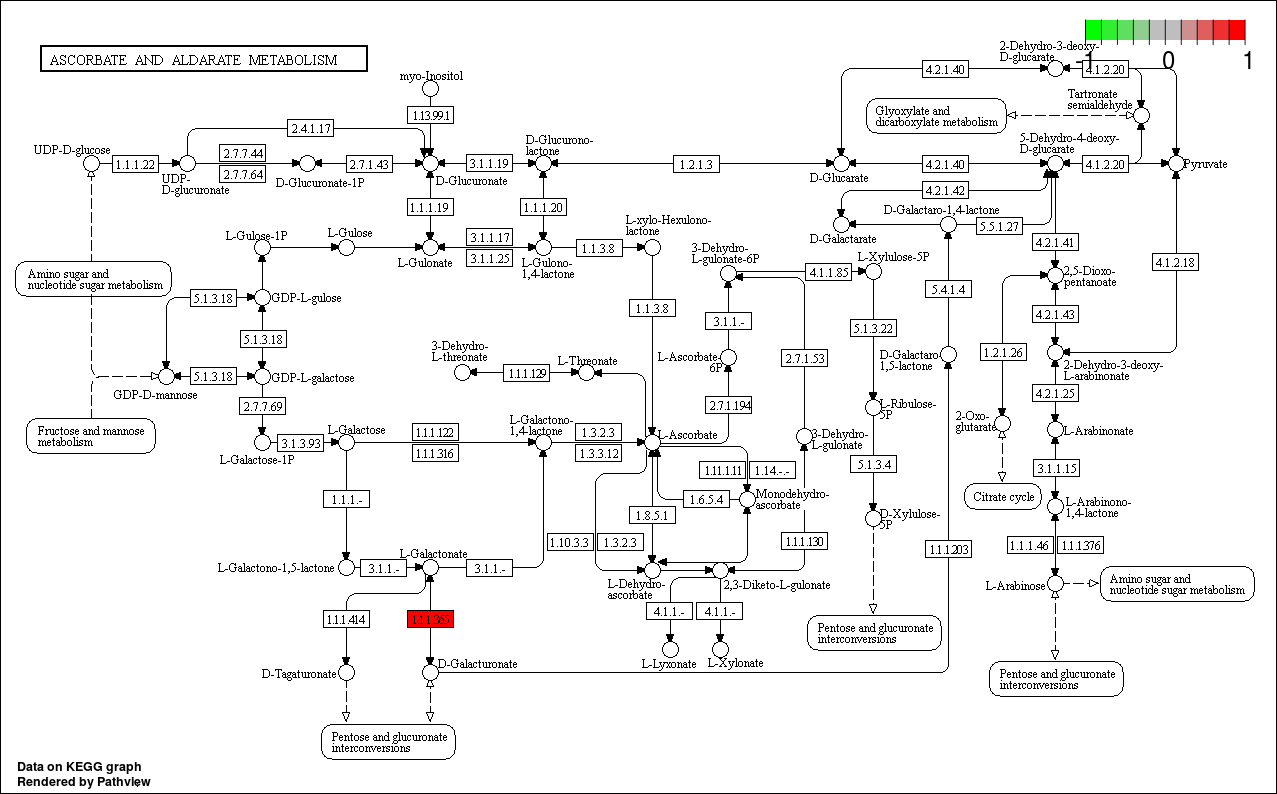

Supplement: Supplementary file 3 [file DataSheet_3.zip › Pathview_results/pmum00053.pathview.png]

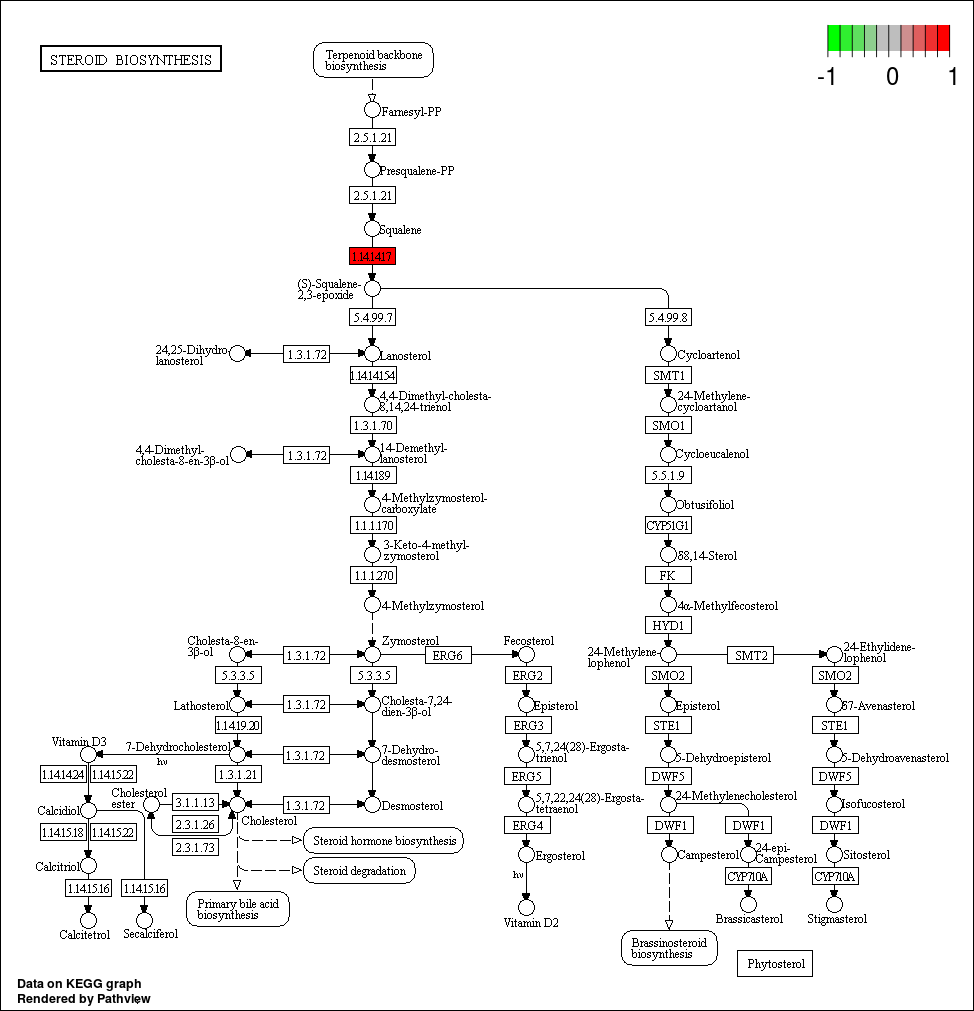

Supplement: Supplementary file 3 [file DataSheet_3.zip › Pathview_results/pmum00100.pathview.png]

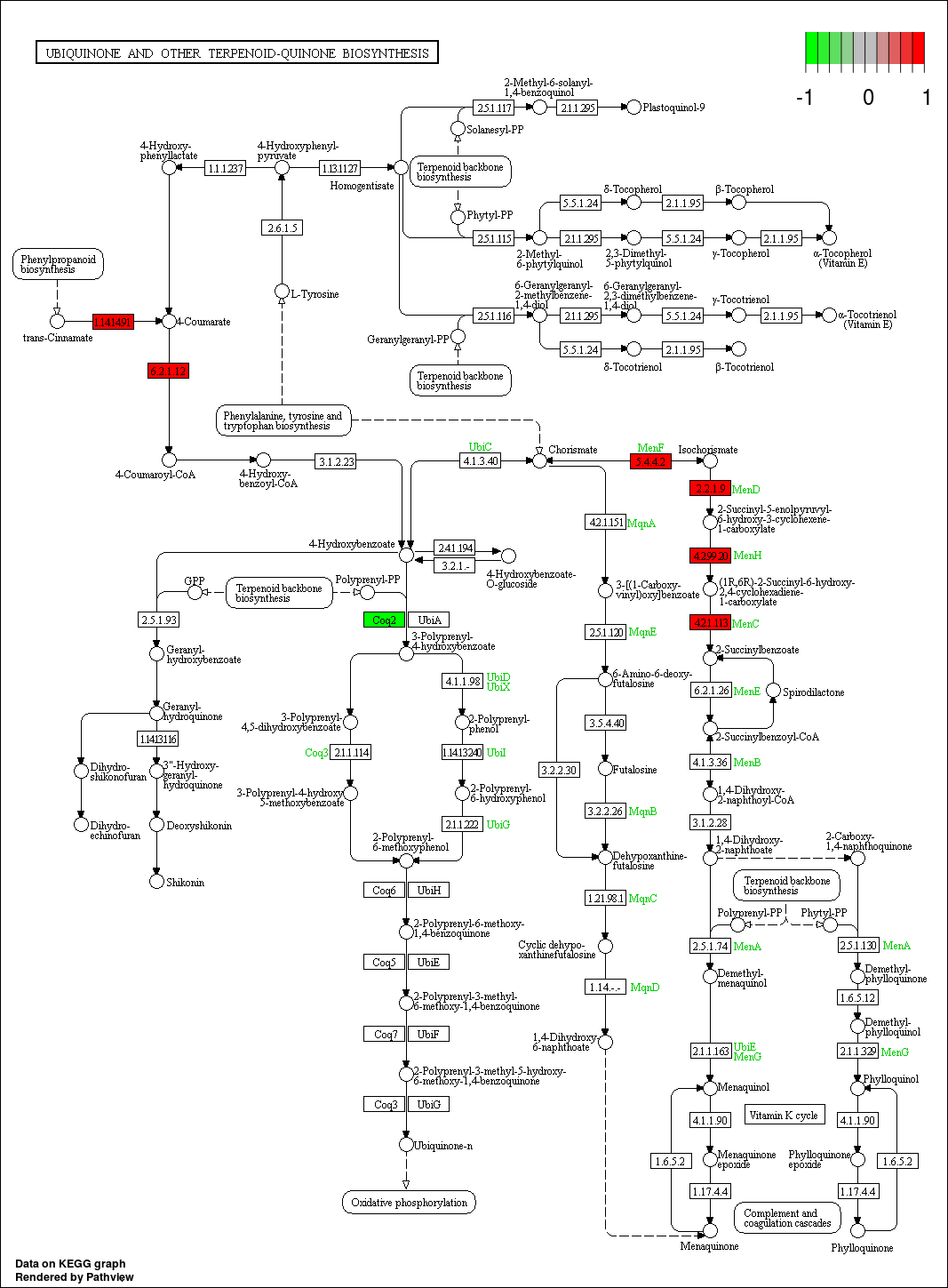

Supplement: Supplementary file 3 [file DataSheet_3.zip › Pathview_results/pmum00130.pathview.png]

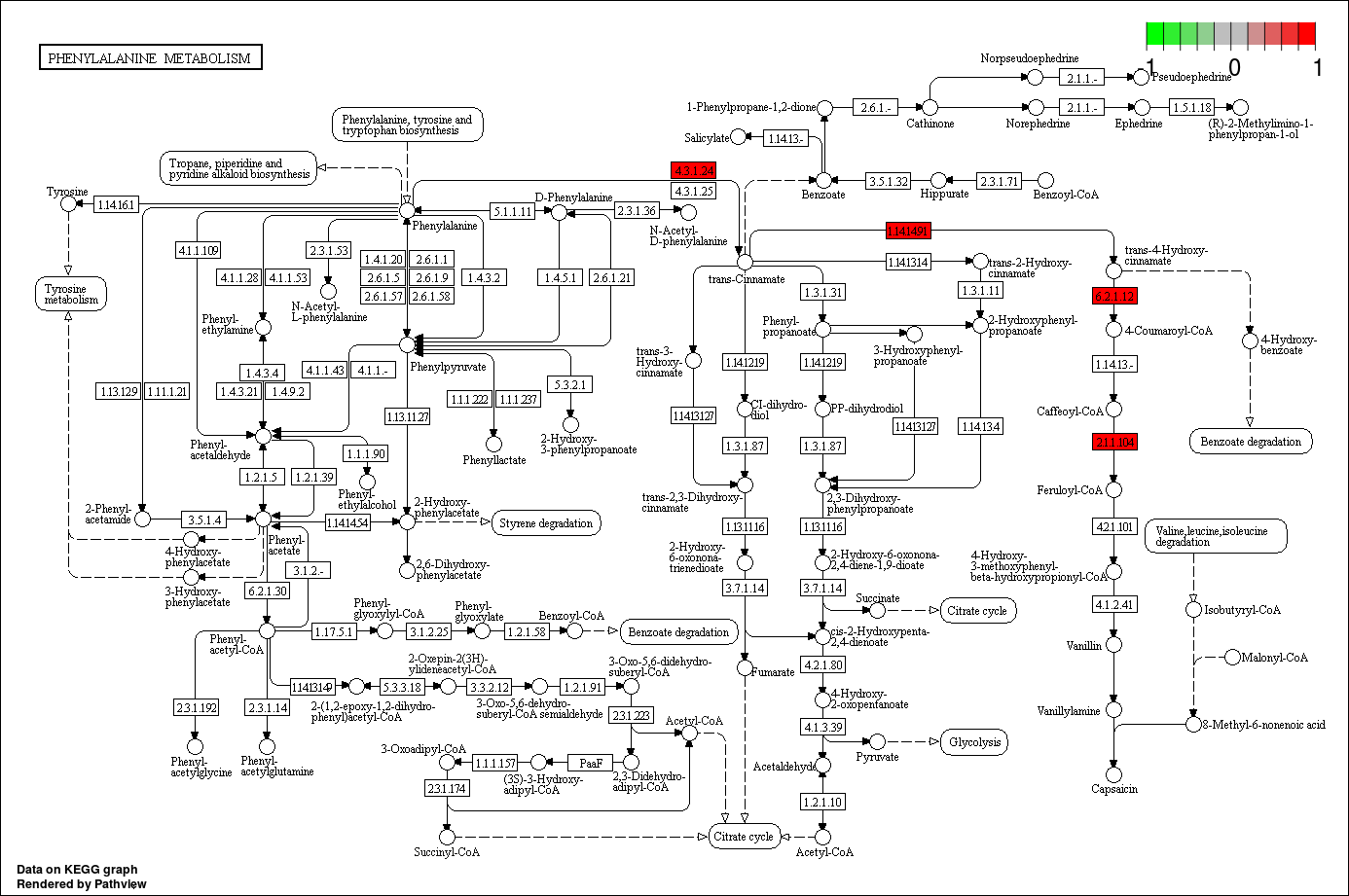

Supplement: Supplementary file 3 [file DataSheet_3.zip › Pathview_results/pmum00360.pathview.png]

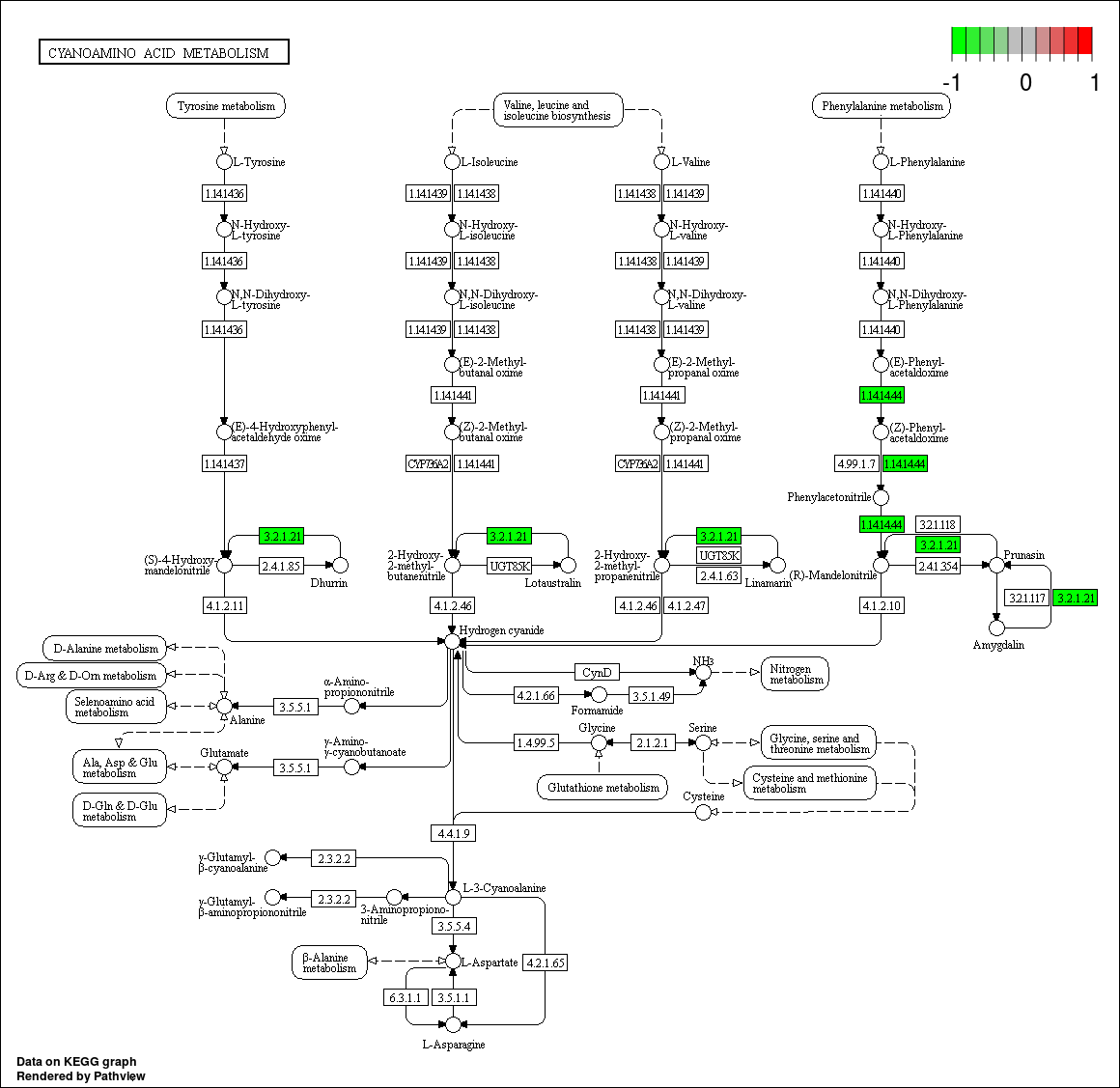

Supplement: Supplementary file 3 [file DataSheet_3.zip › Pathview_results/pmum00460.pathview.png]

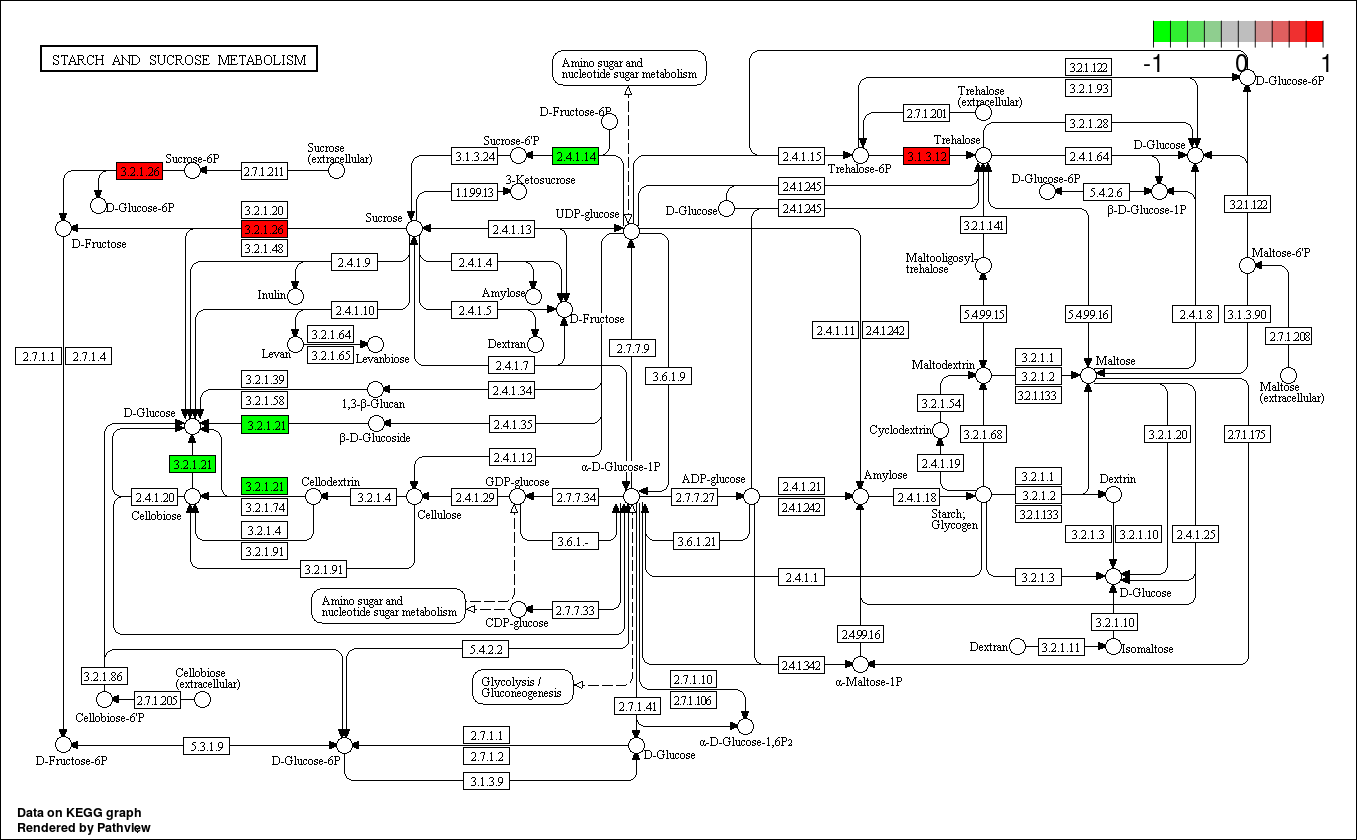

Supplement: Supplementary file 3 [file DataSheet_3.zip › Pathview_results/pmum00500.pathview.png]

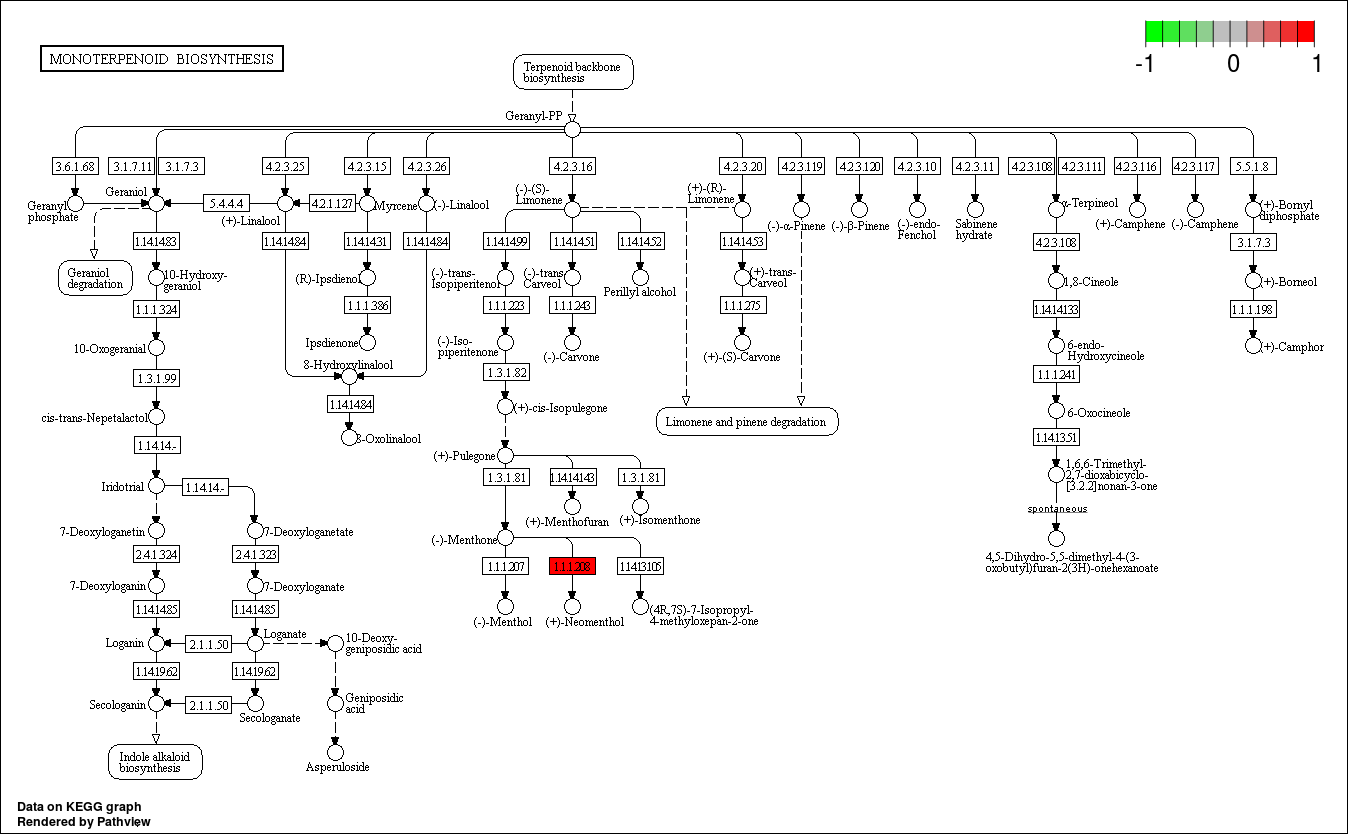

Supplement: Supplementary file 3 [file DataSheet_3.zip › Pathview_results/pmum00902.pathview.png]

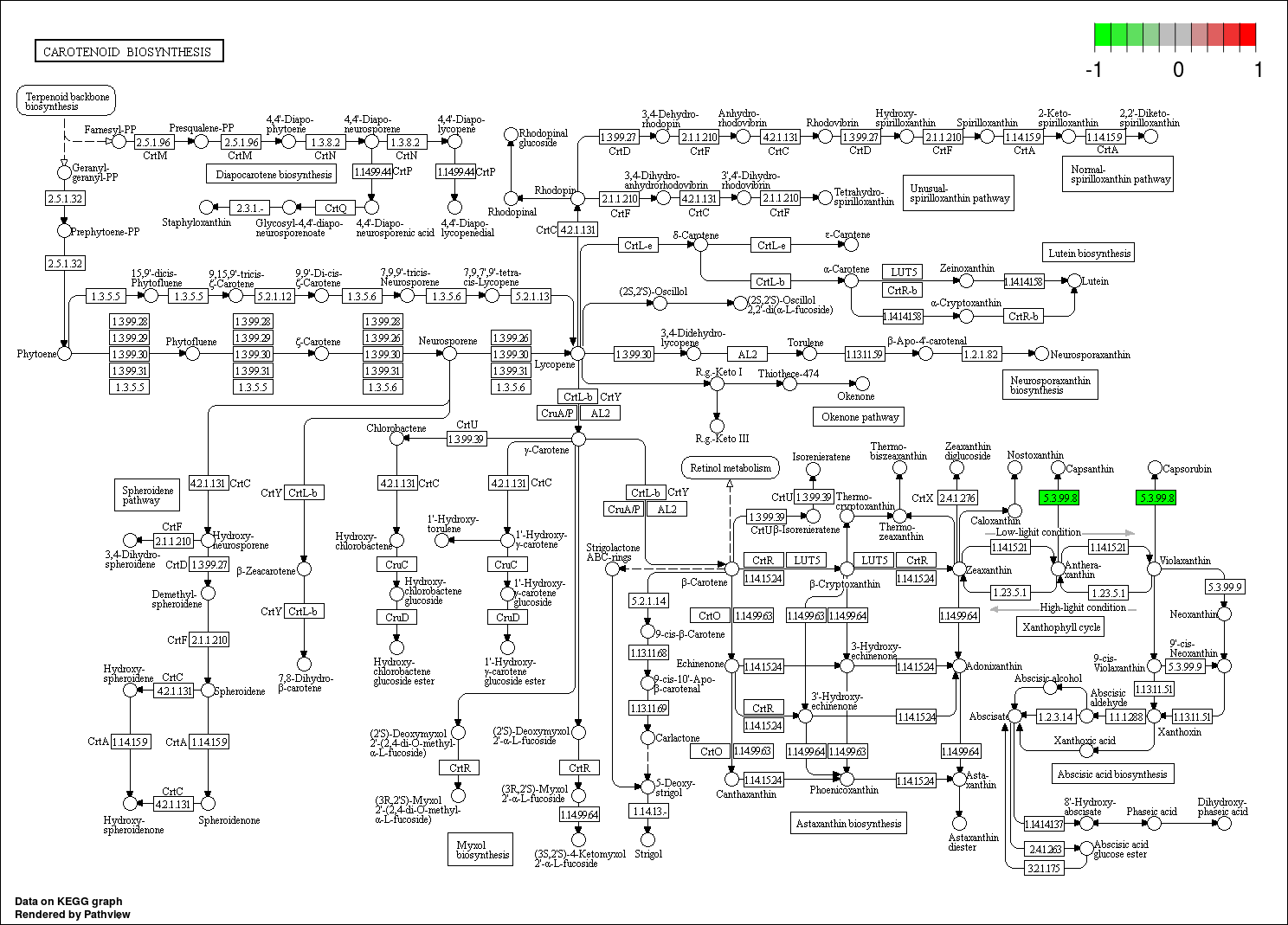

Supplement: Supplementary file 3 [file DataSheet_3.zip › Pathview_results/pmum00906.pathview.png]
