# Supplementary figures and images for: Analysis of Metabolites and Gene Expression Changes Relative to Apricot (Prunus armeniaca L.) Fruit Quality During Development and Ripening (part 3 of 3)
Source: Front Plant Sci. 2020 Aug 19;11:1269. doi: 10.3389/fpls.2020.01269 (PMC7466674; doi:10.3389/fpls.2020.01269)

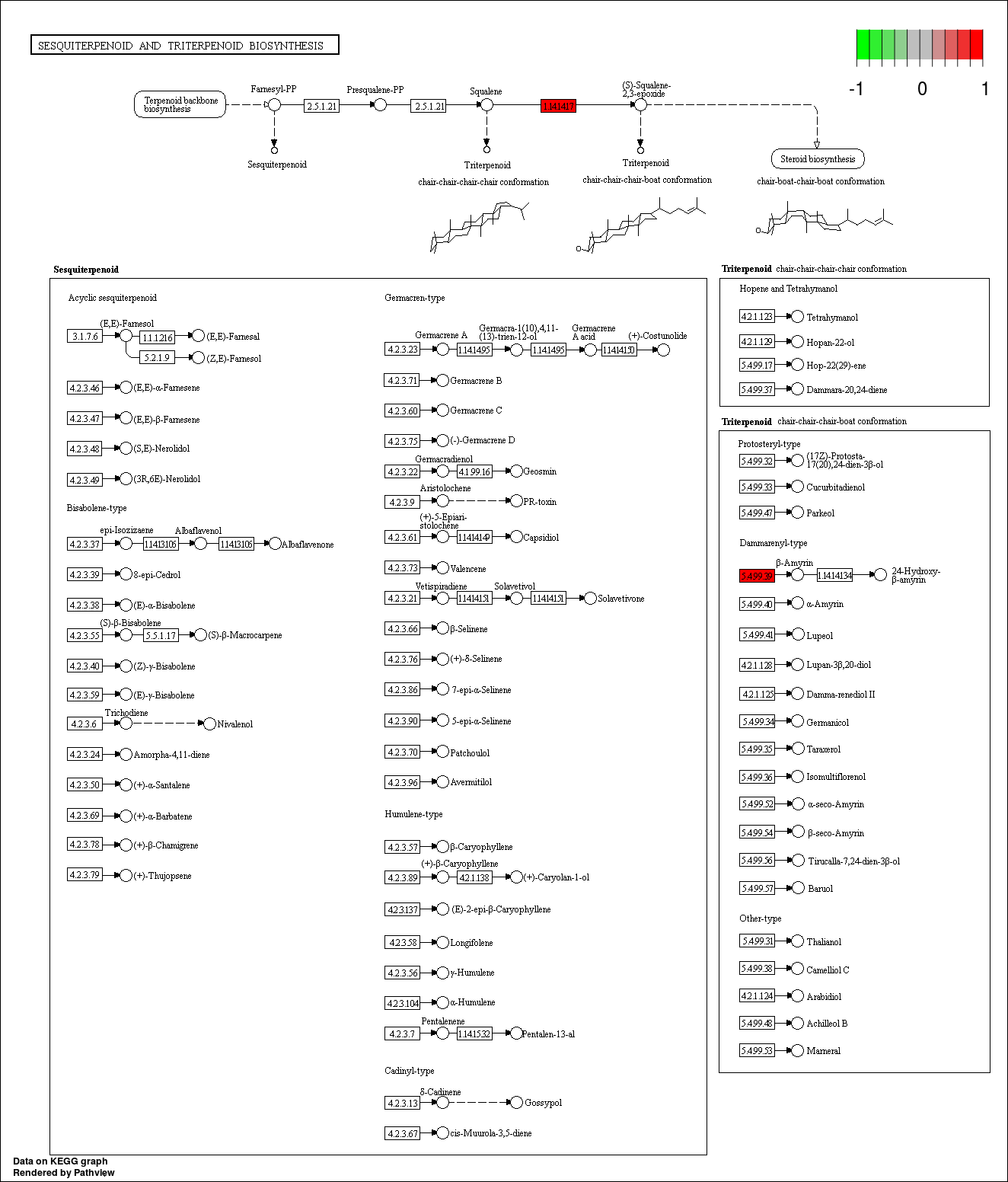

Supplement: Supplementary file 3 [file DataSheet_3.zip › Pathview_results/pmum00909.pathview.png]

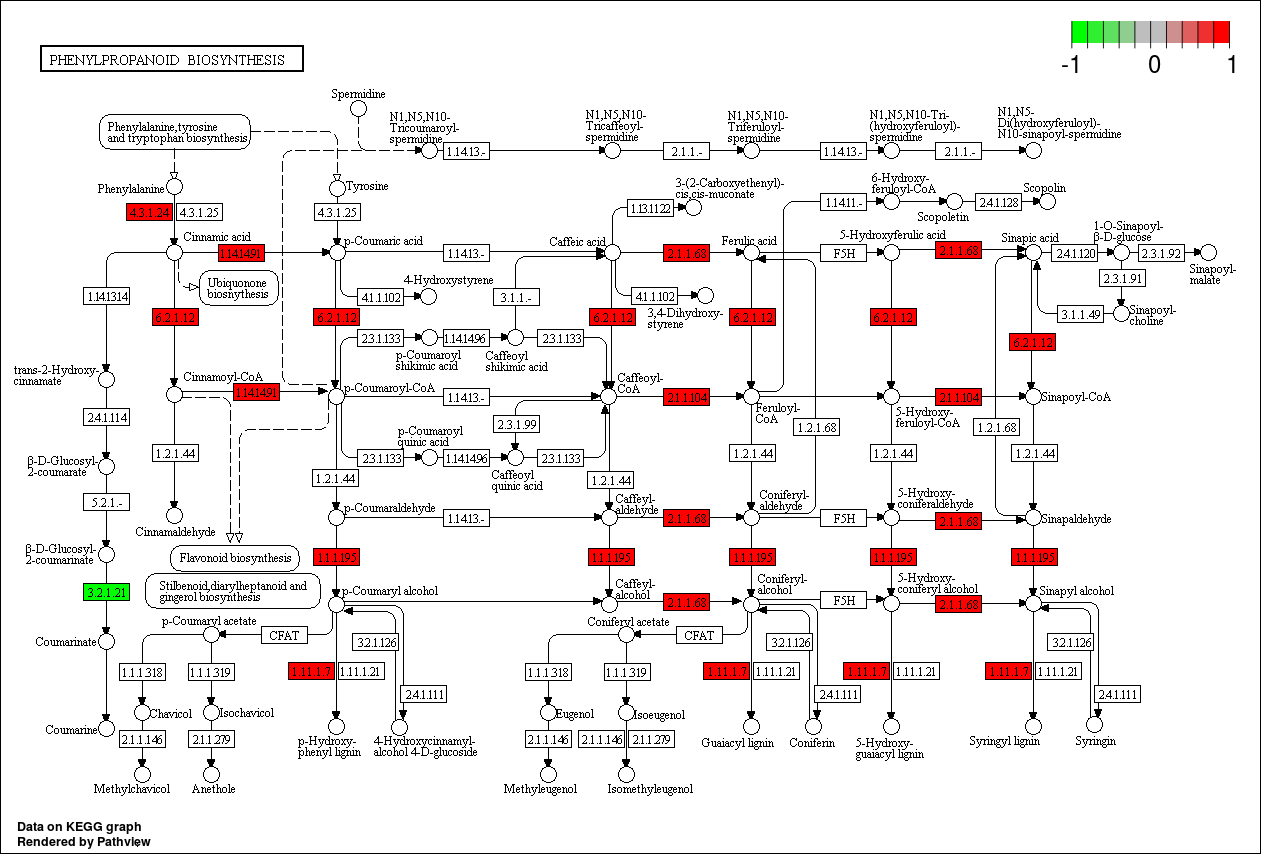

Supplement: Supplementary file 3 [file DataSheet_3.zip › Pathview_results/pmum00940.pathview.png]

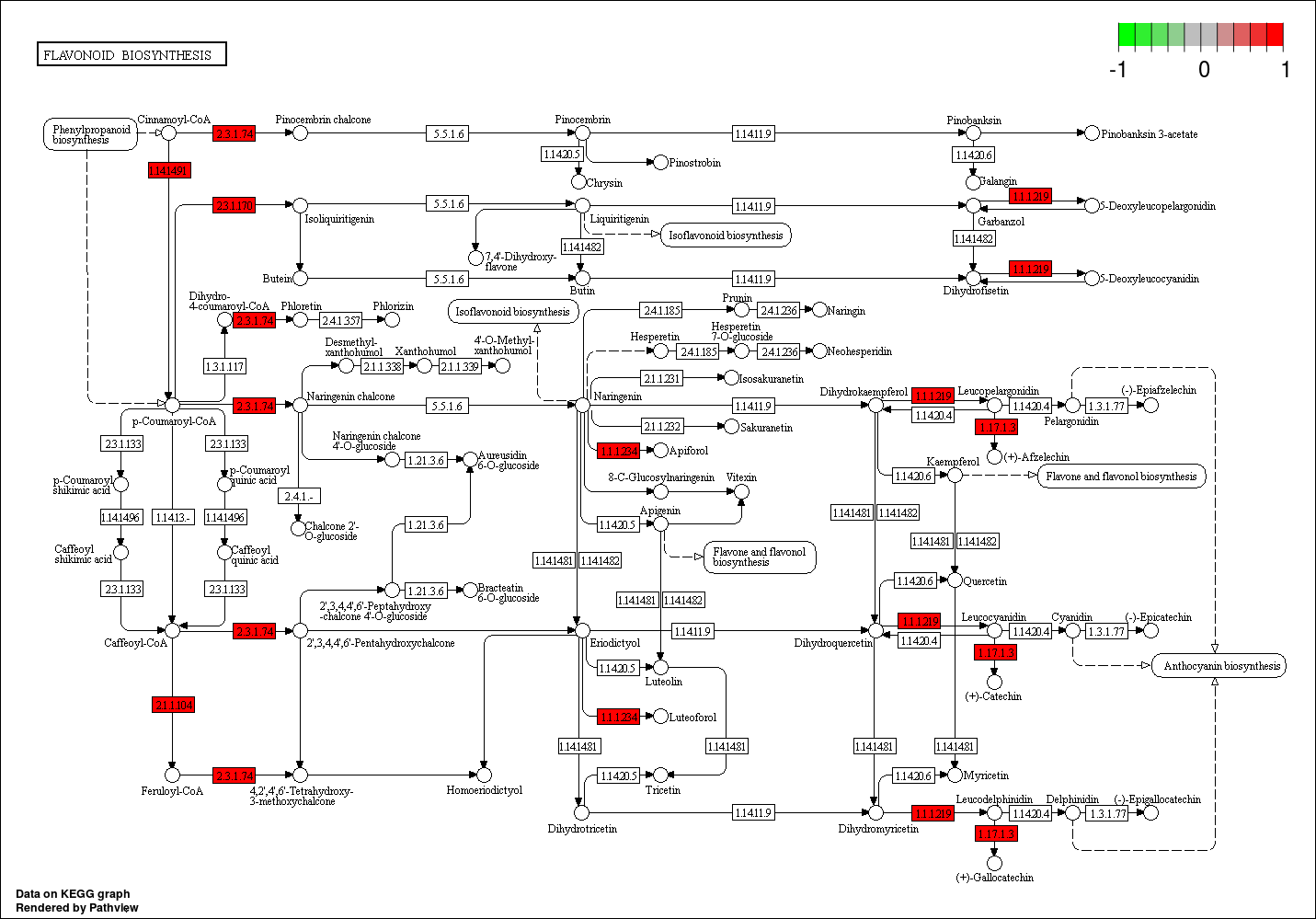

Supplement: Supplementary file 3 [file DataSheet_3.zip › Pathview_results/pmum00941.pathview.png]

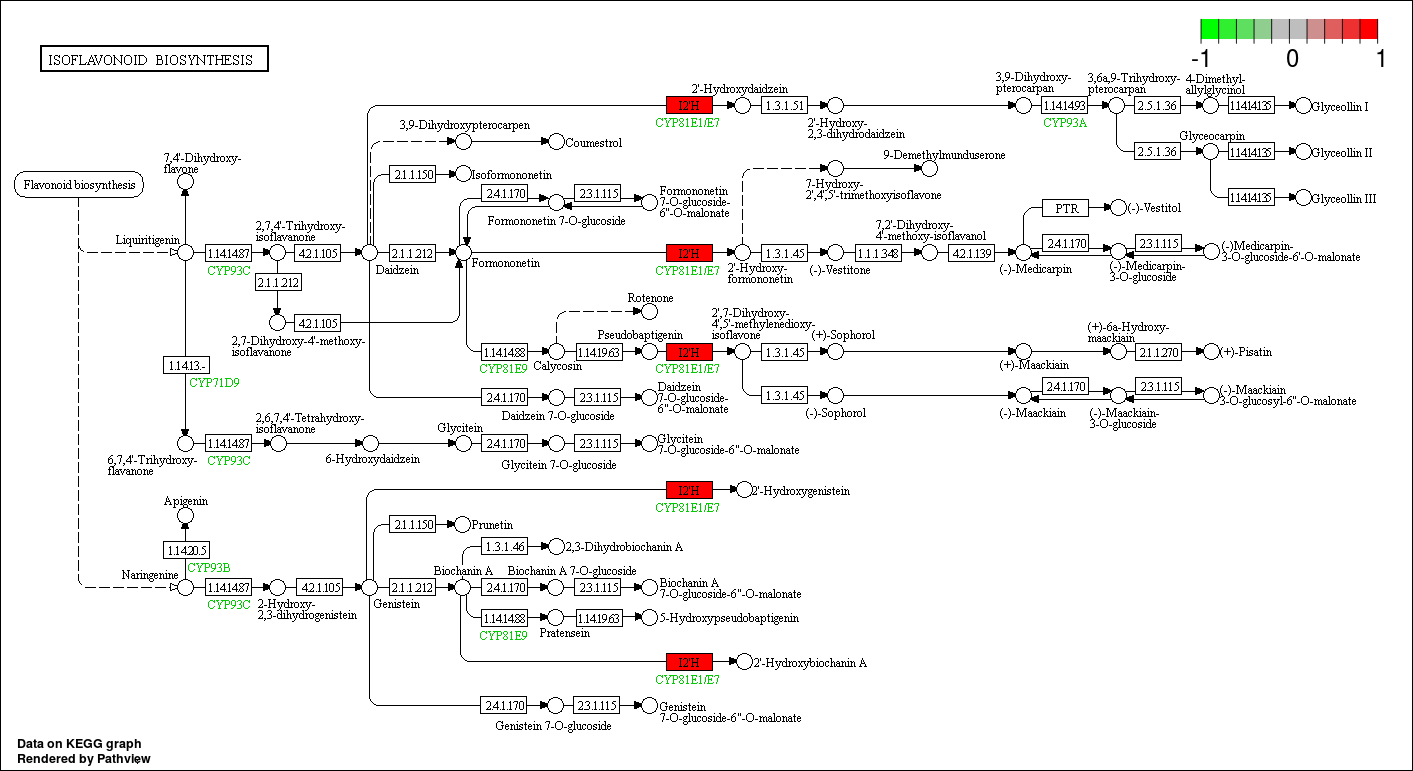

Supplement: Supplementary file 3 [file DataSheet_3.zip › Pathview_results/pmum00943.pathview.png]

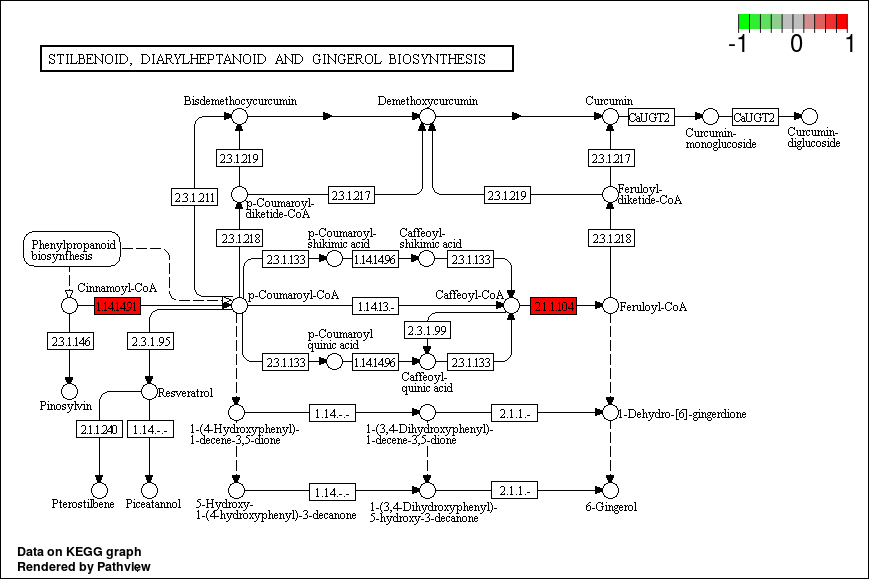

Supplement: Supplementary file 3 [file DataSheet_3.zip › Pathview_results/pmum00945.pathview.png]

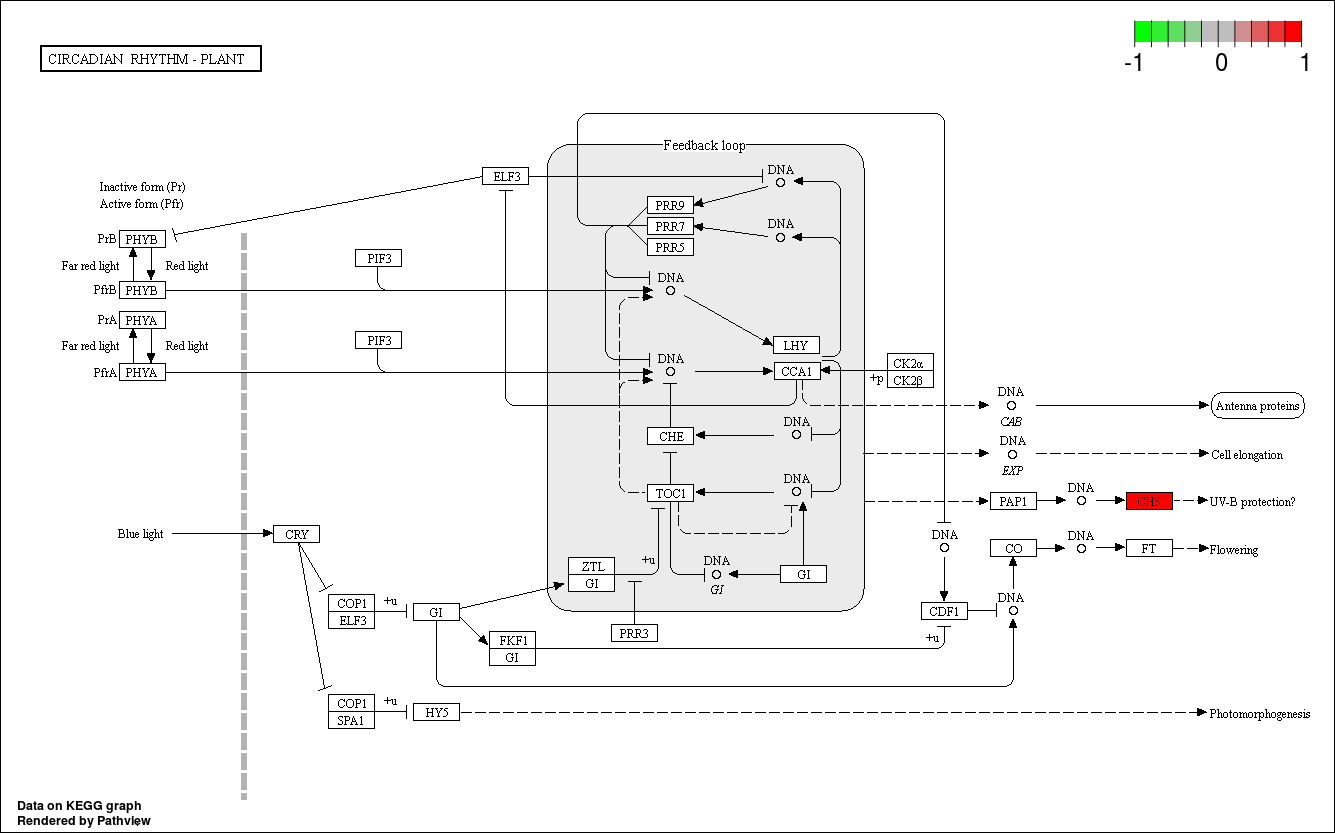

Supplement: Supplementary file 3 [file DataSheet_3.zip › Pathview_results/pmum04712.pathview.png]

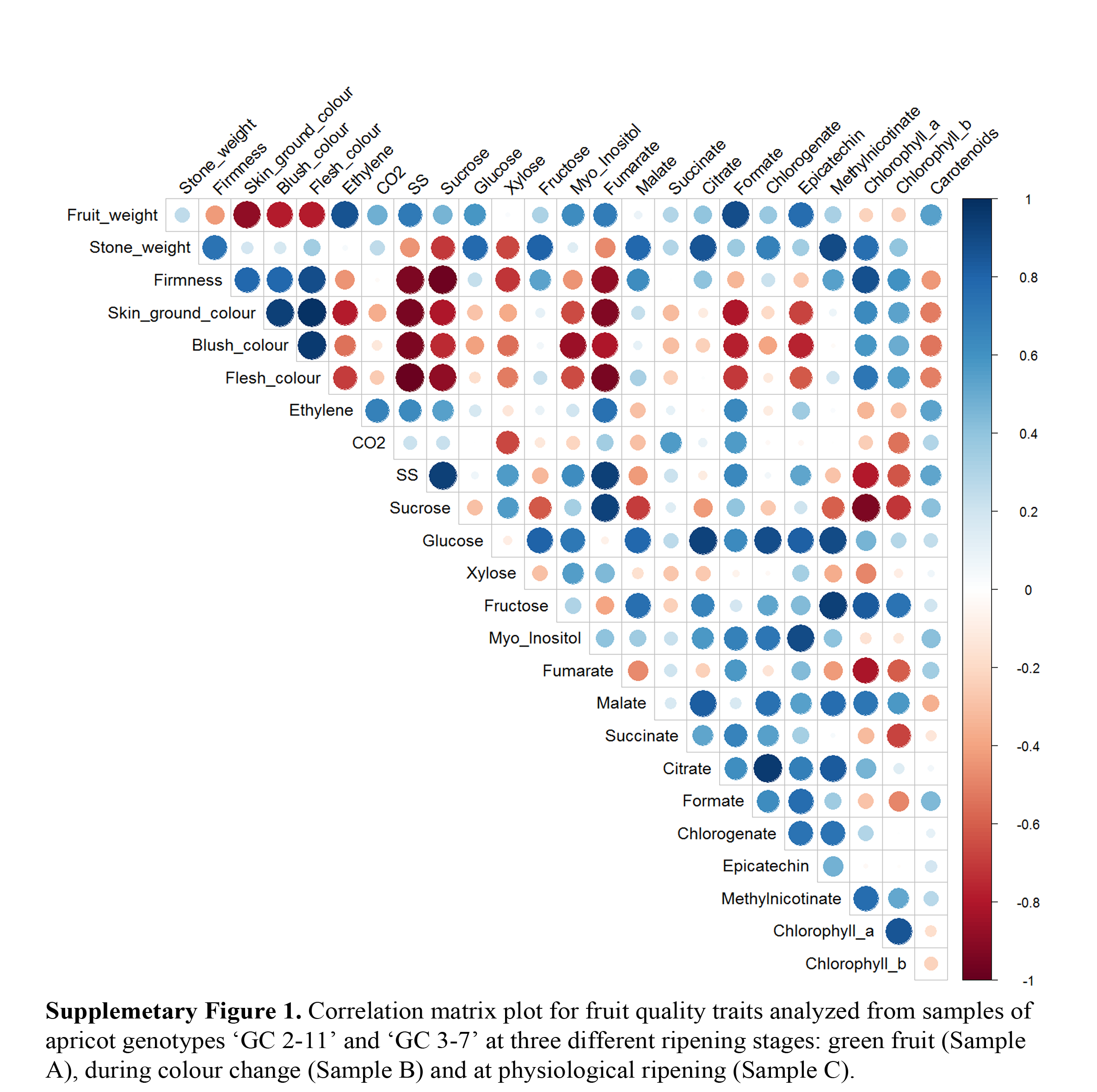

Supplement: Supplementary file 4 [file Image_1.tif]

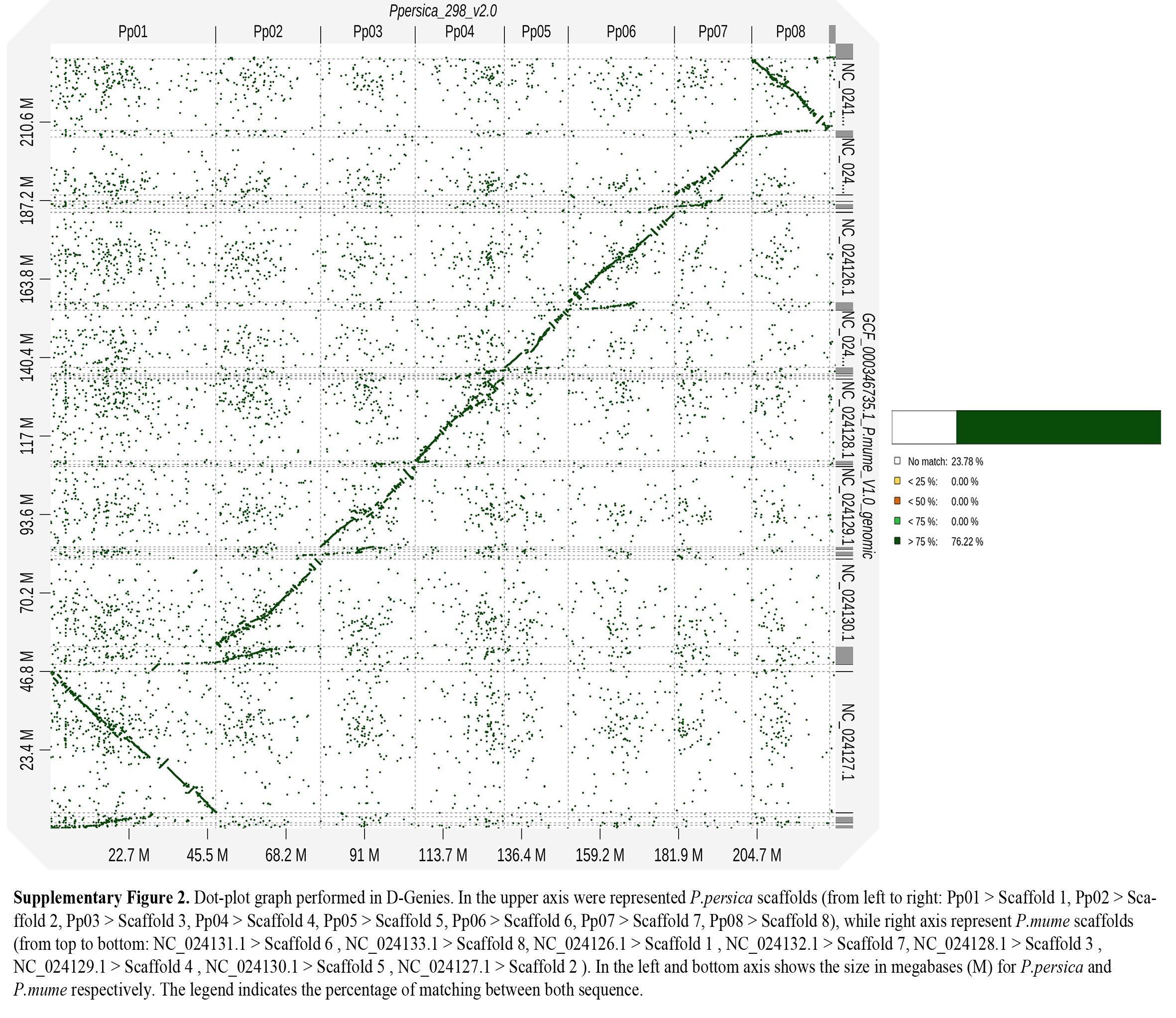

Supplement: Supplementary file 5 [file Image_2.tif]

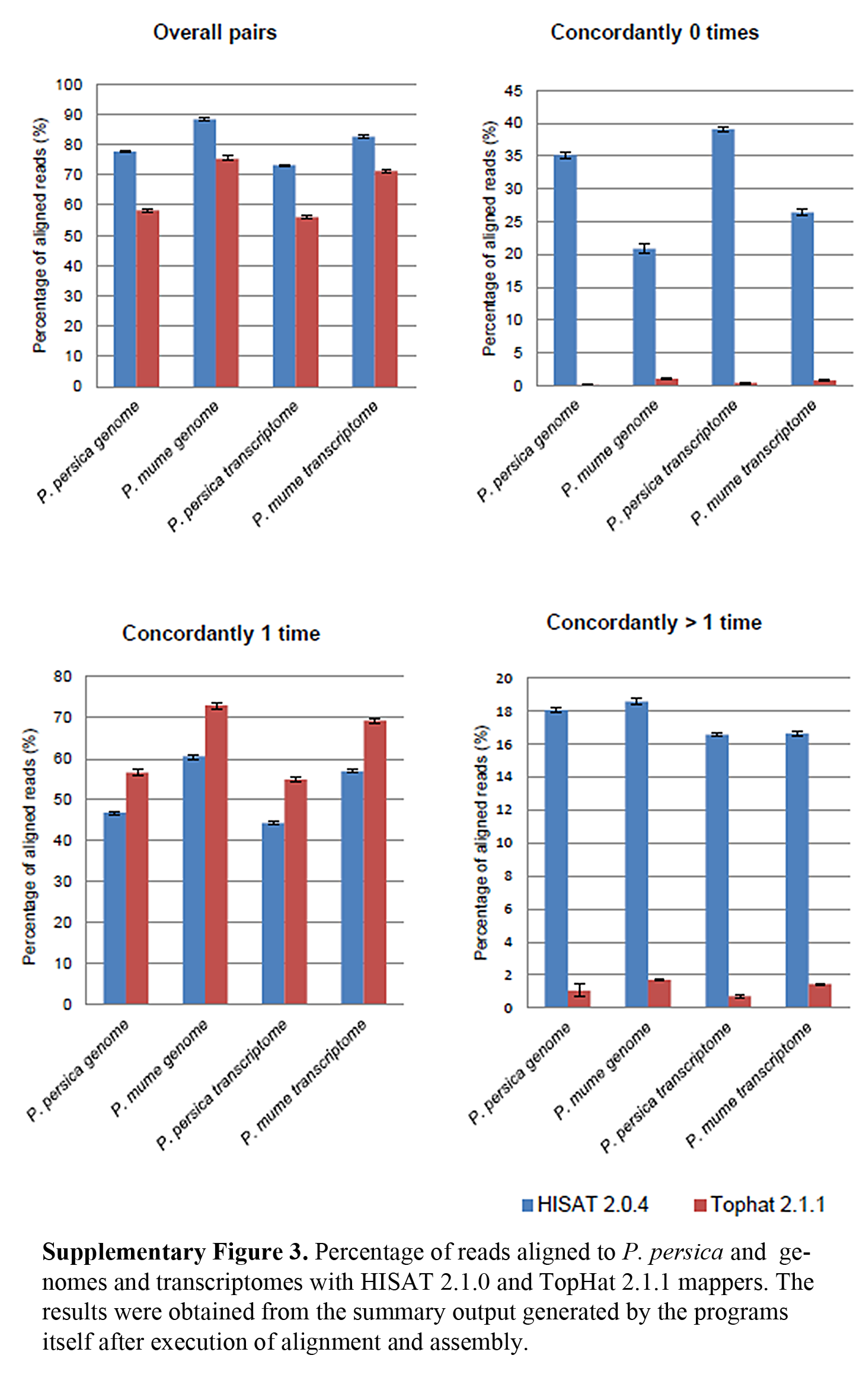

Supplement: Supplementary file 6 [file Image_3.tif]

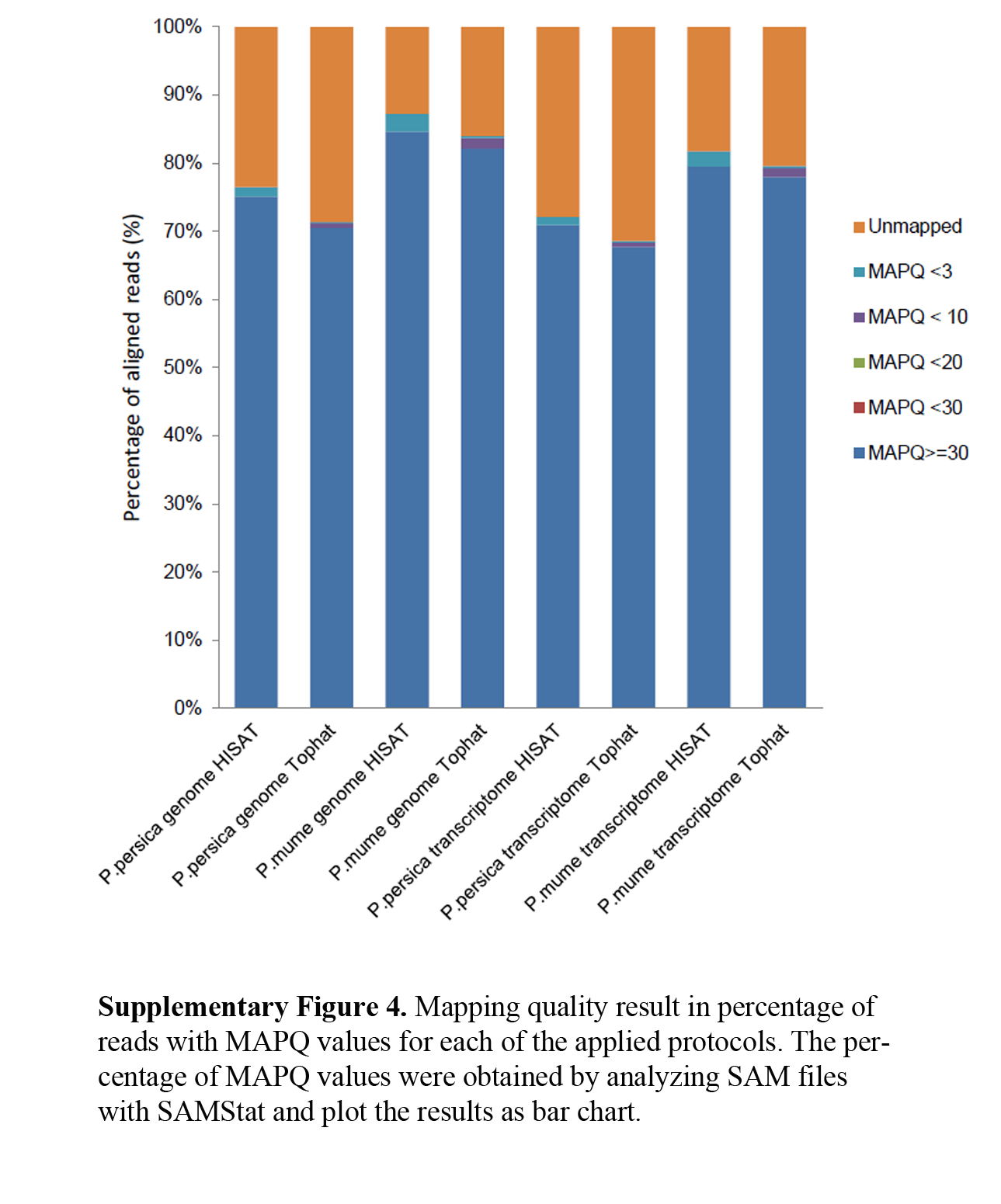

Supplement: Supplementary file 7 [file Image_4.tif]

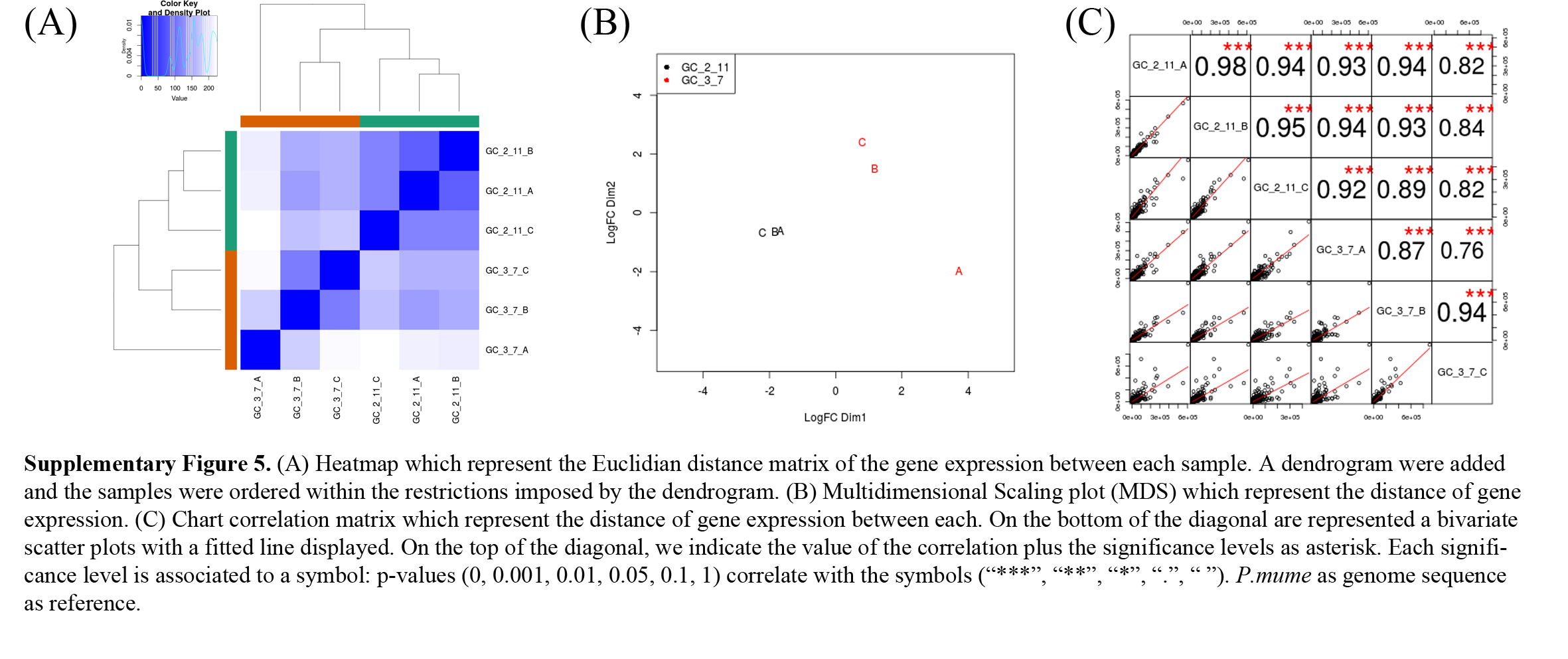

Supplement: Supplementary file 8 [file Image_5.tif]

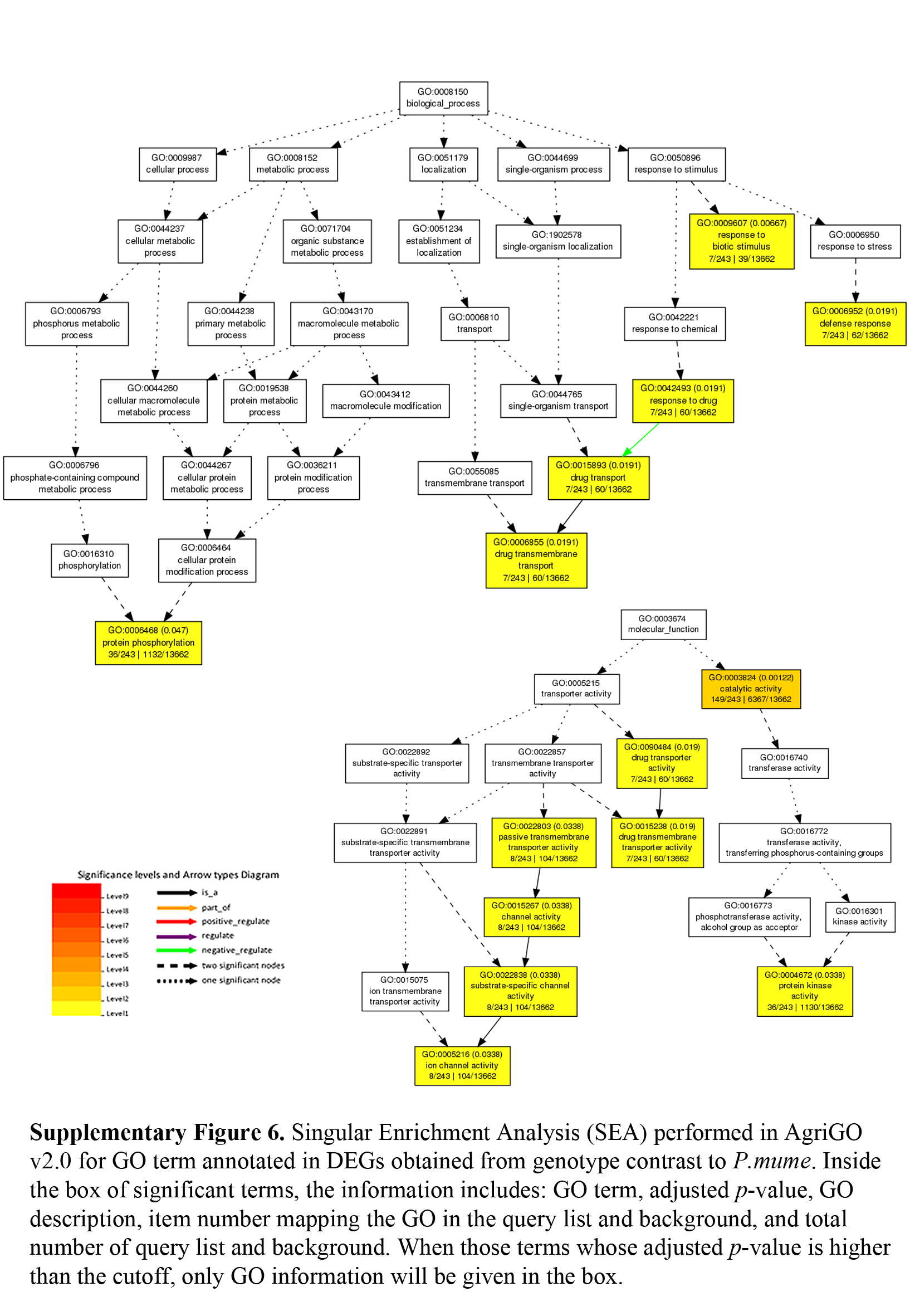

Supplement: Supplementary file 9 [file Image_6.tif]

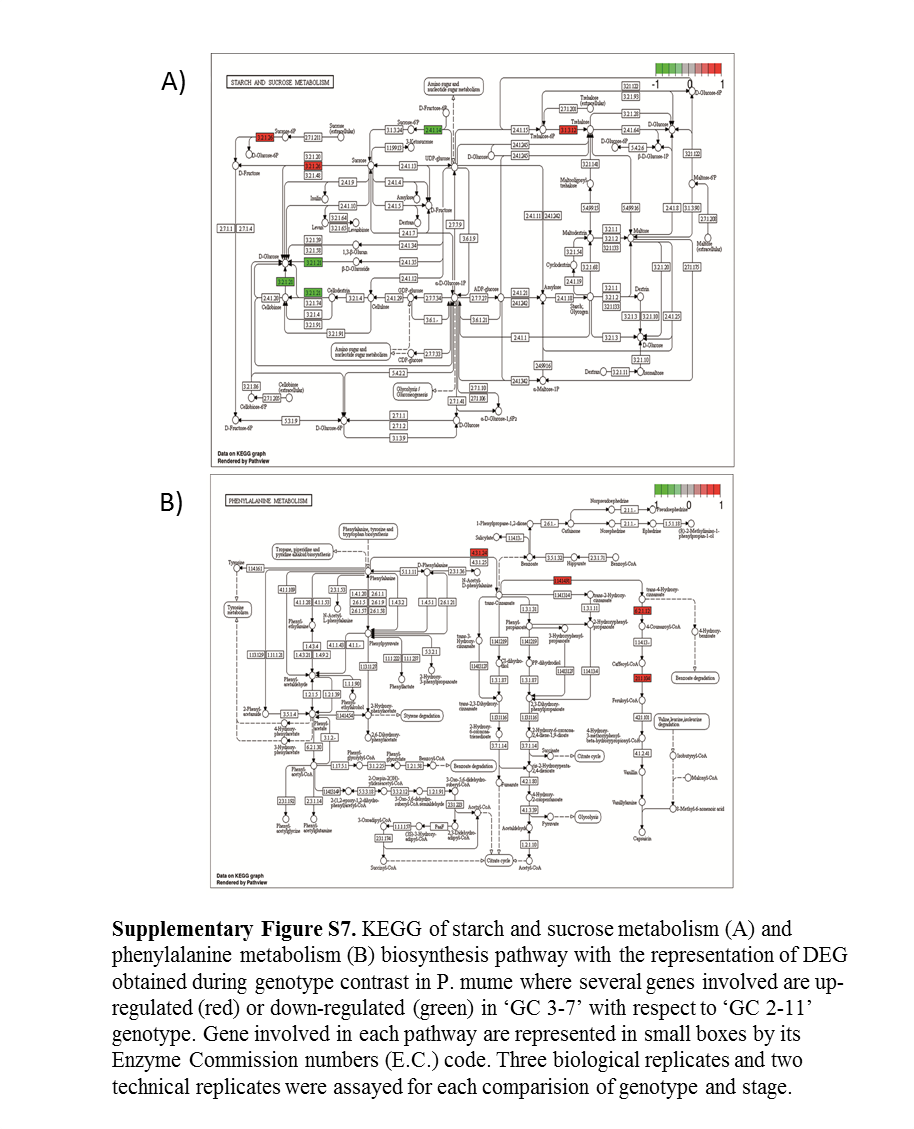

Supplement: Supplementary file 10 [file Image_7.tif]
