# Supplementary material for: Analysis of Metabolites and Gene Expression Changes Relative to Apricot (Prunus armeniaca L.) Fruit Quality During Development and Ripening
Source: Front Plant Sci. 2020 Aug 19;11:1269. doi: 10.3389/fpls.2020.01269 (PMC7466674; doi:10.3389/fpls.2020.01269)
Supplement: Supplementary file 11 [file Table_1.docx]

# SUPPLEMENTARY TABLES

Table S1. Kruskal Wallis Rank Sum Test *p*-value of fruit pomological characteristics and biochemical comntents in the two apricot genotypes ‘GC 2-11’ and ‘GC 3-7’ at three ripening stages: green fruit (Stage A), during colour change (Stage B) and at physiological ripening (Stage C); grouping the stages by genotype or ripening stages.

| Stages | Fruit weight | Stone weight | Skin ground colour | Blush colour | Flesh colour | Firmness | Soluble solids | Titratable acidity | CO_2_ | Ethylene | Chlorophyll *a* | Chlorophyll *b* | Carotenoids |
| --- | --- | --- | --- | --- | --- | --- | --- | --- | --- | --- | --- | --- | --- |
| Genotype | 1.86e-07 | 3.902e-07 | 6.622e-06 | 0.000152 | 0.0004556 | 0.1614 | 0.3265 | 0.03051 | 0.0815 | 0.02094 | 0.3309 | 0.9296 | 0.2004 |
| Stage A – Stage B | 0.1288 | 0.02179 | 2.2e-16 | 1.791e-08 | 2.2e-16 | 2.968e-07 | 0.003948 | 0.5218 | 0.179 | 0.0006986 | 0.003885 | 0.07765 | 0.01041 |
| Stage B – Stage C | 0.1348 | 0.142 | 2.482e-07 | 0.6919 | 2.054e-08 | 4.918e-07 | 0.0528 | 0.1093 | 0.3514 | 0.0008637 | 0.003823 | 0.2607 | 0.2002 |
| Stage A – Stage C | 0.001337 | 6.391e-05 | 2.2e-16 | 7.167e-10 | 2.2e-16 | 7.33e-08 | 0.0027 | 0.01631 | 0.5694 | 0.0001908 | 0.003885 | 0.2615 | 0.1495 |

Table S2. Kruskal Wallis Rank Sum Test *p*-value of metabolite contents recorded in ^1^H-NMR spectra in the two apricot genotypes ‘GC 2_11’ and ‘GC 3_7’ at three ripening stages: green fruit (Stage A), during colour change (Stage B) and at physiological ripening (Stage C); grouping the stages by genotype or ripening stage.

| Stages | Sucrose | Glucose | Xylose | Fructose | *Myo*-Inositol | Fumarate | Malate | Succinate | Citrate | Formate | Chlorogenate | Epicatechin | Methyl nicotinate |
| --- | --- | --- | --- | --- | --- | --- | --- | --- | --- | --- | --- | --- | --- |
| Genotype | 0.6911 | 0.0003486 | 0.3538 | 0.0003486 | 0.1223 | 0.7573 | 0.03798 | 0.2332 | 0.001268 | 0.007077 | 0.0004868 | 0.07027 | 0.04694 |
| Stage A – Stage B | 0.003948 | 0.631 | 0.01041 | 0.7488 | 0.2002 | 0.006485 | 0.2002 | 0.631 | 0.631 | 0.4233 | 0.631 | 0.5218 | 0.7488 |
| Stage B – Stage C | 0.003948 | 1 | 0.3367 | 0.5218 | 0.8728 | 0.01041 | 0.8728 | 0.3367 | 1 | 0.8728 | 0.8728 | 0.631 | 0.631 |
| Stage A – Stage C | 0.003948 | 1 | 0.07817 | 1 | 0.1093 | 0.003948 | 0.1495 | 0.8728 | 0.1093 | 0.2623 | 0.8728 | 0.2623 | 0.5218 |

Table S3. Mapping summary to genomic sequences of *P.persica* and *P.mume* as reference sequences, from stages of the two apricot genotypes assayed ‘GC 2-11’ and ‘GC 3-7’ assayed at three ripening stages: green fruit (Stage A), during colour change (Stage B) and at physiological ripening (Stage C). For mapping were used the software HISAT version 2.1.0 and TopHat version 2.1.1.

| Stages | Total paired reads sequnced | Mapped paired reads to *P. persica* genome with HISAT 2.1.0 (%) | Mapped paired reads to *P. mume* genome with HISAT 2.1.0 (%) | Mapped paired reads to *P. persica* genome with TopHat 2.1.1 (%) | Mapped paired reads to *P. mume* genome with TopHat 2.1.1 (%) |
| --- | --- | --- | --- | --- | --- |
| ‘GC 2-11’ Stage A | 51001371 | 39883072 (78.2) | 45207615 (88.64) | 30154261 (59.1) | 38845264 (76.16) |
| ‘GC 2-11’ Stage B | 55951202 | 43434918 (77.6) | 49416101 (88.32) | 32459397 (58.0) | 42151643 (75.33) |
| ‘GC 2-11’ Stage C | 48814261 | 38026309 (77.9) | 43171332 (88.44) | 28545080 (58.4) | 36886742 (75.56) |
| ‘GC 3-7’ Stage A | 55335050 | 42674390 (77.12) | 48777846 (88.15) | 31936954 (57.7) | 41539807 (75.06) |
| ‘GC 3-7’ Stage B | 48778187 | 38066497 (78.4) | 43168695 (88.5) | 28705178 (58.8) | 36926589 (75.70) |
| ‘GC 3-7’ stage C | 50629133 | 39181886 (77.7) | 44629580 (88.15) | 29068744 (57.4) | 37869701 (74.79) |
| Average | 51751534 ± 3154986 | 40211178 ± 2323803 (77.71 ± 0.41) | 45728528 ± 2737428  (88.36 ± 0.19) | 30144935,67 ± 1694625  (58.26 ± 0.66) | 39036624 ± 2299645 (75.15 ± 0.48) |

Table S4. Mapping summary to transcriptomic sequences of *P.persica* and *P.mume* as references, from stages of *P.armeniaca* genotypes ‘GC 2-11’ and ‘GC 3-7’ assayed at three ripening stages: green fruit (Stage A), during colour change (Stage B) and at physiological ripening (Stage C). For mapping were used the software HISAT version 2.1.0 and TopHat version 2.1.1.

| Stages | Total paired reads sequenced | Mapped paired reads to *P. persica* transcriptome with HISAT 2.1.0 (%) | Mapped paired reads to *P. mume* transcriptome with HISAT 2.1.0 (%) | Mapped paired reads to *P. persica* transcriptome with TopHat 2.1.1 (%) | Mapped paired reads to *P. mume* transcriptome with TopHat 2.1.1 (%) |
| --- | --- | --- | --- | --- | --- |
| ‘GC 2-11’ Stage A | 51001371 | 37720613 (73.96) | 42448441 (83.23) | 29166458 (57.18) | 36835067 (72.22) |
| ‘GC 2-11’ Stage B | 55951202 | 40905923 (73.11) | 46226883 (82.62) | 31303715 (55.94) | 39886191 (71.28) |
| ‘GC 2-11’ Stage C | 48814261 | 35858956 (73.46) | 40481666 (82.93) | 27539267 (56.41) | 35004242 (71.70) |
| ‘GC 3-7’ Stage A | 55335050 | 39791434 (71.91) | 45430076 (82.10) | 30460118 (55.04) | 39091063 (70.64) |
| ‘GC 3-7’ Stage B | 48778187 | 35856845 (73.51) | 40441994 (82.91) | 27504629 (56.38) | 34850269 (71.44) |
| ‘GC 3-7’ Stage C | 50629133 | 36954204 (72.99) | 41713342 (82.39) | 27978365 (55.26) | 35739054 (70.58) |
| Average | 51751534 ± 3154986 | 37847996 ± 2091120 (73.15 ± 0.69) | 42790400 ± 2485942  (82.69 ± 0.40) | 28992092 ± 1604991 (56.04 ± 0.79) | 36900981 ± 2138475 (71.31 ± 0.62) |

Table S5. High-quality reads aligned (MAPQ ≥ 30) to genomic sequences of *P.persica* and *P.mume* as reference sequences, from stages of *P. armeniaca* genotypes ‘GC 2-11’ and ‘GC 3-7’ assayed at three ripening stages: green fruit (Stage A), during colour change (Stage B) and at physiological ripening (Stage C). For mapping were used the software HISAT version 2.1.0 and TopHat version 2.1.1.

| Stages | Total reads sequenced | Mapped reads to *P. persica* genome with HISAT 2.1.0 (%) | Mapped reads to *P. mume* genome with HISAT 2.1.0 (%) | Mapped reads to *P. persica* genome with TopHat 2.1.1 (%) | Mapped reads to *P. mume* genome with TopHat 2.1.1 (%) |
| --- | --- | --- | --- | --- | --- |
| ‘GC 2-11’ Stage A | 102002744 | 77048192 (75.53) | 86534656 (84.83) | 72113064 (70.69) | 84235192 (82.58) |
| ‘GC 2-11’ Stage B | 111902400 | 83985232 (75.05) | 94662512 (84.59) | 78346032 (70.01) | 91928064 (82.15) |
| ‘GC 2-11’ Stage C | 97628520 | 73359008 (75.14) | 82587032 (84.59) | 68530880 (70.19) | 80265832 (82.21) |
| ‘GC 3-7’ Stage A | 110670096 | 82561024 (74.60) | 93387512 (84.38) | 76440240 (69.07) | 90449880 (81.72) |
| ‘GC 3-7’ Stage B | 97556376 | 73574824 (75.41) | 82670344 (84.74) | 68236048 (69.94) | 80242536 (82.25) |
| ‘GC 3-7’ Stage C | 101258264 | 75779280 (74.83) | 85655680 (84.59) | 70343608 (69.46) | 83012720 (81.98) |
| Average | 103503066 ± 6309970 | 77717926 ± 4541080 (75.09 ± 0.34) | 87582956 ± 5248224 (84.62 ± 0.15) | 72334978 ± 4201740 (69.89 ± 0.56) | 85022370 ± 5045320 (82.15 ± 0.28) |

Table S6. High-quality reads aligned (MAPQ ≥ 30) to transcriptomic sequence of *P.persica* and *P.mume* as reference sequence, from stages of the two apricot genotypes assayed ‘GC 2-11’ and ‘GC 3-7’ assayed at three ripening stages: green fruit (Stage A), during colour change (Stage B) and at physiological ripening (Stage C). For mapping were used the software HISAT version 2.1.0 and TopHat version 2.1.1.

| Stages | Total reads sequenced | Mapped reads to *P. persica* transcriptome with HISAT 2.1.0 (%) | Mapped reads to *P. mume* transcriptome with HISAT 2.1.0 (%) | Mapped reads to *P. persica* transcriptome with TopHat 2.1.1 (%) | Mapped reads to *P. mume* transcriptome with TopHat 2.1.1 (%) |
| --- | --- | --- | --- | --- | --- |
| ‘GC 2-11’ Stage A | 102002744 | 73124368 (71.68) | 81549024 (79.94) | 69921232 (68.54) | 80166400 (78.59) |
| ‘GC 2-11’ Stage B | 111902400 | 79368376 (70.92) | 88876232 (79.42) | 75776488 (67.71) | 87304080 (78.01) |
| ‘GC 2-11’ Stage C | 97628520 | 69423496 (71.10) | 77712656 (79.60) | 66395996 (68.00) | 76460032 (78.31) |
| ‘GC 3-7’ Stage A | 110670096 | 77247912 (69.80) | 87299448 (78.88) | 73781008 (66.66) | 85512104 (77.26) |
| ‘GC 3-7’ Stage B | 97556376 | 69539368 (71.28) | 77736344 (79.68) | 66312872 (67.97) | 76140080 (78.04) |
| ‘GC 3-7’ Stage C | 101258264 | 71710376 (70.81) | 80384760 (79.38) | 68184208 (67.33) | 78681016 (77.70) |
| Average | 103503066 ± 6309970 | 73402316 ± 4100628 (70.93 ± 0.63) | 82259744 ± 4781932 (79.48 ± 0.35) | 70061967 ± 3937711 (67.70 ± 0.64) | 80710618 ± 4688299 (77.99 ± 0.46) |

Table S7. Status of mapped reads in count matrix obtained with featureCounts to the genomic sequence of *P.mume* as reference sequence, from stages of the two apricot genotypes assayed ‘GC 2-11’ and ‘GC 3-7’ assayed at three ripening stages: green fruit (Stage A), during colour change (Stage B) and at physiological ripening (Stage C).

| Status | ‘GC 2-11’ Stage A | ‘GC 2-11’ Stage B | ‘GC 2-11’ Stage C | ‘GC 3-7’ Stage A | ‘GC 3-7’ Stage B | ‘GC 3-7’ Stage C |
| --- | --- | --- | --- | --- | --- | --- |
| Assigned | 44515217 | 48749833 | 42577325 | 47803281 | 42415792 | 44140803 |
| Unassigned: Unmapped | 0 | 0 | 0 | 0 | 0 | 0 |
| Unassigned: Mapping Quality | 0 | 0 | 0 | 0 | 0 | 0 |
| Unassigned: Chimera | 0 | 0 | 0 | 0 | 0 | 0 |
| Unassigned: Fragment Length | 0 | 0 | 0 | 0 | 0 | 0 |
| Unassigned: Duplicate | 0 | 0 | 0 | 0 | 0 | 0 |
| Unassigned: Multi-mapping | 0 | 0 | 0 | 0 | 0 | 0 |
| Unassigned: Secondary | 0 | 0 | 0 | 0 | 0 | 0 |
| Unassigned: Non-junction | 0 | 0 | 0 | 0 | 0 | 0 |
| Unassigned: No Features | 1905173 | 2191654 | 1852443 | 2436018 | 2030426 | 2071320 |
| Unassigned: Overlapping | 0 | 0 | 0 | 0 | 0 | 0 |
| Unassigned: Ambiguity | 0 | 0 | 0 | 0 | 0 | 0 |

Table S8. Euclidian distance matrix of gene expression logarithmically transformed using genomic sequence of *P.mume* as reference sequence, from stages of the two apricot genotypes assayed ‘GC 2-11’ and ‘GC 3-7’ assayed at three ripening stages: green fruit (Stage A), during colour change (Stage B) and at physiological ripening (Stage C).

|  | ‘GC 2-11’ Stage A | ‘GC 2-11’ Stage B | ‘GC 2-11’ Stage C | ‘GC 3-7’ Stage A | ‘GC 3-7’ Stage B | ‘GC 3-7’ Stage C |
| --- | --- | --- | --- | --- | --- | --- |
| ‘GC 2-11’ Stage A | 0 | 85.16127 | 115.1622 | 210.6454 | 138.0264 | 156.5388 |
| ‘GC 2-11’ Stage B | 85.16127 | 0 | 115.4561 | 209.0652 | 152.9137 | 156.8313 |
| ‘GC 2-11’ Stage C | 115.1622 | 115.4561 | 0 | 225.033 | 169.3074 | 179.7776 |
| ‘GC 3-7’ Stage A | 210.6454 | 209.0652 | 225.033 | 0 | 180.4259 | 219.8954 |
| ‘GC 3-7’ Stage B | 138.0264 | 152.9137 | 169.3074 | 180.4259 | 0 | 108.4069 |
| ‘GC 3-7’ Stage C | 156.5388 | 156.8313 | 179.7776 | 219.8954 | 108.4069 | 0 |

Table S9. DEGs for each stastical packages applied with genomic sequence of *P.mume* as reference sequence, from stages of *P. armeniaca* genotypes ‘GC 2-11’ and ‘GC 3-7’ assayed at three ripening stages: green fruit (Stage A), during colour change (Stage B) and at physiological ripening (Stage C). Statistical significance threshold was setting in FDR ≤ 0.1. Table 11.a represent the stages group by genotype and ripening stage. Table 11.b shows contrast between stages without replicates.

**Table 9.a**

| Group contrast | EBSeq | NOISeq | edgeR | baySeq | DESeq2 | Consensus |
| --- | --- | --- | --- | --- | --- | --- |
| Genotype | 1392 | 10873 | 1604 | 2150 | 1733 | 443 |
| Stage A – Stage B | 203 | 8953 | 2 | 5703 | 11 | 1 |
| Stage B – Stage C | 275 | 4267 | 4 | 104 | 14 | 0 |
| Stage A – Stage C | 828 | 14194 | 1678 | 5877 | 175 | 96 |

**Table 9.b**

| Stage contrast | EBSeq | NOISeq | edgeR | baySeq | DESeq2 | Consensus |
| --- | --- | --- | --- | --- | --- | --- |
| ‘GC 2-11’ Stage A – ‘GC 3-7’ Stage A | 2707 | 3449 | 21598 | 218 | 506 | 89 |
| ‘GC 2-11’ Stage B – ‘GC 3-7’ Stage B | 2810 | 2149 | 5823 | 12948 | 269 | 260 |
| ‘GC 2-11’ Stage C – ‘GC 3-7’ Stage C | 2751 | 3448 | 9343 | 15264 | 397 | 382 |
| ‘GC 2-11’ Stage A – ‘GC 2-11’ Stage B | 1680 | 751 | 20512 | 7565 | 43 | 43 |
| ‘GC 2-11’ Stage B – ‘GC 2-11’ Stage C | 2137 | 1761 | 5391 | 12469 | 95 | 95 |
| ‘GC 2-11’ Stage A – ‘GC 2-11’ Stage C | 1920 | 1484 | 20437 | 12150 | 68 | 67 |
| ‘GC 3-7’ Stage A – ‘GC 3-7’ Stage B | 2811 | 3258 | 8855 | 14908 | 495 | 494 |
| ‘GC 3-7’ Stage B – ‘GC 3-7’ Stage C | 1832 | 1150 | 3789 | 9885 | 32 | 32 |
| ‘GC 3-7’ Stage A – ‘GC 3-7’ Stage C | 2797 | 4515 | 11188 | 16793 | 1012 | 995 |

Supplementary Table 10. Summary GO annotation terms of DEGs up- and down regulated from genotype contrast in *P.mume.*

|  | | Up-regulated DEGs | Down-regulated DEGs | Total DEGs |
| --- | --- | --- | --- | --- |
| Genes | | 129 | 314 | 443 |
| Annotates genes | | 67 | 179 | 246 |
| GO Terms | Biological process | 43 | 124 | 167 |
|  | Cellular component | 15 | 36 | 51 |
|  | Molecular function | 65 | 167 | 232 |
|  | Total | 123 | 327 | 450 |
