# Supplementary material for: Analysis of Metabolites and Gene Expression Changes Relative to Apricot (Prunus armeniaca L.) Fruit Quality During Development and Ripening
Source: Front Plant Sci. 2020 Aug 19;11:1269. doi: 10.3389/fpls.2020.01269 (PMC7466674; doi:10.3389/fpls.2020.01269)
Supplement: Supplementary file 1 [file DataSheet_1.zip › FastQC_raw/D_S4_L001_R2_001_fastqc/fastqc_report.html]

D\_S4\_L001\_R2\_001.fastq FastQC Report


FastQC Report

dom 3 jun 2018  
D\_S4\_L001\_R2\_001.fastq

## Summary

- Basic Statistics
- Per base sequence quality
- Per sequence quality scores
- Per base sequence content
- Per base GC content
- Per sequence GC content
- Per base N content
- Sequence Length Distribution
- Sequence Duplication Levels
- Overrepresented sequences
- Kmer Content

## Basic Statistics

| Measure | Value |
| --- | --- |
| Filename | D\_S4\_L001\_R2\_001.fastq |
| File type | Conventional base calls |
| Encoding | Sanger / Illumina 1.9 |
| Total Sequences | 27913080 |
| Filtered Sequences | 0 |
| Sequence length | 101 |
| %GC | 45 |

## Per base sequence quality

## Per sequence quality scores

## Per base sequence content

## Per base GC content

## Per sequence GC content

## Per base N content

## Sequence Length Distribution

## Sequence Duplication Levels

## Overrepresented sequences

| Sequence | Count | Percentage | Possible Source |
| --- | --- | --- | --- |
| NNNNNNNNNNNNNNNNNNNNNNNNNNNNNNNNNNNNNNNNNNNNNNNNNN | 43551 | 0.15602362763263675 | No Hit |

## Kmer Content

| Sequence | Count | Obs/Exp Overall | Obs/Exp Max | Max Obs/Exp Position |
| --- | --- | --- | --- | --- |
| CTCTC | 7253450 | 3.7186036 | 6.349911 | 1 |
| TCTCT | 8086825 | 3.217282 | 5.3220425 | 7 |
| GAAGA | 9948995 | 3.1154475 | 7.851364 | 2 |
| TCTTC | 6802660 | 2.706387 | 5.2923365 | 7 |
| CTTCT | 6463735 | 2.571548 | 6.396483 | 1 |
| GAGAA | 7191455 | 2.2519464 | 5.262095 | 2 |
| GGAAG | 6122275 | 2.2251406 | 5.8516345 | 1 |
| CTTCA | 5610375 | 2.2185915 | 7.8677464 | 1 |
| CTCCA | 3964780 | 2.0203598 | 5.609052 | 1 |
| CCCAA | 3899835 | 1.9752868 | 5.203462 | 1 |
| CTCTG | 4266520 | 1.958225 | 5.3531065 | 1 |
| TTCAA | 6012015 | 1.8338158 | 5.217889 | 2 |
| CTCAA | 4661690 | 1.8323278 | 6.989685 | 1 |
| CTTTG | 5098235 | 1.815869 | 5.7841144 | 1 |
| TCCAA | 4596235 | 1.8066001 | 5.437032 | 7 |
| GAAAA | 6543145 | 1.7653241 | 5.6770396 | 2 |
| GGAAA | 5401265 | 1.6913627 | 5.1808195 | 1 |
| CTTGG | 4031545 | 1.6565855 | 5.1698313 | 1 |
| CTCAG | 3528915 | 1.6099201 | 5.883223 | 1 |
| CTTGA | 4408580 | 1.5607657 | 5.9661736 | 1 |

Produced by FastQC (version 0.10.1)
