# Supplementary material for: Analysis of Metabolites and Gene Expression Changes Relative to Apricot (Prunus armeniaca L.) Fruit Quality During Development and Ripening
Source: Front Plant Sci. 2020 Aug 19;11:1269. doi: 10.3389/fpls.2020.01269 (PMC7466674; doi:10.3389/fpls.2020.01269)
Supplement: Supplementary file 1 [file DataSheet_1.zip › FastQC_raw/D_S4_L002_R1_001_fastqc/fastqc_report.html]

D\_S4\_L002\_R1\_001.fastq FastQC Report


FastQC Report

dom 3 jun 2018  
D\_S4\_L002\_R1\_001.fastq

## Summary

- Basic Statistics
- Per base sequence quality
- Per sequence quality scores
- Per base sequence content
- Per base GC content
- Per sequence GC content
- Per base N content
- Sequence Length Distribution
- Sequence Duplication Levels
- Overrepresented sequences
- Kmer Content

## Basic Statistics

| Measure | Value |
| --- | --- |
| Filename | D\_S4\_L002\_R1\_001.fastq |
| File type | Conventional base calls |
| Encoding | Sanger / Illumina 1.9 |
| Total Sequences | 27421970 |
| Filtered Sequences | 0 |
| Sequence length | 101 |
| %GC | 44 |

## Per base sequence quality

## Per sequence quality scores

## Per base sequence content

## Per base GC content

## Per sequence GC content

## Per base N content

## Sequence Length Distribution

## Sequence Duplication Levels

## Overrepresented sequences

No overrepresented sequences

## Kmer Content

| Sequence | Count | Obs/Exp Overall | Obs/Exp Max | Max Obs/Exp Position |
| --- | --- | --- | --- | --- |
| TCTTC | 9688820 | 2.850064 | 6.232095 | 7 |
| CTTCT | 9367025 | 2.7554047 | 5.7192874 | 1 |
| TTCTT | 9728650 | 2.5319948 | 5.69633 | 6 |
| CTTCA | 7939720 | 2.3630729 | 7.6641393 | 1 |
| CTTGG | 4654790 | 2.2357962 | 7.3389807 | 1 |
| CACCA | 6531520 | 2.2230427 | 6.455847 | 1 |
| TCCTC | 6600140 | 2.1943727 | 5.58694 | 2 |
| CTCCA | 6425405 | 2.1614532 | 13.886482 | 1 |
| CCTTG | 5379050 | 2.1495633 | 5.123127 | 1 |
| TCTTG | 5960140 | 2.107306 | 5.1995773 | 7 |
| CTTGA | 5715750 | 2.0447137 | 5.781341 | 1 |
| CTCTG | 4978900 | 1.9896563 | 9.60693 | 1 |
| CTTTG | 5621250 | 1.987486 | 5.3366737 | 1 |
| TCCTT | 6652325 | 1.9568484 | 5.218888 | 2 |
| CTCCT | 5863880 | 1.9495853 | 10.042575 | 1 |
| CTGCA | 4631315 | 1.8725661 | 5.75576 | 1 |
| CTCTT | 6262835 | 1.842276 | 6.771194 | 1 |
| TCCAA | 5964830 | 1.7962142 | 6.492756 | 2 |
| GTTGG | 2894955 | 1.6713269 | 5.891018 | 1 |
| TTCAA | 6232495 | 1.660537 | 5.312664 | 7 |
| TCCAT | 5576745 | 1.6597881 | 5.6650777 | 2 |
| CCTCA | 4918210 | 1.6544453 | 5.6840487 | 1 |
| CTCTC | 4952870 | 1.6466987 | 5.303867 | 1 |
| CTCAG | 3955860 | 1.5994612 | 7.7711296 | 1 |
| CTCAA | 5097185 | 1.5349367 | 6.230792 | 1 |
| TCCAG | 3767225 | 1.523191 | 5.2828593 | 2 |
| GGCAG | 2287910 | 1.5104982 | 5.333387 | 1 |
| CTGGA | 3040990 | 1.4778668 | 5.3669424 | 1 |
| CCCAA | 4283665 | 1.4579716 | 6.6591105 | 1 |
| CTGGG | 2128550 | 1.388919 | 5.4074984 | 1 |
| CTCAT | 4525235 | 1.3468307 | 6.017178 | 1 |
| CCCAT | 3848785 | 1.2946997 | 5.9862094 | 1 |
| GCCAG | 2190795 | 1.20336 | 5.074935 | 1 |
| CCCCA | 3158240 | 1.2007817 | 5.9237375 | 1 |
| CCCAG | 2626725 | 1.200386 | 6.4719286 | 1 |
| GTCCA | 2840330 | 1.1484224 | 7.2159514 | 1 |
| CTCCC | 2838885 | 1.0667889 | 5.1205482 | 1 |
| GTCCT | 2649695 | 1.0588648 | 5.821047 | 1 |
| CTCGG | 1508810 | 0.8191061 | 5.220896 | 1 |
| CTCCG | 1752225 | 0.7914218 | 6.092376 | 1 |

Produced by FastQC (version 0.10.1)
