# Supplementary material for: Analysis of Metabolites and Gene Expression Changes Relative to Apricot (Prunus armeniaca L.) Fruit Quality During Development and Ripening
Source: Front Plant Sci. 2020 Aug 19;11:1269. doi: 10.3389/fpls.2020.01269 (PMC7466674; doi:10.3389/fpls.2020.01269)
Supplement: Supplementary file 1 [file DataSheet_1.zip › FastQC_raw/D_S4_L002_R2_001_fastqc/fastqc_report.html]

D\_S4\_L002\_R2\_001.fastq FastQC Report


FastQC Report

dom 3 jun 2018  
D\_S4\_L002\_R2\_001.fastq

## Summary

- Basic Statistics
- Per base sequence quality
- Per sequence quality scores
- Per base sequence content
- Per base GC content
- Per sequence GC content
- Per base N content
- Sequence Length Distribution
- Sequence Duplication Levels
- Overrepresented sequences
- Kmer Content

## Basic Statistics

| Measure | Value |
| --- | --- |
| Filename | D\_S4\_L002\_R2\_001.fastq |
| File type | Conventional base calls |
| Encoding | Sanger / Illumina 1.9 |
| Total Sequences | 27421970 |
| Filtered Sequences | 0 |
| Sequence length | 101 |
| %GC | 45 |

## Per base sequence quality

## Per sequence quality scores

## Per base sequence content

## Per base GC content

## Per sequence GC content

## Per base N content

## Sequence Length Distribution

## Sequence Duplication Levels

## Overrepresented sequences

| Sequence | Count | Percentage | Possible Source |
| --- | --- | --- | --- |
| NNNNNNNNNNNNNNNNNNNNNNNNNNNNNNNNNNNNNNNNNNNNNNNNNN | 45214 | 0.1648823917464719 | No Hit |

## Kmer Content

| Sequence | Count | Obs/Exp Overall | Obs/Exp Max | Max Obs/Exp Position |
| --- | --- | --- | --- | --- |
| CTCTC | 7131675 | 3.7212284 | 6.3364162 | 1 |
| TCTCT | 7940175 | 3.2183158 | 5.3444424 | 7 |
| GAAGA | 9797190 | 3.121206 | 7.8826723 | 2 |
| TCTTC | 6671645 | 2.7041547 | 5.2772145 | 7 |
| CTTCT | 6341220 | 2.5702267 | 6.4901814 | 1 |
| GAGAA | 7069730 | 2.2522871 | 5.2132654 | 2 |
| GGAAG | 6013545 | 2.2223763 | 5.852859 | 1 |
| CTTCA | 5517735 | 2.2219582 | 7.8349013 | 1 |
| CTCCA | 3902055 | 2.0228546 | 5.671252 | 1 |
| CCCAA | 3845630 | 1.9806839 | 5.2586164 | 1 |
| CTCTG | 4193360 | 1.9588598 | 5.3533225 | 1 |
| TTCAA | 5919485 | 1.8396677 | 5.1741614 | 2 |
| CTCAA | 4580335 | 1.8325202 | 6.979236 | 1 |
| CTTTG | 5015695 | 1.8200201 | 5.86213 | 1 |
| TCCAA | 4518830 | 1.8079131 | 5.4921355 | 7 |
| GAAAA | 6432755 | 1.7666551 | 5.6576896 | 2 |
| GGAAA | 5314665 | 1.6931554 | 5.1006618 | 1 |
| CTTGG | 3975525 | 1.6625783 | 5.1702366 | 1 |
| CTCAG | 3469800 | 1.6103569 | 5.8336716 | 1 |
| CTTGA | 4337900 | 1.5638715 | 5.9458966 | 1 |

Produced by FastQC (version 0.10.1)
