# Supplementary material for: Analysis of Metabolites and Gene Expression Changes Relative to Apricot (Prunus armeniaca L.) Fruit Quality During Development and Ripening
Source: Front Plant Sci. 2020 Aug 19;11:1269. doi: 10.3389/fpls.2020.01269 (PMC7466674; doi:10.3389/fpls.2020.01269)
Supplement: Supplementary file 1 [file DataSheet_1.zip › FastQC_raw/E_S5_L001_R1_001_fastqc/fastqc_report.html]

E\_S5\_L001\_R1\_001.fastq FastQC Report


FastQC Report

lun 4 jun 2018  
E\_S5\_L001\_R1\_001.fastq

## Summary

- Basic Statistics
- Per base sequence quality
- Per sequence quality scores
- Per base sequence content
- Per base GC content
- Per sequence GC content
- Per base N content
- Sequence Length Distribution
- Sequence Duplication Levels
- Overrepresented sequences
- Kmer Content

## Basic Statistics

| Measure | Value |
| --- | --- |
| Filename | E\_S5\_L001\_R1\_001.fastq |
| File type | Conventional base calls |
| Encoding | Sanger / Illumina 1.9 |
| Total Sequences | 24590062 |
| Filtered Sequences | 0 |
| Sequence length | 101 |
| %GC | 45 |

## Per base sequence quality

## Per sequence quality scores

## Per base sequence content

## Per base GC content

## Per sequence GC content

## Per base N content

## Sequence Length Distribution

## Sequence Duplication Levels

## Overrepresented sequences

No overrepresented sequences

## Kmer Content

| Sequence | Count | Obs/Exp Overall | Obs/Exp Max | Max Obs/Exp Position |
| --- | --- | --- | --- | --- |
| TCTTC | 8863695 | 2.9000907 | 6.355798 | 7 |
| CTTCT | 8555125 | 2.7991304 | 5.729062 | 1 |
| TTCTT | 8598715 | 2.5076032 | 5.5804114 | 6 |
| CTTCA | 7121045 | 2.369748 | 7.702225 | 1 |
| CACCA | 6031710 | 2.2905095 | 6.228557 | 1 |
| TCCTC | 6103480 | 2.240504 | 5.8083606 | 2 |
| CTTGG | 4194760 | 2.2129967 | 7.5197735 | 1 |
| CCTTG | 4958005 | 2.1818652 | 5.13117 | 1 |
| CTCCA | 5814640 | 2.1709645 | 14.225448 | 1 |
| CTCTG | 4789285 | 2.1076167 | 10.490767 | 1 |
| TCTTG | 5338110 | 2.093809 | 5.1844954 | 7 |
| CTTGA | 5182950 | 2.0677035 | 5.823033 | 1 |
| CTTTG | 5111330 | 2.004857 | 5.4147124 | 1 |
| CTCCT | 5402915 | 1.9833361 | 10.184203 | 1 |
| TCCTT | 6001585 | 1.9636439 | 5.2179837 | 2 |
| CTGCA | 4196860 | 1.8784823 | 5.673121 | 1 |
| CTCTT | 5741200 | 1.8784492 | 6.9296536 | 1 |
| TCCAA | 5351825 | 1.8114322 | 6.62191 | 2 |
| CCTCA | 4596620 | 1.7162024 | 5.7864814 | 1 |
| GTTGG | 2631890 | 1.6645397 | 6.1057005 | 1 |
| TCCAT | 4982670 | 1.6581377 | 6.004032 | 2 |
| TTCAA | 5455020 | 1.6456788 | 5.1605783 | 7 |
| CTCAG | 3651750 | 1.6344954 | 7.795625 | 1 |
| CTCTC | 4402180 | 1.61598 | 5.3377233 | 1 |
| CTCAA | 4638090 | 1.5698544 | 6.340287 | 1 |
| GGCAG | 2097130 | 1.5135086 | 5.422995 | 1 |
| CCCAA | 3957410 | 1.502805 | 6.6218634 | 1 |
| TCCAG | 3346515 | 1.4978745 | 5.326191 | 2 |
| CTGGA | 2773025 | 1.4879528 | 5.207533 | 1 |
| CCATT | 4324965 | 1.439266 | 5.10772 | 3 |
| CTCAT | 4092510 | 1.3619094 | 6.095562 | 1 |
| CCCAT | 3458165 | 1.2911465 | 6.1554103 | 1 |
| GCCAG | 2034130 | 1.2245753 | 5.0693817 | 1 |
| CCCCA | 2909585 | 1.2187997 | 6.0617175 | 1 |
| CCCAG | 2360085 | 1.1851727 | 6.326157 | 1 |
| GTCCA | 2489370 | 1.1142229 | 7.2742352 | 1 |
| GTCCT | 2419405 | 1.0647055 | 6.0348353 | 1 |
| CTCCG | 1590835 | 0.7854481 | 5.952996 | 1 |

Produced by FastQC (version 0.10.1)
