# Supplementary material for: Analysis of Metabolites and Gene Expression Changes Relative to Apricot (Prunus armeniaca L.) Fruit Quality During Development and Ripening
Source: Front Plant Sci. 2020 Aug 19;11:1269. doi: 10.3389/fpls.2020.01269 (PMC7466674; doi:10.3389/fpls.2020.01269)
Supplement: Supplementary file 1 [file DataSheet_1.zip › FastQC_raw/E_S5_L001_R2_001_fastqc/fastqc_report.html]

E\_S5\_L001\_R2\_001.fastq FastQC Report


FastQC Report

lun 4 jun 2018  
E\_S5\_L001\_R2\_001.fastq

## Summary

- Basic Statistics
- Per base sequence quality
- Per sequence quality scores
- Per base sequence content
- Per base GC content
- Per sequence GC content
- Per base N content
- Sequence Length Distribution
- Sequence Duplication Levels
- Overrepresented sequences
- Kmer Content

## Basic Statistics

| Measure | Value |
| --- | --- |
| Filename | E\_S5\_L001\_R2\_001.fastq |
| File type | Conventional base calls |
| Encoding | Sanger / Illumina 1.9 |
| Total Sequences | 24590062 |
| Filtered Sequences | 0 |
| Sequence length | 101 |
| %GC | 45 |

## Per base sequence quality

## Per sequence quality scores

## Per base sequence content

## Per base GC content

## Per sequence GC content

## Per base N content

## Sequence Length Distribution

## Sequence Duplication Levels

## Overrepresented sequences

| Sequence | Count | Percentage | Possible Source |
| --- | --- | --- | --- |
| NNNNNNNNNNNNNNNNNNNNNNNNNNNNNNNNNNNNNNNNNNNNNNNNNN | 38301 | 0.15575804566901866 | No Hit |

## Kmer Content

| Sequence | Count | Obs/Exp Overall | Obs/Exp Max | Max Obs/Exp Position |
| --- | --- | --- | --- | --- |
| CTCTC | 6419945 | 3.7065115 | 6.2132096 | 1 |
| TCTCT | 7077030 | 3.2187846 | 5.351895 | 7 |
| GAAGA | 8821950 | 3.1285326 | 8.135241 | 2 |
| TCTTC | 6032105 | 2.74353 | 5.3624797 | 7 |
| CTTCT | 5588395 | 2.5417216 | 6.157881 | 1 |
| GAGAA | 6515020 | 2.310425 | 5.3822036 | 2 |
| GGAAG | 5417850 | 2.2188697 | 5.798442 | 1 |
| CTTCA | 4843070 | 2.1765792 | 7.920143 | 1 |
| CCCAA | 3625735 | 2.0438821 | 5.1659884 | 1 |
| CTCCA | 3463985 | 1.9761635 | 5.4840665 | 1 |
| CTCTG | 3733215 | 1.9376056 | 5.3090606 | 1 |
| CAACA | 4255240 | 1.8672545 | 5.1311216 | 1 |
| TTCAA | 5329415 | 1.8644596 | 5.279166 | 2 |
| CTTTG | 4391815 | 1.7956977 | 5.7248254 | 1 |
| CTCAA | 4035205 | 1.7919762 | 6.702153 | 1 |
| TCCAA | 3992060 | 1.7728161 | 5.125012 | 7 |
| GAAAA | 5700295 | 1.7504303 | 5.6943655 | 2 |
| CTTGG | 3591660 | 1.6758145 | 5.1373806 | 1 |
| GGAAA | 4667835 | 1.655357 | 5.0857735 | 1 |
| CTCAG | 3098200 | 1.5889298 | 5.679444 | 1 |
| CTTGA | 3870305 | 1.5636778 | 6.144121 | 1 |

Produced by FastQC (version 0.10.1)
