# Supplementary material for: Analysis of Metabolites and Gene Expression Changes Relative to Apricot (Prunus armeniaca L.) Fruit Quality During Development and Ripening
Source: Front Plant Sci. 2020 Aug 19;11:1269. doi: 10.3389/fpls.2020.01269 (PMC7466674; doi:10.3389/fpls.2020.01269)
Supplement: Supplementary file 1 [file DataSheet_1.zip › FastQC_raw/E_S5_L002_R1_001_fastqc/fastqc_report.html]

E\_S5\_L002\_R1\_001.fastq FastQC Report


FastQC Report

lun 4 jun 2018  
E\_S5\_L002\_R1\_001.fastq

## Summary

- Basic Statistics
- Per base sequence quality
- Per sequence quality scores
- Per base sequence content
- Per base GC content
- Per sequence GC content
- Per base N content
- Sequence Length Distribution
- Sequence Duplication Levels
- Overrepresented sequences
- Kmer Content

## Basic Statistics

| Measure | Value |
| --- | --- |
| Filename | E\_S5\_L002\_R1\_001.fastq |
| File type | Conventional base calls |
| Encoding | Sanger / Illumina 1.9 |
| Total Sequences | 24188125 |
| Filtered Sequences | 0 |
| Sequence length | 101 |
| %GC | 45 |

## Per base sequence quality

## Per sequence quality scores

## Per base sequence content

## Per base GC content

## Per sequence GC content

## Per base N content

## Sequence Length Distribution

## Sequence Duplication Levels

## Overrepresented sequences

No overrepresented sequences

## Kmer Content

| Sequence | Count | Obs/Exp Overall | Obs/Exp Max | Max Obs/Exp Position |
| --- | --- | --- | --- | --- |
| TCTTC | 8727335 | 2.8998928 | 6.3576646 | 7 |
| CTTCT | 8423260 | 2.7988553 | 5.7900677 | 1 |
| TTCTT | 8466435 | 2.5076752 | 5.604845 | 6 |
| CTTCA | 7017970 | 2.3734975 | 7.738832 | 1 |
| CACCA | 5942730 | 2.2949324 | 6.1633945 | 1 |
| TCCTC | 6007420 | 2.2393286 | 5.8925543 | 2 |
| CTTGG | 4127350 | 2.2113798 | 7.5036488 | 1 |
| CCTTG | 4875145 | 2.178705 | 5.155675 | 1 |
| CTCCA | 5731500 | 2.174579 | 14.304761 | 1 |
| CTCTG | 4705695 | 2.1029778 | 10.459113 | 1 |
| TCTTG | 5262180 | 2.0962694 | 5.236668 | 7 |
| CTTGA | 5106795 | 2.0706506 | 5.9146094 | 1 |
| CTTTG | 5031680 | 2.0044463 | 5.4417453 | 1 |
| CTCCT | 5331635 | 1.9874226 | 10.292549 | 1 |
| TCCTT | 5910275 | 1.9638482 | 5.241477 | 2 |
| CTCTT | 5660885 | 1.8809817 | 6.975184 | 1 |
| CTGCA | 4122505 | 1.8752068 | 5.643607 | 1 |
| TCCAA | 5264945 | 1.8123753 | 6.578726 | 2 |
| CCTCT | 4795210 | 1.7874646 | 5.043943 | 3 |
| CCTCA | 4515595 | 1.7132543 | 5.756794 | 1 |
| GTTGG | 2595595 | 1.667286 | 6.140231 | 1 |
| TCCAT | 4898545 | 1.6567017 | 6.0442033 | 2 |
| TTCAA | 5365315 | 1.6463416 | 5.1897807 | 7 |
| CTCAG | 3590080 | 1.6330222 | 7.819644 | 1 |
| CTCTC | 4343585 | 1.6191167 | 5.3916883 | 1 |
| CTCAA | 4558660 | 1.5692476 | 6.311305 | 1 |
| GGCAG | 2063110 | 1.5132204 | 5.4250484 | 1 |
| CCCAA | 3899160 | 1.5057572 | 6.6347613 | 1 |
| TCCAG | 3290685 | 1.4968361 | 5.3228645 | 2 |
| CTGGA | 2729955 | 1.4887596 | 5.148309 | 1 |
| CCATT | 4254360 | 1.4388366 | 5.08547 | 3 |
| CTCAT | 4032310 | 1.3637387 | 6.160654 | 1 |
| CCCAT | 3398655 | 1.289478 | 6.190612 | 1 |
| GCCAG | 2002480 | 1.2250869 | 5.022279 | 1 |
| CCCCA | 2866700 | 1.2201655 | 6.123401 | 1 |
| CCCAG | 2322445 | 1.1851219 | 6.2303677 | 1 |
| GGGGG | 979285 | 1.1378988 | 5.1005955 | 1 |
| GTCCA | 2439180 | 1.1095115 | 7.2036934 | 1 |
| GTCCT | 2388120 | 1.0672523 | 5.951343 | 1 |
| CTCCG | 1566320 | 0.7852736 | 5.9337697 | 1 |

Produced by FastQC (version 0.10.1)
