# Supplementary material for: Analysis of Metabolites and Gene Expression Changes Relative to Apricot (Prunus armeniaca L.) Fruit Quality During Development and Ripening
Source: Front Plant Sci. 2020 Aug 19;11:1269. doi: 10.3389/fpls.2020.01269 (PMC7466674; doi:10.3389/fpls.2020.01269)
Supplement: Supplementary file 1 [file DataSheet_1.zip › FastQC_raw/E_S5_L002_R2_001_fastqc/fastqc_report.html]

E\_S5\_L002\_R2\_001.fastq FastQC Report


FastQC Report

mié 6 jun 2018  
E\_S5\_L002\_R2\_001.fastq

## Summary

- Basic Statistics
- Per base sequence quality
- Per sequence quality scores
- Per base sequence content
- Per base GC content
- Per sequence GC content
- Per base N content
- Sequence Length Distribution
- Sequence Duplication Levels
- Overrepresented sequences
- Kmer Content

## Basic Statistics

| Measure | Value |
| --- | --- |
| Filename | E\_S5\_L002\_R2\_001.fastq |
| File type | Conventional base calls |
| Encoding | Sanger / Illumina 1.9 |
| Total Sequences | 24188125 |
| Filtered Sequences | 0 |
| Sequence length | 101 |
| %GC | 45 |

## Per base sequence quality

## Per sequence quality scores

## Per base sequence content

## Per base GC content

## Per sequence GC content

## Per base N content

## Sequence Length Distribution

## Sequence Duplication Levels

## Overrepresented sequences

| Sequence | Count | Percentage | Possible Source |
| --- | --- | --- | --- |
| NNNNNNNNNNNNNNNNNNNNNNNNNNNNNNNNNNNNNNNNNNNNNNNNNN | 39729 | 0.16425001937934422 | No Hit |

## Kmer Content

| Sequence | Count | Obs/Exp Overall | Obs/Exp Max | Max Obs/Exp Position |
| --- | --- | --- | --- | --- |
| CTCTC | 6337995 | 3.717012 | 6.303376 | 1 |
| TCTCT | 6981445 | 3.2264063 | 5.3253293 | 7 |
| GAAGA | 8675400 | 3.129536 | 8.136725 | 2 |
| TCTTC | 5939990 | 2.745108 | 5.3970504 | 7 |
| CTTCT | 5507630 | 2.5452971 | 6.1742573 | 1 |
| GAGAA | 6397155 | 2.3076892 | 5.451795 | 2 |
| GGAAG | 5326220 | 2.2180073 | 5.823448 | 1 |
| CTTCA | 4762745 | 2.1755064 | 7.9422517 | 1 |
| CCCAA | 3575475 | 2.0484924 | 5.213494 | 1 |
| CTCCA | 3413100 | 1.9784286 | 5.5115542 | 1 |
| CTCTG | 3670920 | 1.9356703 | 5.194864 | 1 |
| TTCAA | 5245470 | 1.8661577 | 5.2366376 | 2 |
| CAACA | 4179240 | 1.8649143 | 5.0559864 | 1 |
| CTTTG | 4325090 | 1.7971456 | 5.6910825 | 1 |
| CTCAA | 3971195 | 1.7928895 | 6.735852 | 1 |
| TCCAA | 3930505 | 1.774519 | 5.088558 | 7 |
| GAAAA | 5588490 | 1.7463508 | 5.7190523 | 2 |
| CTTGG | 3525850 | 1.671609 | 5.079805 | 1 |
| GGAAA | 4587185 | 1.6547662 | 5.0815344 | 1 |
| CTCAG | 3040655 | 1.5847213 | 5.638333 | 1 |
| CTTGA | 3802515 | 1.5616667 | 6.1324024 | 1 |

Produced by FastQC (version 0.10.1)
