# Supplementary material for: Analysis of Metabolites and Gene Expression Changes Relative to Apricot (Prunus armeniaca L.) Fruit Quality During Development and Ripening
Source: Front Plant Sci. 2020 Aug 19;11:1269. doi: 10.3389/fpls.2020.01269 (PMC7466674; doi:10.3389/fpls.2020.01269)
Supplement: Supplementary file 1 [file DataSheet_1.zip › FastQC_raw/F_S6_L001_R1_001_fastqc/fastqc_report.html]

F\_S6\_L001\_R1\_001.fastq FastQC Report


FastQC Report

dom 3 jun 2018  
F\_S6\_L001\_R1\_001.fastq

## Summary

- Basic Statistics
- Per base sequence quality
- Per sequence quality scores
- Per base sequence content
- Per base GC content
- Per sequence GC content
- Per base N content
- Sequence Length Distribution
- Sequence Duplication Levels
- Overrepresented sequences
- Kmer Content

## Basic Statistics

| Measure | Value |
| --- | --- |
| Filename | F\_S6\_L001\_R1\_001.fastq |
| File type | Conventional base calls |
| Encoding | Sanger / Illumina 1.9 |
| Total Sequences | 25517216 |
| Filtered Sequences | 0 |
| Sequence length | 101 |
| %GC | 45 |

## Per base sequence quality

## Per sequence quality scores

## Per base sequence content

## Per base GC content

## Per sequence GC content

## Per base N content

## Sequence Length Distribution

## Sequence Duplication Levels

## Overrepresented sequences

No overrepresented sequences

## Kmer Content

| Sequence | Count | Obs/Exp Overall | Obs/Exp Max | Max Obs/Exp Position |
| --- | --- | --- | --- | --- |
| TCTTC | 9192740 | 2.8926404 | 6.299003 | 7 |
| CTTCT | 8929600 | 2.8098392 | 5.9266458 | 1 |
| TTCTT | 9046825 | 2.520566 | 5.5358233 | 6 |
| CTTCA | 7332585 | 2.354551 | 7.645383 | 1 |
| CCTTG | 5274740 | 2.2380986 | 5.2595167 | 1 |
| CACCA | 6044165 | 2.2368484 | 6.3211718 | 1 |
| CTCCA | 6156425 | 2.232684 | 14.521231 | 1 |
| TCCTC | 6275760 | 2.230301 | 5.8751273 | 2 |
| CTTGG | 4373935 | 2.2158046 | 7.6671314 | 1 |
| CTCTG | 5056895 | 2.145666 | 10.944485 | 1 |
| CTTGA | 5527055 | 2.118975 | 5.9647055 | 1 |
| TCTTG | 5619475 | 2.1111848 | 5.235037 | 7 |
| CTTTG | 5287450 | 1.986446 | 5.4021153 | 1 |
| CTCCT | 5532665 | 1.9662173 | 10.539639 | 1 |
| TCCTT | 6237955 | 1.962871 | 5.227781 | 2 |
| CTCTT | 5927590 | 1.8652098 | 6.973424 | 1 |
| TCCAA | 5617325 | 1.8406963 | 6.711161 | 2 |
| CTGCA | 4239750 | 1.835777 | 5.6068773 | 1 |
| CCTCT | 5068720 | 1.8013389 | 5.047543 | 3 |
| GTTGG | 2843795 | 1.7200395 | 5.905544 | 1 |
| CCTCA | 4738465 | 1.7184478 | 5.6963544 | 1 |
| TCCAT | 5255530 | 1.6875926 | 6.2098002 | 2 |
| CTCTC | 4663200 | 1.6572238 | 5.224177 | 1 |
| CTCAG | 3790065 | 1.6410673 | 8.001805 | 1 |
| TTCAA | 5550035 | 1.6102773 | 5.117447 | 7 |
| CTCAA | 4799420 | 1.5726836 | 6.452318 | 1 |
| CCCAA | 4129420 | 1.528232 | 7.0523763 | 1 |
| GGCAG | 2175795 | 1.5167266 | 5.2847757 | 1 |
| CCATT | 4633540 | 1.4878664 | 5.2673817 | 3 |
| TCCAG | 3403210 | 1.4735621 | 5.123452 | 2 |
| CTGGA | 2840710 | 1.4685462 | 5.3942094 | 1 |
| CTCAT | 4181570 | 1.3427355 | 6.286385 | 1 |
| CCCAT | 3678960 | 1.3342087 | 6.6625943 | 1 |
| CTGGG | 1898345 | 1.29677 | 5.0666385 | 1 |
| CCCCA | 3051120 | 1.2496995 | 6.4402227 | 1 |
| CCCAG | 2443545 | 1.1949443 | 6.4839005 | 1 |
| GTCCA | 2599440 | 1.1255362 | 7.0950084 | 1 |
| CTCCC | 2652480 | 1.0646256 | 5.2669764 | 1 |
| GTCCT | 2411935 | 1.0233961 | 5.6153297 | 1 |
| CTCGG | 1461155 | 0.8359938 | 5.225724 | 1 |
| CTCCG | 1634990 | 0.78350335 | 6.1457934 | 1 |

Produced by FastQC (version 0.10.1)
