# Supplementary material for: Analysis of Metabolites and Gene Expression Changes Relative to Apricot (Prunus armeniaca L.) Fruit Quality During Development and Ripening
Source: Front Plant Sci. 2020 Aug 19;11:1269. doi: 10.3389/fpls.2020.01269 (PMC7466674; doi:10.3389/fpls.2020.01269)
Supplement: Supplementary file 1 [file DataSheet_1.zip › FastQC_raw/F_S6_L001_R2_001_fastqc/fastqc_report.html]

F\_S6\_L001\_R2\_001.fastq FastQC Report


FastQC Report

dom 3 jun 2018  
F\_S6\_L001\_R2\_001.fastq

## Summary

- Basic Statistics
- Per base sequence quality
- Per sequence quality scores
- Per base sequence content
- Per base GC content
- Per sequence GC content
- Per base N content
- Sequence Length Distribution
- Sequence Duplication Levels
- Overrepresented sequences
- Kmer Content

## Basic Statistics

| Measure | Value |
| --- | --- |
| Filename | F\_S6\_L001\_R2\_001.fastq |
| File type | Conventional base calls |
| Encoding | Sanger / Illumina 1.9 |
| Total Sequences | 25517216 |
| Filtered Sequences | 0 |
| Sequence length | 101 |
| %GC | 45 |

## Per base sequence quality

## Per sequence quality scores

## Per base sequence content

## Per base GC content

## Per sequence GC content

## Per base N content

## Sequence Length Distribution

## Sequence Duplication Levels

## Overrepresented sequences

| Sequence | Count | Percentage | Possible Source |
| --- | --- | --- | --- |
| NNNNNNNNNNNNNNNNNNNNNNNNNNNNNNNNNNNNNNNNNNNNNNNNNN | 39993 | 0.15672948020661817 | No Hit |
| CGAGAAAATACAAAATGGGAGCTCAAACTTTCACAACCCCAACCTCAAAT | 29585 | 0.11594133153083785 | No Hit |

## Kmer Content

| Sequence | Count | Obs/Exp Overall | Obs/Exp Max | Max Obs/Exp Position |
| --- | --- | --- | --- | --- |
| CTCTC | 7338785 | 4.018587 | 6.559473 | 1 |
| TCTCT | 8133220 | 3.5129368 | 5.718089 | 7 |
| GAAGA | 9067930 | 3.1324677 | 7.982533 | 2 |
| TCTTC | 6493825 | 2.8048418 | 5.4552627 | 7 |
| CTTCT | 6037375 | 2.60769 | 6.735102 | 1 |
| GAGAA | 6897865 | 2.3828304 | 5.8682475 | 2 |
| GGAAG | 5565160 | 2.2444875 | 5.690392 | 1 |
| CCCAA | 4017820 | 2.149078 | 5.448568 | 1 |
| CTTCA | 5004220 | 2.136242 | 7.9080667 | 1 |
| CTCTG | 3892410 | 1.9399644 | 5.491296 | 1 |
| CAACA | 4635560 | 1.9329859 | 5.9542146 | 1 |
| CTCCA | 3558850 | 1.9260393 | 5.7341833 | 1 |
| TTCAA | 5668625 | 1.8864993 | 5.3516364 | 2 |
| CTCAA | 4276160 | 1.8041567 | 6.6927547 | 1 |
| CTTTG | 4557355 | 1.791623 | 5.855689 | 1 |
| GAAAA | 5926055 | 1.7534081 | 5.649836 | 2 |
| TCCAA | 4134950 | 1.7445787 | 5.06571 | 7 |
| CTTGG | 3725815 | 1.6901388 | 5.307914 | 1 |
| CTCAG | 3163600 | 1.5583427 | 5.734344 | 1 |
| CTTGA | 3941315 | 1.5313735 | 6.0616727 | 1 |

Produced by FastQC (version 0.10.1)
