# Supplementary material for: Analysis of Metabolites and Gene Expression Changes Relative to Apricot (Prunus armeniaca L.) Fruit Quality During Development and Ripening
Source: Front Plant Sci. 2020 Aug 19;11:1269. doi: 10.3389/fpls.2020.01269 (PMC7466674; doi:10.3389/fpls.2020.01269)
Supplement: Supplementary file 1 [file DataSheet_1.zip › FastQC_raw/F_S6_L002_R1_001_fastqc/fastqc_report.html]

F\_S6\_L002\_R1\_001.fastq FastQC Report


FastQC Report

dom 3 jun 2018  
F\_S6\_L002\_R1\_001.fastq

## Summary

- Basic Statistics
- Per base sequence quality
- Per sequence quality scores
- Per base sequence content
- Per base GC content
- Per sequence GC content
- Per base N content
- Sequence Length Distribution
- Sequence Duplication Levels
- Overrepresented sequences
- Kmer Content

## Basic Statistics

| Measure | Value |
| --- | --- |
| Filename | F\_S6\_L002\_R1\_001.fastq |
| File type | Conventional base calls |
| Encoding | Sanger / Illumina 1.9 |
| Total Sequences | 25111917 |
| Filtered Sequences | 0 |
| Sequence length | 101 |
| %GC | 45 |

## Per base sequence quality

## Per sequence quality scores

## Per base sequence content

## Per base GC content

## Per sequence GC content

## Per base N content

## Sequence Length Distribution

## Sequence Duplication Levels

## Overrepresented sequences

No overrepresented sequences

## Kmer Content

| Sequence | Count | Obs/Exp Overall | Obs/Exp Max | Max Obs/Exp Position |
| --- | --- | --- | --- | --- |
| TCTTC | 9058860 | 2.895517 | 6.317834 | 7 |
| CTTCT | 8818115 | 2.818567 | 5.977356 | 1 |
| TTCTT | 8922725 | 2.5262153 | 5.569773 | 6 |
| CTTCA | 7213445 | 2.3532374 | 7.6228247 | 1 |
| CCTTG | 5189880 | 2.2364514 | 5.3391232 | 1 |
| CACCA | 5946670 | 2.2353578 | 6.209586 | 1 |
| CTCCA | 6065505 | 2.233931 | 14.646301 | 1 |
| TCCTC | 6185575 | 2.232094 | 5.900266 | 2 |
| CTTGG | 4298980 | 2.2122664 | 7.7622814 | 1 |
| CTCTG | 4971965 | 2.1425464 | 10.961951 | 1 |
| CTTGA | 5436000 | 2.1177382 | 5.9728494 | 1 |
| TCTTG | 5535995 | 2.1130905 | 5.227664 | 7 |
| CTTTG | 5202760 | 1.9858947 | 5.3624716 | 1 |
| CTCCT | 5462445 | 1.9711492 | 10.601936 | 1 |
| TCCTT | 6146555 | 1.9646463 | 5.3057957 | 2 |
| CTCTT | 5849550 | 1.8697135 | 7.0562196 | 1 |
| CTGCA | 4177140 | 1.8371798 | 5.5695186 | 1 |
| TCCAA | 5517345 | 1.8370609 | 6.689401 | 2 |
| CCTCT | 4989180 | 1.8003693 | 5.037794 | 3 |
| CCTCA | 4679390 | 1.7234237 | 5.706026 | 1 |
| GTTGG | 2794200 | 1.7171147 | 5.9953194 | 1 |
| TCCAT | 5164270 | 1.6847365 | 6.2298036 | 2 |
| CTCTC | 4586060 | 1.6549015 | 5.2225657 | 1 |
| CTCAG | 3743770 | 1.6465759 | 8.025427 | 1 |
| TTCAA | 5469305 | 1.6130425 | 5.139126 | 7 |
| CTCAA | 4733950 | 1.5762209 | 6.4905734 | 1 |
| CCCAA | 4068205 | 1.5292413 | 7.085615 | 1 |
| GGCAG | 2145545 | 1.5192512 | 5.3177786 | 1 |
| CCATT | 4561895 | 1.488224 | 5.249306 | 3 |
| TCCAG | 3348805 | 1.4728633 | 5.1583 | 2 |
| CTGGA | 2783230 | 1.461812 | 5.344113 | 1 |
| CTCAT | 4121045 | 1.3444058 | 6.317376 | 1 |
| CCCAT | 3621995 | 1.333984 | 6.6659107 | 1 |
| CTGGG | 1870795 | 1.2979196 | 5.0997815 | 1 |
| CCCCA | 3018455 | 1.2550682 | 6.435231 | 1 |
| CCCAG | 2404770 | 1.1940594 | 6.49125 | 1 |
| GTCCA | 2555170 | 1.1238087 | 7.0335402 | 1 |
| CTCCC | 2619835 | 1.067299 | 5.2821274 | 1 |
| GTCCT | 2384475 | 1.0275311 | 5.6927085 | 1 |
| CTCGG | 1442115 | 0.83782184 | 5.2078466 | 1 |
| CTCCG | 1603785 | 0.7802399 | 6.134674 | 1 |

Produced by FastQC (version 0.10.1)
