# Supplementary material for: Analysis of Metabolites and Gene Expression Changes Relative to Apricot (Prunus armeniaca L.) Fruit Quality During Development and Ripening
Source: Front Plant Sci. 2020 Aug 19;11:1269. doi: 10.3389/fpls.2020.01269 (PMC7466674; doi:10.3389/fpls.2020.01269)
Supplement: Supplementary file 1 [file DataSheet_1.zip › FastQC_raw/F_S6_L002_R2_001_fastqc/fastqc_report.html]

F\_S6\_L002\_R2\_001.fastq FastQC Report


FastQC Report

dom 3 jun 2018  
F\_S6\_L002\_R2\_001.fastq

## Summary

- Basic Statistics
- Per base sequence quality
- Per sequence quality scores
- Per base sequence content
- Per base GC content
- Per sequence GC content
- Per base N content
- Sequence Length Distribution
- Sequence Duplication Levels
- Overrepresented sequences
- Kmer Content

## Basic Statistics

| Measure | Value |
| --- | --- |
| Filename | F\_S6\_L002\_R2\_001.fastq |
| File type | Conventional base calls |
| Encoding | Sanger / Illumina 1.9 |
| Total Sequences | 25111917 |
| Filtered Sequences | 0 |
| Sequence length | 101 |
| %GC | 45 |

## Per base sequence quality

## Per sequence quality scores

## Per base sequence content

## Per base GC content

## Per sequence GC content

## Per base N content

## Sequence Length Distribution

## Sequence Duplication Levels

## Overrepresented sequences

| Sequence | Count | Percentage | Possible Source |
| --- | --- | --- | --- |
| NNNNNNNNNNNNNNNNNNNNNNNNNNNNNNNNNNNNNNNNNNNNNNNNNN | 40958 | 0.1631018452314891 | No Hit |
| CGAGAAAATACAAAATGGGAGCTCAAACTTTCACAACCCCAACCTCAAAT | 29475 | 0.11737455169193176 | No Hit |

## Kmer Content

| Sequence | Count | Obs/Exp Overall | Obs/Exp Max | Max Obs/Exp Position |
| --- | --- | --- | --- | --- |
| CTCTC | 7259715 | 4.0351934 | 6.607753 | 1 |
| TCTCT | 8022265 | 3.5178907 | 5.6541104 | 7 |
| GAAGA | 8919070 | 3.1331956 | 7.986442 | 2 |
| TCTTC | 6397455 | 2.8053856 | 5.522253 | 7 |
| CTTCT | 5952635 | 2.610325 | 6.779408 | 1 |
| GAGAA | 6808190 | 2.391661 | 5.8914585 | 2 |
| GGAAG | 5487655 | 2.249881 | 5.659394 | 1 |
| CCCAA | 3952905 | 2.1479187 | 5.5183673 | 1 |
| CTTCA | 4926925 | 2.1361887 | 7.9552526 | 1 |
| CTCTG | 3823525 | 1.9347838 | 5.4599147 | 1 |
| CAACA | 4563430 | 1.9342461 | 6.0791287 | 1 |
| CTCCA | 3498025 | 1.92241 | 5.6361947 | 1 |
| TTCAA | 5573370 | 1.8849525 | 5.253624 | 2 |
| CTCAA | 4206370 | 1.803223 | 6.820937 | 1 |
| CTTTG | 4496970 | 1.795267 | 5.8438873 | 1 |
| GAAAA | 5833280 | 1.7558031 | 5.6432643 | 2 |
| TCCAA | 4066735 | 1.7433631 | 5.032791 | 7 |
| CTTGG | 3678760 | 1.6947037 | 5.412993 | 1 |
| CTCAG | 3119365 | 1.5606772 | 5.772023 | 1 |
| CTTGA | 3879945 | 1.5314859 | 6.0693603 | 1 |

Produced by FastQC (version 0.10.1)
